# Genome-wide discovery of DNA polymorphisms by whole genome sequencing differentiates weedy and cultivated rice

**DOI:** 10.1038/s41598-018-32513-z

**Published:** 2018-09-21

**Authors:** Chenglin Chai, Rama Shankar, Mukesh Jain, Prasanta K. Subudhi

**Affiliations:** 1 School of Plant, Environmental, and Soil Sciences, Louisiana State University Agricultural Center, Baton Rouge, LA 70803 USA; 2 School of Computational and Integrative Sciences, Jawaharlal Nehru University, New Delhi, 110067 India; 3 Present Address: Noble Research Institute, LLC, 2510 Sam Noble Parkway, Ardmore, OK 73401 USA

## Abstract

Analyzing the genome level DNA polymorphisms between weedy and cultivated rice is crucial to elucidate the molecular basis of weedy and agronomic traits, which in turn can enhance our ability to control weedy rice and its utilization for rice improvement. Here, we presented the genome-wide genetic variations between a weedy rice accession PSRR-1 and two cultivated rice accessions, Bengal and Nona Bokra, belonging to *japonica* and *indica* subspecies, respectively. The total number of SNPs and InDels in PSRR/Bengal was similar to that of Nona Bokra/Bengal, but was three times greater than that of PSRR/Nona Bokra. There were 11546 large-effect SNPs/InDels affecting 5673 genes, which most likely differentiated weedy rice from cultivated rice. These large effect DNA polymorphisms were mostly resulted in stop codon gain and least by start codon loss. Analysis of the molecular functions and biological processes of weedy rice specific SNPs/InDels indicated that most of these genes were involved in protein modification/phosphorylation, protein kinase activity, and protein/nucleotide binding. By integrating previous QTL mapping results with the DNA polymorphisms data, the candidate genes for seed dormancy and seed shattering were narrowed down. The genomic resource generated in this study will facilitate discovery of functional variants for weedy and agronomic traits.

## Introduction

Weedy rice (*Oryza sativa f. spontanea Rosh.)*, commonly known as red rice, is one of the most noxious weeds in rice growing areas worldwide^1,2^. It competes with cultivated rice for natural resources, leading to significant yield loss^3^. Unexpected mixing of weedy rice and cultivated rice grains during harvesting reduces the quality and marketability. The infestation of red rice in the Southern rice belt (a region includes four southern U.S. states, i.e., Arkansas, Louisiana, Mississippi and Texas, where a significant portion of the nation’s rice crop is grown) results in loss of 50 million dollars annually^4^. The management of weedy rice is particularly troublesome for rice growers. The persistence of weedy rice in the rice field can be due to early flowering, heavy seed shattering, and intense seed dormancy, which ensure continued presence of weedy rice seeds in soil seed bank^5^.

Genetic studies have indicated multiple mechanisms of weedy rice evolution with possible contribution from ancestral cultivated rice and wild rice, depending on different geographic regions worldwide^6–16^. Recent genome-wide analyses of DNA polymorphisms have further confirmed this possible origins^17–20^. It was suggested that weedy rice from Northeast Asia have evolved locally from *japonica* or *indica* varieties^20^ or as hybrids between modern *indica/indica*, or *japonica/japonica*^17^, whereas *aus*, *indica*, and wild rice have contributed toward evolution of weedy rice from South Asia^21^. Similarly, the mitochondrial genome analysis has suggested evolution of Korean weedy rice from cultivated rice^18^. There are two major genetically distinct groups of weedy rice in the United States such as the straw hulled (SH) and black hulled with long awns (BHA), which are believed to have originated in domesticated *indica* and *aus* rice background, respectively^13^. Recent studies involving both morphological data as well as whole genome sequences of weedy, cultivated, and wild rice have supported the evolution of US weedy rice by de-domestication^12,19^. It is evident from these above studies that US weedy rice has diverse origins which has been shaped by evolutionary forces and few genetic changes in domesticated backgrounds led to emergence of weedy attributes^13,19^. Although primarily considered as a destructive weed, weedy rice can be a valuable genetic resource for improving agronomically important traits including blast disease resistance^22^, rapid seedling growth^8^, photosynthetic rate and water use efficiency^23^, flowering time^24^, cold tolerance^25^, seed shattering^26^, and seed dormancy^27^.

Rice, feeding more than half of the world’s population, has been domesticated from wild ancestor approximately 10,000 years ago. During rice domestication, non-shattering and non-dormant rice accessions have been selected to avoid yield loss and asynchronous germination, respectively. However, a certain degree of seed shattering is preferred for easy grain threshing and likewise shallow seed dormancy is required to prevent pre-harvest sprouting (PHS), which adversely affects yield and grain quality^28,29^. Therefore, understanding the genetic basis of these traits generated a great deal of interest among plant geneticists, breeders, and weed scientists.

The degree of seed shattering varies greatly among different rice accessions. Wild rice (*O. rufipogon* and *O. nivara)* and weedy rice shed seeds very easily while the majority of cultivated rice show no or limited shattering^30^. Within cultivated rice, generally *indica* cultivars shatter seed easily than *japonica* cultivars^26^. The shattering trait in rice is controlled by the formation of an abscission layer^31,32^. Several genes responsible for seed shattering have been cloned in rice. *Sh4* is a major quantitative trait locus (QTL) that explained 69% of phenotypic variation between *indica* rice and the wild rice (*O. nivara*) and it encodes a transcription factor (TF) of trihelix family. *SH4* promotes hydrolysis of abscission zone and a nonsynonymous single-nucleotide polymorphism (SNP) in its Myb3 DNA binding domain leading to incomplete abscission zone (AZ) and reduced seed shattering^33,34^. A recent study on African rice has revealed role of *SH4* in controlling grain length^35,36^. Another QTL of seed shattering, *qSH1* encodes a member of homeobox TF with a SNP in 5′ regulatory region causing a failure in abscission layer formation^37^. A recessive shattering locus *sh-h* encoding a C-terminal domain phosphatase-like protein has been shown to repress AZ formation^38^. A transcription factor, *SHAT1*, which is required for AZ development and functions down-stream of *Sh4* and *qSH1*, has been identified^39^. Several research groups have identified QTLs for seed shattering on all chromosomes except 9 and 10 using populations derived from crosses between cultivated rice and different weedy rice accessions^8,40–42^. Genetic and genomic studies on seed shattering using cross between cultivated rice and wild rice (*O. rufipogon*) have also been conducted^43,44^. Recently, our laboratory has reported 3–5 QTLs controlling seed shattering with 38–45% of the phenotypic variation in two recombinant inbred line (RIL) mapping populations involving the US weedy rice accession PSRR-1 and two US *japonica* varieties^26^. Although the largest QTL on chromosome 4 overlapped with the *Sh4*, the presence of the non-shattering SNP allele in the weedy rice accession suggested involvement of a linked locus^26^ or alternative genetic mechanisms^45^.

Seed dormancy refers to the inability of viable seeds to germinate under favorable conditions^46^. Seed dormancy, established and maintained during seed maturation, is gradually broken during dry storage due to after-ripening^47^. It is a complex trait controlled by multiple genes with strong influence of environmental factors^48^. Despite the fact that seed dormancy plays a critical role in environmental adaptation for wild species and is a trait of agronomic importance, the underlying molecular basis is not yet clearly elucidated. Genetic and molecular analyses in *Arabidopsis* have revealed the role of chromatin modification in controlling seed dormancy through cloning and functional analysis of *HUB1* (also known as *RDO4*) and *LDL1* and *LDL2*^47,49^. *DOG1*, encoding a protein with unknown function, has been suggested to play a key role in the onset of seed dormancy^50^. *LDL1* and *LDL2* worked redundantly in repressing seed-dormancy related genes including *DOG1*. In rice, a QTL for seed dormancy, *Sdr4*, was shown to contribute substantially to the difference in seed dormancy between *japonica* and *indica* cultivars^51^. *Sdr4* expression was positively regulated by a global regulator of seed maturation *OsVP1* and acted as an intermediate regulator of dormancy in the seed maturation program. Few studies have been conducted to detect QTL for seed dormancy in weedy rice^27,52^ and a pleiotropic gene *Rc* was responsible for both seed dormancy and pericarp color^53^. Another gene controlling endosperm-imposed dormancy was involved in gibberellin synthesis^54^. Previously, we have detected 6–7 QTL for seed dormancy in two RIL populations developed from the crosses involving a weedy rice accession (PSRR-1) and these QTLs accounted for ~50% of the total phenotypic variation^27^. One of the QTL overlapped with *Sdr4*, however the nucleotide polymorphisms for the variation in seed dormancy could not be validated in our materials.

Based on our previous QTL mapping results on seed shattering and seed dormancy^26,27^, we continued to peruse the genetic basis underlying these two important agronomic traits by taking advantage of next-generation sequencing technology. More importantly, we report here the genome-wide genetic variation of weedy rice to generate genomic resources for discovery of genes associated with both weedy and agronomic traits. The objectives of the current study were (i) to identify genome-wide nucleotide polymorphisms between two rice cultivars and weedy rice, which will be useful for improving agriculturally important traits, and (ii) to identify candidate genes for seed shattering and seed dormancy by integrating our rice whole-genome re-sequencing data with QTL mapping data.

## Results and Discussion

Two cultivated rice (*O. sativa*) (a tropical *japonica* cultivar, Bengal, and an *indica* cultivar, Nona Bokra) and one straw hulled (SH) weedy rice accession (*O. sativa*) (PSRR-1) with contrasting phenotypes of seed shattering and seed dormancy^26,27,55^ were selected for the analysis of genomic variation. PSRR-1 showed higher degree of shattering compared to Bengal and Nona Bokra. Both PSRR-1 and Nona Bokra are intensely dormant compared to non-dormant Bengal.

### Genome re-sequencing and reads mapping

We obtained a total of 307,009,538 paired-end reads and 287,967,294 high quality (HQ) filtered reads from the three genotypes. The percentage of HQ filtered reads ranged from 92% to 95% (Table 1) and all HQ filtered reads were used for mapping. About 92–99% of these reads were successfully mapped to Nipponbare reference genome, covering 92–97% of rice genome. Of the total reads, 94–97% reads were mapped to unique locations in the rice genome (Table 1). All the uniquely mapped reads were used for down-stream data analysis. The Illumina FASTQ files for PSRR-1, Bengal, and Nona Bokra were submitted to the sequence read archive (SRA) at NCBI with the accession numbers PRJNA413818, PRJNA413821, and PRJNA413822, respectively.

**Table 1:** Summary of mapping information of the three rice accessions in this study.

	PSRR-1	Bengal	Nona Bokra
Total number of reads	136,721,696	104,386,506	65,901,336
Sequencing depth (fold)	38.88	28.18	17.78
Total number of HQ filtered reads	128,490,292	98,771,468	60,705,534
HQ filtered reads (%)	93.98	94.64	92.12
Total number of reads mapped	117,696,058	98,132,017	59,995,162
Total reads mapped (%)	91.60	99.35	98.83
Genome coverage (%)	91.94	96.60	92.17
Unique reads mapped	110,072,797	95,208,366	56,863,974
Unique reads mapped (%)	93.52	97.02	94.78

### Identification of SNPs and InDels

SNPs and InDels between Bengal and Nona Bokra (Bengal/Nona Bokra), PSRR-1 and Bengal (PSRR/Bengal), and PSRR-1 and Nona Bokra (PSRR/Nona Bokra), were identified (Table 2). Overall, PSRR/Bengal and Bengal/Nona Bokra showed similar numbers of DNA polymorphisms, which were about 2~3 times higher compared with PSRR/Nona Bokra. The total numbers of SNPs for PSRR/Bengal and Bengal/Nona Bokra were 1,704,184 and 1,414,468, respectively, while that of SNPs for PSRR/Nona Bokra was 632,302. However, the number of InDels was significantly lower than that of SNPs for each of the comparisons. The total numbers of InDels were 85,016 and 102, 242 for PSRR/Bengal and Bengal/Nona Bokra, respectively, whereas the number was 36,163 for PSRR/Nona Bokra. Furthermore, the densities of SNPs and InDels for PSRR/Bengal and Bengal/Nona Bokra were also similar, approximately two to three times higher than that of PSRR/Nona Bokra. Among the cultivated rice, genetic differentiation between *indica* and *japonica* subspecies is well established. Our study showed that PSRR-1 was genetically much closer to *indica* cultivar ‘Nona Bokra’ compared to the *japonica* cultivar ‘Bengal’ based on both total number and density of genome-wide SNPs and InDels. This observation as well as earlier studies^3,11,56^ are in clear agreement with recent reports regarding evolution of straw hulled US weedy rice from *indica* cultivars through de-domestication^19^. Our high-density genetic markers across the whole rice genome could be useful in both theoretical and applied genetics such as genotyping, linkage disequilibrium studies, gene cloning, and marker-assisted breeding.

**Table 2:** Frequency of SNPs and InDels detected in PSRR-1, Bengal, and Nona Bokra.

	SNPs		InDels	
	Total	Per 100 kb	Total	Per 100 kb
PSRR/Bengal	1,704,184	455.1	85,016	22.7
PSRR/Nona Bokra	632,302	168.9	36,163	9.7
Bengal/Nona Bokra	1,414,468	377.7	102,242	27.3

### Nonrandom genomic organization of DNA polymorphisms

**Figure 1:**
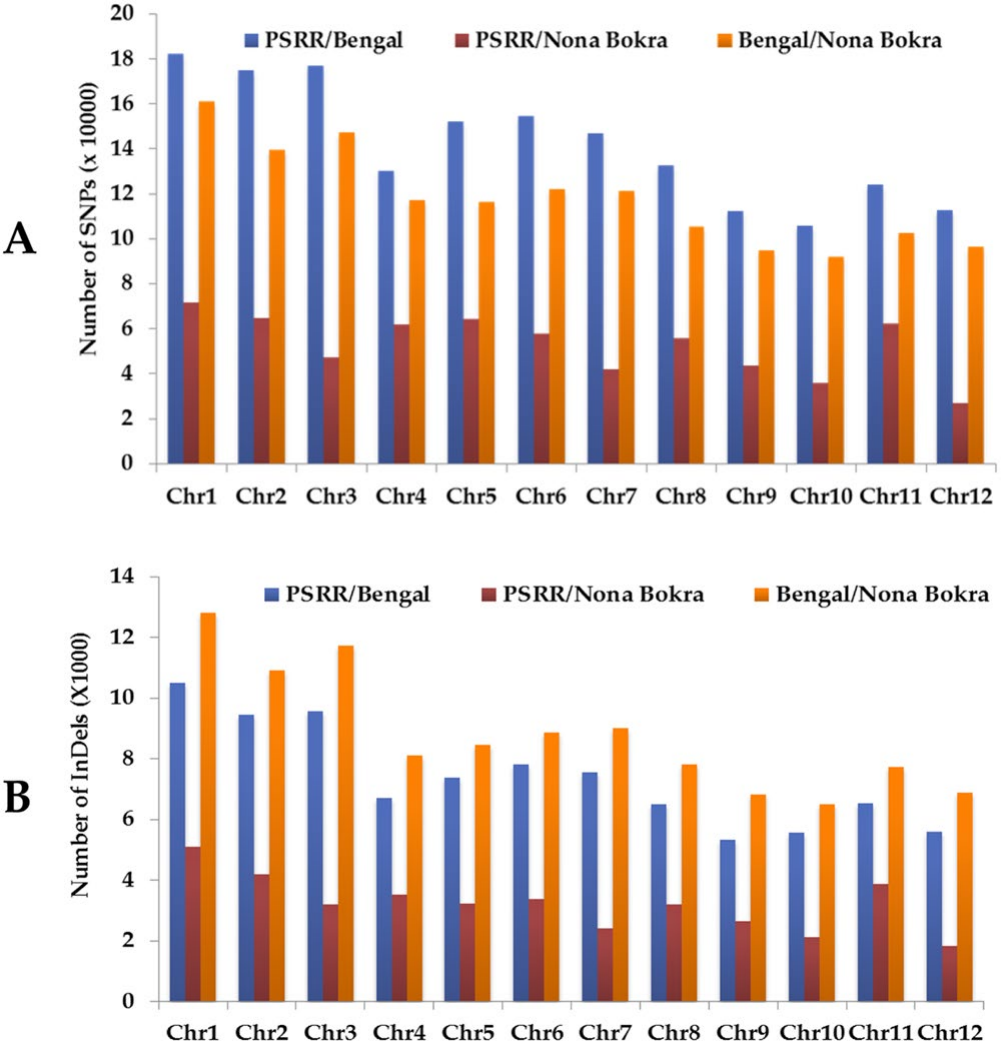
Total numbers of SNPs (**A**) and InDels (**B**) in PSRR/Bengal, PSRR/Nona Bokra, and Bengal/Nona Bokra detected on 12 rice chromosomes. PSRR/Bengal, PSRR/Nona Bokra, and Bengal/Nona Bokra refer to SNPs/InDels identified among these three varieties, respectively.

The genomic organization of DNA polymorphisms was investigated among PSRR-1 and two cultivars (Bengal and Nona Bokra) across all 12 rice chromosomes. The number of identified SNPs and InDels displayed considerable variations across chromosomes (Fig. 1). For both PSRR/Bengal and Bengal/Nona Bokra, the total number of DNA polymorphisms (SNPs and InDels) on each chromosome was proportional to the size of the chromosome (Fig. 1A, B, Supplementary Tables S1, S2). The SNPs and InDels were most abundant in chromosomes 1, 2, and 3, and less abundant in chromosomes 9, 10, and 12. However, PSRR/Nona Bokra showed different pattern of DNA polymorphism distribution among chromosomes: the SNPs were most abundant in chromosomes 1, 2, and 5 and InDels in chromosomes 1, 2, and 11; while SNP and InDel were scarce in chromosomes 7, 10, and 12 (Fig. 1A,B; Supplementary Tables S1, S2).

**Figure 2:**
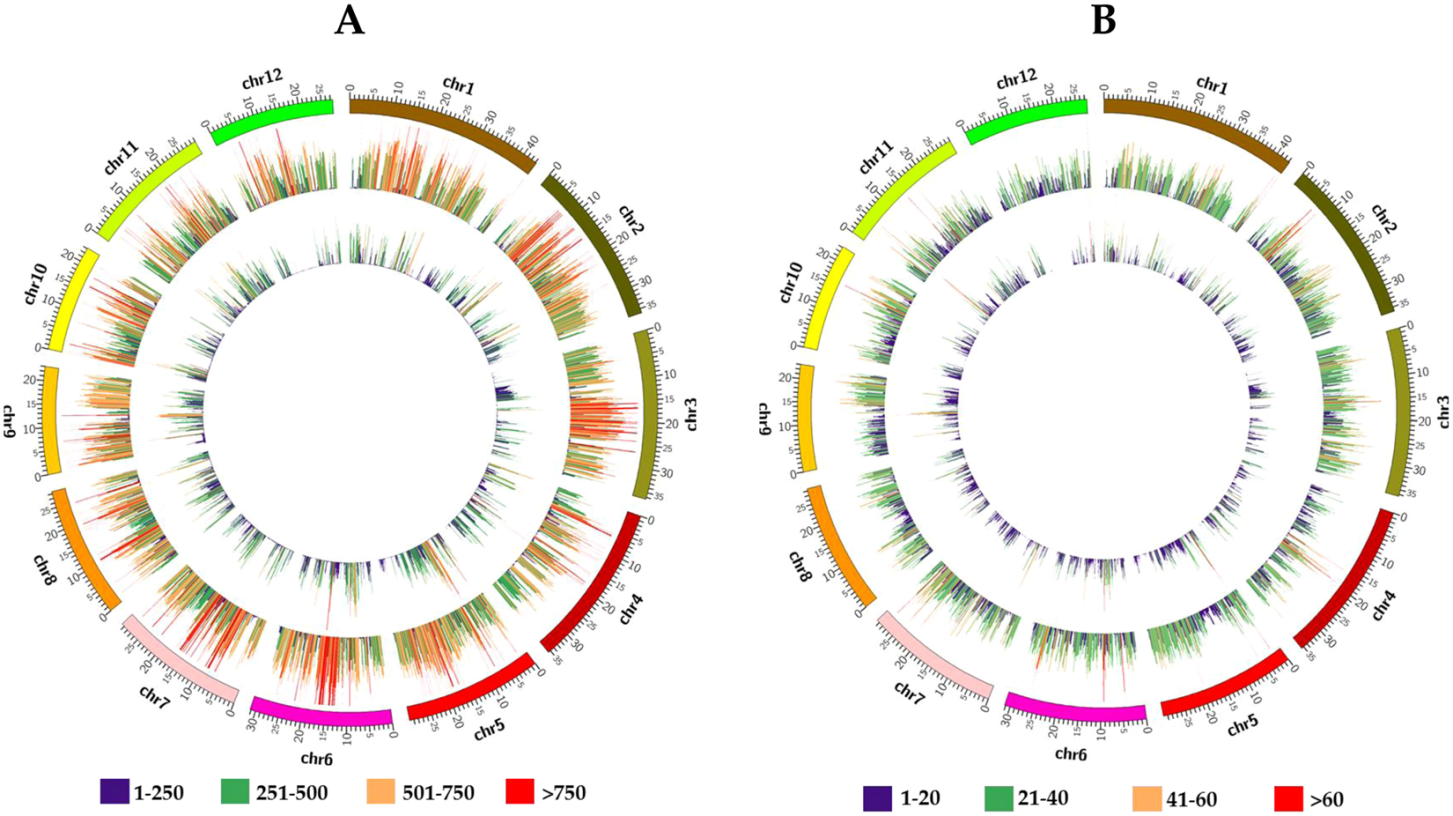
Distribution of SNPs (**A**) and InDels (**B**) in PSRR/Bengal and PSRR/Nona Bokra on each rice chromosome (100 kb window size). The outermost circle represents 12 rice chromosomes in different colors; the middle and innermost circle represents SNP/InDel distribution in PSRR/Bengal and PSRR/Nona Bokra, respectively. Different colors represent different ranges of SNPs and InDels.

The distributions of SNPs and InDels within chromosomes in PSRR-1, Bengal, and Nona Bokra were not uniform (Fig. 2; Supplementary Tables S3, S4). Overall, more DNA polymorphisms were distributed in PSRR/Bengal, compared with those between PSRR/Nona Bokra. The number of high-density (≥250) SNP regions of 100 kb for PSRR/Bengal and PSRR/Nona Bokra were 2962 and 1132, respectively (Fig. 2A; Supplementary Table S3). Similarly, 51 and 582 low-density (≤5) SNP regions of 100 kb were detected for PSRR/Bengal and PSRR/Nona Bokra, respectively. Interestingly, we found 1783 and 244 SNP “hotspots” with extremely high density (≥1000 SNPs/100 kb) for PSRR/Bengal and PSRR/Nona Bokra, respectively. The InDels were not evenly distributed within chromosomes (Fig. 2B; Supplementary Table S4). We found 434 and 77 InDel rich (≥40) regions for PSRR/Bengal and PSRR/Nona Bokra, respectively. Likewise, low-frequency InDel regions were also detected. The significantly differential distribution of DNA polymorphisms has been documented in many plants including rice^57–60^.

### Analysis of SNPs and InDels

**Figure 3:**
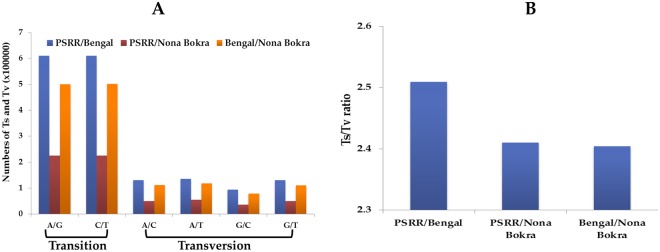
Number of substitutions types (**A**) and Ts (transition)/Tv (transversion) ratio (**B**) in the identified SNPs in PSRR/Bengal, PSRR/Nona Bokra, and Bengal/Nona Bokra.

We further investigated the total numbers of transition (Ts) and transversion (Tv) for PSRR/Bengal, PSRR/Nona Bokra, and Bengal/Nona Bokra (Fig. 3A). The total numbers of Ts (A/G and C/T) were significantly higher than those of Tv (A/C, A/T, C/G, and G/T) for all three pairs. The total number of each type of Ts and Tv was nearly similar in PSRR/Bengal and Bengal/Nona Bokra, but was 2~3 times higher than that of PSRR/Nona Bokra. Overall, PSRR/Bengal and Bengal/Nona Bokra had similar frequency of each type of SNPs, which were 2~3 times higher than that of PSRR/Nona Bokra. The frequencies of A/G were at similar level as C/T in all cases. However, the frequencies of Tv were not at the similar level; the frequency of C/G was lower than the other three types of Tv, which were at the similar level. The ratio of Ts/Tv of PSRR/Bengal (~2.5) was slightly higher than those of PSRR/Nona Bokra and Bengal/Nona Bokra (~2.4), which showed different pattern as PSRR/Bengal was grouped with Bengal/Nona Bokra (Fig. 3B). The higher Ts/Tv (termed as transition bias), which had been reported in rice and maize^61,62^, was caused by a higher frequency of Ts mutations over Tv mutations (due to conformational advantage in case of mispairing) and better tolerance to Ts changes because of less chance of changing protein structures/functions compared with Tv^59,63^. Our results were consistent with previous reports from rice and other plants^60,64^.

The length distributions of InDels identified among three rice accessions were analyzed (Supplementary Fig. S1). The size of insertions ranged from 1 to 16 for PSRR/Nona Bokra and 1 to 19 for PSRR/Bengal and Bengal/Nona Bokra. In all cases, the number of insertions was negatively correlated with the length of the insertions, i.e., most insertions (73~74%) involved single nucleotide followed by two nucleotides (14~15%) and three nucleotides (4~6%) and so on in a decreasing order, which led to the majority of insertions (97~98%) being 1 to 4 nucleotides in length. The longest insertion (16 or 19 nucleotides long) was only 0.003~0.007% of the total insertions. The deletions among the three rice accessions ranged from 1 to 32 nucleotides long and showed a similar pattern of distribution as insertions with the largest proportion of deletions (66~68%) with one nucleotide and the smallest proportion (~0.002%) with 32 nucleotides (Supplementary Fig. S1). Although the length distribution of InDels observed in this study was consistent with previous studies^59,60^, the maximum length of InDels was greater in this study, which may be due to use of different rice accessions.

### Annotation of DNA polymorphisms

**Figure 4:**
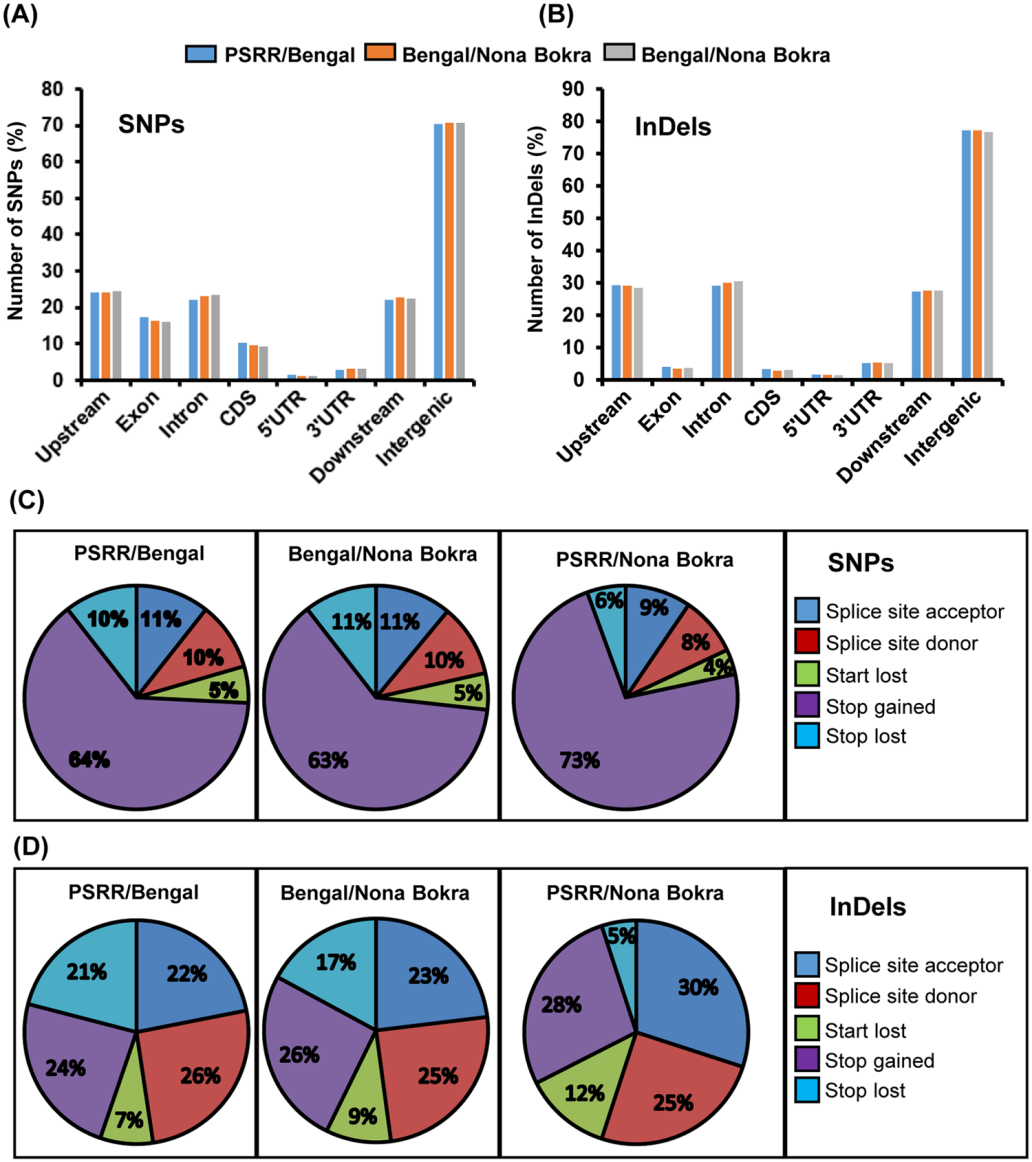
Annotation of SNPs and InDels. (**A**) Distribution of SNPs in different genomic regions; (**B**) Distribution of InDels in different genomic regions; (**C**) Distribution of large-effect SNPs in different genic regions; (**D**) Distribution of large-effect InDels in different genic regions.

The location and nature of DNA polymorphisms are known to influence gene expressions and functions that govern various biological processes^26,34,51^. We conducted genome-wide annotation of the SNPs and InDels identified in different genomic regions (Fig. 4). In general, the patterns of SNPs and InDels in different genomic regions were quite similar for all comparisons, though the number of variants in PSRR/Bengal and Bengal/Nona Bokra was much higher than that of PSRR/Nona Bokra. A genic region was defined as the region between the transcription start site and the end of 3′ UTR^65,66^. For all three pair-wise comparisons, SNPs occurred more frequently in noncoding regions (including intergenic regions, 5′ UTR, 3′ UTR, and introns) than in the coding regions (Fig. 4A). High frequency of genetic variants in the noncoding regions could result from less pressure from natural selection and/or domestication in these regions^67^. However, DNA polymorphisms in these regions were reported to play important role during evolution and domestication. For example, some causal mutations responsible for important agriculturally important traits such as seed shattering^37^ and pre-harvest sprouting^29^ occurred in intergenic region and intron, respectively. In case of InDels, the highest frequencies were also found in the intergenic regions and the lowest frequencies were detected in coding regions for all pair-wise comparisons (Fig. 4B). Since large-effect genetic variants cause non-functional proteins leading to various phenotypic changes during evolution, we were prompted to investigate large-effect SNPs and InDels among the three rice genotypes in this study. The large-effect variants include disruption of splicing sites, loss of translation start codon, and introduction of premature stop codon. The non-synonymous SNPs and large-effect InDels only accounted for 5~6% and 1~2% of total polymorphisms, respectively (Fig. 4C,D). The high frequency of large-effect SNPs and InDels were in the coding regions (CDS) and low frequency of large-effect SNPs and InDels were found in the intergenic regions. For all the comparisons, large-effect SNPs in the intergenic regions largely resulted from stop codon gain (~64% for PSRR/Bengal and Bengal/Nona Bokra, and 73% for PSRR/Nona Bokra) and least from start codon loss (5% for PSRR/Bengal and Bengal/Nona Bokra, and 4% for PSRR/Nona Bokra) (Fig. 4A,C). In contrast, the frequencies of large-effect InDels in the intergenic regions were relatively even among different origin of variants (Fig. 4B,D). The patterns of large-effect InDels of PSRR/Bengal and Bengal/Nona Bokra were quite similar, with the highest percentage of large-effect InDels occurred either in the 5′ end of introns (splicing donor site) or through gain of stop codons and the lowest percentage of large-effect InDels occurred through lost start codons. For PSRR/Nona Bokra, however, the greatest portion of large-effect InDels were in the 3′ end of introns (splicing acceptor site) and the smallest portion of large-effect InDels was caused by loss of stop codon.

### Validation of SNPs and InDels

The reliability of the DNA polymorphisms on a global scale is a prerequisite for various genome-wide studies. To experimentally validate SNPs and InDels identified in this study, we sequenced the PCR amplified DNA fragments harboring randomly selected 27 variants including seven SNPs, eight insertions, and 12 deletions. About 92% of selected variants were validated successfully by this approach (Supplementary Table S5). This high validation rate suggested high reliability of the identified DNA polymorphisms with great potential. Since PSRR-1 and Nona Bokra have been demonstrated to be a reservoir of genes for improving agronomic traits as well as for understanding the domestication process^25–27,57,68,69^, the genome-wide DNA polymorphism resources will be useful in future studies.

### Functions of large-effect DNA polymorphism

**Figure 5:**
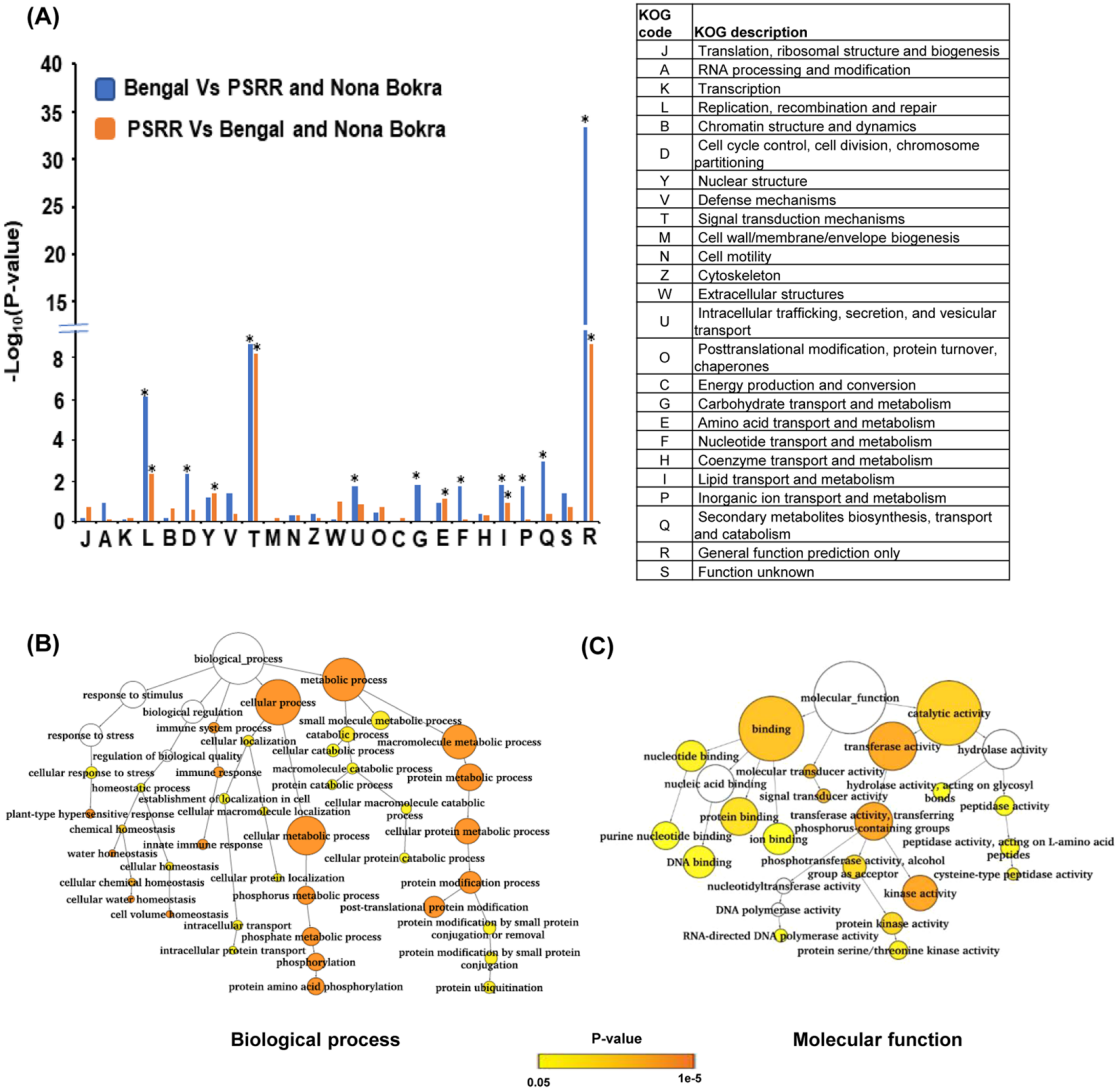
Functional classification of genes identified with nonsynonymous single-nucleotide polymorphisms (SNPs) or large-effect SNPs/InDels specific to PSRR and Bengal. (**A**) Distribution of the eukaryotic orthologous group (KOG) classes in the genes identified with nonsynonymous SNPs or large-effect SNPs/InDels (Significant differences (P ≤ 0.03) are marked with asterisks). (**B**) GO enrichment analysis of genes with nonsynonymous/large-effect SNPs and InDels specific to PSRR-1 showing involvement in possible biological processes. (**C**) GO enrichment analysis of genes with nonsynonymous/large-effect SNPs and InDels specific to PSRR showing the possible molecular functions. Node size is proportional to the number of transcripts in each category and the shaded colors indicate different significance level: white represents no significant difference; orange color represents significant level at P-value < 0.0000005; yellow represents significant level at P-value < 0.05.

In this study, we identified 11546 nonsynonymous/large-effect SNPs/InDels that were specific to PSRR-1 (not present in Bengal or Nona Bokra), affecting about 5673 genes (Supplementary Table S6). In order to investigate their putative functions affected in weedy rice PSRR-1 compared to Bengal and Nona Bokra, eukaryotic orthologous group (KOG) analysis was conducted (Fig. 5A). Besides genes with general and unknown functions, genes involved in ‘signal transduction’, ‘amino acid transport and metabolism’, and ‘lipid transport and metabolism’ were significantly enriched. The functions of genes were further investigated by gene ontology (GO) analysis (Fig. 5B,C). Genes involved in biological processes such as ‘protein modification/ phosphorylation’ were over-represented (Fig. 5B). Analysis at molecular function level revealed that genes involved in ‘protein kinase activity’ and ‘protein/nucleotide binding’ were significantly represented (Fig. 5C). Those large-effect SNPs/InDels and non-synonymous SNPs might be, to some extent, responsible for the contrasting phenotypes (including seed shattering and dormancy) between weedy rice and cultivated rice.

We were prompted to explore biological insight of those DNA variants. Rice is sensitive to cold and low temperature stress negatively affects early establishment and eventual grain yield^70^. PSRR was tolerant to cold stress at germination stage, while Bengal was susceptible to low temperature^25^. A recent genome-wide association study on cold tolerance at germination stage has revealed 42 cold tolerance QTLs and corresponding candidate genes^71^. By searching PSRR specific SNPs/InDels, we identified two SNPs responsible for nonsense and missense mutations in two cold tolerance candidate genes (LOC_Os01g02750, a putative protein kinase, and LOC_05g36240, an unknown protein), respectively, which are among the candidate gene list (Supplementary Table S6, Supplementary Figs 2 and 3). These candidate genes need to be functionally characterized for their role in improving cold tolerance. Using a similar approach, we also found a candidate gene (LOC_Os11g45980, encoding an NBS-LRR type disease resistance protein) for blast disease resistance in rice (Supplementary Table S6, Supplementary Fig. S4). This gene candidate was close to one of the QTLs associated with blast disease resistance^72^ and harbors a PSRR specific nonsynonymous SNP (Supplementary Table S6, Supplementary Fig. S4). Bengal is reported to be susceptible to blast disease^73^ while nearly 50% of US weedy rice accessions are resistant to this disease^72^. It will be interesting to investigate if PSRR is resistant to the blast disease, and if so, to explore the function of this candidate gene. However, to fully understand biological functions of all the over-enriched genes harboring PSRR specific SNPs/InDels, which may account for the many contrasting traits of agronomic importance between PSRR and cultivated rice, it is imperative to identify/characterize those traits and incorporate other data such as QTL mapping, transcriptomic profiling, and genetic complementation for each trait to narrow down the candidate genes and confirm their role in expression of those desirable traits.

When genes harboring SNPs/InDels in promoter regions were considered for GO enrichment analysis, similar functional categories were represented (Supplementary Fig. S5). But when KOG analysis was done for genes harboring nonsynonymous SNPs/InDels and SNPs/InDels in the promoters of the genes in the QTL regions (*Sh4* region for shattering and *qSD7-1* for seed dormancy), there was difference in enrichment pattern for these traits (Supplementary Fig. S6). For seed dormancy, Bengal was contrasted against PSRR-1 and Nona Bokra, which were both dormant. Similarly, GO enrichment analysis was done for genes affected in *sh4* region of PSRR-1 in relation to Bengal and Nona Bokra (both non shattering types). Significant enrichment was observed for genes involved in translation, ribosomal structure and biogenesis, and transcription for seed dormancy attribute; whereas genes involved in amino acid transport and metabolism, coenzyme transport, and metabolism were enriched for seed shattering. GO enrichment analysis revealed carbohydrate metabolism and dephosphorylation biological processes for both traits. Substrate specific and transmembrane transporter activity were important for seed dormancy (Supplementary Fig. S7) whereas meiotic cell cycle, cellular response to stimulus, beta xylanase and glucosyl transferase were enriched for seed shattering QTL (Supplementary Fig. S8).

### Candidate genes for seed shattering and seed dormancy

Previously we have identified QTL for both seed shattering and seed dormancy using two recombinant inbred line (RIL) populations developed from the crosses involving rice cultivars (Bengal and Cypress) and the same weedy rice accession PSRR-1 used in this study^26,27^. Although the major QTL for seed shattering *qSH4* overlapped with known shattering gene *Sh4*, the presence of non-shattering SNP allele in the weedy rice suggested that another gene nearby might be responsible for the shattering phenotype in weedy rice^26^. Similarly, one of the major QTL for seed dormancy overlapped with known dormancy gene *Sdr4*, but the non-dormant allele in weedy rice indicated involvement of other gene(s)^27^. To further explore the genetic basis of these two traits, we narrowed down the candidate genes by linking large-effect DNA polymorphisms with predicted functional and agronomic relevance using the next generation sequencing (NGS) data of the parents (Bengal, Nona Bokra, and PSRR-1).

For seed shattering, the *qSH4* was mapped to a region on chromosome 4 between two SSR markers RM5506 and RM127, which is about 1.2 Mb in physical size (Chr4: 33307270.0.34529722 bp interval) and harbors 254 genes^26^. By filtering out low-impact genetic variances, we identified 15 non-synonymous/large-effect SNPs between PSRR-1 (shattering phenotype) and two non-/reduced shattering cultivars (Bengal and Nona Bokra), which were distributed in 8 genes (Table 3). None of the listed genes was among those reported genes controlling seed shattering, pod dehiscence, or fruit shedding suggesting a new genetic mechanism. More experimental evidence and/or bioinformatics prediction including expression profiling and genetic complementation will be needed to identify and functionally characterize the candidate genes.

**Table 3:** Unique SNP/InDel and candidate genes for seed shattering in *qSH4* QTL region.

Gene #	SNP location	PSRR-1	Bengal/Nona Bokra	Type of mutation	Locus ID	Putative function
1	Chr4: 33665181	C	T	start gain	LOC_Os04g56480	Pelota
1	Chr4: 33666866	G	A	missense	LOC_Os04g56480	Pelota
2	Chr4: 33489119	A	G	missense	LOC_Os04g56170	SBP-box
2	Chr4: 33489148	C	A	missense	LOC_Os04g56170	SBP-box (TF, control of early flower development)
3	Chr4: 33634394	G	A	start gain	LOC_Os04g56405	Expressed protein (predominantly expressed in developing seeds and SAM)
3	Chr4: 33632704	T	A	missense	LOC_Os04g56405	Expressed protein
3	Chr4: 33633208	A	G	missense	LOC_Os04g56405	Expressed protein
3	Chr4: 33633367	A	G	missense	LOC_Os04g56405	Expressed protein
3	Chr4: 33633406	A	G	missense	LOC_Os04g56405	Expressed protein
3	Chr4: 33633746	A	G	missense	LOC_Os04g56405	Expressed protein
4	Chr4: 33659169	A	C	missense	LOC_Os04g56460	BTB/POZ domain (mediates homomeric dimerisation and in some instances heteromeric dimerization, expressed on later seed developmental stages)
5	Chr4: 33662010	A	G	missense	LOC_Os04g56470	Amino acid transporter (expressed in inflorescence),
6	Chr4: 34091958	A	C	missense	LOC_Os04g57230	RecX
7	Chr4: 34274774	T	C	missense	LOC_Os04g57600	Zinc-finger
8	Chr4: 34210040	T	C	splice site	LOC_Os04g57500	Phosphatidate cytidylyltransferase

For seed dormancy, the major QTL *qSD7-2*^*BR*^ was mapped to a 4.5 Mb region between two molecular markers^27^. Twenty-one genetic variants were unevenly distributed in 11 genes (Table 4). Among these genes, at least two genes were identified that may play major role in the control of seed dormancy. The nonsynonymous SNP in the 13th exon of LOC_Os07g10490, which is annotated as a zeta-carotene desaturase, caused an amino acid change from Arginine in dormant genotypes ‘PSRR-1’ and ‘Nona Bokra’ to Glutamine in non-dormant cultivated rice ‘Bengal’ (Supplementary Fig. 9). The zeta-carotene desaturase was a key enzyme in carotenoid biosynthesis and carotenoids serves as precursors in ABA biosynthesis. Mutation in this gene caused ABA deficiency leading to decreased seed dormancy/preharvest sprouting phenotype. The other gene, *Rc*, which showed a 14-bp deletion within exon 6 in cultivated rice^74^, was also identified in our study. The 14-bp deletion was found in *Rc* gene of non-dormant white-pericarp cultivar Bengal, but not in that of the dormant red pericarp weedy rice PSRR-1 and *indica* cultivar Nona Bokra. The *Rc* gene was recently reported to play pleiotropic role controlling both seed dormancy and pericarp color^53^. In this study, we identified few other candidates for further investigation to unambiguously associate a gene with seed dormancy using a different approach. More importantly, this study demonstrated that combining mapped QTL with whole genome sequence data could be a reliable approach for gene identification.

**Table 4:** Unique SNP/InDel and candidates in seed dormancy QTL (*qSD7-2*) region.

Gene #	Variant location	Bengal	PSRR/Nona Bokra	Type of mutation	Locus ID	Putative function
1	Chr7: 5650154	A	G	missense	LOC_Os07g10490	zeta-carotene desaturase
1	Chr7: 5649936	G	A	splice site	LOC_Os07g10490	zeta-carotene desaturase
2	Chr7: 5777725	C	T	start gain	LOC_Os07g10630	expressed protein
2	Chr7: 5775277	A	G	missense	LOC_Os07g10630	expressed protein
3	Chr7: 5786357	C	T	missense	LOC_Os07g10650	hypothetical protein
3	Chr7: 5786557	G	A	missense	LOC_Os07g10650	hypothetical protein
4	Chr7: 5787795	A	G	missense	LOC_Os07g10660	ribosomal protein
5	Chr7: 5801339	T	G	missense	LOC_Os07g10680	polygalacturonase
5	Chr7: 5801501	T	C	missense	LOC_Os07g10680	polygalacturonase
5	Chr7: 5802260	G	A	missense	LOC_Os07g10680	polygalacturonase
6	Chr7: 5812776	C	T	missense	LOC_Os07g10700	polygalacturonase
6	Chr7: 5813434	C	G	missense	LOC_Os07g10700	polygalacturonase
6	Chr7: 5813797	C	T	missense	LOC_Os07g10700	polygalacturonase
6	Chr7: 5813766	G	T	splice site	LOC_Os07g10700	polygalacturonase
7	Chr7: 5817353	A	G	missense	LOC_Os07g10710	F-box protein
8	Chr7: 5823118	T	C	missense	LOC_Os07g10730	polygalacturonase
9	Chr7: 5828464	C	T	missense	LOC_Os07g10740	polygalacturonase
10	Chr7: 6039051	C	T	missense	LOC_Os07g11000	structural constituent of ribosome
10	Chr7: 6039626	A	G	missense	LOC_Os07g11000	structural constituent of ribosome
10	Chr7: 6041779	T	G	splice site	LOC_Os07g11000	structural constituent of ribosome
11	Chr7: 6068071	A	AACGCGAAAAGTCGG	frame shift	LOC_Os07g11020	*Rc*

## Conclusions

Weedy rice is a promising valuable genetic resource for rice improvement due to its fitness advantage, early flowering time, and biotic and abiotic stress tolerance. Despite its morphological similarity with cultivated rice, differences between weedy and cultivated rice at whole genome level shed some light on the genome organization in weedy rice compared to the cultivated rice. High degree of similarity of weedy rice to *indica* cultivar revealed through genome-wide DNA polymorphisms suggested that it might have originated from *indica* rice. Majority of SNPs/InDels were present in intergenic regions. Gain of stop codon was more prevalent compared to start codon loss resulting non-synonymous and large effect SNPs and InDels. Combining our earlier QTL mapping results with the NGS data, candidate genes for two QTLs *Sh4* and *qSD7-2* were narrowed down. Genome-wide DNA polymorphisms reported here will now facilitate discovery of functional variants associated with important agronomic traits. The genomic resources generated in this study will accelerate both molecular genetics and molecular breeding investigations in rice.

## Materials and Methods

### DNA sample preparation and sequencing

Genomic DNA was extracted from leaves of two-week old seedlings of two cultivated rice (Bengal and Nona Bokra) and a weedy rice accession (PSRR-1) using Qiagen DNeasy kit (Qiagen Inc., Valencia, CA, USA). Bengal is medium grain high yielding non-dormant *japonica* rice cultivar with reduced seed shattering released by the Louisiana State University Agricultural Center^75^. Nona Bokra is a salt tolerant land race from India belonging to *indica* subspecies of rice with tall plant stature, red pericarp, non-shattering, and strong seed dormancy. Nona Bokra has been used to map seed dormancy QTLs^55^. PSRR-1 was collected from the Rice Research Station at Crowley, LA and was purified by single plant selection for two generations before its use for developing mapping populations and sequencing. It has light green leaves, vigorous growth, long auricles and ligules, straw-hulled medium grains, lax open panicles, and pubescent leaves. PSRR-1 is extremely susceptible to shattering and has a higher intensity of both hull and pericarp dormancy compared to Bengal^26,27^. The quality and quantity of DNA samples were analyzed by Bioanalyzer 2100 (Agilent Technologies, Singapore) and Qubit 2.0 Fluorometer (Invitrogen Life Technologies, Eugene, Oregon), respectively. The libraries were prepared using Illumina TruSeq DNA sample preparation kit (Illumina, USA) according to the manufacturer’s protocol and paired-end sequencing was performed in an Illumina Hiseq 2000 at the Virginia Bioinformatics Institute, Blacksburg, VA, for generating 101-bp long reads. The generated raw data were filtered using an in-built standard Illumina pipeline.

### Read quality checking and read mapping

The filtered reads from the Illumina pipeline were further processed using NGS QC Toolkit (v2.3.3; http://www.nipgr.res.in/ngsqctoolkit.html) to remove primer/adopter sequences and low quality reads; Phred quality score <30) and only high-quality reads (Phred quality score ≥30) were used for mapping^76^. Mapping of the high-quality filtered reads on the rice reference genome (MSU7 version; http://rice.plantbiology.msu.edu/index.shtml) was performed using Burrows-Wheeler Alignment (BWA) software (v0.7.12; http://bio-bwa.sourceforge.net/)^77^. Coverage of the reference genome was estimated using SAMtools (v1.1; http://samtools.sourceforge.net/)^78^.

### Detection and analysis of SNPs and InDels

FreeBayes software (v0.9.21; https://github.com/ekg/freebayes) was used for the identification of SNPs and InDels using three criteria: the minimum variant frequency of ≥90%, average quality of the SNP base ≥30, and minimum read depth of 10. Additional filtering of SNPs and InDels was performed when there were three or more SNPs/InDels in any 10-bp window^79^. The frequency of SNPs/InDels in each 100 kb interval on each rice chromosome was calculated to reveal the genome-wide distribution of polymorphisms. Circos^80^ was used to visualize the distribution of DNA polymorphisms on rice chromosomes. The distribution of DNA polymorphisms in different genomic regions was evaluated by integrating the positions of DNA polymorphisms with GFF file. Analyses including identification, genomic distribution, and annotation of DNA polymorphisms (synonymous/nonsynonymous SNPs), and large-effect SNPs/InDel were performed using SnpEff (v4.1k)^81^ using default parameters. We used sequence of 2 kb upstream regions of genes for the promoter analysis.

### Gene ontology and KOG analysis

Gene ontology (GO) enrichment analysis was carried out using BiNGO plug-in (v 2.44, https://www.psb.ugent.be/cbd/papers/BiNGO/Home.html) available in Cytoscape (version 3.2.1, http://www.cytoscape.org/), with *P-*value cut-off of ≤0.05. Rice GO information for biological process and molecular function categories available in BiNGO were used for GO enrichment analysis. Genes were classified according to eukaryotic orthologous group (KOG) grouping by searching gene sequences against KOGnitor database available at the National Center for Biotechnology Information (NCBI: https://www.ncbi.nlm.nih.gov/).

### Mapping of SNPs/InDels on QTLs

Two major effect QTLs (*qSH4* and *qSD7-2*^*BR*^) have been reported for seed shattering and seed dormancy, respectively^26,27^. The large effect SNPs/InDels and nonsynonymous SNPs present in these two QTLs were identified based on their co-localization in genomic coordinates of QTLs.

### Validation of SNPs and Indels

For validation, primers were designed from 400 bp flanking sequences of 27 randomly selected SNPs/Indels. The fragments were amplified from the genomic DNA of Bengal, Nona Bokra, and PSRR-1 as templates via polymerase chain reaction (PCR) using Phusion® High-Fidelity DNA Polymerase (New England Biolabs, Ipswich, USA). The PCR products were purified by using either DNA Clean and Concentrator^TM^-5 (ZYMO Research, Irvine, USA) or Gel Extraction Kit (OMEGA Bio-tek, Norcross, USA). The purified PCR products were sequenced at the Genomic Facility of Louisiana State University.

## Electronic supplementary material

Combined Supplemental information except Table S6

Supplementary Dataset Table S6
