# Supplementary material for: Genome-wide discovery of DNA polymorphisms by whole genome sequencing differentiates weedy and cultivated rice
Source: Sci Rep. 2018 Sep 21;8:14218. doi: 10.1038/s41598-018-32513-z (PMC6155081; doi:10.1038/s41598-018-32513-z)
Supplement: Supplementary file 1 — Combined Supplemental information except Table S6 [file 41598_2018_32513_MOESM1_ESM.pdf]

Supplementary Materials for

Genome-wide discovery of DNA polymorphisms by whole genome sequencing differentiates weedy and cultivated rice

Chenglin Chai, Rama Shankar, Mukesh Jain†, Prasanta K. Subudhi†

†Corresponding authors. Email: psubudhi@agcenter.lsu.edu (PKS), mjain@jnu.ac.in (MJ)

Tables (Six)

Table S1. Frequency of SNPs detected on individual rice chromosome.

Table S2. Frequency of InDels detected on individual rice chromosome.

Table S3. Distribution of SNPs in PSRR/Bengal, PSRR/Nona Bokra, and Bengal/Nona Bokra on 12 rice chromosomes.

Table S4. Distribution of InDels in PSRR/Bengal, PSRR/Nona Bokra, and Bengal/Nona Bokra on 12 rice chromosomes.

Table S5. Amplification and validation of randomly selected SNPs and InDels by PCR followed by Sanger sequencing.

Table S6. List of nonsynonymous SNPs and large-effect SNPs and InDels specific to PSRR-1.

Figures (Nine)

Figure S1. Length distributions of InDels in PSRR/Bengal, PSRR/Nona Bokra, and Bengal/Nona Bokra.

Figure S2. Amino-acid sequence alignment of LOC_Os01g02750 in PSRR and Bengal. The cold tolerant variety (PSRR) had a truncated protein, while the cold sensitive variety (Bengal) had a full length protein.

Figure S3. Amino-acid sequence alignment of LOC_Os05g36240 in PSRR and Bengal. The red arrow indicates the location of amino acid change from Tryptophan (W) in cold tolerant

genotype (PSRR) to Arginine (R) in cold sensitive variety (Bengal).

Figure S4. Amino-acid sequence alignment of LOC_Os11g45980 in PSRR and Bengal. The red arrow indicates the location of amino acid change from Threonine (T) in potentially blast disease resistant genotype (PSRR) to Isoleucine (I) in blast disease susceptible variety (Bengal).

Figure S5. GO enrichment analysis of genes harboring SNPs and InDels in promoter regions specific to PSRR showing involvement in possible biological processes (A) and molecular functions (B). The shaded colors indicate different significance level: white represents no significant difference; orange color – significant at P <0.0000005; yellow color - significant at P <0.05.

Figure S6. Functional classification of genes identified with nonsynonymous single-nucleotide polymorphisms (SNPs) or large-effect SNPs/InDels specific to PSRR and Bengal in the seed dormancy QTL *Sh4* and *qSD7-1* regions, respectively. Distribution of the eukaryotic orthologous group (KOG) classes in the genes were identified with nonsynonymous SNPs or large-effect SNPs/InDels. Annotation of KOG classes: J, Translation, ribosomal structure and biogenesis; K, Transcription; D, Cell cycle control, cell division, chromosome partitioning; T, Signal transduction mechanisms; U, Intracellular trafficking, secretion, and vesicular transport; G, Carbohydrate transport and metabolism; E, Amino acid transport and metabolism; H, Coenzyme transport and metabolism; Q, Secondary metabolites biosynthesis, transport and catabolism; R, General function prediction only; S, Function unknown.

Figure S7. GO enrichment analysis of genes harboring nonsynonymous/large-effect SNPs/InDels and SNPs/InDels in promoter regions in *Sh4* QTL specific to Bengal

showing involvement in possible biological processes (A) and molecular functions (B). The shaded colors indicate different significance level: white represents no significant difference; orange color – significant at P <0.0000005; yellow color – significant at P <0.05.

Figure S8. GO enrichment analysis of genes with nonsynonymous/large-effect SNPs/InDels and SNPs/InDels in promoter regions in *qSD7-1* specific to PSRR showing involvement in possible biological processes (A) and molecular functions (B). The shaded colors indicate different significance level: white represents no significant difference; orange color – significant at P <0.0000005; yellow color – significant at P <0.05.

Figure S9. Amino-acid sequence alignment of LOC_Os07g10490 in PSRR/Nona Bokra and Bengal. The red arrow indicates the location of amino acid change from Arginine (R) in genotypes with seed dormancy (PSRR and Nona Bokra) to Glutamine (Q) in non-dormant variety (Bengal).

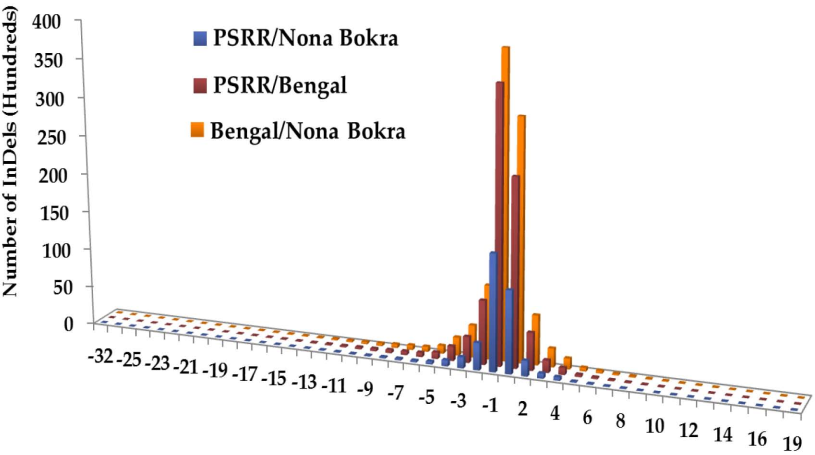

Figure S1. Length distributions of InDels in PSRR/Bengal, PSRR/Nona Bokra, and Bengal/Nona Bokra.

```
Majority                 MNTSMI HWHGCTXXXXXXXXXXXXXXXXXXXXXXXXXXXXXXXXXXXXXXXXXXXXXXXXXXXXXXXXXXXXXXXXXXXX
                                  10        20        30        40        50        60        70        80
LOC_01g02750_Bengal.pro  MNTSMI HWHGCTCKKLLKKQNLPAI TDAI FQLSVAAVQGSHVKVI AATSSVTAFVVLLSMVAI ALYLSLKTRYNEEI HMK  80
LOC_01g02750_PSRR.pro    MNTSMI HWHGCT                                                                      12

Majority                 XXXXXXXXXXXXXXXXXXXXXXXXXXXXXXXXXXXXXXXXXXXXXXXXXXXXXXXXXXXXXXXXXXXXXXXXXXXXXXXX
                                  90       100       110       120       130       140       150       160
LOC_01g02750_Bengal.pro  VEMFLKTYGTSKPTRYTFSEVKKI ARRFKVKVGQGGFGSVYRGELPNGVPVAVKMLENPKGEGDEFI NEVATI GRI HHAN  160
LOC_01g02750_PSRR.pro                                                                               12

Majority                 XXXXXXXXXXXXXXXXXXXXXXXXXXXXXXXXXXXXXXXXXXXXXXXXXXXXXXXXXXXXXXXXXXXXXXXXXXXXXXXX
                                 170       180       190       200       210       220       230       240
LOC_01g02750_Bengal.pro  I VRLLGFCSEGTRRALI YEYI PNDSLEKYI FSHDSNTSQELLVPSKMLDI ALGI ARGMEYLHQGCNQRI LHFDI KPNNI L  240
LOC_01g02750_PSRR.pro                                                                               12

Majority                 XXXXXXXXXXXXXXXXXXXXXXXXXXXXXXXXXXXXXXXXXXXXXXXXXXXXXXXXXXXXXXXXXXXXXXXXXXXXXXXX
                                 250       260       270       280       290       300       310       320
LOC_01g02750_Bengal.pro  LDYNFSPKI SDFGLAKLCARDQSI VTLTAARGTMGYI APELYSRNFGEI SYKSDVYSFGMLVLEMVSGRRNSDPSVESQN  320
LOC_01g02750_PSRR.pro                                                                               12

Majority                 XXXXXXXXXXXXXXXXXXXXXXXXXXXXXXXXXXXXXXXXXXXXXXXXXXXXXXXXXXXXXXXXXXXXXXXXXXXXXXXX
                                 330       340       350       360       370       380       390       400
LOC_01g02750_Bengal.pro  EVYFPECI YEQVTTGRDLELGREMTQEEKETMRQLAI VALWCI QWNPKNRPSMTKVVNMLTGRLQNLHVPPKPFFSADSH  400
LOC_01g02750_PSRR.pro                                                                               12

Majority                 XXX

LOC_01g02750_Bengal.pro  PI M                                                                        403
LOC_01g02750_PSRR.pro                                                                               12
```

Figure S2. Amino-acid sequence alignment of LOC_Os01g02750 in PSRR and Bengal. The cold tolerant variety (PSRR) had a truncated protein, while the cold sensitive variety (Bengal) had a full length protein.

```
Majority                      MNSIHRKRLARQQQGRNISTRSNASATNAKNRVPEMINGGGSSNSGANSSQRLCAGNSSNTDNASQATNISGSGMSSVPP
                                       10        20        30        40        50        60        70        80
LOC_Os05g36240_Bengal.pro     MNSIHRKRLARQQQGRNISTRSNASATNAKNRVPEMINGGGSSNSGANSSQRLCAGNSSNTDNASQATNISGSGMSSVPP  80
LOC_Os05g36240_PSRR.pro       MNSIHRKRLARQQQGRNISTRSNASATNAKNRVPEMINGGGSSNSGANSSQRLCAGNSSNTDNASQATNISGSGMSSVPP  80

Majority                      NKRGRKKGSRTALKVPPRGRKIALKPRGDEQFEYVTYPLLKSDPKYGTHMGIILKREYPGIIEEKDENGLIISSRLATSW
                                       90       100       110       120       130       140       150       160
LOC_Os05g36240_Bengal.pro     NKRGRKKGSRTALKVPPRGRKIALKPRGDEQFEYVTYPLLKSDPKYGTHMGIILKREYPGIIEEKDENGLIISSRLATSW  160
LOC_Os05g36240_PSRR.pro       NKRGRKKGSRTALKVPPRGRKIALKPRGDEQFEYVTYPLLKSDPKYGTHMGIILKREYPGIIEEKDENGLIISSRLATSW  160

Majority                      FDYYEKKDDSGVTNADKVKQEFWNAFCVSEADKVEADRVLENYAAKSVRQMMYQLRVDSVKVYHDKIGEQIDDALARPKE
                                      170       180       190       200       210       220       230       240
LOC_Os05g36240_Bengal.pro     FDYYEKKDDSGVTNADKVKQEFWNAFCVSEADKVEADRVLENYAAKSVRQMMYQLRVDSVKVYHDKIGEQIDDALARPKE  240
LOC_Os05g36240_PSRR.pro       FDYYEKKDDSGVTNADKVKQEFWNAFCVSEADKVEADRVLENYAAKSVRQMMYQLRVDSVKVYHDKIGEQIDDALARPKE  240

Majority                      LEVDEYKDGPVDWCRDNGFSYLADYWCTEEFKAKRKRGQQARLNSEDVAQNRGGSXPFTETQQYMGVKFGPEKATTLNTF
                                      250       260       270       280       290       300       310       320
LOC_Os05g36240_Bengal.pro     LEVDEYKDGPVDWCRDNGFSYLADYWCTEEFKAKRKRGQQARLNSEDVAQNRGGSRPFTETQQYMGVKFGPEKATTLNTF  320
LOC_Os05g36240_PSRR.pro       LEVDEYKDGPVDWCRDNGFSYLADYWCTEEFKAKRKRGQQARLNSEDVAQNRGGSWPFTETQQYMGVKFGPEKATTLNTF  320

Majority                      SAMKTGFKNIDNDGNIGPIPSQKAQKRVALYEKLGEQVPQELDCPVEEEDPFAQKIGLEGNKFFPCSLTQPVEDNGNNNS
                                      330       340       350       360       370       380       390       400
LOC_Os05g36240_Bengal.pro     SAMKTGFKNIDNDGNIGPIPSQKAQKRVALYEKLGEQVPQELDCPVEEEDPFAQKIGLEGNKFFPCSLTQPVEDNGNNNS  400
LOC_Os05g36240_PSRR.pro       SAMKTGFKNIDNDGNIGPIPSQKAQKRVALYEKLGEQVPQELDCPVEEEDPFAQKIGLEGNKFFPCSLTQPVEDNGNNNS  400

Majority                      NGCNTASGNGGNNNGDNNCNGNNTSSLNANDGSNIGGNNNSGGCNTSTS
                                      410       420       430       440
LOC_Os05g36240_Bengal.pro     NGCNTASGNGGNNNGDNNCNGNNTSSLNANDGSNIGGNNNSGGCNTSTS                                 449
LOC_Os05g36240_PSRR.pro       NGCNTASGNGGNNNGDNNCNGNNTSSLNANDGSNIGGNNNSGGCNTSTS                                 449
```

Figure S3. Amino-acid sequence alignment of LOC_Os05g36240 in PSRR and Bengal. The red arrow indicates the location of amino acid change from Tryptophan (W) in cold tolerant genotype (PSRR) to Arginine (R) in cold sensitive variety (Bengal).

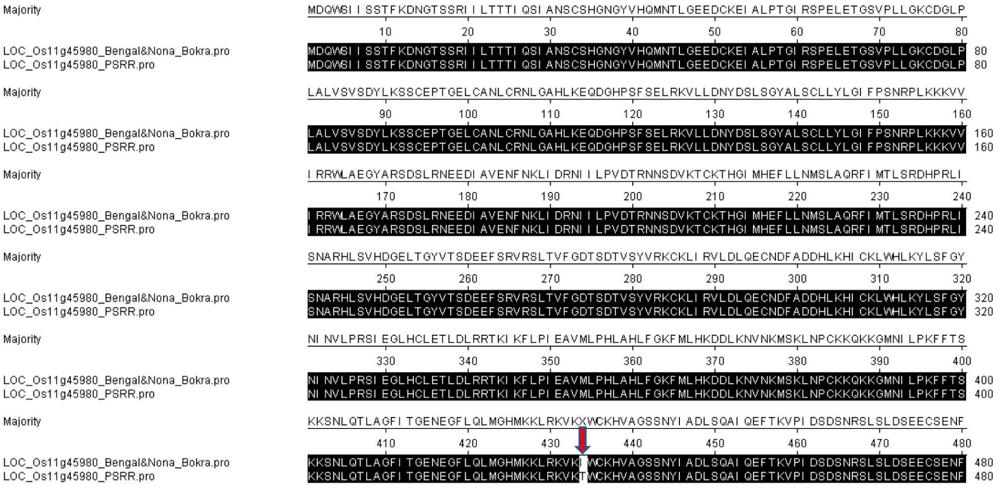

Figure S4. Amino-acid sequence alignment of LOC_Os11g45980 in PSRR and Bengal. The red arrow indicates the location of amino acid change from Threonine (T) in potentially blast disease resistant genotype (PSRR) to Isoleucine (I) in blast disease susceptible variety (Bengal).

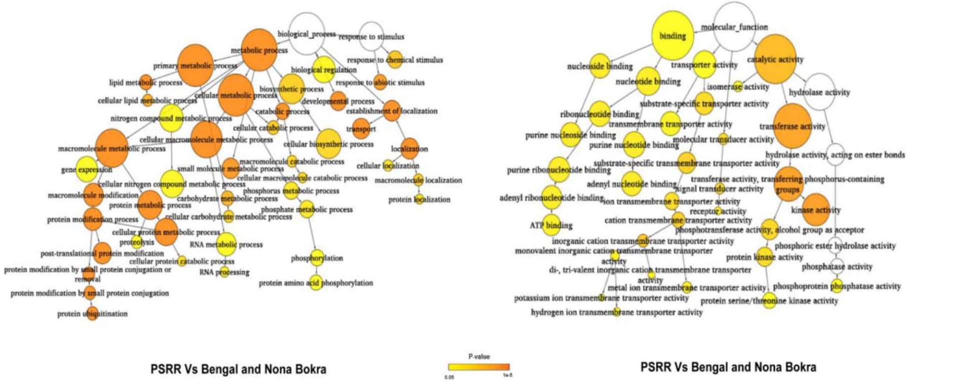

Figure S5. GO enrichment analysis of genes harboring SNPs and InDels in promoter regions specific to PSRR showing involvement in possible biological processes (A) and molecular functions (B). The shaded colors indicate different significance level: white represents no significant difference; orange color – significant at P <0.0000005; yellow color - significant at P <0.05.

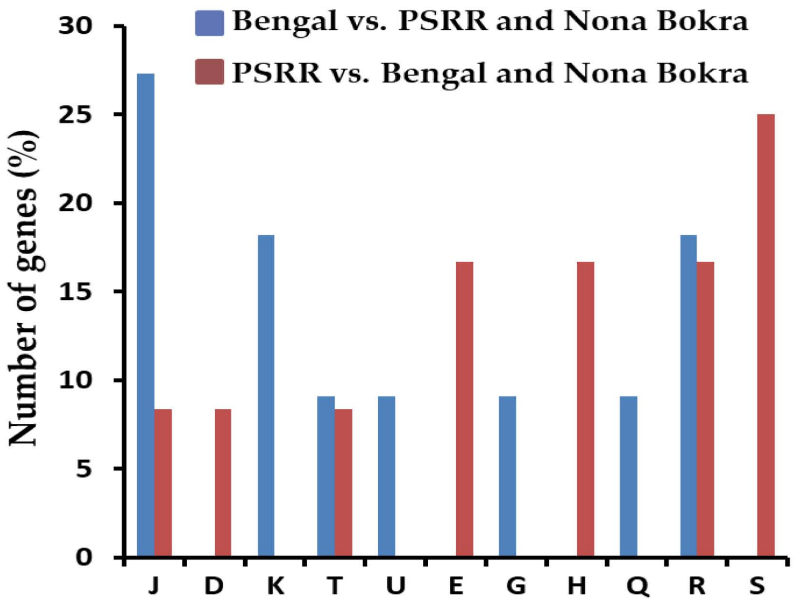

Figure S6. Functional classification of genes identified with nonsynonymous single-nucleotide polymorphisms (SNPs) or large-effect SNPs/InDels specific to PSRR and Bengal in the seed dormancy QTL *Sh4* and *qSD7-1* regions, respectively. Distribution of the eukaryotic orthologous group (KOG) classes in the genes were identified with nonsynonymous SNPs or large-effect SNPs/InDels. Annotation of KOG classes: J, Translation, ribosomal structure and biogenesis; K, Transcription; D, Cell cycle control, cell division, chromosome partitioning; T, Signal transduction mechanisms; U, Intracellular trafficking, secretion, and vesicular transport; G, Carbohydrate transport and metabolism; E, Amino acid transport and metabolism; H, Coenzyme transport and metabolism; Q, Secondary metabolites biosynthesis, transport and catabolism; R, General function prediction only; S, Function unknown.

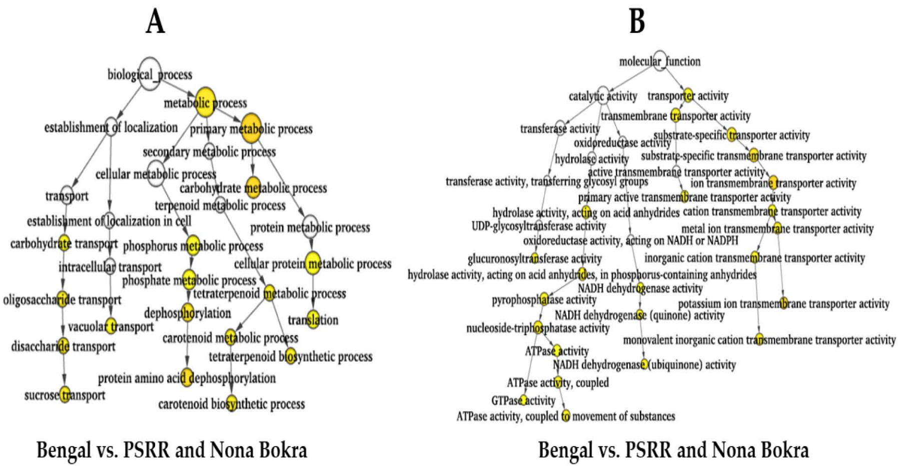

Figure S7. GO enrichment analysis of genes harboring nonsynonymous/large-effect SNPs/InDels and SNPs/InDels in promoter regions in *Sh4* QTL specific to Bengal showing involvement in possible biological processes (A) and molecular functions (B). The shaded colors indicate different significance level: white represents no significant difference; orange color – significant at P <0.0000005; yellow color – significant at P <0.05.

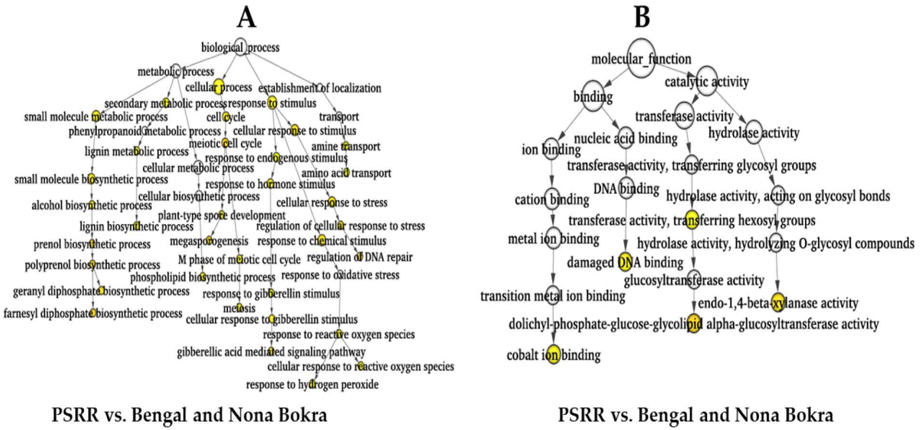

Figure S8. GO enrichment analysis of genes with nonsynonymous/large-effect SNPs/InDels and SNPs/InDels in promoter regions in *qSD7-1* specific to PSRR showing involvement in possible biological processes (A) and molecular functions (B). The shaded colors indicate different significance level: white represents no significant difference; orange color – significant at P <0.0000005; yellow color – significant at P <0.05.

```
                     1 [         .         .         .         .        : 50
Bengal                 MAMAATSRAPSTLAPASFSAAGGSRRRRRCPNPRVRVGVGVRCSLDSNVS
PSRR/Nona Bokra        MAMAATSRAPSTLAPASFSAAGGSRRRRRCPNPRVRVGVGVRCSLDSNVS

                    51           .         .         .         .          100
Bengal                 DMAVNAPKGLFPPEPEHYRGPKLKVAIIGAGLAGMSTAVELLDQGHEVDL
PSRR/Nona Bokra        DMAVNAPKGLFPPEPEHYRGPKLKVAIIGAGLAGMSTAVELLDQGHEVDL

                   101           .         .         .         .        : 150
Bengal                 YESRPFIGGKVGSFVDRKGNHIEMGLHVFFGCYSNLFRLMKKVGADNNLL
PSRR/Nona Bokra        YESRPFIGGKVGSFVDRKGNHIEMGLHVFFGCYSNLFRLMKKVGADNNLL

                   151           .         .         .         .          200
Bengal                 VKEHTHTFVNKGGTIGELDFRFPVGAPLHGIQAFLRTNQLKVYDKARNAV
PSRR/Nona Bokra        VKEHTHTFVNKGGTIGELDFRFPVGAPLHGIQAFLRTNQLKVYDKARNAV

                   201           .         .         .         .        : 250
Bengal                 ALALSPVVRALVDPDGALQQVRDLDDVSFSDWFLSKGGTRESITRMWDPV
PSRR/Nona Bokra        ALALSPVVRALVDPDGALQQVRDLDDVSFSDWFLSKGGTRESITRMWDPV

                   251           .         .         .         .          300
Bengal                 AYALGFIDCDNISARCMLTIFTLFATKTEASLLRMLKGSPDVYLSGPIKK
PSRR/Nona Bokra        AYALGFIDCDNISARCMLTIFTLFATKTEASLLRMLKGSPDVYLSGPIKK

                   301           .         .         .         .        : 350
Bengal                 YITDRGGRFHLRWGCREVLYDKSPDGETYVKGLLLSKATSREIIKADAYV
PSRR/Nona Bokra        YITDRGGRFHLRWGCREVLYDKSPDGETYVKGLLLSKATSREIIKADAYV
                   351           .         .         .         .          400
Bengal                 AACDVPGIKRLLPSEWRQWDTFDNIYKLDGVPVVTVQLRYNGWVTELQDL
PSRR/Nona Bokra        AACDVPGIKRLLPSEWRQWDTFDNIYKLDGVPVVTVQLRYNGWVTELQDL
                   401           .         .         .         .        : 450
Bengal                 EKSRQLKKAVGLDNLLYTPDADFSCFSDLALSSPADYYIEGQGSLIQAVL
PSRR/Nona Bokra        EKSRQLKKAVGLDNLLYTPDADFSCFSDLALSSPADYYIEGQGSLIQAVL

                   451           .         .         .         .          500
Bengal                 TPGDPYMPLPNEEIISKVQKQVLELFPSSQGLELTWSSVVKIGQSLYRES
PSRR/Nona Bokra        TPGDPYMPLPNEEIISKVQKQVLELFPSSRGLELTWSSVVKIGQSLYRES

                   501           .         .         .         .        : 550
Bengal                 PGNDPFRPDQKTPVKNFFLSGSYTKQDYIDSMEGATLSGRRTAAYICGAG
PSRR/Nona Bokra        PGNDPFRPDQKTPVKNFFLSGSYTKQDYIDSMEGATLSGRRTAAYICGAG

                   551           .         .       ] 578
Bengal                 EELLALRKKLIVDDSEKARGKVEALQTS
PSRR/Nona Bokra        EELLALRKKLIVDDSEKARGKVEALQTS
```

Figure S9. Amino-acid sequence alignment of LOC_Os07g10490 in PSRR/Nona Bokra and Bengal. The red arrow indicates the location of amino acid change from Arginine (R) in genotypes with seed dormancy (PSRR and Nona Bokra) to Glutamine (Q) in non-dormant variety (Bengal).

Table S1 Frequency of SNPs detected on individual rice chromosome.

Chromosomes	Length (bp)	PSRR/Bengal		PSRR/Nona Bokra		Bengal/ Nona Bokra	
		No. of SNPs	SNPs/100kb	No. of SNPs	SNPs/100kb	No. of SNPs	SNPs/100kb
Chr1	43270923	182344	421.4	71758	165.8	161146	372.4
Chr2	35937250	174874	486.6	64717	180.1	139388	387.9
Chr3	36413819	177126	486.4	47133	129.4	147477	405.0
Chr4	35502694	130249	366.9	61725	173.9	117284	330.4
Chr5	29958434	152311	508.4	64362	214.8	116401	388.5
Chr6	31248787	154459	494.3	57919	185.3	122133	390.8
Chr7	29697621	146932	494.8	42112	141.8	121168	408.0
Chr8	28443022	132699	466.5	55900	196.5	105252	370.0
Chr9	23012720	112462	488.7	43573	189.3	94966	412.7
Chr10	23207287	105933	456.5	35964	155.0	92137	397.0
Chr11	29021106	123942	427.1	62187	214.3	102725	354.0
Chr12	27531856	112771	409.6	26859	97.6	96332	349.9
ChrSyn	592136	1130	190.8	183	30.9	895	151.1
ChrUn	633585	530	83.7	658	103.9	563	88.9
Total	374471240	1707762		635050		1417867	

ChrSy and ChrUn represent the Syngenta and unanchored BAC pseudomolecules, respectively, as included in the MSU7 rice genome.

Table S2 Frequency of InDels detected on individual rice chromosome.

		PSRR/Bengal		PSRR/Nona Bokra		Bengal/ Nona Bokra	
Chr.	Length (bp)	No. of InDels	InDels/100kb	No. of InDels	InDels/100kb	No. of InDels	InDels/100kb
Chr1	43270923	10506	24.3	5088	11.8	12794	29.6
Chr2	35937250	9435	26.3	4182	11.6	10904	30.3
Chr3	36413819	9556	26.2	3211	8.8	11732	32.2
Chr4	35502694	6712	18.9	3519	9.9	8100	22.8
Chr5	29958434	7363	24.6	3231	10.8	8441	28.2
Chr6	31248787	7814	25.0	3386	10.8	8873	28.4
Chr7	29697621	7555	25.4	2416	8.1	9023	30.4
Chr8	28443022	6499	22.8	3187	11.2	7808	27.5
Chr9	23012720	5328	23.2	2650	11.5	6819	29.6
Chr10	23207287	5560	24.0	2124	9.2	6508	28.0
Chr11	29021106	6518	22.5	3871	13.3	7721	26.6
Chr12	27531856	5589	20.3	1826	6.6	6868	24.9
ChrSyn	592136	58	9.8	8	1.4	60	10.1
ChrUn	633585	25	3.9	34	5.4	22	3.5
Total	374471240	88518		38733		105673	

ChrSy and ChrUn represent the Syngenta and unanchored BAC pseudomolecules, respectively, as included in the MSU7 rice genome.

**Table S3.** Distribution of SNPs in PSRR/Bengal, PSRR/Nona Bokra, and Bengal/Nona Bokra on 12 rice chromosomes							
					SNP		
				All	Bengal/Nona Bokra	PSSR/Bengal	PSSR/Nona Bokra
Chr1							
Chr1	1	100000		465	436	15	432
Chr1	100001	200000		372	354	14	348
Chr1	200001	300000		435	414	19	415
Chr1	300001	400000		349	339	8	343
Chr1	400001	500000		762	743	16	741
Chr1	500001	600000		406	379	7	384
Chr1	600001	700000		550	480	385	159
Chr1	700001	800000		917	537	496	93
Chr1	800001	900000		648	401	413	1
Chr1	900001	1000000		701	228	236	13
Chr1	1000001	1100000		732	318	395	160
Chr1	1100001	1200000		50	39	8	39
Chr1	1200001	1300000		202	183	14	186
Chr1	1300001	1400000		546	508	26	516
Chr1	1400001	1500000		380	308	67	308
Chr1	1500001	1600000		706	245	340	308
Chr1	1600001	1700000		603	333	33	321
Chr1	1700001	1800000		854	274	18	278
Chr1	1800001	1900000		747	338	18	355
Chr1	1900001	2000000		909	17	18	13
Chr1	2000001	2100000		472	11	11	5
Chr1	2100001	2200000		1212	641	618	198
Chr1	2200001	2300000		973	580	124	433
Chr1	2300001	2400000		660	408	319	377
Chr1	2400001	2500000		859	117	289	64
Chr1	2500001	2600000		844	537	515	295
Chr1	2600001	2700000		811	489	457	455
Chr1	2700001	2800000		822	464	419	475
Chr1	2800001	2900000		1203	902	800	456
Chr1	2900001	3000000		602	424	269	353
Chr1	3000001	3100000		819	493	183	479
Chr1	3100001	3200000		679	436	204	267
Chr1	3200001	3300000		949	475	312	443
Chr1	3300001	3400000		988	717	167	672
Chr1	3400001	3500000		785	416	464	399
Chr1	3500001	3600000		685	388	435	414
Chr1	3600001	3700000		623	396	500	206
Chr1	3700001	3800000		643	351	331	299
Chr1	3800001	3900000		1230	1019	383	804
Chr1	3900001	4000000		708	342	509	368
Chr1	4000001	4100000		843	534	585	315
Chr1	4100001	4200000		692	407	481	227
Chr1	4200001	4300000		727	380	603	248

					SNP		
				All	Bengal/Nona Bokra	PSSR/Bengal	PSSR/Nona Bokra
Chr1	4300001	4400000		626	347	414	146
Chr1	4400001	4500000		741	448	442	151
Chr1	4500001	4600000		747	405	538	110
Chr1	4600001	4700000		717	354	513	182
Chr1	4700001	4800000		527	297	391	179
Chr1	4800001	4900000		665	438	469	86
Chr1	4900001	5000000		834	450	564	16
Chr1	5000001	5100000		759	542	658	7
Chr1	5100001	5200000		916	600	719	5
Chr1	5200001	5300000		579	422	476	78
Chr1	5300001	5400000		804	517	605	255
Chr1	5400001	5500000		722	369	448	181
Chr1	5500001	5600000		820	517	645	195
Chr1	5600001	5700000		892	548	494	542
Chr1	5700001	5800000		489	322	230	348
Chr1	5800001	5900000		58	49	13	42
Chr1	5900001	6000000		812	393	519	470
Chr1	6000001	6100000		893	260	725	544
Chr1	6100001	6200000		869	325	606	355
Chr1	6200001	6300000		936	505	583	9
Chr1	6300001	6400000		1076	522	655	179
Chr1	6400001	6500000		441	266	266	284
Chr1	6500001	6600000		421	144	400	203
Chr1	6600001	6700000		793	292	602	294
Chr1	6700001	6800000		694	348	379	2
Chr1	6800001	6900000		1012	584	686	6
Chr1	6900001	7000000		832	327	376	8
Chr1	7000001	7100000		1091	352	566	438
Chr1	7100001	7200000		1013	679	478	658
Chr1	7200001	7300000		918	537	617	463
Chr1	7300001	7400000		1024	567	798	399
Chr1	7400001	7500000		605	498	575	2
Chr1	7500001	7600000		572	425	537	5
Chr1	7600001	7700000		929	622	768	6
Chr1	7700001	7800000		757	499	592	8
Chr1	7800001	7900000		613	406	481	4
Chr1	7900001	8000000		47	27	27	5
Chr1	8000001	8100000		24	8	6	5
Chr1	8100001	8200000		25	6	9	6
Chr1	8200001	8300000		12	7	7	3
Chr1	8300001	8400000		27	16	18	6
Chr1	8400001	8500000		25	13	14	8
Chr1	8500001	8600000		30	11	16	8
Chr1	8600001	8700000		24	14	15	8
Chr1	8700001	8800000		203	188	10	186

					SNP		
				All	Bengal/Nona Bokra	PSSR/Bengal	PSSR/Nona Bokra
Chr1	8800001	8900000		383	353	19	359
Chr1	8900001	9000000		597	541	111	466
Chr1	9000001	9100000		630	473	515	93
Chr1	9100001	9200000		525	444	200	307
Chr1	9200001	9300000		540	470	320	200
Chr1	9300001	9400000		711	521	494	142
Chr1	9400001	9500000		708	500	635	5
Chr1	9500001	9600000		682	524	633	6
Chr1	9600001	9700000		513	418	497	4
Chr1	9700001	9800000		702	519	661	5
Chr1	9800001	9900000		703	535	646	4
Chr1	9900001	10000000		688	474	641	13
Chr1	1E+07	10100000		557	357	463	59
Chr1	1E+07	10200000		665	425	602	5
Chr1	1E+07	10300000		581	435	544	7
Chr1	1E+07	10400000		732	470	609	18
Chr1	1E+07	10500000		662	463	602	6
Chr1	1.1E+07	10600000		858	586	780	10
Chr1	1.1E+07	10700000		706	531	641	7
Chr1	1.1E+07	10800000		573	410	469	2
Chr1	1.1E+07	10900000		590	389	466	185
Chr1	1.1E+07	11000000		886	523	679	331
Chr1	1.1E+07	11100000		783	554	692	125
Chr1	1.1E+07	11200000		1096	498	653	520
Chr1	1.1E+07	11300000		818	481	672	132
Chr1	1.1E+07	11400000		1017	624	786	268
Chr1	1.1E+07	11500000		795	472	627	259
Chr1	1.2E+07	11600000		913	584	778	174
Chr1	1.2E+07	11700000		695	474	662	24
Chr1	1.2E+07	11800000		865	657	803	31
Chr1	1.2E+07	11900000		966	497	717	8
Chr1	1.2E+07	12000000		867	574	711	9
Chr1	1.2E+07	12100000		763	491	682	21
Chr1	1.2E+07	12200000		917	604	723	12
Chr1	1.2E+07	12300000		709	449	696	124
Chr1	1.2E+07	12400000		706	13	681	576
Chr1	1.2E+07	12500000		425	11	392	324
Chr1	1.3E+07	12600000		731	7	713	607
Chr1	1.3E+07	12700000		519	11	504	425
Chr1	1.3E+07	12800000		599	410	516	5
Chr1	1.3E+07	12900000		723	466	575	12
Chr1	1.3E+07	13000000		544	297	400	7
Chr1	1.3E+07	13100000		317	51	64	2
Chr1	1.3E+07	13200000		1205	670	762	7
Chr1	1.3E+07	13300000		812	471	619	106

					SNP		
				All	Bengal/Nona Bokra	PSSR/Bengal	PSSR/Nona Bokra
Chr1	1.3E+07	13400000		937	487	723	347
Chr1	1.3E+07	13500000		824	376	448	374
Chr1	1.4E+07	13600000		867	422	566	423
Chr1	1.4E+07	13700000		114	43	72	10
Chr1	1.4E+07	13800000		835	463	552	339
Chr1	1.4E+07	13900000		744	390	420	451
Chr1	1.4E+07	14000000		1067	713	790	170
Chr1	1.4E+07	14100000		1077	634	847	243
Chr1	1.4E+07	14200000		871	445	592	270
Chr1	1.4E+07	14300000		1140	587	534	271
Chr1	1.4E+07	14400000		1239	737	754	649
Chr1	1.4E+07	14500000		891	531	662	347
Chr1	1.5E+07	14600000		767	394	411	333
Chr1	1.5E+07	14700000		767	465	583	182
Chr1	1.5E+07	14800000		1024	603	767	390
Chr1	1.5E+07	14900000		832	643	213	467
Chr1	1.5E+07	15000000		181	131	56	8
Chr1	1.5E+07	15100000		122	44	72	8
Chr1	1.5E+07	15200000		633	430	435	147
Chr1	1.5E+07	15300000		587	408	301	105
Chr1	1.5E+07	15400000		772	455	481	301
Chr1	1.5E+07	15500000		1003	762	861	107
Chr1	1.6E+07	15600000		611	439	533	11
Chr1	1.6E+07	15700000		761	568	660	1
Chr1	1.6E+07	15800000		260	148	136	2
Chr1	1.6E+07	15900000		1069	780	990	14
Chr1	1.6E+07	16000000		848	679	643	8
Chr1	1.6E+07	16100000		659	466	587	3
Chr1	1.6E+07	16200000		873	618	811	13
Chr1	1.6E+07	16300000		858	603	769	10
Chr1	1.6E+07	16400000		768	545	720	13
Chr1	1.6E+07	16500000		981	733	911	10
Chr1	1.7E+07	16600000		724	527	655	5
Chr1	1.7E+07	16700000		618	433	559	3
Chr1	1.7E+07	16800000		496	276	384	9
Chr1	1.7E+07	16900000		201	45	87	3
Chr1	1.7E+07	17000000		261	98	181	7
Chr1	1.7E+07	17100000		793	502	692	6
Chr1	1.7E+07	17200000		970	577	764	6
Chr1	1.7E+07	17300000		791	549	696	9
Chr1	1.7E+07	17400000		642	502	587	8
Chr1	1.7E+07	17500000		710	521	667	8
Chr1	1.8E+07	17600000		770	541	709	8
Chr1	1.8E+07	17700000		742	515	687	5
Chr1	1.8E+07	17800000		788	532	691	5

					SNP		
				All	Bengal/Nona Bokra	PSSR/Bengal	PSSR/Nona Bokra
Chr1	1.8E+07	17900000		1324	833	1124	14
Chr1	1.8E+07	18000000		745	456	580	109
Chr1	1.8E+07	18100000		920	535	712	330
Chr1	1.8E+07	18200000		564	386	490	134
Chr1	1.8E+07	18300000		847	575	792	13
Chr1	1.8E+07	18400000		620	476	587	36
Chr1	1.8E+07	18500000		748	503	647	67
Chr1	1.9E+07	18600000		420	308	317	5
Chr1	1.9E+07	18700000		663	423	539	55
Chr1	1.9E+07	18800000		1155	613	770	386
Chr1	1.9E+07	18900000		923	475	652	210
Chr1	1.9E+07	19000000		822	419	505	351
Chr1	1.9E+07	19100000		1018	737	889	12
Chr1	1.9E+07	19200000		741	579	704	2
Chr1	1.9E+07	19300000		537	319	395	11
Chr1	1.9E+07	19400000		806	478	596	176
Chr1	1.9E+07	19500000		591	22	564	509
Chr1	2E+07	19600000		386	16	360	326
Chr1	2E+07	19700000		323	9	299	265
Chr1	2E+07	19800000		113	14	87	78
Chr1	2E+07	19900000		45	13	26	17
Chr1	2E+07	20000000		439	14	421	379
Chr1	2E+07	20100000		616	480	169	508
Chr1	2E+07	20200000		498	468	25	466
Chr1	2E+07	20300000		973	946	26	943
Chr1	2E+07	20400000		436	424	9	423
Chr1	2E+07	20500000		471	445	8	451
Chr1	2.1E+07	20600000		424	409	8	406
Chr1	2.1E+07	20700000		533	519	12	518
Chr1	2.1E+07	20800000		198	174	13	174
Chr1	2.1E+07	20900000		106	81	18	39
Chr1	2.1E+07	21000000		449	428	12	425
Chr1	2.1E+07	21100000		204	186	14	191
Chr1	2.1E+07	21200000		56	45	48	5
Chr1	2.1E+07	21300000		236	190	207	5
Chr1	2.1E+07	21400000		500	350	412	3
Chr1	2.1E+07	21500000		574	442	258	36
Chr1	2.2E+07	21600000		110	40	47	1
Chr1	2.2E+07	21700000		971	759	335	491
Chr1	2.2E+07	21800000		993	486	673	513
Chr1	2.2E+07	21900000		796	503	455	235
Chr1	2.2E+07	22000000		745	395	485	406
Chr1	2.2E+07	22100000		965	525	683	488
Chr1	2.2E+07	22200000		653	288	603	227
Chr1	2.2E+07	22300000		482	375	402	8

					SNP		
				All	Bengal/Nona Bokra	PSSR/Bengal	PSSR/Nona Bokra
Chr1	2.2E+07	22400000		662	443	389	205
Chr1	2.2E+07	22500000		1284	759	981	524
Chr1	2.3E+07	22600000		648	495	505	18
Chr1	2.3E+07	22700000		712	480	625	79
Chr1	2.3E+07	22800000		739	550	264	543
Chr1	2.3E+07	22900000		1038	450	631	548
Chr1	2.3E+07	23000000		984	379	427	211
Chr1	2.3E+07	23100000		604	169	234	127
Chr1	2.3E+07	23200000		807	345	392	308
Chr1	2.3E+07	23300000		934	517	700	283
Chr1	2.3E+07	23400000		1063	464	668	497
Chr1	2.3E+07	23500000		1419	726	778	360
Chr1	2.4E+07	23600000		1022	630	607	265
Chr1	2.4E+07	23700000		1247	659	784	547
Chr1	2.4E+07	23800000		984	532	675	497
Chr1	2.4E+07	23900000		878	483	580	344
Chr1	2.4E+07	24000000		612	465	551	63
Chr1	2.4E+07	24100000		928	638	791	7
Chr1	2.4E+07	24200000		622	417	497	140
Chr1	2.4E+07	24300000		668	555	639	8
Chr1	2.4E+07	24400000		546	411	524	7
Chr1	2.4E+07	24500000		683	517	645	3
Chr1	2.5E+07	24600000		646	456	608	4
Chr1	2.5E+07	24700000		974	743	920	7
Chr1	2.5E+07	24800000		914	636	722	257
Chr1	2.5E+07	24900000		822	384	454	274
Chr1	2.5E+07	25000000		970	230	369	201
Chr1	2.5E+07	25100000		889	272	394	226
Chr1	2.5E+07	25200000		743	236	303	28
Chr1	2.5E+07	25300000		1056	565	727	103
Chr1	2.5E+07	25400000		931	481	599	533
Chr1	2.5E+07	25500000		679	397	384	436
Chr1	2.6E+07	25600000		250	174	159	124
Chr1	2.6E+07	25700000		657	471	357	349
Chr1	2.6E+07	25800000		83	12	13	5
Chr1	2.6E+07	25900000		185	8	8	3
Chr1	2.6E+07	26000000		238	7	10	5
Chr1	2.6E+07	26100000		779	13	368	335
Chr1	2.6E+07	26200000		701	12	594	518
Chr1	2.6E+07	26300000		857	8	752	640
Chr1	2.6E+07	26400000		842	14	619	542
Chr1	2.6E+07	26500000		810	371	374	536
Chr1	2.7E+07	26600000		395	5	271	226
Chr1	2.7E+07	26700000		570	388	465	11
Chr1	2.7E+07	26800000		699	240	275	8

					SNP		
				All	Bengal/Nona Bokra	PSSR/Bengal	PSSR/Nona Bokra
Chr1	2.7E+07	26900000		654	457	590	8
Chr1	2.7E+07	27000000		457	266	342	8
Chr1	2.7E+07	27100000		722	478	478	88
Chr1	2.7E+07	27200000		486	216	10	213
Chr1	2.7E+07	27300000		530	421	505	5
Chr1	2.7E+07	27400000		372	270	354	2
Chr1	2.7E+07	27500000		328	213	255	15
Chr1	2.8E+07	27600000		687	553	635	8
Chr1	2.8E+07	27700000		706	504	605	88
Chr1	2.8E+07	27800000		700	523	660	8
Chr1	2.8E+07	27900000		814	437	581	14
Chr1	2.8E+07	28000000		572	55	59	3
Chr1	2.8E+07	28100000		509	5	4	5
Chr1	2.8E+07	28200000		706	14	92	83
Chr1	2.8E+07	28300000		694	6	221	221
Chr1	2.8E+07	28400000		919	297	383	303
Chr1	2.8E+07	28500000		785	479	599	11
Chr1	2.9E+07	28600000		1011	497	574	7
Chr1	2.9E+07	28700000		689	542	649	8
Chr1	2.9E+07	28800000		418	305	405	6
Chr1	2.9E+07	28900000		605	435	566	14
Chr1	2.9E+07	29000000		779	620	684	13
Chr1	2.9E+07	29100000		372	300	307	11
Chr1	2.9E+07	29200000		668	517	637	5
Chr1	2.9E+07	29300000		647	475	601	2
Chr1	2.9E+07	29400000		515	378	487	14
Chr1	2.9E+07	29500000		590	463	578	3
Chr1	3E+07	29600000		852	719	741	4
Chr1	3E+07	29700000		519	380	480	5
Chr1	3E+07	29800000		354	270	305	4
Chr1	3E+07	29900000		677	485	646	17
Chr1	3E+07	30000000		797	628	764	7
Chr1	3E+07	30100000		563	432	553	2
Chr1	3E+07	30200000		497	372	468	5
Chr1	3E+07	30300000		647	460	589	5
Chr1	3E+07	30400000		543	407	519	5
Chr1	3E+07	30500000		532	418	514	3
Chr1	3.1E+07	30600000		574	477	557	5
Chr1	3.1E+07	30700000		700	488	633	7
Chr1	3.1E+07	30800000		1189	986	1024	4
Chr1	3.1E+07	30900000		843	536	680	168
Chr1	3.1E+07	31000000		772	559	685	86
Chr1	3.1E+07	31100000		701	547	645	8
Chr1	3.1E+07	31200000		406	293	372	3
Chr1	3.1E+07	31300000		757	534	676	42

					SNP		
				All	Bengal/Nona Bokra	PSSR/Bengal	PSSR/Nona Bokra
Chr1	3.1E+07	31400000		752	482	615	253
Chr1	3.1E+07	31500000		559	337	446	173
Chr1	3.2E+07	31600000		741	469	590	203
Chr1	3.2E+07	31700000		830	482	576	242
Chr1	3.2E+07	31800000		723	454	540	6
Chr1	3.2E+07	31900000		735	314	377	4
Chr1	3.2E+07	32000000		636	20	19	5
Chr1	3.2E+07	32100000		535	15	14	5
Chr1	3.2E+07	32200000		649	72	12	64
Chr1	3.2E+07	32300000		801	326	255	144
Chr1	3.2E+07	32400000		765	182	254	96
Chr1	3.2E+07	32500000		689	250	299	7
Chr1	3.3E+07	32600000		655	103	125	8
Chr1	3.3E+07	32700000		490	228	267	5
Chr1	3.3E+07	32800000		411	346	397	3
Chr1	3.3E+07	32900000		614	462	561	3
Chr1	3.3E+07	33000000		476	364	457	4
Chr1	3.3E+07	33100000		546	325	377	2
Chr1	3.3E+07	33200000		465	263	315	1
Chr1	3.3E+07	33300000		758	629	571	1
Chr1	3.3E+07	33400000		561	400	528	1
Chr1	3.3E+07	33500000		932	718	867	13
Chr1	3.4E+07	33600000		204	145	143	11
Chr1	3.4E+07	33700000		432	331	385	7
Chr1	3.4E+07	33800000		836	665	789	9
Chr1	3.4E+07	33900000		433	314	407	5
Chr1	3.4E+07	34000000		505	395	476	2
Chr1	3.4E+07	34100000		486	374	466	5
Chr1	3.4E+07	34200000		510	360	481	6
Chr1	3.4E+07	34300000		767	578	730	5
Chr1	3.4E+07	34400000		690	532	660	15
Chr1	3.4E+07	34500000		752	592	682	12
Chr1	3.5E+07	34600000		716	593	681	30
Chr1	3.5E+07	34700000		668	379	528	247
Chr1	3.5E+07	34800000		672	421	509	120
Chr1	3.5E+07	34900000		655	439	510	155
Chr1	3.5E+07	35000000		511	391	478	9
Chr1	3.5E+07	35100000		597	373	484	153
Chr1	3.5E+07	35200000		931	564	699	213
Chr1	3.5E+07	35300000		777	405	492	230
Chr1	3.5E+07	35400000		673	410	528	274
Chr1	3.5E+07	35500000		531	311	421	193
Chr1	3.6E+07	35600000		601	366	487	168
Chr1	3.6E+07	35700000		588	415	492	137
Chr1	3.6E+07	35800000		570	403	481	92

					SNP		
				All	Bengal/Nona Bokra	PSSR/Bengal	PSSR/Nona Bokra
Chr1	3.6E+07	35900000		516	324	428	165
Chr1	3.6E+07	36000000		703	394	547	271
Chr1	3.6E+07	36100000		718	453	576	256
Chr1	3.6E+07	36200000		633	411	485	246
Chr1	3.6E+07	36300000		767	476	671	158
Chr1	3.6E+07	36400000		569	349	498	128
Chr1	3.6E+07	36500000		733	482	603	213
Chr1	3.7E+07	36600000		394	230	316	76
Chr1	3.7E+07	36700000		623	415	491	180
Chr1	3.7E+07	36800000		623	446	544	79
Chr1	3.7E+07	36900000		396	268	346	39
Chr1	3.7E+07	37000000		621	389	512	160
Chr1	3.7E+07	37100000		587	423	531	61
Chr1	3.7E+07	37200000		669	463	527	206
Chr1	3.7E+07	37300000		569	339	462	111
Chr1	3.7E+07	37400000		592	374	454	109
Chr1	3.7E+07	37500000		701	179	14	175
Chr1	3.8E+07	37600000		607	239	5	237
Chr1	3.8E+07	37700000		634	201	124	233
Chr1	3.8E+07	37800000		898	128	386	354
Chr1	3.8E+07	37900000		724	239	10	247
Chr1	3.8E+07	38000000		591	87	6	85
Chr1	3.8E+07	38100000		629	38	5	35
Chr1	3.8E+07	38200000		777	196	14	181
Chr1	3.8E+07	38300000		746	157	4	163
Chr1	3.8E+07	38400000		720	206	55	200
Chr1	3.8E+07	38500000		585	114	1	115
Chr1	3.9E+07	38600000		633	364	3	362
Chr1	3.9E+07	38700000		570	208	0	208
Chr1	3.9E+07	38800000		959	398	8	362
Chr1	3.9E+07	38900000		638	55	0	54
Chr1	3.9E+07	39000000		571	11	4	6
Chr1	3.9E+07	39100000		557	4	1	5
Chr1	3.9E+07	39200000		605	11	8	13
Chr1	3.9E+07	39300000		619	51	3	49
Chr1	3.9E+07	39400000		660	3	0	3
Chr1	3.9E+07	39500000		458	3	3	3
Chr1	4E+07	39600000		696	227	2	234
Chr1	4E+07	39700000		549	70	0	67
Chr1	4E+07	39800000		546	136	14	130
Chr1	4E+07	39900000		599	57	1	61
Chr1	4E+07	40000000		499	11	7	7
Chr1	4E+07	40100000		707	17	8	13
Chr1	4E+07	40200000		566	10	8	10
Chr1	4E+07	40300000		606	2	8	6

					SNP		
				All	Bengal/Nona Bokra	PSSR/Bengal	PSSR/Nona Bokra
Chr1	4E+07	40400000		770	266	2	334
Chr1	4E+07	40500000		802	269	73	279
Chr1	4.1E+07	40600000		897	191	9	179
Chr1	4.1E+07	40700000		776	271	8	269
Chr1	4.1E+07	40800000		612	220	4	217
Chr1	4.1E+07	40900000		733	83	52	119
Chr1	4.1E+07	41000000		767	105	221	261
Chr1	4.1E+07	41100000		683	287	227	123
Chr1	4.1E+07	41200000		817	307	338	12
Chr1	4.1E+07	41300000		807	96	437	263
Chr1	4.1E+07	41400000		775	147	366	268
Chr1	4.1E+07	41500000		93	0	28	39
Chr1	4.2E+07	41600000		691	13	321	284
Chr1	4.2E+07	41700000		257	26	74	34
Chr1	4.2E+07	41800000		827	416	430	133
Chr1	4.2E+07	41900000		708	126	434	311
Chr1	4.2E+07	42000000		631	121	530	471
Chr1	4.2E+07	42100000		834	276	659	548
Chr1	4.2E+07	42200000		423	118	282	268
Chr1	4.2E+07	42300000		599	203	354	409
Chr1	4.2E+07	42400000		775	616	661	72
Chr1	4.2E+07	42500000		633	469	545	7
Chr1	4.3E+07	42600000		537	345	394	4
Chr1	4.3E+07	42700000		516	205	213	35
Chr1	4.3E+07	42800000		645	158	367	330
Chr1	4.3E+07	42900000		384	84	312	301
Chr1	4.3E+07	43000000		582	250	176	336
Chr1	4.3E+07	43100000		499	9	429	303
Chr1	4.3E+07	43200000		951	729	838	4
Chr1	4.3E+07	43300000		374	276	312	5
Chr2	Chr2						
Chr2	1	100000		423	407	12	409
Chr2	100001	200000		398	378	14	383
Chr2	200001	300000		377	370	9	356
Chr2	300001	400000		204	196	6	197
Chr2	400001	500000		321	168	233	166
Chr2	500001	600000		460	240	332	283
Chr2	600001	700000		467	261	328	195
Chr2	700001	800000		490	275	421	126
Chr2	800001	900000		503	384	444	72
Chr2	900001	1000000		625	416	332	381
Chr2	1000001	1100000		588	389	480	214
Chr2	1100001	1200000		458	408	82	391
Chr2	1200001	1300000		415	397	12	399
Chr2	1300001	1400000		552	377	412	200

					SNP		
				All	Bengal/Nona Bokra	PSSR/Bengal	PSSR/Nona Bokra
Chr2	1400001	1500000		645	362	550	252
Chr2	1500001	1600000		523	11	510	451
Chr2	1600001	1700000		32	22	29	3
Chr2	1700001	1800000		24	10	11	9
Chr2	1800001	1900000		22	9	14	8
Chr2	1900001	2000000		15	3	4	3
Chr2	2000001	2100000		471	329	221	315
Chr2	2100001	2200000		881	458	655	331
Chr2	2200001	2300000		735	252	532	179
Chr2	2300001	2400000		723	316	382	258
Chr2	2400001	2500000		547	52	213	155
Chr2	2500001	2600000		321	31	74	49
Chr2	2600001	2700000		309	143	202	174
Chr2	2700001	2800000		431	189	316	263
Chr2	2800001	2900000		374	329	62	323
Chr2	2900001	3000000		1122	747	10	580
Chr2	3000001	3100000		886	616	44	616
Chr2	3100001	3200000		366	299	68	296
Chr2	3200001	3300000		522	112	208	291
Chr2	3300001	3400000		409	7	6	5
Chr2	3400001	3500000		415	2	7	8
Chr2	3500001	3600000		46	17	22	7
Chr2	3600001	3700000		76	13	10	8
Chr2	3700001	3800000		168	12	8	9
Chr2	3800001	3900000		507	9	402	338
Chr2	3900001	4000000		710	6	540	444
Chr2	4000001	4100000		523	26	285	284
Chr2	4100001	4200000		217	183	142	59
Chr2	4200001	4300000		664	501	633	8
Chr2	4300001	4400000		916	375	628	341
Chr2	4400001	4500000		690	205	219	110
Chr2	4500001	4600000		956	224	250	161
Chr2	4600001	4700000		518	69	30	60
Chr2	4700001	4800000		957	71	349	276
Chr2	4800001	4900000		1032	486	447	410
Chr2	4900001	5000000		469	402	14	393
Chr2	5000001	5100000		553	421	260	347
Chr2	5100001	5200000		706	470	568	254
Chr2	5200001	5300000		644	426	510	246
Chr2	5300001	5400000		606	388	463	176
Chr2	5400001	5500000		706	482	588	184
Chr2	5500001	5600000		747	480	622	189
Chr2	5600001	5700000		863	391	678	471
Chr2	5700001	5800000		724	499	586	228
Chr2	5800001	5900000		758	482	628	196

					SNP		
				All	Bengal/Nona Bokra	PSSR/Bengal	PSSR/Nona Bokra
Chr2	5900001	6000000		837	544	641	225
Chr2	6000001	6100000		725	504	588	174
Chr2	6100001	6200000		781	487	617	207
Chr2	6200001	6300000		1089	783	757	197
Chr2	6300001	6400000		437	327	296	6
Chr2	6400001	6500000		622	482	523	2
Chr2	6500001	6600000		288	84	265	132
Chr2	6600001	6700000		611	469	551	3
Chr2	6700001	6800000		972	717	748	144
Chr2	6800001	6900000		1215	737	829	49
Chr2	6900001	7000000		1662	1166	1271	3
Chr2	7000001	7100000		907	445	612	7
Chr2	7100001	7200000		1008	521	574	37
Chr2	7200001	7300000		1018	586	708	26
Chr2	7300001	7400000		1088	656	875	302
Chr2	7400001	7500000		1083	581	775	388
Chr2	7500001	7600000		834	579	694	12
Chr2	7600001	7700000		627	500	580	5
Chr2	7700001	7800000		816	620	780	5
Chr2	7800001	7900000		841	612	781	13
Chr2	7900001	8000000		1008	658	732	87
Chr2	8000001	8100000		773	63	708	597
Chr2	8100001	8200000		891	15	751	636
Chr2	8200001	8300000		1473	560	768	438
Chr2	8300001	8400000		995	376	741	485
Chr2	8400001	8500000		1918	1140	1540	709
Chr2	8500001	8600000		275	175	74	134
Chr2	8600001	8700000		297	132	149	5
Chr2	8700001	8800000		386	166	309	196
Chr2	8800001	8900000		754	34	686	578
Chr2	8900001	9000000		934	546	804	57
Chr2	9000001	9100000		623	441	561	7
Chr2	9100001	9200000		372	241	252	5
Chr2	9200001	9300000		272	163	184	13
Chr2	9300001	9400000		108	65	67	3
Chr2	9400001	9500000		726	447	584	7
Chr2	9500001	9600000		428	308	387	13
Chr2	9600001	9700000		32	15	21	4
Chr2	9700001	9800000		47	20	29	5
Chr2	9800001	9900000		247	168	12	164
Chr2	9900001	10000000		1218	471	733	796
Chr2	1E+07	10100000		1044	374	676	581
Chr2	1E+07	10200000		1012	644	459	559
Chr2	1E+07	10300000		806	435	443	330
Chr2	1E+07	10400000		1061	423	748	592

					SNP		
				All	Bengal/Nona Bokra	PSSR/Bengal	PSSR/Nona Bokra
Chr2	1E+07	10500000		689	395	466	271
Chr2	1.1E+07	10600000		1117	549	582	476
Chr2	1.1E+07	10700000		1116	710	803	262
Chr2	1.1E+07	10800000		958	570	718	354
Chr2	1.1E+07	10900000		1124	526	812	650
Chr2	1.1E+07	11000000		1191	511	714	612
Chr2	1.1E+07	11100000		1322	460	1005	816
Chr2	1.1E+07	11200000		868	491	599	390
Chr2	1.1E+07	11300000		935	586	716	418
Chr2	1.1E+07	11400000		728	454	657	65
Chr2	1.1E+07	11500000		948	580	809	299
Chr2	1.2E+07	11600000		730	493	655	58
Chr2	1.2E+07	11700000		795	543	697	7
Chr2	1.2E+07	11800000		549	393	431	8
Chr2	1.2E+07	11900000		349	238	291	3
Chr2	1.2E+07	12000000		139	64	109	6
Chr2	1.2E+07	12100000		700	520	663	7
Chr2	1.2E+07	12200000		892	662	821	14
Chr2	1.2E+07	12300000		905	651	850	14
Chr2	1.2E+07	12400000		563	404	516	4
Chr2	1.2E+07	12500000		813	608	781	6
Chr2	1.3E+07	12600000		818	623	762	11
Chr2	1.3E+07	12700000		714	542	661	7
Chr2	1.3E+07	12800000		905	701	689	7
Chr2	1.3E+07	12900000		646	464	599	5
Chr2	1.3E+07	13000000		646	425	598	8
Chr2	1.3E+07	13100000		630	479	589	8
Chr2	1.3E+07	13200000		516	374	444	14
Chr2	1.3E+07	13300000		611	457	576	8
Chr2	1.3E+07	13400000		514	389	486	13
Chr2	1.3E+07	13500000		583	412	486	9
Chr2	1.4E+07	13600000		757	532	712	14
Chr2	1.4E+07	13700000		270	154	154	8
Chr2	1.4E+07	13800000		242	149	176	12
Chr2	1.4E+07	13900000		570	403	505	8
Chr2	1.4E+07	14000000		673	493	609	21
Chr2	1.4E+07	14100000		600	465	544	7
Chr2	1.4E+07	14200000		655	447	596	8
Chr2	1.4E+07	14300000		104	30	64	6
Chr2	1.4E+07	14400000		779	540	710	10
Chr2	1.4E+07	14500000		803	629	759	11
Chr2	1.5E+07	14600000		739	539	671	9
Chr2	1.5E+07	14700000		792	587	734	9
Chr2	1.5E+07	14800000		857	653	811	10
Chr2	1.5E+07	14900000		754	576	699	9

					SNP		
				All	Bengal/Nona Bokra	PSSR/Bengal	PSSR/Nona Bokra
Chr2	1.5E+07	15000000		477	345	436	5
Chr2	1.5E+07	15100000		752	585	704	12
Chr2	1.5E+07	15200000		567	420	517	4
Chr2	1.5E+07	15300000		949	649	886	15
Chr2	1.5E+07	15400000		794	578	753	10
Chr2	1.5E+07	15500000		883	659	824	4
Chr2	1.6E+07	15600000		423	303	365	7
Chr2	1.6E+07	15700000		652	415	487	241
Chr2	1.6E+07	15800000		741	476	608	169
Chr2	1.6E+07	15900000		595	435	553	0
Chr2	1.6E+07	16000000		460	369	427	5
Chr2	1.6E+07	16100000		783	563	710	14
Chr2	1.6E+07	16200000		553	398	510	3
Chr2	1.6E+07	16300000		543	380	507	6
Chr2	1.6E+07	16400000		814	514	674	2
Chr2	1.6E+07	16500000		908	303	18	287
Chr2	1.7E+07	16600000		849	402	11	380
Chr2	1.7E+07	16700000		1318	402	420	328
Chr2	1.7E+07	16800000		1311	206	250	137
Chr2	1.7E+07	16900000		1487	251	302	147
Chr2	1.7E+07	17000000		914	244	337	158
Chr2	1.7E+07	17100000		1179	466	481	300
Chr2	1.7E+07	17200000		890	507	631	366
Chr2	1.7E+07	17300000		907	560	671	359
Chr2	1.7E+07	17400000		644	396	480	284
Chr2	1.7E+07	17500000		744	455	540	286
Chr2	1.8E+07	17600000		834	517	554	435
Chr2	1.8E+07	17700000		348	198	267	25
Chr2	1.8E+07	17800000		377	249	225	22
Chr2	1.8E+07	17900000		855	289	259	278
Chr2	1.8E+07	18000000		997	373	320	381
Chr2	1.8E+07	18100000		1148	381	453	3
Chr2	1.8E+07	18200000		737	245	321	4
Chr2	1.8E+07	18300000		1210	138	139	65
Chr2	1.8E+07	18400000		886	303	319	229
Chr2	1.8E+07	18500000		1238	529	487	417
Chr2	1.9E+07	18600000		1254	626	756	349
Chr2	1.9E+07	18700000		852	545	696	205
Chr2	1.9E+07	18800000		776	573	746	6
Chr2	1.9E+07	18900000		877	556	561	261
Chr2	1.9E+07	19000000		806	513	477	296
Chr2	1.9E+07	19100000		654	466	252	500
Chr2	1.9E+07	19200000		1077	579	655	376
Chr2	1.9E+07	19300000		1028	480	572	83
Chr2	1.9E+07	19400000		923	400	427	169

					SNP		
				All	Bengal/Nona Bokra	PSSR/Bengal	PSSR/Nona Bokra
Chr2	1.9E+07	19500000		778	405	472	10
Chr2	2E+07	19600000		273	192	246	9
Chr2	2E+07	19700000		780	583	730	5
Chr2	2E+07	19800000		883	637	812	6
Chr2	2E+07	19900000		733	578	622	9
Chr2	2E+07	20000000		773	569	712	7
Chr2	2E+07	20100000		796	604	734	7
Chr2	2E+07	20200000		931	776	769	7
Chr2	2E+07	20300000		626	397	475	207
Chr2	2E+07	20400000		919	553	735	172
Chr2	2E+07	20500000		680	457	447	64
Chr2	2.1E+07	20600000		1130	581	795	311
Chr2	2.1E+07	20700000		740	468	611	100
Chr2	2.1E+07	20800000		738	476	598	222
Chr2	2.1E+07	20900000		675	442	509	186
Chr2	2.1E+07	21000000		705	414	546	275
Chr2	2.1E+07	21100000		834	680	793	6
Chr2	2.1E+07	21200000		595	440	515	70
Chr2	2.1E+07	21300000		504	342	484	15
Chr2	2.1E+07	21400000		512	299	366	234
Chr2	2.1E+07	21500000		513	398	457	6
Chr2	2.2E+07	21600000		700	538	643	68
Chr2	2.2E+07	21700000		736	525	624	14
Chr2	2.2E+07	21800000		727	537	671	6
Chr2	2.2E+07	21900000		982	686	867	7
Chr2	2.2E+07	22000000		941	625	750	196
Chr2	2.2E+07	22100000		814	567	754	13
Chr2	2.2E+07	22200000		871	431	620	464
Chr2	2.2E+07	22300000		845	541	600	311
Chr2	2.2E+07	22400000		688	490	624	91
Chr2	2.2E+07	22500000		658	544	574	4
Chr2	2.3E+07	22600000		718	567	515	7
Chr2	2.3E+07	22700000		678	511	626	3
Chr2	2.3E+07	22800000		502	380	470	7
Chr2	2.3E+07	22900000		496	418	475	7
Chr2	2.3E+07	23000000		19	10	9	8
Chr2	2.3E+07	23100000		569	405	523	11
Chr2	2.3E+07	23200000		573	313	474	175
Chr2	2.3E+07	23300000		420	217	300	200
Chr2	2.3E+07	23400000		156	99	51	92
Chr2	2.3E+07	23500000		511	275	282	268
Chr2	2.4E+07	23600000		759	523	437	449
Chr2	2.4E+07	23700000		612	221	526	349
Chr2	2.4E+07	23800000		163	15	143	123
Chr2	2.4E+07	23900000		344	40	306	266

					SNP		
				All	Bengal/Nona Bokra	PSSR/Bengal	PSSR/Nona Bokra
Chr2	2.4E+07	24000000		643	128	624	368
Chr2	2.4E+07	24100000		621	463	594	9
Chr2	2.4E+07	24200000		823	390	622	456
Chr2	2.4E+07	24300000		1113	546	438	598
Chr2	2.4E+07	24400000		951	185	105	78
Chr2	2.4E+07	24500000		651	265	148	119
Chr2	2.5E+07	24600000		796	409	438	358
Chr2	2.5E+07	24700000		1141	449	401	489
Chr2	2.5E+07	24800000		965	428	599	488
Chr2	2.5E+07	24900000		709	238	518	439
Chr2	2.5E+07	25000000		788	37	670	562
Chr2	2.5E+07	25100000		816	57	691	576
Chr2	2.5E+07	25200000		596	111	441	369
Chr2	2.5E+07	25300000		927	532	234	430
Chr2	2.5E+07	25400000		1165	536	572	3
Chr2	2.5E+07	25500000		746	402	548	5
Chr2	2.6E+07	25600000		617	433	584	5
Chr2	2.6E+07	25700000		597	457	563	4
Chr2	2.6E+07	25800000		535	448	513	7
Chr2	2.6E+07	25900000		704	540	631	42
Chr2	2.6E+07	26000000		502	329	396	139
Chr2	2.6E+07	26100000		1198	643	990	415
Chr2	2.6E+07	26200000		1052	495	620	97
Chr2	2.6E+07	26300000		721	425	547	203
Chr2	2.6E+07	26400000		775	458	677	229
Chr2	2.6E+07	26500000		614	65	543	502
Chr2	2.7E+07	26600000		668	88	602	508
Chr2	2.7E+07	26700000		722	27	680	582
Chr2	2.7E+07	26800000		622	14	603	517
Chr2	2.7E+07	26900000		459	82	424	343
Chr2	2.7E+07	27000000		591	359	482	220
Chr2	2.7E+07	27100000		600	401	494	177
Chr2	2.7E+07	27200000		995	682	797	343
Chr2	2.7E+07	27300000		575	365	463	185
Chr2	2.7E+07	27400000		661	463	555	132
Chr2	2.7E+07	27500000		806	537	648	267
Chr2	2.8E+07	27600000		657	458	585	56
Chr2	2.8E+07	27700000		823	581	755	6
Chr2	2.8E+07	27800000		630	442	573	4
Chr2	2.8E+07	27900000		655	495	608	3
Chr2	2.8E+07	28000000		648	442	610	2
Chr2	2.8E+07	28100000		466	288	415	9
Chr2	2.8E+07	28200000		623	467	582	5
Chr2	2.8E+07	28300000		588	424	545	9
Chr2	2.8E+07	28400000		616	460	565	8

					SNP		
				All	Bengal/Nona Bokra	PSSR/Bengal	PSSR/Nona Bokra
Chr2	2.8E+07	28500000		645	451	575	12
Chr2	2.9E+07	28600000		586	404	497	51
Chr2	2.9E+07	28700000		573	369	462	141
Chr2	2.9E+07	28800000		528	186	455	212
Chr2	2.9E+07	28900000		812	15	736	637
Chr2	2.9E+07	29000000		622	20	556	480
Chr2	2.9E+07	29100000		423	10	407	355
Chr2	2.9E+07	29200000		593	298	427	250
Chr2	2.9E+07	29300000		595	349	429	325
Chr2	2.9E+07	29400000		659	455	546	177
Chr2	2.9E+07	29500000		851	498	617	425
Chr2	3E+07	29600000		619	213	414	396
Chr2	3E+07	29700000		44	21	28	17
Chr2	3E+07	29800000		592	290	492	311
Chr2	3E+07	29900000		691	478	548	225
Chr2	3E+07	30000000		668	427	527	259
Chr2	3E+07	30100000		627	395	520	206
Chr2	3E+07	30200000		549	342	425	184
Chr2	3E+07	30300000		574	307	379	234
Chr2	3E+07	30400000		636	425	465	273
Chr2	3E+07	30500000		658	251	528	421
Chr2	3.1E+07	30600000		507	18	479	442
Chr2	3.1E+07	30700000		713	194	621	440
Chr2	3.1E+07	30800000		588	380	455	218
Chr2	3.1E+07	30900000		588	368	451	185
Chr2	3.1E+07	31000000		728	459	551	217
Chr2	3.1E+07	31100000		606	378	461	268
Chr2	3.1E+07	31200000		659	437	510	254
Chr2	3.1E+07	31300000		770	479	581	342
Chr2	3.1E+07	31400000		590	361	426	192
Chr2	3.1E+07	31500000		728	430	535	257
Chr2	3.2E+07	31600000		551	371	443	160
Chr2	3.2E+07	31700000		519	392	510	13
Chr2	3.2E+07	31800000		503	411	481	7
Chr2	3.2E+07	31900000		452	333	386	7
Chr2	3.2E+07	32000000		642	510	623	9
Chr2	3.2E+07	32100000		726	567	677	5
Chr2	3.2E+07	32200000		535	390	512	7
Chr2	3.2E+07	32300000		489	359	444	5
Chr2	3.2E+07	32400000		553	447	507	44
Chr2	3.2E+07	32500000		941	612	721	146
Chr2	3.3E+07	32600000		547	392	499	7
Chr2	3.3E+07	32700000		575	450	529	49
Chr2	3.3E+07	32800000		410	310	390	35
Chr2	3.3E+07	32900000		740	607	686	22

					SNP		
				All	Bengal/Nona Bokra	PSSR/Bengal	PSSR/Nona Bokra
Chr2	3.3E+07	33000000		551	431	522	6
Chr2	3.3E+07	33100000		578	416	501	47
Chr2	3.3E+07	33200000		691	508	577	166
Chr2	3.3E+07	33300000		779	481	598	254
Chr2	3.3E+07	33400000		534	314	429	176
Chr2	3.3E+07	33500000		743	559	644	152
Chr2	3.4E+07	33600000		701	447	585	166
Chr2	3.4E+07	33700000		805	512	672	265
Chr2	3.4E+07	33800000		428	360	416	6
Chr2	3.4E+07	33900000		616	441	543	138
Chr2	3.4E+07	34000000		621	349	453	290
Chr2	3.4E+07	34100000		568	329	307	355
Chr2	3.4E+07	34200000		190	106	96	102
Chr2	3.4E+07	34300000		846	403	617	499
Chr2	3.4E+07	34400000		504	330	407	178
Chr2	3.4E+07	34500000		872	587	651	305
Chr2	3.5E+07	34600000		675	439	504	159
Chr2	3.5E+07	34700000		634	437	534	139
Chr2	3.5E+07	34800000		534	22	21	5
Chr2	3.5E+07	34900000		722	178	19	177
Chr2	3.5E+07	35000000		422	117	9	118
Chr2	3.5E+07	35100000		593	270	8	260
Chr2	3.5E+07	35200000		793	597	40	478
Chr2	3.5E+07	35300000		510	69	81	38
Chr2	3.5E+07	35400000		605	482	580	16
Chr2	3.5E+07	35500000		385	263	291	9
Chr2	3.6E+07	35600000		462	382	442	6
Chr2	3.6E+07	35700000		400	297	369	55
Chr2	3.6E+07	35800000		490	324	386	164
Chr2	3.6E+07	35900000		792	518	644	222
Chr2	3.6E+07	36000000		236	145	195	65
Chr3	Chr3						
Chr3	1	100000		43	18	23	9
Chr3	100001	200000		89	77	14	74
Chr3	200001	300000		590	428	475	65
Chr3	300001	400000		414	330	367	5
Chr3	400001	500000		455	379	431	14
Chr3	500001	600000		581	460	548	5
Chr3	600001	700000		505	405	476	5
Chr3	700001	800000		505	428	469	4
Chr3	800001	900000		773	481	567	6
Chr3	900001	1000000		614	394	465	16
Chr3	1000001	1100000		631	419	484	11
Chr3	1100001	1200000		636	474	539	4
Chr3	1200001	1300000		762	494	603	9

					SNP		
				All	Bengal/Nona Bokra	PSSR/Bengal	PSSR/Nona Bokra
Chr3	1300001	1400000		585	442	525	6
Chr3	1400001	1500000		864	457	548	2
Chr3	1500001	1600000		631	335	389	3
Chr3	1600001	1700000		318	206	306	36
Chr3	1700001	1800000		573	462	547	42
Chr3	1800001	1900000		464	367	451	7
Chr3	1900001	2000000		545	413	493	12
Chr3	2000001	2100000		581	455	528	36
Chr3	2100001	2200000		432	407	17	410
Chr3	2200001	2300000		677	481	412	279
Chr3	2300001	2400000		441	336	408	11
Chr3	2400001	2500000		427	322	383	8
Chr3	2500001	2600000		412	344	402	4
Chr3	2600001	2700000		581	422	567	11
Chr3	2700001	2800000		740	465	697	133
Chr3	2800001	2900000		442	335	417	3
Chr3	2900001	3000000		533	390	492	13
Chr3	3000001	3100000		524	378	464	7
Chr3	3100001	3200000		468	376	456	4
Chr3	3200001	3300000		487	394	462	18
Chr3	3300001	3400000		727	518	646	80
Chr3	3400001	3500000		554	437	529	9
Chr3	3500001	3600000		429	340	406	6
Chr3	3600001	3700000		480	395	468	2
Chr3	3700001	3800000		477	357	451	27
Chr3	3800001	3900000		747	471	530	8
Chr3	3900001	4000000		560	179	202	13
Chr3	4000001	4100000		662	304	375	5
Chr3	4100001	4200000		442	252	291	6
Chr3	4200001	4300000		485	274	342	30
Chr3	4300001	4400000		752	431	542	10
Chr3	4400001	4500000		698	449	528	4
Chr3	4500001	4600000		594	413	488	8
Chr3	4600001	4700000		963	593	718	317
Chr3	4700001	4800000		732	443	493	340
Chr3	4800001	4900000		763	462	598	228
Chr3	4900001	5000000		625	507	574	5
Chr3	5000001	5100000		744	529	646	5
Chr3	5100001	5200000		870	664	811	3
Chr3	5200001	5300000		673	512	635	5
Chr3	5300001	5400000		491	307	362	4
Chr3	5400001	5500000		599	444	523	2
Chr3	5500001	5600000		573	443	540	20
Chr3	5600001	5700000		564	427	517	13
Chr3	5700001	5800000		634	523	606	3

					SNP		
				All	Bengal/Nona Bokra	PSSR/Bengal	PSSR/Nona Bokra
Chr3	5800001	5900000		551	423	511	14
Chr3	5900001	6000000		359	287	346	3
Chr3	6000001	6100000		498	368	383	149
Chr3	6100001	6200000		450	336	400	22
Chr3	6200001	6300000		436	345	413	7
Chr3	6300001	6400000		560	412	532	7
Chr3	6400001	6500000		538	401	494	5
Chr3	6500001	6600000		472	346	425	10
Chr3	6600001	6700000		469	345	428	5
Chr3	6700001	6800000		588	452	499	66
Chr3	6800001	6900000		651	500	351	344
Chr3	6900001	7000000		771	535	625	255
Chr3	7000001	7100000		747	521	619	202
Chr3	7100001	7200000		530	337	405	181
Chr3	7200001	7300000		691	433	544	199
Chr3	7300001	7400000		604	392	468	185
Chr3	7400001	7500000		470	265	370	151
Chr3	7500001	7600000		543	344	437	157
Chr3	7600001	7700000		677	434	538	228
Chr3	7700001	7800000		720	434	592	194
Chr3	7800001	7900000		759	467	662	150
Chr3	7900001	8000000		693	480	597	127
Chr3	8000001	8100000		499	463	81	417
Chr3	8100001	8200000		692	428	542	149
Chr3	8200001	8300000		730	399	452	108
Chr3	8300001	8400000		813	425	498	127
Chr3	8400001	8500000		637	347	449	46
Chr3	8500001	8600000		605	407	502	106
Chr3	8600001	8700000		609	398	476	216
Chr3	8700001	8800000		576	353	466	220
Chr3	8800001	8900000		513	336	406	173
Chr3	8900001	9000000		725	441	582	220
Chr3	9000001	9100000		498	333	401	145
Chr3	9100001	9200000		694	399	562	184
Chr3	9200001	9300000		698	452	539	258
Chr3	9300001	9400000		706	441	534	183
Chr3	9400001	9500000		839	536	646	265
Chr3	9500001	9600000		673	429	519	235
Chr3	9600001	9700000		737	460	609	220
Chr3	9700001	9800000		562	376	463	195
Chr3	9800001	9900000		774	517	608	205
Chr3	9900001	10000000		788	516	659	249
Chr3	1E+07	10100000		750	516	614	196
Chr3	1E+07	10200000		751	469	618	266
Chr3	1E+07	10300000		740	458	591	261

					SNP		
				All	Bengal/Nona Bokra	PSSR/Bengal	PSSR/Nona Bokra
Chr3	1E+07	10400000		733	227	89	254
Chr3	1E+07	10500000		870	320	245	313
Chr3	1.1E+07	10600000		900	356	443	307
Chr3	1.1E+07	10700000		792	214	238	170
Chr3	1.1E+07	10800000		893	283	374	211
Chr3	1.1E+07	10900000		751	240	205	223
Chr3	1.1E+07	11000000		641	195	65	212
Chr3	1.1E+07	11100000		833	276	4	279
Chr3	1.1E+07	11200000		700	225	14	226
Chr3	1.1E+07	11300000		810	209	12	217
Chr3	1.1E+07	11400000		940	373	26	361
Chr3	1.1E+07	11500000		970	269	6	264
Chr3	1.2E+07	11600000		831	284	9	271
Chr3	1.2E+07	11700000		615	196	11	194
Chr3	1.2E+07	11800000		723	249	132	230
Chr3	1.2E+07	11900000		717	463	543	217
Chr3	1.2E+07	12000000		784	405	597	377
Chr3	1.2E+07	12100000		701	74	642	567
Chr3	1.2E+07	12200000		628	27	577	498
Chr3	1.2E+07	12300000		527	92	458	410
Chr3	1.2E+07	12400000		647	354	526	260
Chr3	1.2E+07	12500000		568	366	421	221
Chr3	1.3E+07	12600000		725	488	594	212
Chr3	1.3E+07	12700000		762	470	578	305
Chr3	1.3E+07	12800000		656	444	355	378
Chr3	1.3E+07	12900000		432	425	13	419
Chr3	1.3E+07	13000000		621	594	26	600
Chr3	1.3E+07	13100000		174	158	24	149
Chr3	1.3E+07	13200000		284	220	268	3
Chr3	1.3E+07	13300000		623	474	606	3
Chr3	1.3E+07	13400000		215	106	153	8
Chr3	1.3E+07	13500000		260	94	173	14
Chr3	1.4E+07	13600000		105	44	81	4
Chr3	1.4E+07	13700000		328	235	302	5
Chr3	1.4E+07	13800000		541	427	519	6
Chr3	1.4E+07	13900000		525	389	501	3
Chr3	1.4E+07	14000000		670	520	634	7
Chr3	1.4E+07	14100000		806	627	752	5
Chr3	1.4E+07	14200000		461	355	352	3
Chr3	1.4E+07	14300000		979	738	780	134
Chr3	1.4E+07	14400000		611	436	570	27
Chr3	1.4E+07	14500000		848	671	693	11
Chr3	1.5E+07	14600000		782	566	709	11
Chr3	1.5E+07	14700000		767	566	716	8
Chr3	1.5E+07	14800000		331	226	297	15

					SNP		
				All	Bengal/Nona Bokra	PSSR/Bengal	PSSR/Nona Bokra
Chr3	1.5E+07	14900000		330	190	259	94
Chr3	1.5E+07	15000000		687	499	518	4
Chr3	1.5E+07	15100000		916	581	651	12
Chr3	1.5E+07	15200000		822	601	694	2
Chr3	1.5E+07	15300000		559	429	506	2
Chr3	1.5E+07	15400000		1064	809	809	27
Chr3	1.5E+07	15500000		620	486	583	2
Chr3	1.6E+07	15600000		588	440	564	6
Chr3	1.6E+07	15700000		959	734	782	6
Chr3	1.6E+07	15800000		800	598	754	5
Chr3	1.6E+07	15900000		881	703	792	9
Chr3	1.6E+07	16000000		799	462	584	386
Chr3	1.6E+07	16100000		742	389	505	413
Chr3	1.6E+07	16200000		1005	582	755	445
Chr3	1.6E+07	16300000		886	521	637	252
Chr3	1.6E+07	16400000		834	487	718	9
Chr3	1.6E+07	16500000		1025	433	524	175
Chr3	1.7E+07	16600000		1096	398	501	155
Chr3	1.7E+07	16700000		960	507	756	449
Chr3	1.7E+07	16800000		1233	724	857	566
Chr3	1.7E+07	16900000		1107	593	810	554
Chr3	1.7E+07	17000000		904	614	775	184
Chr3	1.7E+07	17100000		882	525	719	251
Chr3	1.7E+07	17200000		752	493	628	155
Chr3	1.7E+07	17300000		703	548	548	8
Chr3	1.7E+07	17400000		914	647	873	16
Chr3	1.7E+07	17500000		656	511	638	4
Chr3	1.8E+07	17600000		828	629	788	10
Chr3	1.8E+07	17700000		784	574	734	11
Chr3	1.8E+07	17800000		598	456	559	5
Chr3	1.8E+07	17900000		747	521	690	10
Chr3	1.8E+07	18000000		908	651	870	13
Chr3	1.8E+07	18100000		861	644	792	11
Chr3	1.8E+07	18200000		764	557	711	11
Chr3	1.8E+07	18300000		816	564	735	13
Chr3	1.8E+07	18400000		214	115	180	8
Chr3	1.8E+07	18500000		427	300	379	4
Chr3	1.9E+07	18600000		677	476	621	11
Chr3	1.9E+07	18700000		609	398	577	7
Chr3	1.9E+07	18800000		866	620	791	9
Chr3	1.9E+07	18900000		612	432	546	10
Chr3	1.9E+07	19000000		587	379	539	10
Chr3	1.9E+07	19100000		941	675	877	12
Chr3	1.9E+07	19200000		778	536	732	16
Chr3	1.9E+07	19300000		794	556	737	10

					SNP		
				All	Bengal/Nona Bokra	PSSR/Bengal	PSSR/Nona Bokra
Chr3	1.9E+07	19400000		561	327	522	12
Chr3	1.9E+07	19500000		778	557	739	13
Chr3	2E+07	19600000		503	321	435	8
Chr3	2E+07	19700000		746	541	682	8
Chr3	2E+07	19800000		784	549	738	10
Chr3	2E+07	19900000		829	633	792	8
Chr3	2E+07	20000000		830	612	781	6
Chr3	2E+07	20100000		743	608	706	11
Chr3	2E+07	20200000		839	578	780	11
Chr3	2E+07	20300000		941	669	897	14
Chr3	2E+07	20400000		794	573	741	11
Chr3	2E+07	20500000		776	557	706	3
Chr3	2.1E+07	20600000		731	505	697	4
Chr3	2.1E+07	20700000		603	412	560	6
Chr3	2.1E+07	20800000		741	548	680	12
Chr3	2.1E+07	20900000		750	497	697	13
Chr3	2.1E+07	21000000		768	536	716	9
Chr3	2.1E+07	21100000		879	523	669	322
Chr3	2.1E+07	21200000		942	670	841	114
Chr3	2.1E+07	21300000		852	639	779	12
Chr3	2.1E+07	21400000		461	290	370	136
Chr3	2.1E+07	21500000		807	435	608	299
Chr3	2.2E+07	21600000		715	381	577	178
Chr3	2.2E+07	21700000		603	407	516	136
Chr3	2.2E+07	21800000		629	371	497	231
Chr3	2.2E+07	21900000		262	140	196	48
Chr3	2.2E+07	22000000		886	566	725	172
Chr3	2.2E+07	22100000		1026	626	833	318
Chr3	2.2E+07	22200000		935	486	720	340
Chr3	2.2E+07	22300000		825	509	718	241
Chr3	2.2E+07	22400000		988	642	771	366
Chr3	2.2E+07	22500000		847	575	792	11
Chr3	2.3E+07	22600000		807	550	764	6
Chr3	2.3E+07	22700000		743	540	676	12
Chr3	2.3E+07	22800000		620	506	506	2
Chr3	2.3E+07	22900000		978	813	860	13
Chr3	2.3E+07	23000000		721	471	648	80
Chr3	2.3E+07	23100000		949	671	823	161
Chr3	2.3E+07	23200000		798	281	654	146
Chr3	2.3E+07	23300000		1005	83	943	69
Chr3	2.3E+07	23400000		577	393	521	83
Chr3	2.3E+07	23500000		728	525	685	9
Chr3	2.4E+07	23600000		803	529	688	196
Chr3	2.4E+07	23700000		790	576	719	3
Chr3	2.4E+07	23800000		793	486	643	195

					SNP		
				All	Bengal/Nona Bokra	PSSR/Bengal	PSSR/Nona Bokra
Chr3	2.4E+07	23900000		731	480	617	48
Chr3	2.4E+07	24000000		801	595	756	13
Chr3	2.4E+07	24100000		489	315	424	4
Chr3	2.4E+07	24200000		328	238	281	8
Chr3	2.4E+07	24300000		841	624	779	7
Chr3	2.4E+07	24400000		914	626	840	14
Chr3	2.4E+07	24500000		815	532	644	289
Chr3	2.5E+07	24600000		834	538	625	277
Chr3	2.5E+07	24700000		944	537	646	499
Chr3	2.5E+07	24800000		813	493	492	359
Chr3	2.5E+07	24900000		937	497	625	427
Chr3	2.5E+07	25000000		848	466	564	471
Chr3	2.5E+07	25100000		680	406	438	338
Chr3	2.5E+07	25200000		389	273	338	57
Chr3	2.5E+07	25300000		174	146	19	149
Chr3	2.5E+07	25400000		478	439	11	445
Chr3	2.5E+07	25500000		566	532	9	531
Chr3	2.6E+07	25600000		111	99	13	96
Chr3	2.6E+07	25700000		41	23	20	7
Chr3	2.6E+07	25800000		67	40	41	12
Chr3	2.6E+07	25900000		37	17	25	8
Chr3	2.6E+07	26000000		111	29	33	9
Chr3	2.6E+07	26100000		34	22	23	10
Chr3	2.6E+07	26200000		57	27	37	20
Chr3	2.6E+07	26300000		51	40	36	9
Chr3	2.6E+07	26400000		29	9	9	8
Chr3	2.6E+07	26500000		193	155	19	167
Chr3	2.7E+07	26600000		610	476	364	208
Chr3	2.7E+07	26700000		1015	756	850	7
Chr3	2.7E+07	26800000		1113	591	801	477
Chr3	2.7E+07	26900000		888	631	788	115
Chr3	2.7E+07	27000000		510	406	468	8
Chr3	2.7E+07	27100000		848	671	629	75
Chr3	2.7E+07	27200000		731	162	649	456
Chr3	2.7E+07	27300000		551	180	489	312
Chr3	2.7E+07	27400000		577	436	563	5
Chr3	2.7E+07	27500000		586	432	563	9
Chr3	2.8E+07	27600000		149	11	138	127
Chr3	2.8E+07	27700000		388	42	366	314
Chr3	2.8E+07	27800000		819	530	721	76
Chr3	2.8E+07	27900000		731	390	564	341
Chr3	2.8E+07	28000000		863	418	627	447
Chr3	2.8E+07	28100000		781	435	608	351
Chr3	2.8E+07	28200000		659	459	546	169
Chr3	2.8E+07	28300000		485	336	463	6

					SNP		
				All	Bengal/Nona Bokra	PSSR/Bengal	PSSR/Nona Bokra
Chr3	2.8E+07	28400000		710	549	683	6
Chr3	2.8E+07	28500000		596	458	574	7
Chr3	2.9E+07	28600000		107	78	54	49
Chr3	2.9E+07	28700000		20	7	8	3
Chr3	2.9E+07	28800000		195	11	181	156
Chr3	2.9E+07	28900000		480	5	461	401
Chr3	2.9E+07	29000000		176	14	154	132
Chr3	2.9E+07	29100000		565	550	17	543
Chr3	2.9E+07	29200000		416	370	76	330
Chr3	2.9E+07	29300000		595	468	571	4
Chr3	2.9E+07	29400000		427	45	415	314
Chr3	2.9E+07	29500000		630	119	565	480
Chr3	3E+07	29600000		719	453	494	366
Chr3	3E+07	29700000		841	530	660	304
Chr3	3E+07	29800000		704	438	485	341
Chr3	3E+07	29900000		747	481	596	291
Chr3	3E+07	30000000		530	319	408	176
Chr3	3E+07	30100000		676	432	549	202
Chr3	3E+07	30200000		686	435	558	189
Chr3	3E+07	30300000		478	292	390	169
Chr3	3E+07	30400000		886	555	665	302
Chr3	3E+07	30500000		810	483	660	289
Chr3	3.1E+07	30600000		331	203	265	117
Chr3	3.1E+07	30700000		75	56	63	6
Chr3	3.1E+07	30800000		450	382	428	1
Chr3	3.1E+07	30900000		512	392	486	3
Chr3	3.1E+07	31000000		562	426	520	9
Chr3	3.1E+07	31100000		539	424	507	1
Chr3	3.1E+07	31200000		444	314	404	5
Chr3	3.1E+07	31300000		632	484	591	7
Chr3	3.1E+07	31400000		607	476	597	2
Chr3	3.1E+07	31500000		625	461	573	12
Chr3	3.2E+07	31600000		625	460	597	4
Chr3	3.2E+07	31700000		525	420	505	3
Chr3	3.2E+07	31800000		558	422	517	3
Chr3	3.2E+07	31900000		527	397	506	3
Chr3	3.2E+07	32000000		798	609	770	7
Chr3	3.2E+07	32100000		596	481	583	5
Chr3	3.2E+07	32200000		593	411	512	101
Chr3	3.2E+07	32300000		784	491	642	233
Chr3	3.2E+07	32400000		360	111	333	195
Chr3	3.2E+07	32500000		646	238	592	302
Chr3	3.3E+07	32600000		604	431	473	185
Chr3	3.3E+07	32700000		655	385	465	345
Chr3	3.3E+07	32800000		464	334	433	16

					SNP		
				All	Bengal/Nona Bokra	PSSR/Bengal	PSSR/Nona Bokra
Chr3	3.3E+07	32900000		489	341	460	5
Chr3	3.3E+07	33000000		648	471	622	3
Chr3	3.3E+07	33100000		537	448	520	1
Chr3	3.3E+07	33200000		703	556	693	2
Chr3	3.3E+07	33300000		877	667	826	13
Chr3	3.3E+07	33400000		553	463	540	1
Chr3	3.3E+07	33500000		475	378	455	3
Chr3	3.4E+07	33600000		82	64	81	1
Chr3	3.4E+07	33700000		8	6	8	2
Chr3	3.4E+07	33800000		17	13	16	0
Chr3	3.4E+07	33900000		370	259	335	1
Chr3	3.4E+07	34000000		518	409	496	3
Chr3	3.4E+07	34100000		622	494	537	11
Chr3	3.4E+07	34200000		468	300	419	71
Chr3	3.4E+07	34300000		708	473	603	162
Chr3	3.4E+07	34400000		850	542	685	292
Chr3	3.4E+07	34500000		701	343	532	420
Chr3	3.5E+07	34600000		599	13	582	531
Chr3	3.5E+07	34700000		726	514	540	223
Chr3	3.5E+07	34800000		623	412	432	306
Chr3	3.5E+07	34900000		557	534	22	537
Chr3	3.5E+07	35000000		206	202	8	198
Chr3	3.5E+07	35100000		20	14	15	9
Chr3	3.5E+07	35200000		18	12	12	9
Chr3	3.5E+07	35300000		671	628	36	641
Chr3	3.5E+07	35400000		894	705	714	156
Chr3	3.5E+07	35500000		736	572	582	73
Chr3	3.6E+07	35600000		629	328	416	399
Chr3	3.6E+07	35700000		476	289	278	277
Chr3	3.6E+07	35800000		420	324	175	213
Chr3	3.6E+07	35900000		372	266	269	162
Chr3	3.6E+07	36000000		354	289	346	8
Chr3	3.6E+07	36100000		182	132	168	9
Chr3	3.6E+07	36200000		281	267	36	249
Chr3	3.6E+07	36300000		379	359	14	367
Chr3	3.6E+07	36400000		396	376	13	377
Chr3	3.6E+07	36500000		44	34	3	26
Chr4	Chr4						
Chr4	1	100000		893	233	141	211
Chr4	100001	200000		1409	721	314	728
Chr4	200001	300000		1274	589	364	564
Chr4	300001	400000		1035	604	354	562
Chr4	400001	500000		1131	598	563	528
Chr4	500001	600000		762	411	275	386
Chr4	600001	700000		1504	594	321	511

					SNP		
				All	Bengal/Nona Bokra	PSSR/Bengal	PSSR/Nona Bokra
Chr4	700001	800000		812	448	396	322
Chr4	800001	900000		506	178	275	154
Chr4	900001	1000000		1261	527	334	481
Chr4	1000001	1100000		902	427	319	443
Chr4	1100001	1200000		526	270	307	111
Chr4	1200001	1300000		959	510	515	14
Chr4	1300001	1400000		520	39	38	0
Chr4	1400001	1500000		354	27	23	9
Chr4	1500001	1600000		254	16	35	22
Chr4	1600001	1700000		540	67	74	31
Chr4	1700001	1800000		1327	637	496	8
Chr4	1800001	1900000		877	464	566	12
Chr4	1900001	2000000		618	261	352	5
Chr4	2000001	2100000		767	312	301	153
Chr4	2100001	2200000		188	6	5	8
Chr4	2200001	2300000		219	12	16	17
Chr4	2300001	2400000		454	40	37	25
Chr4	2400001	2500000		1064	330	366	345
Chr4	2500001	2600000		740	268	344	187
Chr4	2600001	2700000		503	10	14	110
Chr4	2700001	2800000		661	180	274	147
Chr4	2800001	2900000		997	388	442	325
Chr4	2900001	3000000		832	189	191	176
Chr4	3000001	3100000		159	3	7	18
Chr4	3100001	3200000		289	12	18	8
Chr4	3200001	3300000		374	61	61	40
Chr4	3300001	3400000		618	338	148	213
Chr4	3400001	3500000		756	231	70	134
Chr4	3500001	3600000		782	280	140	173
Chr4	3600001	3700000		983	272	329	123
Chr4	3700001	3800000		827	185	208	95
Chr4	3800001	3900000		732	187	269	154
Chr4	3900001	4000000		611	219	272	134
Chr4	4000001	4100000		960	418	394	283
Chr4	4100001	4200000		1116	547	496	471
Chr4	4200001	4300000		1046	559	540	530
Chr4	4300001	4400000		775	8	454	386
Chr4	4400001	4500000		956	6	499	407
Chr4	4500001	4600000		817	187	480	379
Chr4	4600001	4700000		984	455	479	544
Chr4	4700001	4800000		794	15	754	632
Chr4	4800001	4900000		391	308	85	50
Chr4	4900001	5000000		585	429	275	134
Chr4	5000001	5100000		1015	641	727	432
Chr4	5100001	5200000		1442	718	732	553

					SNP		
				All	Bengal/Nona Bokra	PSSR/Bengal	PSSR/Nona Bokra
Chr4	5200001	5300000		991	520	566	104
Chr4	5300001	5400000		945	716	660	55
Chr4	5400001	5500000		616	496	520	15
Chr4	5500001	5600000		807	584	753	2
Chr4	5600001	5700000		483	353	460	5
Chr4	5700001	5800000		545	267	518	167
Chr4	5800001	5900000		1013	271	816	521
Chr4	5900001	6000000		926	602	544	413
Chr4	6000001	6100000		1026	561	773	412
Chr4	6100001	6200000		988	528	600	541
Chr4	6200001	6300000		994	582	708	513
Chr4	6300001	6400000		995	511	775	402
Chr4	6400001	6500000		1215	678	984	394
Chr4	6500001	6600000		1030	585	843	376
Chr4	6600001	6700000		460	418	23	16
Chr4	6700001	6800000		310	224	53	18
Chr4	6800001	6900000		283	233	27	5
Chr4	6900001	7000000		1439	401	737	582
Chr4	7000001	7100000		490	57	60	120
Chr4	7100001	7200000		763	258	270	333
Chr4	7200001	7300000		756	358	427	269
Chr4	7300001	7400000		809	365	494	250
Chr4	7400001	7500000		448	183	257	134
Chr4	7500001	7600000		699	169	193	89
Chr4	7600001	7700000		285	24	39	4
Chr4	7700001	7800000		268	15	35	8
Chr4	7800001	7900000		517	87	266	24
Chr4	7900001	8000000		428	84	160	33
Chr4	8000001	8100000		865	93	104	26
Chr4	8100001	8200000		746	73	77	38
Chr4	8200001	8300000		1166	245	280	53
Chr4	8300001	8400000		761	108	166	49
Chr4	8400001	8500000		588	209	234	182
Chr4	8500001	8600000		986	533	611	73
Chr4	8600001	8700000		825	339	447	52
Chr4	8700001	8800000		926	468	594	60
Chr4	8800001	8900000		1145	529	582	63
Chr4	8900001	9000000		677	366	403	55
Chr4	9000001	9100000		919	511	584	64
Chr4	9100001	9200000		27	2	12	6
Chr4	9200001	9300000		836	491	599	51
Chr4	9300001	9400000		975	593	710	65
Chr4	9400001	9500000		891	270	321	84
Chr4	9500001	9600000		425	41	55	18
Chr4	9600001	9700000		1145	624	786	72

					SNP		
				All	Bengal/Nona Bokra	PSSR/Bengal	PSSR/Nona Bokra
Chr4	9700001	9800000		537	159	215	20
Chr4	9800001	9900000		612	179	220	39
Chr4	9900001	10000000		961	552	653	68
Chr4	1E+07	10100000		1218	645	765	85
Chr4	1E+07	10200000		343	30	36	14
Chr4	1E+07	10300000		569	62	50	1
Chr4	1E+07	10400000		1198	655	583	49
Chr4	1E+07	10500000		629	304	347	21
Chr4	1.1E+07	10600000		816	171	197	7
Chr4	1.1E+07	10700000		1331	608	731	86
Chr4	1.1E+07	10800000		1451	804	973	80
Chr4	1.1E+07	10900000		1179	518	567	71
Chr4	1.1E+07	11000000		727	342	448	111
Chr4	1.1E+07	11100000		981	429	573	185
Chr4	1.1E+07	11200000		857	242	524	109
Chr4	1.1E+07	11300000		1002	561	635	228
Chr4	1.1E+07	11400000		599	289	399	107
Chr4	1.1E+07	11500000		887	450	575	213
Chr4	1.2E+07	11600000		1037	645	817	238
Chr4	1.2E+07	11700000		849	366	543	106
Chr4	1.2E+07	11800000		736	411	505	123
Chr4	1.2E+07	11900000		1247	358	442	311
Chr4	1.2E+07	12000000		484	205	310	206
Chr4	1.2E+07	12100000		1209	539	718	364
Chr4	1.2E+07	12200000		1043	465	597	78
Chr4	1.2E+07	12300000		690	393	487	5
Chr4	1.2E+07	12400000		763	610	696	7
Chr4	1.2E+07	12500000		464	296	321	7
Chr4	1.3E+07	12600000		656	464	466	32
Chr4	1.3E+07	12700000		1271	979	981	114
Chr4	1.3E+07	12800000		437	241	281	182
Chr4	1.3E+07	12900000		122	45	57	1
Chr4	1.3E+07	13000000		1164	587	787	438
Chr4	1.3E+07	13100000		535	242	364	168
Chr4	1.3E+07	13200000		961	608	638	400
Chr4	1.3E+07	13300000		924	361	590	415
Chr4	1.3E+07	13400000		1269	621	786	626
Chr4	1.3E+07	13500000		730	442	612	9
Chr4	1.4E+07	13600000		663	439	631	5
Chr4	1.4E+07	13700000		749	557	708	10
Chr4	1.4E+07	13800000		536	354	486	14
Chr4	1.4E+07	13900000		649	474	605	12
Chr4	1.4E+07	14000000		530	354	384	7
Chr4	1.4E+07	14100000		535	267	374	8
Chr4	1.4E+07	14200000		651	236	316	4

					SNP		
				All	Bengal/Nona Bokra	PSSR/Bengal	PSSR/Nona Bokra
Chr4	1.4E+07	14300000		403	39	56	1
Chr4	1.4E+07	14400000		277	22	38	7
Chr4	1.4E+07	14500000		124	8	21	3
Chr4	1.5E+07	14600000		502	207	164	200
Chr4	1.5E+07	14700000		487	283	177	281
Chr4	1.5E+07	14800000		432	403	12	395
Chr4	1.5E+07	14900000		306	255	12	237
Chr4	1.5E+07	15000000		101	51	8	65
Chr4	1.5E+07	15100000		251	168	1	119
Chr4	1.5E+07	15200000		160	117	12	111
Chr4	1.5E+07	15300000		178	112	18	109
Chr4	1.5E+07	15400000		317	275	21	274
Chr4	1.5E+07	15500000		144	93	27	90
Chr4	1.6E+07	15600000		26	6	14	6
Chr4	1.6E+07	15700000		2	2	0	0
Chr4	1.6E+07	15800000		4	2	0	0
Chr4	1.6E+07	15900000		300	242	6	238
Chr4	1.6E+07	16000000		1230	983	27	982
Chr4	1.6E+07	16100000		385	341	18	346
Chr4	1.6E+07	16200000		1091	653	509	617
Chr4	1.6E+07	16300000		1338	1046	1150	175
Chr4	1.6E+07	16400000		749	623	696	14
Chr4	1.6E+07	16500000		528	396	471	9
Chr4	1.7E+07	16600000		686	525	635	21
Chr4	1.7E+07	16700000		981	839	772	4
Chr4	1.7E+07	16800000		160	110	133	8
Chr4	1.7E+07	16900000		35	19	17	11
Chr4	1.7E+07	17000000		44	21	28	11
Chr4	1.7E+07	17100000		32	12	15	6
Chr4	1.7E+07	17200000		45	20	20	12
Chr4	1.7E+07	17300000		46	20	28	8
Chr4	1.7E+07	17400000		61	26	31	15
Chr4	1.7E+07	17500000		325	285	25	277
Chr4	1.8E+07	17600000		275	216	35	217
Chr4	1.8E+07	17700000		798	758	33	759
Chr4	1.8E+07	17800000		965	410	528	497
Chr4	1.8E+07	17900000		867	484	464	435
Chr4	1.8E+07	18000000		897	363	441	533
Chr4	1.8E+07	18100000		1183	378	368	323
Chr4	1.8E+07	18200000		1058	434	419	232
Chr4	1.8E+07	18300000		1492	677	662	178
Chr4	1.8E+07	18400000		777	346	294	82
Chr4	1.8E+07	18500000		1067	640	736	99
Chr4	1.9E+07	18600000		1310	852	759	62
Chr4	1.9E+07	18700000		1323	599	946	751

					SNP		
				All	Bengal/Nona Bokra	PSSR/Bengal	PSSR/Nona Bokra
Chr4	1.9E+07	18800000		684	356	437	370
Chr4	1.9E+07	18900000		784	437	356	240
Chr4	1.9E+07	19000000		689	430	632	114
Chr4	1.9E+07	19100000		615	12	583	506
Chr4	1.9E+07	19200000		436	218	402	114
Chr4	1.9E+07	19300000		851	588	793	5
Chr4	1.9E+07	19400000		678	499	633	11
Chr4	1.9E+07	19500000		705	490	635	4
Chr4	2E+07	19600000		901	681	845	12
Chr4	2E+07	19700000		756	563	716	10
Chr4	2E+07	19800000		565	355	444	40
Chr4	2E+07	19900000		738	473	577	98
Chr4	2E+07	20000000		528	352	390	11
Chr4	2E+07	20100000		680	462	591	151
Chr4	2E+07	20200000		676	480	626	28
Chr4	2E+07	20300000		778	492	616	174
Chr4	2E+07	20400000		705	382	522	93
Chr4	2E+07	20500000		933	465	610	359
Chr4	2.1E+07	20600000		875	461	571	205
Chr4	2.1E+07	20700000		1077	639	803	242
Chr4	2.1E+07	20800000		1103	477	597	397
Chr4	2.1E+07	20900000		962	578	350	427
Chr4	2.1E+07	21000000		706	454	442	212
Chr4	2.1E+07	21100000		925	771	254	633
Chr4	2.1E+07	21200000		245	204	25	207
Chr4	2.1E+07	21300000		698	457	441	369
Chr4	2.1E+07	21400000		503	416	150	355
Chr4	2.1E+07	21500000		634	346	406	308
Chr4	2.2E+07	21600000		671	179	465	518
Chr4	2.2E+07	21700000		565	17	549	456
Chr4	2.2E+07	21800000		564	215	407	396
Chr4	2.2E+07	21900000		761	351	569	446
Chr4	2.2E+07	22000000		584	40	533	469
Chr4	2.2E+07	22100000		680	345	565	273
Chr4	2.2E+07	22200000		482	368	159	362
Chr4	2.2E+07	22300000		318	304	17	303
Chr4	2.2E+07	22400000		28	20	20	6
Chr4	2.2E+07	22500000		282	185	249	13
Chr4	2.3E+07	22600000		272	174	236	5
Chr4	2.3E+07	22700000		239	145	200	11
Chr4	2.3E+07	22800000		56	31	41	2
Chr4	2.3E+07	22900000		85	46	56	10
Chr4	2.3E+07	23000000		15	9	9	4
Chr4	2.3E+07	23100000		23	10	16	8
Chr4	2.3E+07	23200000		25	10	18	20

					SNP		
				All	Bengal/Nona Bokra	PSSR/Bengal	PSSR/Nona Bokra
Chr4	2.3E+07	23300000		22	11	11	5
Chr4	2.3E+07	23400000		103	75	83	5
Chr4	2.3E+07	23500000		627	490	589	8
Chr4	2.4E+07	23600000		697	542	648	4
Chr4	2.4E+07	23700000		555	308	440	208
Chr4	2.4E+07	23800000		768	452	565	345
Chr4	2.4E+07	23900000		830	586	631	192
Chr4	2.4E+07	24000000		630	487	469	63
Chr4	2.4E+07	24100000		670	442	579	164
Chr4	2.4E+07	24200000		724	451	616	191
Chr4	2.4E+07	24300000		641	470	603	7
Chr4	2.4E+07	24400000		432	221	414	151
Chr4	2.4E+07	24500000		560	336	508	129
Chr4	2.5E+07	24600000		575	368	426	277
Chr4	2.5E+07	24700000		706	466	585	220
Chr4	2.5E+07	24800000		726	436	577	208
Chr4	2.5E+07	24900000		573	349	454	229
Chr4	2.5E+07	25000000		1025	707	654	374
Chr4	2.5E+07	25100000		663	407	548	217
Chr4	2.5E+07	25200000		652	419	510	242
Chr4	2.5E+07	25300000		693	466	404	372
Chr4	2.5E+07	25400000		394	298	143	288
Chr4	2.5E+07	25500000		438	416	21	419
Chr4	2.6E+07	25600000		488	438	35	447
Chr4	2.6E+07	25700000		455	430	13	438
Chr4	2.6E+07	25800000		283	253	25	253
Chr4	2.6E+07	25900000		59	20	15	17
Chr4	2.6E+07	26000000		26	10	4	7
Chr4	2.6E+07	26100000		15	5	4	5
Chr4	2.6E+07	26200000		46	6	2	5
Chr4	2.6E+07	26300000		142	13	10	9
Chr4	2.6E+07	26400000		119	10	11	10
Chr4	2.6E+07	26500000		31	11	12	4
Chr4	2.7E+07	26600000		24	9	10	3
Chr4	2.7E+07	26700000		43	22	22	7
Chr4	2.7E+07	26800000		26	15	17	7
Chr4	2.7E+07	26900000		38	13	19	7
Chr4	2.7E+07	27000000		39	10	24	8
Chr4	2.7E+07	27100000		26	19	21	5
Chr4	2.7E+07	27200000		461	331	417	6
Chr4	2.7E+07	27300000		562	453	533	7
Chr4	2.7E+07	27400000		614	444	574	2
Chr4	2.7E+07	27500000		578	424	525	2
Chr4	2.8E+07	27600000		583	425	558	4
Chr4	2.8E+07	27700000		607	464	579	5

					SNP		
				All	Bengal/Nona Bokra	PSSR/Bengal	PSSR/Nona Bokra
Chr4	2.8E+07	27800000		629	479	599	5
Chr4	2.8E+07	27900000		696	517	654	9
Chr4	2.8E+07	28000000		580	418	506	71
Chr4	2.8E+07	28100000		667	359	481	355
Chr4	2.8E+07	28200000		689	412	545	219
Chr4	2.8E+07	28300000		638	385	488	236
Chr4	2.8E+07	28400000		847	506	645	365
Chr4	2.8E+07	28500000		850	556	675	311
Chr4	2.9E+07	28600000		568	487	348	191
Chr4	2.9E+07	28700000		383	361	15	358
Chr4	2.9E+07	28800000		488	459	18	460
Chr4	2.9E+07	28900000		481	444	13	410
Chr4	2.9E+07	29000000		392	286	56	276
Chr4	2.9E+07	29100000		539	410	123	415
Chr4	2.9E+07	29200000		579	442	147	470
Chr4	2.9E+07	29300000		521	494	17	496
Chr4	2.9E+07	29400000		841	659	194	670
Chr4	2.9E+07	29500000		483	303	362	140
Chr4	3E+07	29600000		492	382	467	2
Chr4	3E+07	29700000		540	414	478	12
Chr4	3E+07	29800000		574	358	448	183
Chr4	3E+07	29900000		313	261	77	260
Chr4	3E+07	30000000		339	207	269	102
Chr4	3E+07	30100000		306	132	188	129
Chr4	3E+07	30200000		666	141	535	461
Chr4	3E+07	30300000		457	18	431	403
Chr4	3E+07	30400000		407	10	298	252
Chr4	3E+07	30500000		444	4	386	345
Chr4	3.1E+07	30600000		326	15	293	251
Chr4	3.1E+07	30700000		359	29	285	222
Chr4	3.1E+07	30800000		553	198	437	178
Chr4	3.1E+07	30900000		615	241	520	211
Chr4	3.1E+07	31000000		638	121	571	510
Chr4	3.1E+07	31100000		685	461	640	104
Chr4	3.1E+07	31200000		644	419	483	184
Chr4	3.1E+07	31300000		602	260	460	194
Chr4	3.1E+07	31400000		1159	563	663	392
Chr4	3.1E+07	31500000		888	479	661	352
Chr4	3.2E+07	31600000		555	297	387	228
Chr4	3.2E+07	31700000		623	354	340	256
Chr4	3.2E+07	31800000		608	286	457	358
Chr4	3.2E+07	31900000		594	338	431	247
Chr4	3.2E+07	32000000		480	226	335	250
Chr4	3.2E+07	32100000		623	376	458	52
Chr4	3.2E+07	32200000		588	288	313	5

					SNP		
				All	Bengal/Nona Bokra	PSSR/Bengal	PSSR/Nona Bokra
Chr4	3.2E+07	32300000		574	347	422	7
Chr4	3.2E+07	32400000		387	252	291	3
Chr4	3.2E+07	32500000		546	350	403	2
Chr4	3.3E+07	32600000		513	359	418	4
Chr4	3.3E+07	32700000		467	363	433	3
Chr4	3.3E+07	32800000		576	377	458	10
Chr4	3.3E+07	32900000		454	297	373	8
Chr4	3.3E+07	33000000		434	315	374	9
Chr4	3.3E+07	33100000		480	362	448	2
Chr4	3.3E+07	33200000		546	425	267	306
Chr4	3.3E+07	33300000		113	82	76	32
Chr4	3.3E+07	33400000		519	371	326	234
Chr4	3.3E+07	33500000		571	311	338	407
Chr4	3.4E+07	33600000		573	235	356	45
Chr4	3.4E+07	33700000		510	6	323	287
Chr4	3.4E+07	33800000		501	10	1	5
Chr4	3.4E+07	33900000		482	233	6	233
Chr4	3.4E+07	34000000		128	7	6	5
Chr4	3.4E+07	34100000		258	9	9	6
Chr4	3.4E+07	34200000		77	20	29	6
Chr4	3.4E+07	34300000		286	214	256	5
Chr4	3.4E+07	34400000		759	631	721	1
Chr4	3.4E+07	34500000		484	379	459	3
Chr4	3.5E+07	34600000		660	514	621	4
Chr4	3.5E+07	34700000		448	359	444	3
Chr4	3.5E+07	34800000		520	379	500	4
Chr4	3.5E+07	34900000		556	454	476	2
Chr4	3.5E+07	35000000		557	435	532	3
Chr4	3.5E+07	35100000		662	530	639	4
Chr4	3.5E+07	35200000		523	338	452	75
Chr4	3.5E+07	35300000		430	338	397	37
Chr4	3.5E+07	35400000		440	370	427	1
Chr4	3.5E+07	35500000		491	378	444	7
Chr4	3.6E+07	35600000		18	7	15	2
Chr5	Chr5						
Chr5	1	100000		499	336	400	118
Chr5	100001	200000		700	410	518	264
Chr5	200001	300000		373	336	32	338
Chr5	300001	400000		474	352	316	133
Chr5	400001	500000		590	417	499	126
Chr5	500001	600000		669	443	575	73
Chr5	600001	700000		553	403	331	247
Chr5	700001	800000		990	691	621	417
Chr5	800001	900000		1028	483	618	503
Chr5	900001	1000000		775	445	529	361

					SNP		
				All	Bengal/Nona Bokra	PSSR/Bengal	PSSR/Nona Bokra
Chr5	1000001	1100000		619	448	548	72
Chr5	1100001	1200000		719	464	569	267
Chr5	1200001	1300000		678	364	448	205
Chr5	1300001	1400000		334	168	174	138
Chr5	1400001	1500000		619	395	396	273
Chr5	1500001	1600000		688	401	475	247
Chr5	1600001	1700000		677	422	537	184
Chr5	1700001	1800000		1083	581	577	349
Chr5	1800001	1900000		1127	293	337	386
Chr5	1900001	2000000		1254	752	770	331
Chr5	2000001	2100000		993	405	688	650
Chr5	2100001	2200000		1188	573	739	858
Chr5	2200001	2300000		610	436	208	435
Chr5	2300001	2400000		1138	633	449	648
Chr5	2400001	2500000		626	350	449	24
Chr5	2500001	2600000		663	340	436	8
Chr5	2600001	2700000		640	334	384	8
Chr5	2700001	2800000		683	462	542	6
Chr5	2800001	2900000		452	336	392	6
Chr5	2900001	3000000		500	354	419	6
Chr5	3000001	3100000		692	420	497	112
Chr5	3100001	3200000		814	446	569	436
Chr5	3200001	3300000		454	336	425	6
Chr5	3300001	3400000		618	509	524	7
Chr5	3400001	3500000		235	161	198	7
Chr5	3500001	3600000		770	259	549	529
Chr5	3600001	3700000		624	402	309	433
Chr5	3700001	3800000		334	153	313	134
Chr5	3800001	3900000		308	5	302	258
Chr5	3900001	4000000		185	2	172	153
Chr5	4000001	4100000		333	10	323	282
Chr5	4100001	4200000		504	10	498	414
Chr5	4200001	4300000		473	115	395	376
Chr5	4300001	4400000		567	311	496	134
Chr5	4400001	4500000		308	206	258	10
Chr5	4500001	4600000		521	396	480	9
Chr5	4600001	4700000		750	489	646	5
Chr5	4700001	4800000		792	597	561	198
Chr5	4800001	4900000		39	24	27	10
Chr5	4900001	5000000		24	12	17	3
Chr5	5000001	5100000		28	14	16	2
Chr5	5100001	5200000		425	328	403	8
Chr5	5200001	5300000		408	349	393	6
Chr5	5300001	5400000		970	813	914	11
Chr5	5400001	5500000		941	627	740	116

					SNP		
				All	Bengal/Nona Bokra	PSSR/Bengal	PSSR/Nona Bokra
Chr5	5500001	5600000		1023	460	556	10
Chr5	5600001	5700000		946	76	97	6
Chr5	5700001	5800000		757	265	299	6
Chr5	5800001	5900000		1125	546	692	226
Chr5	5900001	6000000		863	502	331	520
Chr5	6000001	6100000		1003	652	307	596
Chr5	6100001	6200000		1005	612	436	617
Chr5	6200001	6300000		914	537	360	546
Chr5	6300001	6400000		1292	733	667	729
Chr5	6400001	6500000		953	14	909	795
Chr5	6500001	6600000		944	604	392	590
Chr5	6600001	6700000		1034	666	358	670
Chr5	6700001	6800000		854	576	498	251
Chr5	6800001	6900000		1130	966	114	883
Chr5	6900001	7000000		683	602	19	600
Chr5	7000001	7100000		284	116	20	104
Chr5	7100001	7200000		371	22	19	9
Chr5	7200001	7300000		587	138	16	135
Chr5	7300001	7400000		680	198	480	468
Chr5	7400001	7500000		808	22	706	596
Chr5	7500001	7600000		604	13	571	486
Chr5	7600001	7700000		514	14	488	445
Chr5	7700001	7800000		663	10	648	545
Chr5	7800001	7900000		918	15	852	756
Chr5	7900001	8000000		795	7	758	659
Chr5	8000001	8100000		590	11	565	507
Chr5	8100001	8200000		726	244	578	448
Chr5	8200001	8300000		855	381	648	458
Chr5	8300001	8400000		1035	540	680	644
Chr5	8400001	8500000		1019	585	617	610
Chr5	8500001	8600000		631	391	351	358
Chr5	8600001	8700000		948	514	486	520
Chr5	8700001	8800000		1016	499	665	546
Chr5	8800001	8900000		502	169	391	275
Chr5	8900001	9000000		905	343	733	540
Chr5	9000001	9100000		736	193	619	530
Chr5	9100001	9200000		545	103	490	407
Chr5	9200001	9300000		474	72	421	362
Chr5	9300001	9400000		1026	272	837	720
Chr5	9400001	9500000		474	223	335	223
Chr5	9500001	9600000		562	229	450	280
Chr5	9600001	9700000		577	299	408	264
Chr5	9700001	9800000		880	532	681	363
Chr5	9800001	9900000		482	271	326	150
Chr5	9900001	10000000		239	112	182	65

					SNP		
				All	Bengal/Nona Bokra	PSSR/Bengal	PSSR/Nona Bokra
Chr5	1E+07	10100000		245	102	187	81
Chr5	1E+07	10200000		433	171	313	198
Chr5	1E+07	10300000		508	214	337	261
Chr5	1E+07	10400000		680	237	529	432
Chr5	1E+07	10500000		745	274	524	487
Chr5	1.1E+07	10600000		448	171	345	249
Chr5	1.1E+07	10700000		297	94	234	181
Chr5	1.1E+07	10800000		455	218	340	218
Chr5	1.1E+07	10900000		574	330	433	189
Chr5	1.1E+07	11000000		643	452	467	225
Chr5	1.1E+07	11100000		874	538	561	449
Chr5	1.1E+07	11200000		372	215	222	229
Chr5	1.1E+07	11300000		371	205	274	155
Chr5	1.1E+07	11400000		607	352	436	239
Chr5	1.1E+07	11500000		950	514	657	536
Chr5	1.2E+07	11600000		143	67	100	48
Chr5	1.2E+07	11700000		676	376	449	369
Chr5	1.2E+07	11800000		1013	514	687	558
Chr5	1.2E+07	11900000		447	157	344	143
Chr5	1.2E+07	12000000		680	263	516	270
Chr5	1.2E+07	12100000		709	404	501	381
Chr5	1.2E+07	12200000		783	470	540	414
Chr5	1.2E+07	12300000		924	611	464	333
Chr5	1.2E+07	12400000		918	521	635	448
Chr5	1.2E+07	12500000		418	245	242	218
Chr5	1.3E+07	12600000		357	138	237	146
Chr5	1.3E+07	12700000		1043	577	743	593
Chr5	1.3E+07	12800000		652	323	463	334
Chr5	1.3E+07	12900000		843	503	530	340
Chr5	1.3E+07	13000000		604	303	458	258
Chr5	1.3E+07	13100000		653	406	462	336
Chr5	1.3E+07	13200000		368	200	260	164
Chr5	1.3E+07	13300000		603	326	437	275
Chr5	1.3E+07	13400000		608	369	473	236
Chr5	1.3E+07	13500000		789	447	549	442
Chr5	1.4E+07	13600000		760	408	512	418
Chr5	1.4E+07	13700000		681	433	531	233
Chr5	1.4E+07	13800000		776	429	571	384
Chr5	1.4E+07	13900000		862	555	685	314
Chr5	1.4E+07	14000000		720	380	582	267
Chr5	1.4E+07	14100000		946	618	398	333
Chr5	1.4E+07	14200000		777	530	319	339
Chr5	1.4E+07	14300000		793	464	498	441
Chr5	1.4E+07	14400000		848	463	572	429
Chr5	1.4E+07	14500000		751	471	642	172

					SNP		
				All	Bengal/Nona Bokra	PSSR/Bengal	PSSR/Nona Bokra
Chr5	1.5E+07	14600000		728	409	606	127
Chr5	1.5E+07	14700000		969	560	708	448
Chr5	1.5E+07	14800000		920	503	609	505
Chr5	1.5E+07	14900000		751	443	598	279
Chr5	1.5E+07	15000000		839	496	654	296
Chr5	1.5E+07	15100000		799	466	581	338
Chr5	1.5E+07	15200000		712	417	490	290
Chr5	1.5E+07	15300000		812	466	481	341
Chr5	1.5E+07	15400000		146	71	93	54
Chr5	1.5E+07	15500000		288	163	191	139
Chr5	1.6E+07	15600000		988	598	741	360
Chr5	1.6E+07	15700000		539	323	466	101
Chr5	1.6E+07	15800000		591	403	492	145
Chr5	1.6E+07	15900000		563	368	492	84
Chr5	1.6E+07	16000000		932	571	763	284
Chr5	1.6E+07	16100000		650	373	507	180
Chr5	1.6E+07	16200000		777	534	580	245
Chr5	1.6E+07	16300000		889	560	736	267
Chr5	1.6E+07	16400000		778	471	618	248
Chr5	1.6E+07	16500000		400	263	294	103
Chr5	1.7E+07	16600000		803	108	689	536
Chr5	1.7E+07	16700000		767	441	599	272
Chr5	1.7E+07	16800000		1056	671	822	405
Chr5	1.7E+07	16900000		791	366	676	345
Chr5	1.7E+07	17000000		441	5	426	377
Chr5	1.7E+07	17100000		780	20	754	670
Chr5	1.7E+07	17200000		893	424	875	284
Chr5	1.7E+07	17300000		771	425	536	5
Chr5	1.7E+07	17400000		741	537	704	5
Chr5	1.7E+07	17500000		929	587	783	12
Chr5	1.8E+07	17600000		852	672	665	1
Chr5	1.8E+07	17700000		808	561	731	12
Chr5	1.8E+07	17800000		534	378	367	8
Chr5	1.8E+07	17900000		831	511	679	4
Chr5	1.8E+07	18000000		744	556	697	6
Chr5	1.8E+07	18100000		632	458	587	4
Chr5	1.8E+07	18200000		218	167	206	6
Chr5	1.8E+07	18300000		607	431	586	15
Chr5	1.8E+07	18400000		521	394	368	1
Chr5	1.8E+07	18500000		906	398	806	281
Chr5	1.9E+07	18600000		1083	688	864	339
Chr5	1.9E+07	18700000		1085	758	944	191
Chr5	1.9E+07	18800000		912	565	610	424
Chr5	1.9E+07	18900000		825	517	652	254
Chr5	1.9E+07	19000000		1052	640	822	397

					SNP		
				All	Bengal/Nona Bokra	PSSR/Bengal	PSSR/Nona Bokra
Chr5	1.9E+07	19100000		635	388	487	288
Chr5	1.9E+07	19200000		845	431	636	445
Chr5	1.9E+07	19300000		880	525	705	345
Chr5	1.9E+07	19400000		873	529	726	234
Chr5	1.9E+07	19500000		1066	694	784	196
Chr5	2E+07	19600000		842	306	579	449
Chr5	2E+07	19700000		864	417	633	427
Chr5	2E+07	19800000		406	287	111	313
Chr5	2E+07	19900000		600	270	484	282
Chr5	2E+07	20000000		705	539	667	11
Chr5	2E+07	20100000		850	555	784	12
Chr5	2E+07	20200000		735	551	684	6
Chr5	2E+07	20300000		716	489	671	4
Chr5	2E+07	20400000		805	564	742	58
Chr5	2E+07	20500000		774	349	743	317
Chr5	2.1E+07	20600000		478	338	417	8
Chr5	2.1E+07	20700000		934	566	692	11
Chr5	2.1E+07	20800000		846	636	761	6
Chr5	2.1E+07	20900000		690	509	649	7
Chr5	2.1E+07	21000000		625	456	573	8
Chr5	2.1E+07	21100000		572	451	548	4
Chr5	2.1E+07	21200000		663	437	625	6
Chr5	2.1E+07	21300000		671	512	618	8
Chr5	2.1E+07	21400000		579	14	547	485
Chr5	2.1E+07	21500000		572	218	502	297
Chr5	2.2E+07	21600000		624	383	517	126
Chr5	2.2E+07	21700000		945	613	770	272
Chr5	2.2E+07	21800000		869	537	718	251
Chr5	2.2E+07	21900000		447	297	385	53
Chr5	2.2E+07	22000000		600	388	490	152
Chr5	2.2E+07	22100000		795	484	649	159
Chr5	2.2E+07	22200000		697	433	577	144
Chr5	2.2E+07	22300000		910	609	817	147
Chr5	2.2E+07	22400000		653	477	609	3
Chr5	2.2E+07	22500000		781	559	671	121
Chr5	2.3E+07	22600000		571	395	484	93
Chr5	2.3E+07	22700000		669	479	632	7
Chr5	2.3E+07	22800000		601	478	570	6
Chr5	2.3E+07	22900000		675	473	599	3
Chr5	2.3E+07	23000000		590	431	553	12
Chr5	2.3E+07	23100000		661	504	627	1
Chr5	2.3E+07	23200000		529	369	474	2
Chr5	2.3E+07	23300000		553	376	503	7
Chr5	2.3E+07	23400000		615	480	587	3
Chr5	2.3E+07	23500000		848	655	787	13

					SNP		
				All	Bengal/Nona Bokra	PSSR/Bengal	PSSR/Nona Bokra
Chr5	2.4E+07	23600000		633	448	354	209
Chr5	2.4E+07	23700000		69	44	49	5
Chr5	2.4E+07	23800000		178	110	153	13
Chr5	2.4E+07	23900000		308	196	249	7
Chr5	2.4E+07	24000000		817	641	768	12
Chr5	2.4E+07	24100000		509	389	476	6
Chr5	2.4E+07	24200000		770	436	755	219
Chr5	2.4E+07	24300000		539	102	527	360
Chr5	2.4E+07	24400000		451	344	427	7
Chr5	2.4E+07	24500000		578	440	547	3
Chr5	2.5E+07	24600000		570	416	546	6
Chr5	2.5E+07	24700000		630	501	611	9
Chr5	2.5E+07	24800000		699	519	659	4
Chr5	2.5E+07	24900000		843	599	766	9
Chr5	2.5E+07	25000000		574	395	540	8
Chr5	2.5E+07	25100000		828	659	770	5
Chr5	2.5E+07	25200000		684	511	648	2
Chr5	2.5E+07	25300000		444	359	425	7
Chr5	2.5E+07	25400000		502	423	495	2
Chr5	2.5E+07	25500000		639	497	603	12
Chr5	2.6E+07	25600000		851	613	817	10
Chr5	2.6E+07	25700000		638	495	604	4
Chr5	2.6E+07	25800000		572	408	542	5
Chr5	2.6E+07	25900000		391	288	375	2
Chr5	2.6E+07	26000000		439	325	413	1
Chr5	2.6E+07	26100000		483	318	424	4
Chr5	2.6E+07	26200000		742	610	663	7
Chr5	2.6E+07	26300000		799	601	693	44
Chr5	2.6E+07	26400000		680	532	602	8
Chr5	2.6E+07	26500000		391	301	359	5
Chr5	2.7E+07	26600000		511	419	496	6
Chr5	2.7E+07	26700000		438	372	428	2
Chr5	2.7E+07	26800000		715	511	640	9
Chr5	2.7E+07	26900000		464	355	451	4
Chr5	2.7E+07	27000000		640	453	606	23
Chr5	2.7E+07	27100000		834	537	619	269
Chr5	2.7E+07	27200000		326	220	227	7
Chr5	2.7E+07	27300000		557	432	496	58
Chr5	2.7E+07	27400000		669	378	503	230
Chr5	2.7E+07	27500000		719	581	647	12
Chr5	2.8E+07	27600000		486	370	441	45
Chr5	2.8E+07	27700000		507	383	471	15
Chr5	2.8E+07	27800000		434	344	398	6
Chr5	2.8E+07	27900000		542	431	514	21
Chr5	2.8E+07	28000000		827	574	694	220

					SNP		
				All	Bengal/Nona Bokra	PSSR/Bengal	PSSR/Nona Bokra
Chr5	2.8E+07	28100000		723	531	624	132
Chr5	2.8E+07	28200000		584	420	542	5
Chr5	2.8E+07	28300000		574	424	539	55
Chr5	2.8E+07	28400000		560	429	517	61
Chr5	2.8E+07	28500000		544	367	450	163
Chr5	2.9E+07	28600000		879	515	683	369
Chr5	2.9E+07	28700000		516	373	439	151
Chr5	2.9E+07	28800000		662	403	535	222
Chr5	2.9E+07	28900000		582	420	516	93
Chr5	2.9E+07	29000000		493	383	444	60
Chr5	2.9E+07	29100000		715	519	626	136
Chr5	2.9E+07	29200000		762	471	615	232
Chr5	2.9E+07	29300000		465	341	401	73
Chr5	2.9E+07	29400000		451	345	426	5
Chr5	2.9E+07	29500000		691	486	632	7
Chr5	3E+07	29600000		586	347	499	183
Chr5	3E+07	29700000		137	10	130	105
Chr5	3E+07	29800000		26	9	9	10
Chr5	3E+07	29900000		92	33	53	29
Chr5	3E+07	30000000		39	13	15	5
Chr6	Chr6						
Chr6	1	100000		240	107	120	20
Chr6	100001	200000		653	529	569	10
Chr6	200001	300000		459	380	416	61
Chr6	300001	400000		440	316	268	116
Chr6	400001	500000		732	454	590	239
Chr6	500001	600000		561	378	518	65
Chr6	600001	700000		611	422	521	128
Chr6	700001	800000		815	554	558	374
Chr6	800001	900000		845	479	626	437
Chr6	900001	1000000		726	271	528	246
Chr6	1000001	1100000		591	212	400	137
Chr6	1100001	1200000		683	380	397	379
Chr6	1200001	1300000		563	255	421	252
Chr6	1300001	1400000		465	320	337	159
Chr6	1400001	1500000		663	342	489	386
Chr6	1500001	1600000		494	296	377	224
Chr6	1600001	1700000		707	448	576	250
Chr6	1700001	1800000		650	430	561	125
Chr6	1800001	1900000		575	482	453	65
Chr6	1900001	2000000		478	384	378	50
Chr6	2000001	2100000		273	208	261	0
Chr6	2100001	2200000		661	540	646	1
Chr6	2200001	2300000		469	365	459	5
Chr6	2300001	2400000		715	570	685	8

					SNP		
				All	Bengal/Nona Bokra	PSSR/Bengal	PSSR/Nona Bokra
Chr6	2400001	2500000		389	310	320	7
Chr6	2500001	2600000		525	401	497	6
Chr6	2600001	2700000		418	319	391	2
Chr6	2700001	2800000		472	367	408	7
Chr6	2800001	2900000		659	535	616	5
Chr6	2900001	3000000		535	420	518	7
Chr6	3000001	3100000		822	691	627	1
Chr6	3100001	3200000		225	180	135	99
Chr6	3200001	3300000		260	129	243	81
Chr6	3300001	3400000		400	327	303	4
Chr6	3400001	3500000		688	316	362	364
Chr6	3500001	3600000		602	206	291	200
Chr6	3600001	3700000		198	165	189	5
Chr6	3700001	3800000		18	8	6	5
Chr6	3800001	3900000		8	3	3	1
Chr6	3900001	4000000		23	12	15	3
Chr6	4000001	4100000		230	181	224	1
Chr6	4100001	4200000		558	421	236	390
Chr6	4200001	4300000		1002	795	746	141
Chr6	4300001	4400000		1348	1111	1068	24
Chr6	4400001	4500000		472	313	383	55
Chr6	4500001	4600000		622	266	493	252
Chr6	4600001	4700000		788	475	591	299
Chr6	4700001	4800000		837	437	657	379
Chr6	4800001	4900000		644	494	620	10
Chr6	4900001	5000000		383	290	366	3
Chr6	5000001	5100000		504	396	428	3
Chr6	5100001	5200000		789	458	447	5
Chr6	5200001	5300000		125	89	100	9
Chr6	5300001	5400000		45	27	32	3
Chr6	5400001	5500000		29	19	21	7
Chr6	5500001	5600000		237	208	93	134
Chr6	5600001	5700000		905	680	734	149
Chr6	5700001	5800000		689	391	523	265
Chr6	5800001	5900000		604	367	468	163
Chr6	5900001	6000000		773	616	365	392
Chr6	6000001	6100000		510	481	20	487
Chr6	6100001	6200000		468	442	28	444
Chr6	6200001	6300000		158	145	29	130
Chr6	6300001	6400000		34	18	23	12
Chr6	6400001	6500000		27	10	6	6
Chr6	6500001	6600000		33	25	21	6
Chr6	6600001	6700000		282	18	267	202
Chr6	6700001	6800000		539	14	505	413
Chr6	6800001	6900000		788	206	660	559

					SNP		
				All	Bengal/Nona Bokra	PSSR/Bengal	PSSR/Nona Bokra
Chr6	6900001	7000000		796	340	604	470
Chr6	7000001	7100000		1089	496	697	627
Chr6	7100001	7200000		1062	595	573	726
Chr6	7200001	7300000		1094	519	671	604
Chr6	7300001	7400000		1342	881	529	495
Chr6	7400001	7500000		1187	738	714	649
Chr6	7500001	7600000		774	95	713	640
Chr6	7600001	7700000		722	100	651	522
Chr6	7700001	7800000		852	327	721	473
Chr6	7800001	7900000		561	415	450	4
Chr6	7900001	8000000		726	563	346	419
Chr6	8000001	8100000		1247	859	830	388
Chr6	8100001	8200000		1106	506	581	524
Chr6	8200001	8300000		680	348	276	337
Chr6	8300001	8400000		916	479	497	505
Chr6	8400001	8500000		838	273	588	259
Chr6	8500001	8600000		755	51	577	52
Chr6	8600001	8700000		974	441	433	410
Chr6	8700001	8800000		830	444	418	485
Chr6	8800001	8900000		845	234	294	292
Chr6	8900001	9000000		1380	561	891	509
Chr6	9000001	9100000		1042	364	299	293
Chr6	9100001	9200000		898	215	449	448
Chr6	9200001	9300000		1306	351	959	753
Chr6	9300001	9400000		1019	677	803	48
Chr6	9400001	9500000		802	356	428	4
Chr6	9500001	9600000		1109	916	983	10
Chr6	9600001	9700000		1035	542	573	480
Chr6	9700001	9800000		216	68	31	67
Chr6	9800001	9900000		1036	451	319	496
Chr6	9900001	10000000		955	424	493	352
Chr6	1E+07	10100000		1160	566	496	538
Chr6	1E+07	10200000		1046	327	675	484
Chr6	1E+07	10300000		487	182	307	256
Chr6	1E+07	10400000		834	403	446	288
Chr6	1E+07	10500000		1114	396	147	380
Chr6	1.1E+07	10600000		848	289	7	267
Chr6	1.1E+07	10700000		748	390	14	385
Chr6	1.1E+07	10800000		717	293	402	417
Chr6	1.1E+07	10900000		690	251	432	382
Chr6	1.1E+07	11000000		423	257	186	227
Chr6	1.1E+07	11100000		856	536	661	196
Chr6	1.1E+07	11200000		1260	718	874	145
Chr6	1.1E+07	11300000		691	434	573	8
Chr6	1.1E+07	11400000		784	562	718	12

					SNP		
				All	Bengal/Nona Bokra	PSSR/Bengal	PSSR/Nona Bokra
Chr6	1.1E+07	11500000		664	476	480	197
Chr6	1.2E+07	11600000		881	631	304	495
Chr6	1.2E+07	11700000		659	294	361	142
Chr6	1.2E+07	11800000		106	52	74	28
Chr6	1.2E+07	11900000		506	252	375	207
Chr6	1.2E+07	12000000		798	297	631	412
Chr6	1.2E+07	12100000		855	594	748	52
Chr6	1.2E+07	12200000		685	508	591	1
Chr6	1.2E+07	12300000		803	593	758	12
Chr6	1.2E+07	12400000		584	424	537	5
Chr6	1.2E+07	12500000		517	395	496	4
Chr6	1.3E+07	12600000		491	312	401	5
Chr6	1.3E+07	12700000		712	543	588	6
Chr6	1.3E+07	12800000		704	481	666	13
Chr6	1.3E+07	12900000		1014	747	968	9
Chr6	1.3E+07	13000000		981	731	905	7
Chr6	1.3E+07	13100000		855	602	803	13
Chr6	1.3E+07	13200000		778	494	657	196
Chr6	1.3E+07	13300000		983	596	768	352
Chr6	1.3E+07	13400000		819	565	776	40
Chr6	1.3E+07	13500000		951	756	866	9
Chr6	1.4E+07	13600000		836	586	747	10
Chr6	1.4E+07	13700000		845	655	791	11
Chr6	1.4E+07	13800000		729	515	653	8
Chr6	1.4E+07	13900000		670	469	605	18
Chr6	1.4E+07	14000000		948	638	811	154
Chr6	1.4E+07	14100000		1118	730	997	137
Chr6	1.4E+07	14200000		929	634	784	170
Chr6	1.4E+07	14300000		1064	683	940	191
Chr6	1.4E+07	14400000		891	563	730	200
Chr6	1.4E+07	14500000		1013	512	853	211
Chr6	1.5E+07	14600000		864	546	693	193
Chr6	1.5E+07	14700000		503	304	364	77
Chr6	1.5E+07	14800000		956	696	518	125
Chr6	1.5E+07	14900000		1127	712	946	219
Chr6	1.5E+07	15000000		745	387	520	168
Chr6	1.5E+07	15100000		994	528	799	181
Chr6	1.5E+07	15200000		747	377	600	150
Chr6	1.5E+07	15300000		1163	355	1028	134
Chr6	1.5E+07	15400000		1000	668	815	209
Chr6	1.5E+07	15500000		353	236	268	70
Chr6	1.6E+07	15600000		1195	797	1014	247
Chr6	1.6E+07	15700000		1141	776	954	272
Chr6	1.6E+07	15800000		933	611	764	302
Chr6	1.6E+07	15900000		999	620	839	234

					SNP		
				All	Bengal/Nona Bokra	PSSR/Bengal	PSSR/Nona Bokra
Chr6	1.6E+07	16000000		803	495	690	160
Chr6	1.6E+07	16100000		991	625	797	235
Chr6	1.6E+07	16200000		1200	397	1033	182
Chr6	1.6E+07	16300000		1067	487	821	513
Chr6	1.6E+07	16400000		1071	534	773	598
Chr6	1.6E+07	16500000		1439	818	841	912
Chr6	1.7E+07	16600000		1281	653	785	798
Chr6	1.7E+07	16700000		810	417	607	380
Chr6	1.7E+07	16800000		714	363	551	269
Chr6	1.7E+07	16900000		861	453	676	312
Chr6	1.7E+07	17000000		997	433	752	486
Chr6	1.7E+07	17100000		839	545	502	194
Chr6	1.7E+07	17200000		956	560	716	362
Chr6	1.7E+07	17300000		1155	767	918	326
Chr6	1.7E+07	17400000		1009	661	881	155
Chr6	1.7E+07	17500000		956	497	592	413
Chr6	1.8E+07	17600000		825	563	691	210
Chr6	1.8E+07	17700000		646	463	564	9
Chr6	1.8E+07	17800000		977	651	857	168
Chr6	1.8E+07	17900000		803	470	671	176
Chr6	1.8E+07	18000000		651	504	633	14
Chr6	1.8E+07	18100000		349	264	328	9
Chr6	1.8E+07	18200000		686	464	624	16
Chr6	1.8E+07	18300000		210	118	179	8
Chr6	1.8E+07	18400000		58	14	37	5
Chr6	1.8E+07	18500000		56	14	33	0
Chr6	1.9E+07	18600000		217	101	172	2
Chr6	1.9E+07	18700000		46	11	27	1
Chr6	1.9E+07	18800000		197	106	172	1
Chr6	1.9E+07	18900000		627	438	536	19
Chr6	1.9E+07	19000000		478	356	432	12
Chr6	1.9E+07	19100000		267	179	224	4
Chr6	1.9E+07	19200000		356	255	330	8
Chr6	1.9E+07	19300000		329	224	291	7
Chr6	1.9E+07	19400000		731	518	693	2
Chr6	1.9E+07	19500000		732	536	669	20
Chr6	2E+07	19600000		609	409	527	150
Chr6	2E+07	19700000		815	504	691	210
Chr6	2E+07	19800000		727	518	569	148
Chr6	2E+07	19900000		601	339	522	132
Chr6	2E+07	20000000		1157	871	1017	8
Chr6	2E+07	20100000		688	469	648	28
Chr6	2E+07	20200000		772	578	733	5
Chr6	2E+07	20300000		511	382	411	8
Chr6	2E+07	20400000		316	171	249	44

					SNP		
				All	Bengal/Nona Bokra	PSSR/Bengal	PSSR/Nona Bokra
Chr6	2E+07	20500000		867	642	844	13
Chr6	2.1E+07	20600000		813	513	705	134
Chr6	2.1E+07	20700000		625	346	456	251
Chr6	2.1E+07	20800000		1110	636	764	566
Chr6	2.1E+07	20900000		824	510	637	317
Chr6	2.1E+07	21000000		984	596	609	444
Chr6	2.1E+07	21100000		988	654	834	276
Chr6	2.1E+07	21200000		584	433	542	5
Chr6	2.1E+07	21300000		1023	655	785	343
Chr6	2.1E+07	21400000		1110	571	745	323
Chr6	2.1E+07	21500000		624	275	472	344
Chr6	2.2E+07	21600000		984	351	745	641
Chr6	2.2E+07	21700000		692	68	631	537
Chr6	2.2E+07	21800000		102	6	77	72
Chr6	2.2E+07	21900000		18	9	8	8
Chr6	2.2E+07	22000000		106	8	98	73
Chr6	2.2E+07	22100000		859	13	839	623
Chr6	2.2E+07	22200000		820	406	782	251
Chr6	2.2E+07	22300000		560	360	464	179
Chr6	2.2E+07	22400000		811	389	560	524
Chr6	2.2E+07	22500000		652	286	396	306
Chr6	2.3E+07	22600000		556	170	233	162
Chr6	2.3E+07	22700000		972	317	336	235
Chr6	2.3E+07	22800000		436	126	86	101
Chr6	2.3E+07	22900000		1193	393	472	175
Chr6	2.3E+07	23000000		564	50	39	46
Chr6	2.3E+07	23100000		836	415	471	215
Chr6	2.3E+07	23200000		1168	720	823	344
Chr6	2.3E+07	23300000		802	401	520	228
Chr6	2.3E+07	23400000		1134	508	670	390
Chr6	2.3E+07	23500000		1223	570	671	10
Chr6	2.4E+07	23600000		583	190	254	31
Chr6	2.4E+07	23700000		776	340	375	44
Chr6	2.4E+07	23800000		662	389	508	279
Chr6	2.4E+07	23900000		742	461	579	280
Chr6	2.4E+07	24000000		604	438	561	5
Chr6	2.4E+07	24100000		469	433	120	341
Chr6	2.4E+07	24200000		256	240	60	198
Chr6	2.4E+07	24300000		456	426	71	381
Chr6	2.4E+07	24400000		572	517	89	506
Chr6	2.4E+07	24500000		591	428	435	159
Chr6	2.5E+07	24600000		553	420	540	5
Chr6	2.5E+07	24700000		898	631	855	17
Chr6	2.5E+07	24800000		688	524	654	11
Chr6	2.5E+07	24900000		454	368	381	8

					SNP		
				All	Bengal/Nona Bokra	PSSR/Bengal	PSSR/Nona Bokra
Chr6	2.5E+07	25000000		696	537	528	7
Chr6	2.5E+07	25100000		651	461	628	8
Chr6	2.5E+07	25200000		508	373	488	2
Chr6	2.5E+07	25300000		228	144	214	4
Chr6	2.5E+07	25400000		321	224	300	0
Chr6	2.5E+07	25500000		334	235	307	3
Chr6	2.6E+07	25600000		303	167	218	5
Chr6	2.6E+07	25700000		457	290	357	2
Chr6	2.6E+07	25800000		648	493	606	1
Chr6	2.6E+07	25900000		528	397	502	1
Chr6	2.6E+07	26000000		593	467	568	1
Chr6	2.6E+07	26100000		783	564	730	6
Chr6	2.6E+07	26200000		806	588	744	6
Chr6	2.6E+07	26300000		652	466	568	3
Chr6	2.6E+07	26400000		632	503	595	0
Chr6	2.6E+07	26500000		663	543	645	7
Chr6	2.7E+07	26600000		831	644	786	5
Chr6	2.7E+07	26700000		699	525	657	6
Chr6	2.7E+07	26800000		903	657	835	10
Chr6	2.7E+07	26900000		695	495	665	9
Chr6	2.7E+07	27000000		795	434	514	7
Chr6	2.7E+07	27100000		1085	440	523	11
Chr6	2.7E+07	27200000		699	444	483	118
Chr6	2.7E+07	27300000		687	463	538	260
Chr6	2.7E+07	27400000		660	395	515	242
Chr6	2.7E+07	27500000		841	435	633	395
Chr6	2.8E+07	27600000		731	524	348	462
Chr6	2.8E+07	27700000		115	100	10	101
Chr6	2.8E+07	27800000		209	6	172	169
Chr6	2.8E+07	27900000		725	15	702	621
Chr6	2.8E+07	28000000		592	175	405	361
Chr6	2.8E+07	28100000		939	567	612	449
Chr6	2.8E+07	28200000		703	451	581	180
Chr6	2.8E+07	28300000		688	432	562	203
Chr6	2.8E+07	28400000		797	508	676	168
Chr6	2.8E+07	28500000		608	391	454	213
Chr6	2.9E+07	28600000		858	419	699	204
Chr6	2.9E+07	28700000		775	517	631	248
Chr6	2.9E+07	28800000		585	444	473	69
Chr6	2.9E+07	28900000		675	455	526	177
Chr6	2.9E+07	29000000		700	507	588	184
Chr6	2.9E+07	29100000		734	386	554	308
Chr6	2.9E+07	29200000		667	20	633	578
Chr6	2.9E+07	29300000		316	8	304	280
Chr6	2.9E+07	29400000		330	11	315	271

					SNP		
				All	Bengal/Nona Bokra	PSSR/Bengal	PSSR/Nona Bokra
Chr6	2.9E+07	29500000		490	15	474	427
Chr6	3E+07	29600000		607	248	509	188
Chr6	3E+07	29700000		832	366	463	25
Chr6	3E+07	29800000		677	203	314	224
Chr6	3E+07	29900000		778	247	289	233
Chr6	3E+07	30000000		341	95	94	12
Chr6	3E+07	30100000		606	174	209	30
Chr6	3E+07	30200000		650	231	278	63
Chr6	3E+07	30300000		274	88	90	2
Chr6	3E+07	30400000		430	71	85	5
Chr6	3E+07	30500000		479	115	124	8
Chr6	3.1E+07	30600000		307	114	40	75
Chr6	3.1E+07	30700000		23	3	1	2
Chr6	3.1E+07	30800000		235	24	131	137
Chr6	3.1E+07	30900000		425	10	12	9
Chr6	3.1E+07	31000000		567	8	4	4
Chr6	3.1E+07	31100000		468	7	8	7
Chr6	3.1E+07	31200000		416	6	4	5
Chr6	3.1E+07	31300000		146	1	1	0
Chr7	Chr7						
Chr7	1	100000		535	418	498	7
Chr7	100001	200000		728	440	570	19
Chr7	200001	300000		817	412	506	3
Chr7	300001	400000		431	317	397	3
Chr7	400001	500000		644	458	538	129
Chr7	500001	600000		536	403	438	105
Chr7	600001	700000		970	405	712	423
Chr7	700001	800000		754	526	568	186
Chr7	800001	900000		595	463	549	3
Chr7	900001	1000000		904	793	824	4
Chr7	1000001	1100000		573	440	551	3
Chr7	1100001	1200000		715	485	596	128
Chr7	1200001	1300000		641	432	482	256
Chr7	1300001	1400000		114	89	109	4
Chr7	1400001	1500000		346	254	319	10
Chr7	1500001	1600000		829	616	683	9
Chr7	1600001	1700000		786	348	318	290
Chr7	1700001	1800000		912	405	348	128
Chr7	1800001	1900000		553	393	505	7
Chr7	1900001	2000000		771	574	709	11
Chr7	2000001	2100000		819	510	637	339
Chr7	2100001	2200000		668	370	520	283
Chr7	2200001	2300000		811	503	623	280
Chr7	2300001	2400000		599	366	374	208
Chr7	2400001	2500000		401	303	349	4

					SNP		
				All	Bengal/Nona Bokra	PSSR/Bengal	PSSR/Nona Bokra
Chr7	2500001	2600000		806	556	743	8
Chr7	2600001	2700000		777	617	746	12
Chr7	2700001	2800000		131	81	85	4
Chr7	2800001	2900000		27	7	12	14
Chr7	2900001	3000000		24	8	8	7
Chr7	3000001	3100000		16	1	7	6
Chr7	3100001	3200000		449	8	9	2
Chr7	3200001	3300000		307	168	221	13
Chr7	3300001	3400000		507	483	15	485
Chr7	3400001	3500000		373	363	10	364
Chr7	3500001	3600000		37	18	22	7
Chr7	3600001	3700000		37	17	25	6
Chr7	3700001	3800000		56	24	24	12
Chr7	3800001	3900000		293	8	11	2
Chr7	3900001	4000000		406	21	28	11
Chr7	4000001	4100000		71	49	61	5
Chr7	4100001	4200000		537	384	477	27
Chr7	4200001	4300000		609	565	106	498
Chr7	4300001	4400000		627	597	17	605
Chr7	4400001	4500000		446	405	19	413
Chr7	4500001	4600000		618	492	24	493
Chr7	4600001	4700000		817	458	598	181
Chr7	4700001	4800000		795	561	164	544
Chr7	4800001	4900000		597	239	527	272
Chr7	4900001	5000000		681	253	612	407
Chr7	5000001	5100000		777	469	723	113
Chr7	5100001	5200000		672	216	643	327
Chr7	5200001	5300000		470	350	391	5
Chr7	5300001	5400000		762	549	696	9
Chr7	5400001	5500000		716	522	678	12
Chr7	5500001	5600000		627	504	585	9
Chr7	5600001	5700000		690	227	663	310
Chr7	5700001	5800000		601	106	586	404
Chr7	5800001	5900000		641	604	111	518
Chr7	5900001	6000000		464	431	21	441
Chr7	6000001	6100000		715	527	410	391
Chr7	6100001	6200000		564	367	412	68
Chr7	6200001	6300000		812	604	582	101
Chr7	6300001	6400000		382	235	251	28
Chr7	6400001	6500000		322	183	155	10
Chr7	6500001	6600000		97	33	36	3
Chr7	6600001	6700000		658	492	554	9
Chr7	6700001	6800000		857	630	793	9
Chr7	6800001	6900000		930	677	823	12
Chr7	6900001	7000000		802	507	685	169

					SNP		
				All	Bengal/Nona Bokra	PSSR/Bengal	PSSR/Nona Bokra
Chr7	7000001	7100000		875	608	798	14
Chr7	7100001	7200000		772	508	674	80
Chr7	7200001	7300000		764	538	565	61
Chr7	7300001	7400000		907	673	762	11
Chr7	7400001	7500000		774	479	705	12
Chr7	7500001	7600000		1004	657	945	24
Chr7	7600001	7700000		808	582	744	18
Chr7	7700001	7800000		777	562	666	124
Chr7	7800001	7900000		791	477	679	77
Chr7	7900001	8000000		792	517	658	174
Chr7	8000001	8100000		955	553	795	274
Chr7	8100001	8200000		957	557	631	486
Chr7	8200001	8300000		635	332	497	165
Chr7	8300001	8400000		1100	768	1035	23
Chr7	8400001	8500000		921	567	845	93
Chr7	8500001	8600000		1081	621	812	459
Chr7	8600001	8700000		1025	471	773	577
Chr7	8700001	8800000		1030	641	838	316
Chr7	8800001	8900000		858	565	730	201
Chr7	8900001	9000000		925	535	731	351
Chr7	9000001	9100000		808	530	687	193
Chr7	9100001	9200000		792	542	734	8
Chr7	9200001	9300000		898	655	806	10
Chr7	9300001	9400000		860	594	769	14
Chr7	9400001	9500000		716	476	657	11
Chr7	9500001	9600000		642	432	559	12
Chr7	9600001	9700000		281	141	230	14
Chr7	9700001	9800000		367	212	318	11
Chr7	9800001	9900000		448	275	402	8
Chr7	9900001	10000000		358	157	285	130
Chr7	1E+07	10100000		552	403	433	18
Chr7	1E+07	10200000		437	310	416	5
Chr7	1E+07	10300000		761	546	708	8
Chr7	1E+07	10400000		417	274	384	6
Chr7	1E+07	10500000		519	332	463	6
Chr7	1.1E+07	10600000		779	562	707	7
Chr7	1.1E+07	10700000		715	542	691	13
Chr7	1.1E+07	10800000		876	626	831	11
Chr7	1.1E+07	10900000		482	350	454	10
Chr7	1.1E+07	11000000		698	523	668	5
Chr7	1.1E+07	11100000		685	502	663	7
Chr7	1.1E+07	11200000		554	360	484	7
Chr7	1.1E+07	11300000		824	649	628	3
Chr7	1.1E+07	11400000		910	688	821	6
Chr7	1.1E+07	11500000		520	365	452	10

					SNP		
				All	Bengal/Nona Bokra	PSSR/Bengal	PSSR/Nona Bokra
Chr7	1.2E+07	11600000		1025	824	914	18
Chr7	1.2E+07	11700000		829	655	728	9
Chr7	1.2E+07	11800000		566	417	528	12
Chr7	1.2E+07	11900000		466	353	437	6
Chr7	1.2E+07	12000000		660	520	619	8
Chr7	1.2E+07	12100000		444	285	357	14
Chr7	1.2E+07	12200000		312	158	244	4
Chr7	1.2E+07	12300000		158	82	143	8
Chr7	1.2E+07	12400000		579	423	514	8
Chr7	1.2E+07	12500000		297	215	263	15
Chr7	1.3E+07	12600000		486	344	449	9
Chr7	1.3E+07	12700000		424	314	334	6
Chr7	1.3E+07	12800000		452	323	437	12
Chr7	1.3E+07	12900000		563	391	539	10
Chr7	1.3E+07	13000000		349	285	256	6
Chr7	1.3E+07	13100000		816	655	642	7
Chr7	1.3E+07	13200000		661	455	617	22
Chr7	1.3E+07	13300000		938	654	837	15
Chr7	1.3E+07	13400000		1145	859	991	8
Chr7	1.3E+07	13500000		448	326	425	13
Chr7	1.4E+07	13600000		424	292	399	8
Chr7	1.4E+07	13700000		942	649	882	5
Chr7	1.4E+07	13800000		658	512	638	9
Chr7	1.4E+07	13900000		501	331	457	12
Chr7	1.4E+07	14000000		93	55	71	1
Chr7	1.4E+07	14100000		84	25	66	2
Chr7	1.4E+07	14200000		131	62	117	0
Chr7	1.4E+07	14300000		94	39	59	0
Chr7	1.4E+07	14400000		176	97	135	3
Chr7	1.4E+07	14500000		283	110	225	17
Chr7	1.5E+07	14600000		858	664	788	5
Chr7	1.5E+07	14700000		1084	591	895	310
Chr7	1.5E+07	14800000		862	534	690	327
Chr7	1.5E+07	14900000		267	155	201	69
Chr7	1.5E+07	15000000		1049	706	859	148
Chr7	1.5E+07	15100000		732	385	499	415
Chr7	1.5E+07	15200000		539	175	386	50
Chr7	1.5E+07	15300000		816	629	786	16
Chr7	1.5E+07	15400000		1588	969	912	59
Chr7	1.5E+07	15500000		1111	435	134	317
Chr7	1.6E+07	15600000		1265	537	432	542
Chr7	1.6E+07	15700000		1221	588	748	489
Chr7	1.6E+07	15800000		1172	700	894	264
Chr7	1.6E+07	15900000		1227	745	938	203
Chr7	1.6E+07	16000000		784	559	737	11

					SNP		
				All	Bengal/Nona Bokra	PSSR/Bengal	PSSR/Nona Bokra
Chr7	1.6E+07	16100000		1013	616	787	399
Chr7	1.6E+07	16200000		1222	614	847	615
Chr7	1.6E+07	16300000		1049	467	807	299
Chr7	1.6E+07	16400000		1262	763	869	609
Chr7	1.6E+07	16500000		955	519	738	369
Chr7	1.7E+07	16600000		1063	488	841	382
Chr7	1.7E+07	16700000		945	605	678	350
Chr7	1.7E+07	16800000		739	474	321	472
Chr7	1.7E+07	16900000		607	341	454	184
Chr7	1.7E+07	17000000		721	538	643	102
Chr7	1.7E+07	17100000		546	374	500	4
Chr7	1.7E+07	17200000		680	428	598	100
Chr7	1.7E+07	17300000		767	487	606	195
Chr7	1.7E+07	17400000		1049	673	857	319
Chr7	1.7E+07	17500000		812	386	670	271
Chr7	1.8E+07	17600000		503	19	475	410
Chr7	1.8E+07	17700000		505	23	477	396
Chr7	1.8E+07	17800000		738	12	710	608
Chr7	1.8E+07	17900000		525	9	510	439
Chr7	1.8E+07	18000000		187	12	160	125
Chr7	1.8E+07	18100000		286	5	166	124
Chr7	1.8E+07	18200000		720	273	526	433
Chr7	1.8E+07	18300000		422	257	164	266
Chr7	1.8E+07	18400000		617	252	333	302
Chr7	1.8E+07	18500000		1231	235	388	99
Chr7	1.9E+07	18600000		885	417	335	247
Chr7	1.9E+07	18700000		709	318	318	200
Chr7	1.9E+07	18800000		1029	331	389	221
Chr7	1.9E+07	18900000		1011	585	332	518
Chr7	1.9E+07	19000000		968	406	484	343
Chr7	1.9E+07	19100000		1066	287	356	302
Chr7	1.9E+07	19200000		1049	367	474	8
Chr7	1.9E+07	19300000		654	221	260	2
Chr7	1.9E+07	19400000		994	298	348	100
Chr7	1.9E+07	19500000		1051	279	406	216
Chr7	2E+07	19600000		936	337	421	367
Chr7	2E+07	19700000		785	317	349	156
Chr7	2E+07	19800000		880	268	319	26
Chr7	2E+07	19900000		700	181	239	5
Chr7	2E+07	20000000		864	308	384	6
Chr7	2E+07	20100000		1017	493	456	2
Chr7	2E+07	20200000		940	142	154	8
Chr7	2E+07	20300000		520	78	101	2
Chr7	2E+07	20400000		796	178	214	13
Chr7	2E+07	20500000		778	349	438	6

					SNP		
				All	Bengal/Nona Bokra	PSSR/Bengal	PSSR/Nona Bokra
Chr7	2.1E+07	20600000		1362	462	599	6
Chr7	2.1E+07	20700000		866	298	391	9
Chr7	2.1E+07	20800000		965	348	407	14
Chr7	2.1E+07	20900000		870	215	152	126
Chr7	2.1E+07	21000000		1306	415	747	655
Chr7	2.1E+07	21100000		931	244	351	230
Chr7	2.1E+07	21200000		946	491	430	307
Chr7	2.1E+07	21300000		815	520	418	517
Chr7	2.1E+07	21400000		1310	488	499	368
Chr7	2.1E+07	21500000		1172	431	472	433
Chr7	2.2E+07	21600000		496	364	204	184
Chr7	2.2E+07	21700000		990	767	812	29
Chr7	2.2E+07	21800000		1038	805	853	141
Chr7	2.2E+07	21900000		920	562	608	412
Chr7	2.2E+07	22000000		626	454	473	16
Chr7	2.2E+07	22100000		671	509	635	11
Chr7	2.2E+07	22200000		540	370	494	65
Chr7	2.2E+07	22300000		784	460	604	133
Chr7	2.2E+07	22400000		799	528	671	173
Chr7	2.2E+07	22500000		944	399	565	368
Chr7	2.3E+07	22600000		783	493	604	250
Chr7	2.3E+07	22700000		401	261	336	144
Chr7	2.3E+07	22800000		623	432	539	112
Chr7	2.3E+07	22900000		658	487	525	124
Chr7	2.3E+07	23000000		996	432	708	569
Chr7	2.3E+07	23100000		581	301	376	174
Chr7	2.3E+07	23200000		689	389	433	244
Chr7	2.3E+07	23300000		776	415	589	140
Chr7	2.3E+07	23400000		819	356	464	11
Chr7	2.3E+07	23500000		844	463	575	8
Chr7	2.4E+07	23600000		665	448	555	12
Chr7	2.4E+07	23700000		709	531	687	9
Chr7	2.4E+07	23800000		739	546	697	10
Chr7	2.4E+07	23900000		588	437	566	5
Chr7	2.4E+07	24000000		553	399	519	5
Chr7	2.4E+07	24100000		825	586	714	7
Chr7	2.4E+07	24200000		685	410	482	7
Chr7	2.4E+07	24300000		706	423	527	195
Chr7	2.4E+07	24400000		708	320	415	159
Chr7	2.4E+07	24500000		1032	699	828	2
Chr7	2.5E+07	24600000		1111	750	763	8
Chr7	2.5E+07	24700000		864	459	569	13
Chr7	2.5E+07	24800000		800	487	588	115
Chr7	2.5E+07	24900000		381	260	288	28
Chr7	2.5E+07	25000000		639	527	616	6

					SNP		
				All	Bengal/Nona Bokra	PSSR/Bengal	PSSR/Nona Bokra
Chr7	2.5E+07	25100000		273	205	241	4
Chr7	2.5E+07	25200000		341	245	254	0
Chr7	2.5E+07	25300000		631	451	490	15
Chr7	2.5E+07	25400000		716	410	530	107
Chr7	2.5E+07	25500000		649	486	566	14
Chr7	2.6E+07	25600000		668	504	602	10
Chr7	2.6E+07	25700000		609	446	553	75
Chr7	2.6E+07	25800000		699	451	609	122
Chr7	2.6E+07	25900000		653	422	570	82
Chr7	2.6E+07	26000000		680	452	568	190
Chr7	2.6E+07	26100000		1067	631	745	397
Chr7	2.6E+07	26200000		621	396	508	118
Chr7	2.6E+07	26300000		635	457	583	51
Chr7	2.6E+07	26400000		699	522	676	4
Chr7	2.6E+07	26500000		565	433	557	3
Chr7	2.7E+07	26600000		518	402	497	4
Chr7	2.7E+07	26700000		430	355	420	3
Chr7	2.7E+07	26800000		1022	816	681	133
Chr7	2.7E+07	26900000		695	545	542	108
Chr7	2.7E+07	27000000		324	193	283	101
Chr7	2.7E+07	27100000		474	148	396	337
Chr7	2.7E+07	27200000		578	220	373	323
Chr7	2.7E+07	27300000		204	137	61	40
Chr7	2.7E+07	27400000		154	111	45	26
Chr7	2.7E+07	27500000		494	310	190	179
Chr7	2.8E+07	27600000		892	543	642	228
Chr7	2.8E+07	27700000		670	458	529	217
Chr7	2.8E+07	27800000		933	746	806	106
Chr7	2.8E+07	27900000		616	388	479	259
Chr7	2.8E+07	28000000		858	531	647	284
Chr7	2.8E+07	28100000		714	483	301	490
Chr7	2.8E+07	28200000		184	173	17	175
Chr7	2.8E+07	28300000		25	10	17	21
Chr7	2.8E+07	28400000		481	364	189	379
Chr7	2.8E+07	28500000		684	415	517	332
Chr7	2.9E+07	28600000		491	380	469	14
Chr7	2.9E+07	28700000		470	385	422	13
Chr7	2.9E+07	28800000		651	208	532	460
Chr7	2.9E+07	28900000		641	377	475	329
Chr7	2.9E+07	29000000		668	486	527	190
Chr7	2.9E+07	29100000		568	475	543	10
Chr7	2.9E+07	29200000		539	445	530	3
Chr7	2.9E+07	29300000		533	345	449	158
Chr7	2.9E+07	29400000		766	449	568	406
Chr7	2.9E+07	29500000		732	511	680	44

					SNP		
				All	Bengal/Nona Bokra	PSSR/Bengal	PSSR/Nona Bokra
Chr7	3E+07	29600000		702	391	530	282
Chr7	3E+07	29700000		717	463	564	168
Chr8	Chr8						
Chr8	1	100000		623	177	204	4
Chr8	100001	200000		728	329	353	2
Chr8	200001	300000		825	450	358	183
Chr8	300001	400000		705	437	288	502
Chr8	400001	500000		600	121	493	487
Chr8	500001	600000		461	30	436	396
Chr8	600001	700000		505	17	485	432
Chr8	700001	800000		675	12	653	557
Chr8	800001	900000		626	94	582	445
Chr8	900001	1000000		157	9	146	125
Chr8	1000001	1100000		523	464	106	417
Chr8	1100001	1200000		664	287	383	209
Chr8	1200001	1300000		411	178	199	146
Chr8	1300001	1400000		400	175	244	65
Chr8	1400001	1500000		438	163	357	82
Chr8	1500001	1600000		1083	548	759	392
Chr8	1600001	1700000		862	470	576	252
Chr8	1700001	1800000		733	404	433	186
Chr8	1800001	1900000		664	127	164	96
Chr8	1900001	2000000		608	173	331	172
Chr8	2000001	2100000		1033	716	746	399
Chr8	2100001	2200000		712	521	568	188
Chr8	2200001	2300000		748	501	680	104
Chr8	2300001	2400000		712	436	591	199
Chr8	2400001	2500000		413	358	113	315
Chr8	2500001	2600000		155	125	13	124
Chr8	2600001	2700000		339	185	215	19
Chr8	2700001	2800000		491	224	266	6
Chr8	2800001	2900000		553	391	309	19
Chr8	2900001	3000000		923	710	581	219
Chr8	3000001	3100000		743	518	286	533
Chr8	3100001	3200000		539	139	349	208
Chr8	3200001	3300000		710	264	572	370
Chr8	3300001	3400000		701	491	582	70
Chr8	3400001	3500000		792	555	633	5
Chr8	3500001	3600000		777	527	673	97
Chr8	3600001	3700000		646	421	553	7
Chr8	3700001	3800000		570	280	338	88
Chr8	3800001	3900000		538	276	392	68
Chr8	3900001	4000000		766	430	541	5
Chr8	4000001	4100000		817	573	658	6
Chr8	4100001	4200000		518	383	488	2

					SNP		
				All	Bengal/Nona Bokra	PSSR/Bengal	PSSR/Nona Bokra
Chr8	4200001	4300000		630	424	596	14
Chr8	4300001	4400000		1173	704	820	7
Chr8	4400001	4500000		421	344	394	2
Chr8	4500001	4600000		561	441	534	32
Chr8	4600001	4700000		661	402	533	236
Chr8	4700001	4800000		698	502	604	9
Chr8	4800001	4900000		420	319	243	3
Chr8	4900001	5000000		286	213	238	4
Chr8	5000001	5100000		743	529	668	66
Chr8	5100001	5200000		610	373	537	58
Chr8	5200001	5300000		847	601	723	127
Chr8	5300001	5400000		568	363	413	283
Chr8	5400001	5500000		586	451	573	4
Chr8	5500001	5600000		688	391	511	193
Chr8	5600001	5700000		1175	715	745	536
Chr8	5700001	5800000		869	418	637	517
Chr8	5800001	5900000		604	432	315	315
Chr8	5900001	6000000		634	333	406	144
Chr8	6000001	6100000		885	478	526	444
Chr8	6100001	6200000		910	462	636	413
Chr8	6200001	6300000		689	411	567	249
Chr8	6300001	6400000		282	157	167	68
Chr8	6400001	6500000		294	74	219	69
Chr8	6500001	6600000		294	144	206	49
Chr8	6600001	6700000		129	55	76	35
Chr8	6700001	6800000		180	33	120	36
Chr8	6800001	6900000		146	72	87	48
Chr8	6900001	7000000		169	57	111	17
Chr8	7000001	7100000		153	57	96	15
Chr8	7100001	7200000		562	239	464	178
Chr8	7200001	7300000		482	264	305	217
Chr8	7300001	7400000		466	309	211	326
Chr8	7400001	7500000		830	461	605	406
Chr8	7500001	7600000		396	241	348	80
Chr8	7600001	7700000		639	470	602	6
Chr8	7700001	7800000		858	731	641	8
Chr8	7800001	7900000		647	488	540	5
Chr8	7900001	8000000		698	546	630	4
Chr8	8000001	8100000		741	558	681	15
Chr8	8100001	8200000		483	361	439	12
Chr8	8200001	8300000		869	623	725	17
Chr8	8300001	8400000		577	423	463	8
Chr8	8400001	8500000		697	471	426	266
Chr8	8500001	8600000		962	558	479	570
Chr8	8600001	8700000		850	516	743	167

					SNP		
				All	Bengal/Nona Bokra	PSSR/Bengal	PSSR/Nona Bokra
Chr8	8700001	8800000		788	606	722	2
Chr8	8800001	8900000		923	746	700	6
Chr8	8900001	9000000		897	613	740	174
Chr8	9000001	9100000		1091	721	727	351
Chr8	9100001	9200000		828	575	789	17
Chr8	9200001	9300000		524	307	458	99
Chr8	9300001	9400000		933	519	776	299
Chr8	9400001	9500000		945	775	660	83
Chr8	9500001	9600000		547	360	356	224
Chr8	9600001	9700000		683	371	495	313
Chr8	9700001	9800000		713	455	464	270
Chr8	9800001	9900000		757	425	510	330
Chr8	9900001	10000000		842	340	651	359
Chr8	1E+07	10100000		995	360	764	395
Chr8	1E+07	10200000		889	451	653	500
Chr8	1E+07	10300000		916	465	695	501
Chr8	1E+07	10400000		970	604	789	329
Chr8	1E+07	10500000		946	508	824	201
Chr8	1.1E+07	10600000		1051	563	894	210
Chr8	1.1E+07	10700000		1060	671	846	385
Chr8	1.1E+07	10800000		1056	602	728	510
Chr8	1.1E+07	10900000		1007	560	819	360
Chr8	1.1E+07	11000000		874	470	619	450
Chr8	1.1E+07	11100000		544	298	316	251
Chr8	1.1E+07	11200000		430	157	329	195
Chr8	1.1E+07	11300000		413	206	310	217
Chr8	1.1E+07	11400000		951	476	715	551
Chr8	1.1E+07	11500000		794	379	599	430
Chr8	1.2E+07	11600000		682	321	485	396
Chr8	1.2E+07	11700000		625	358	341	330
Chr8	1.2E+07	11800000		802	383	563	471
Chr8	1.2E+07	11900000		1150	674	801	579
Chr8	1.2E+07	12000000		1211	585	705	497
Chr8	1.2E+07	12100000		1187	730	481	433
Chr8	1.2E+07	12200000		475	179	358	230
Chr8	1.2E+07	12300000		282	159	159	138
Chr8	1.2E+07	12400000		541	328	301	387
Chr8	1.2E+07	12500000		419	167	325	194
Chr8	1.3E+07	12600000		308	128	251	140
Chr8	1.3E+07	12700000		464	250	286	146
Chr8	1.3E+07	12800000		399	215	299	155
Chr8	1.3E+07	12900000		530	252	431	275
Chr8	1.3E+07	13000000		277	123	181	87
Chr8	1.3E+07	13100000		430	211	320	209
Chr8	1.3E+07	13200000		314	134	219	101

					SNP		
				All	Bengal/Nona Bokra	PSSR/Bengal	PSSR/Nona Bokra
Chr8	1.3E+07	13300000		54	25	28	7
Chr8	1.3E+07	13400000		284	130	216	144
Chr8	1.3E+07	13500000		450	266	288	170
Chr8	1.4E+07	13600000		539	288	349	202
Chr8	1.4E+07	13700000		550	190	439	158
Chr8	1.4E+07	13800000		437	211	342	235
Chr8	1.4E+07	13900000		373	178	279	160
Chr8	1.4E+07	14000000		440	200	347	216
Chr8	1.4E+07	14100000		511	160	425	155
Chr8	1.4E+07	14200000		430	202	337	228
Chr8	1.4E+07	14300000		557	279	385	272
Chr8	1.4E+07	14400000		370	221	211	240
Chr8	1.4E+07	14500000		478	227	369	260
Chr8	1.5E+07	14600000		540	295	401	190
Chr8	1.5E+07	14700000		524	308	329	220
Chr8	1.5E+07	14800000		433	176	332	270
Chr8	1.5E+07	14900000		191	48	165	109
Chr8	1.5E+07	15000000		752	291	576	440
Chr8	1.5E+07	15100000		1090	343	864	743
Chr8	1.5E+07	15200000		631	339	477	301
Chr8	1.5E+07	15300000		439	213	331	235
Chr8	1.5E+07	15400000		790	432	521	435
Chr8	1.5E+07	15500000		708	448	621	163
Chr8	1.6E+07	15600000		653	458	619	4
Chr8	1.6E+07	15700000		715	525	661	9
Chr8	1.6E+07	15800000		476	272	336	7
Chr8	1.6E+07	15900000		600	332	413	5
Chr8	1.6E+07	16000000		790	308	337	3
Chr8	1.6E+07	16100000		545	175	235	15
Chr8	1.6E+07	16200000		444	26	28	0
Chr8	1.6E+07	16300000		170	6	8	2
Chr8	1.6E+07	16400000		1188	920	949	4
Chr8	1.6E+07	16500000		730	438	582	233
Chr8	1.7E+07	16600000		618	293	363	329
Chr8	1.7E+07	16700000		1068	275	720	552
Chr8	1.7E+07	16800000		705	164	172	154
Chr8	1.7E+07	16900000		822	342	212	146
Chr8	1.7E+07	17000000		1260	529	696	289
Chr8	1.7E+07	17100000		852	361	510	264
Chr8	1.7E+07	17200000		1281	601	719	562
Chr8	1.7E+07	17300000		631	177	472	167
Chr8	1.7E+07	17400000		1182	643	665	574
Chr8	1.7E+07	17500000		661	158	484	151
Chr8	1.8E+07	17600000		1138	475	453	373
Chr8	1.8E+07	17700000		1315	655	819	3

					SNP		
				All	Bengal/Nona Bokra	PSSR/Bengal	PSSR/Nona Bokra
Chr8	1.8E+07	17800000		449	175	209	4
Chr8	1.8E+07	17900000		1058	405	447	1
Chr8	1.8E+07	18000000		728	330	408	3
Chr8	1.8E+07	18100000		1276	620	755	6
Chr8	1.8E+07	18200000		696	401	542	9
Chr8	1.8E+07	18300000		760	418	540	0
Chr8	1.8E+07	18400000		804	527	719	12
Chr8	1.8E+07	18500000		448	321	402	11
Chr8	1.9E+07	18600000		780	464	609	197
Chr8	1.9E+07	18700000		1291	775	841	483
Chr8	1.9E+07	18800000		943	685	259	230
Chr8	1.9E+07	18900000		928	621	347	312
Chr8	1.9E+07	19000000		707	533	341	59
Chr8	1.9E+07	19100000		950	644	683	278
Chr8	1.9E+07	19200000		831	486	684	172
Chr8	1.9E+07	19300000		975	532	850	272
Chr8	1.9E+07	19400000		1055	506	695	579
Chr8	1.9E+07	19500000		1013	609	785	360
Chr8	2E+07	19600000		875	584	764	116
Chr8	2E+07	19700000		1230	671	685	492
Chr8	2E+07	19800000		929	522	532	445
Chr8	2E+07	19900000		1248	528	892	580
Chr8	2E+07	20000000		913	550	564	407
Chr8	2E+07	20100000		1059	559	625	623
Chr8	2E+07	20200000		957	662	731	217
Chr8	2E+07	20300000		956	686	258	733
Chr8	2E+07	20400000		664	412	440	177
Chr8	2E+07	20500000		912	607	792	158
Chr8	2.1E+07	20600000		580	409	513	27
Chr8	2.1E+07	20700000		955	581	735	174
Chr8	2.1E+07	20800000		738	563	689	6
Chr8	2.1E+07	20900000		826	501	648	7
Chr8	2.1E+07	21000000		798	406	338	119
Chr8	2.1E+07	21100000		804	289	5	292
Chr8	2.1E+07	21200000		936	221	8	218
Chr8	2.1E+07	21300000		934	331	12	324
Chr8	2.1E+07	21400000		1144	573	694	52
Chr8	2.1E+07	21500000		625	203	237	4
Chr8	2.2E+07	21600000		740	622	725	8
Chr8	2.2E+07	21700000		725	529	694	7
Chr8	2.2E+07	21800000		980	279	374	7
Chr8	2.2E+07	21900000		878	307	335	0
Chr8	2.2E+07	22000000		544	117	147	10
Chr8	2.2E+07	22100000		568	454	548	11
Chr8	2.2E+07	22200000		942	692	912	13

					SNP		
				All	Bengal/Nona Bokra	PSSR/Bengal	PSSR/Nona Bokra
Chr8	2.2E+07	22300000		805	569	737	10
Chr8	2.2E+07	22400000		653	463	599	7
Chr8	2.2E+07	22500000		657	468	614	5
Chr8	2.3E+07	22600000		435	328	375	8
Chr8	2.3E+07	22700000		496	370	464	3
Chr8	2.3E+07	22800000		803	550	732	6
Chr8	2.3E+07	22900000		644	442	575	7
Chr8	2.3E+07	23000000		635	487	605	2
Chr8	2.3E+07	23100000		742	567	711	12
Chr8	2.3E+07	23200000		596	441	580	6
Chr8	2.3E+07	23300000		714	544	682	7
Chr8	2.3E+07	23400000		554	387	539	4
Chr8	2.3E+07	23500000		681	536	645	8
Chr8	2.4E+07	23600000		575	417	517	7
Chr8	2.4E+07	23700000		402	300	376	10
Chr8	2.4E+07	23800000		27	17	20	3
Chr8	2.4E+07	23900000		17	11	12	7
Chr8	2.4E+07	24000000		18	7	13	3
Chr8	2.4E+07	24100000		40	25	32	9
Chr8	2.4E+07	24200000		11	7	5	7
Chr8	2.4E+07	24300000		15	7	11	9
Chr8	2.4E+07	24400000		389	13	379	319
Chr8	2.4E+07	24500000		610	12	593	507
Chr8	2.5E+07	24600000		823	25	805	656
Chr8	2.5E+07	24700000		476	11	462	407
Chr8	2.5E+07	24800000		809	101	752	657
Chr8	2.5E+07	24900000		811	39	756	673
Chr8	2.5E+07	25000000		78	18	69	53
Chr8	2.5E+07	25100000		40	30	29	13
Chr8	2.5E+07	25200000		41	20	22	14
Chr8	2.5E+07	25300000		46	29	24	8
Chr8	2.5E+07	25400000		407	344	77	318
Chr8	2.5E+07	25500000		707	444	657	93
Chr8	2.6E+07	25600000		616	398	480	83
Chr8	2.6E+07	25700000		516	490	15	499
Chr8	2.6E+07	25800000		507	478	18	475
Chr8	2.6E+07	25900000		355	318	104	244
Chr8	2.6E+07	26000000		438	311	405	9
Chr8	2.6E+07	26100000		731	434	571	308
Chr8	2.6E+07	26200000		878	455	575	273
Chr8	2.6E+07	26300000		951	293	456	290
Chr8	2.6E+07	26400000		828	212	233	234
Chr8	2.6E+07	26500000		902	249	400	260
Chr8	2.7E+07	26600000		845	549	694	260
Chr8	2.7E+07	26700000		202	69	138	58

					SNP		
				All	Bengal/Nona Bokra	PSSR/Bengal	PSSR/Nona Bokra
Chr8	2.7E+07	26800000		657	246	301	254
Chr8	2.7E+07	26900000		977	216	406	348
Chr8	2.7E+07	27000000		849	406	486	363
Chr8	2.7E+07	27100000		988	551	568	519
Chr8	2.7E+07	27200000		652	441	451	326
Chr8	2.7E+07	27300000		716	505	619	117
Chr8	2.7E+07	27400000		824	521	653	255
Chr8	2.7E+07	27500000		921	570	698	419
Chr8	2.8E+07	27600000		734	464	572	269
Chr8	2.8E+07	27700000		525	325	362	301
Chr8	2.8E+07	27800000		830	585	737	72
Chr8	2.8E+07	27900000		593	421	522	113
Chr8	2.8E+07	28000000		539	394	463	60
Chr8	2.8E+07	28100000		661	338	419	161
Chr8	2.8E+07	28200000		812	511	557	253
Chr8	2.8E+07	28300000		597	430	502	113
Chr8	2.8E+07	28400000		531	434	504	7
Chr8	2.8E+07	28500000		247	161	199	16
Chr9	Chr9						
Chr9	1	100000		164	79	119	27
Chr9	100001	200000		364	231	293	19
Chr9	200001	300000		731	543	530	56
Chr9	300001	400000		393	274	280	22
Chr9	400001	500000		536	386	465	121
Chr9	500001	600000		522	296	435	118
Chr9	600001	700000		1119	596	904	364
Chr9	700001	800000		889	498	739	228
Chr9	800001	900000		825	548	738	117
Chr9	900001	1000000		874	579	753	145
Chr9	1000001	1100000		864	558	728	189
Chr9	1100001	1200000		659	457	577	96
Chr9	1200001	1300000		592	453	553	29
Chr9	1300001	1400000		614	418	579	30
Chr9	1400001	1500000		635	358	581	22
Chr9	1500001	1600000		671	459	603	45
Chr9	1600001	1700000		412	269	387	25
Chr9	1700001	1800000		684	526	644	39
Chr9	1800001	1900000		686	460	626	27
Chr9	1900001	2000000		730	518	688	28
Chr9	2000001	2100000		872	658	814	35
Chr9	2100001	2200000		483	326	439	21
Chr9	2200001	2300000		415	217	367	21
Chr9	2300001	2400000		595	432	530	31
Chr9	2400001	2500000		589	408	527	23
Chr9	2500001	2600000		659	464	611	24

					SNP		
				All	Bengal/Nona Bokra	PSSR/Bengal	PSSR/Nona Bokra
Chr9	2600001	2700000		548	408	493	31
Chr9	2700001	2800000		302	157	252	11
Chr9	2800001	2900000		273	167	234	9
Chr9	2900001	3000000		784	553	730	49
Chr9	3000001	3100000		684	495	620	38
Chr9	3100001	3200000		818	570	743	26
Chr9	3200001	3300000		473	291	417	23
Chr9	3300001	3400000		470	348	433	39
Chr9	3400001	3500000		680	477	610	41
Chr9	3500001	3600000		660	475	614	30
Chr9	3600001	3700000		531	361	478	43
Chr9	3700001	3800000		822	567	762	28
Chr9	3800001	3900000		745	515	651	110
Chr9	3900001	4000000		718	430	594	191
Chr9	4000001	4100000		948	443	610	436
Chr9	4100001	4200000		1078	544	597	470
Chr9	4200001	4300000		1752	812	1059	884
Chr9	4300001	4400000		1199	716	630	672
Chr9	4400001	4500000		709	468	597	50
Chr9	4500001	4600000		290	218	272	5
Chr9	4600001	4700000		445	322	309	8
Chr9	4700001	4800000		764	486	657	8
Chr9	4800001	4900000		84	48	77	2
Chr9	4900001	5000000		369	268	345	5
Chr9	5000001	5100000		560	393	532	5
Chr9	5100001	5200000		860	656	794	16
Chr9	5200001	5300000		452	354	347	3
Chr9	5300001	5400000		729	601	640	5
Chr9	5400001	5500000		699	529	612	8
Chr9	5500001	5600000		305	198	273	8
Chr9	5600001	5700000		1187	912	1018	11
Chr9	5700001	5800000		623	516	494	9
Chr9	5800001	5900000		728	470	658	85
Chr9	5900001	6000000		799	477	501	420
Chr9	6000001	6100000		706	442	409	441
Chr9	6100001	6200000		814	329	597	212
Chr9	6200001	6300000		1180	526	811	643
Chr9	6300001	6400000		811	391	491	408
Chr9	6400001	6500000		572	269	311	171
Chr9	6500001	6600000		1038	256	860	398
Chr9	6600001	6700000		870	501	629	438
Chr9	6700001	6800000		1347	683	983	567
Chr9	6800001	6900000		829	611	803	7
Chr9	6900001	7000000		418	324	392	2
Chr9	7000001	7100000		868	353	770	84

					SNP		
				All	Bengal/Nona Bokra	PSSR/Bengal	PSSR/Nona Bokra
Chr9	7100001	7200000		793	356	632	225
Chr9	7200001	7300000		905	479	601	386
Chr9	7300001	7400000		844	475	668	310
Chr9	7400001	7500000		856	399	587	552
Chr9	7500001	7600000		471	129	361	304
Chr9	7600001	7700000		327	191	221	84
Chr9	7700001	7800000		853	588	581	140
Chr9	7800001	7900000		455	285	364	159
Chr9	7900001	8000000		635	375	453	295
Chr9	8000001	8100000		617	344	479	195
Chr9	8100001	8200000		659	448	586	76
Chr9	8200001	8300000		126	69	100	6
Chr9	8300001	8400000		92	50	33	4
Chr9	8400001	8500000		70	35	39	8
Chr9	8500001	8600000		436	320	414	5
Chr9	8600001	8700000		393	262	329	28
Chr9	8700001	8800000		484	396	308	13
Chr9	8800001	8900000		310	208	202	2
Chr9	8900001	9000000		484	201	412	223
Chr9	9000001	9100000		692	481	648	9
Chr9	9100001	9200000		996	669	878	120
Chr9	9200001	9300000		925	623	686	215
Chr9	9300001	9400000		1048	653	759	435
Chr9	9400001	9500000		426	36	393	332
Chr9	9500001	9600000		449	13	411	371
Chr9	9600001	9700000		503	42	446	391
Chr9	9700001	9800000		635	424	219	128
Chr9	9800001	9900000		594	450	425	1
Chr9	9900001	10000000		660	495	627	5
Chr9	1E+07	10100000		424	276	351	57
Chr9	1E+07	10200000		909	625	571	267
Chr9	1E+07	10300000		1061	513	822	261
Chr9	1E+07	10400000		1220	556	603	472
Chr9	1E+07	10500000		886	339	419	71
Chr9	1.1E+07	10600000		495	171	210	59
Chr9	1.1E+07	10700000		90	3	3	2
Chr9	1.1E+07	10800000		863	383	549	345
Chr9	1.1E+07	10900000		853	548	652	129
Chr9	1.1E+07	11000000		406	352	11	356
Chr9	1.1E+07	11100000		549	432	121	421
Chr9	1.1E+07	11200000		702	441	246	449
Chr9	1.1E+07	11300000		911	554	633	367
Chr9	1.1E+07	11400000		635	474	182	191
Chr9	1.1E+07	11500000		699	409	391	201
Chr9	1.2E+07	11600000		425	304	111	266

					SNP		
				All	Bengal/Nona Bokra	PSSR/Bengal	PSSR/Nona Bokra
Chr9	1.2E+07	11700000		606	504	22	505
Chr9	1.2E+07	11800000		777	748	18	753
Chr9	1.2E+07	11900000		616	562	17	571
Chr9	1.2E+07	12000000		517	499	19	496
Chr9	1.2E+07	12100000		796	552	222	566
Chr9	1.2E+07	12200000		571	513	21	504
Chr9	1.2E+07	12300000		605	565	36	574
Chr9	1.2E+07	12400000		860	566	449	533
Chr9	1.2E+07	12500000		1213	580	816	513
Chr9	1.3E+07	12600000		1184	564	822	524
Chr9	1.3E+07	12700000		654	484	273	424
Chr9	1.3E+07	12800000		576	543	19	544
Chr9	1.3E+07	12900000		624	556	202	410
Chr9	1.3E+07	13000000		631	505	536	6
Chr9	1.3E+07	13100000		528	261	312	18
Chr9	1.3E+07	13200000		663	344	426	11
Chr9	1.3E+07	13300000		415	225	274	8
Chr9	1.3E+07	13400000		401	252	301	12
Chr9	1.3E+07	13500000		440	270	320	13
Chr9	1.4E+07	13600000		307	174	160	13
Chr9	1.4E+07	13700000		482	372	327	127
Chr9	1.4E+07	13800000		422	172	23	174
Chr9	1.4E+07	13900000		30	11	13	6
Chr9	1.4E+07	14000000		83	18	21	6
Chr9	1.4E+07	14100000		485	265	315	4
Chr9	1.4E+07	14200000		732	394	503	2
Chr9	1.4E+07	14300000		202	99	122	1
Chr9	1.4E+07	14400000		571	385	436	10
Chr9	1.4E+07	14500000		1304	977	1088	4
Chr9	1.5E+07	14600000		606	388	488	37
Chr9	1.5E+07	14700000		884	496	662	418
Chr9	1.5E+07	14800000		719	446	591	181
Chr9	1.5E+07	14900000		828	584	744	114
Chr9	1.5E+07	15000000		831	586	695	207
Chr9	1.5E+07	15100000		759	583	581	8
Chr9	1.5E+07	15200000		512	327	426	96
Chr9	1.5E+07	15300000		856	559	678	278
Chr9	1.5E+07	15400000		692	427	535	279
Chr9	1.5E+07	15500000		883	488	626	403
Chr9	1.6E+07	15600000		1010	698	523	462
Chr9	1.6E+07	15700000		1104	711	754	511
Chr9	1.6E+07	15800000		738	462	586	261
Chr9	1.6E+07	15900000		913	567	729	309
Chr9	1.6E+07	16000000		954	544	748	351
Chr9	1.6E+07	16100000		575	494	510	4

					SNP		
				All	Bengal/Nona Bokra	PSSR/Bengal	PSSR/Nona Bokra
Chr9	1.6E+07	16200000		794	515	692	73
Chr9	1.6E+07	16300000		825	477	673	256
Chr9	1.6E+07	16400000		703	441	566	191
Chr9	1.6E+07	16500000		731	465	604	233
Chr9	1.7E+07	16600000		672	430	533	247
Chr9	1.7E+07	16700000		765	502	628	194
Chr9	1.7E+07	16800000		711	473	538	258
Chr9	1.7E+07	16900000		698	411	552	272
Chr9	1.7E+07	17000000		699	452	582	194
Chr9	1.7E+07	17100000		442	272	321	157
Chr9	1.7E+07	17200000		610	365	414	284
Chr9	1.7E+07	17300000		851	533	718	226
Chr9	1.7E+07	17400000		814	485	674	262
Chr9	1.7E+07	17500000		715	462	605	165
Chr9	1.8E+07	17600000		894	551	727	329
Chr9	1.8E+07	17700000		735	417	618	200
Chr9	1.8E+07	17800000		847	501	604	277
Chr9	1.8E+07	17900000		719	470	550	219
Chr9	1.8E+07	18000000		771	509	590	261
Chr9	1.8E+07	18100000		653	425	504	248
Chr9	1.8E+07	18200000		619	445	512	126
Chr9	1.8E+07	18300000		165	151	18	154
Chr9	1.8E+07	18400000		56	40	43	19
Chr9	1.8E+07	18500000		238	39	224	175
Chr9	1.9E+07	18600000		525	36	499	434
Chr9	1.9E+07	18700000		693	353	502	357
Chr9	1.9E+07	18800000		825	565	661	256
Chr9	1.9E+07	18900000		784	545	653	260
Chr9	1.9E+07	19000000		845	551	689	278
Chr9	1.9E+07	19100000		671	355	544	306
Chr9	1.9E+07	19200000		941	490	595	490
Chr9	1.9E+07	19300000		904	560	503	614
Chr9	1.9E+07	19400000		716	380	484	308
Chr9	1.9E+07	19500000		780	479	631	192
Chr9	2E+07	19600000		516	355	432	3
Chr9	2E+07	19700000		693	425	551	152
Chr9	2E+07	19800000		654	388	494	228
Chr9	2E+07	19900000		741	520	671	80
Chr9	2E+07	20000000		543	416	494	7
Chr9	2E+07	20100000		431	362	425	1
Chr9	2E+07	20200000		713	554	683	7
Chr9	2E+07	20300000		771	492	648	153
Chr9	2E+07	20400000		750	494	582	257
Chr9	2E+07	20500000		810	523	661	228
Chr9	2.1E+07	20600000		665	459	601	105

					SNP		
				All	Bengal/Nona Bokra	PSSR/Bengal	PSSR/Nona Bokra
Chr9	2.1E+07	20700000		730	116	663	545
Chr9	2.1E+07	20800000		660	452	402	404
Chr9	2.1E+07	20900000		677	243	605	307
Chr9	2.1E+07	21000000		624	358	464	317
Chr9	2.1E+07	21100000		487	444	55	421
Chr9	2.1E+07	21200000		574	337	372	302
Chr9	2.1E+07	21300000		784	112	747	531
Chr9	2.1E+07	21400000		506	157	487	269
Chr9	2.1E+07	21500000		84	24	27	10
Chr9	2.2E+07	21600000		89	68	79	12
Chr9	2.2E+07	21700000		332	273	324	5
Chr9	2.2E+07	21800000		578	493	538	7
Chr9	2.2E+07	21900000		774	628	683	4
Chr9	2.2E+07	22000000		592	444	577	11
Chr9	2.2E+07	22100000		622	488	576	6
Chr9	2.2E+07	22200000		413	323	360	93
Chr9	2.2E+07	22300000		1122	851	895	260
Chr9	2.2E+07	22400000		1017	743	640	406
Chr9	2.2E+07	22500000		473	272	316	136
Chr9	2.3E+07	22600000		551	340	387	268
Chr9	2.3E+07	22700000		326	236	278	88
Chr9	2.3E+07	22800000		493	411	482	5
Chr9	2.3E+07	22900000		756	517	665	8
Chr9	2.3E+07	23000000		224	169	202	1
Chr9	2.3E+07	23100000		0	0	0	0
Chr10	Chr10						
Chr10	1	100000		621	357	470	4
Chr10	100001	200000		973	618	879	98
Chr10	200001	300000		762	262	604	207
Chr10	300001	400000		683	423	545	189
Chr10	400001	500000		1277	658	794	528
Chr10	500001	600000		954	509	564	383
Chr10	600001	700000		972	545	805	238
Chr10	700001	800000		987	601	700	456
Chr10	800001	900000		1161	671	744	319
Chr10	900001	1000000		859	501	697	217
Chr10	1000001	1100000		428	267	309	146
Chr10	1100001	1200000		736	420	402	179
Chr10	1200001	1300000		638	189	246	178
Chr10	1300001	1400000		888	287	382	217
Chr10	1400001	1500000		1199	587	643	375
Chr10	1500001	1600000		984	427	611	223
Chr10	1600001	1700000		670	399	466	290
Chr10	1700001	1800000		802	478	556	350
Chr10	1800001	1900000		798	520	651	237

					SNP		
				All	Bengal/Nona Bokra	PSSR/Bengal	PSSR/Nona Bokra
Chr10	1900001	2000000		557	276	395	319
Chr10	2000001	2100000		1132	810	901	146
Chr10	2100001	2200000		1247	683	766	776
Chr10	2200001	2300000		1504	752	554	377
Chr10	2300001	2400000		1269	342	494	620
Chr10	2400001	2500000		1344	724	620	719
Chr10	2500001	2600000		1115	494	652	444
Chr10	2600001	2700000		859	482	241	490
Chr10	2700001	2800000		1110	406	442	398
Chr10	2800001	2900000		1304	562	722	92
Chr10	2900001	3000000		1726	777	740	18
Chr10	3000001	3100000		1492	916	961	740
Chr10	3100001	3200000		983	516	608	544
Chr10	3200001	3300000		309	90	209	39
Chr10	3300001	3400000		519	315	284	196
Chr10	3400001	3500000		1067	597	468	570
Chr10	3500001	3600000		890	266	424	235
Chr10	3600001	3700000		1391	660	395	345
Chr10	3700001	3800000		1414	642	357	530
Chr10	3800001	3900000		658	367	261	317
Chr10	3900001	4000000		712	434	244	393
Chr10	4000001	4100000		919	550	399	477
Chr10	4100001	4200000		286	18	133	13
Chr10	4200001	4300000		1619	694	532	691
Chr10	4300001	4400000		1111	414	100	403
Chr10	4400001	4500000		1038	366	180	367
Chr10	4500001	4600000		1458	648	383	536
Chr10	4600001	4700000		1059	396	445	184
Chr10	4700001	4800000		1322	640	654	586
Chr10	4800001	4900000		866	264	401	173
Chr10	4900001	5000000		1074	157	194	13
Chr10	5000001	5100000		334	17	39	8
Chr10	5100001	5200000		618	130	160	4
Chr10	5200001	5300000		1253	607	718	40
Chr10	5300001	5400000		792	273	378	214
Chr10	5400001	5500000		1304	535	673	570
Chr10	5500001	5600000		704	273	329	242
Chr10	5600001	5700000		891	564	727	6
Chr10	5700001	5800000		340	245	298	8
Chr10	5800001	5900000		655	270	331	3
Chr10	5900001	6000000		507	81	106	13
Chr10	6000001	6100000		854	126	148	7
Chr10	6100001	6200000		565	395	483	8
Chr10	6200001	6300000		472	239	268	5
Chr10	6300001	6400000		563	305	361	9

					SNP		
				All	Bengal/Nona Bokra	PSSR/Bengal	PSSR/Nona Bokra
Chr10	6400001	6500000		444	221	276	11
Chr10	6500001	6600000		769	374	461	8
Chr10	6600001	6700000		974	353	407	9
Chr10	6700001	6800000		680	166	204	14
Chr10	6800001	6900000		515	238	315	8
Chr10	6900001	7000000		609	318	382	8
Chr10	7000001	7100000		281	151	186	14
Chr10	7100001	7200000		295	148	202	3
Chr10	7200001	7300000		641	384	498	15
Chr10	7300001	7400000		747	433	502	10
Chr10	7400001	7500000		1173	734	887	13
Chr10	7500001	7600000		980	643	745	11
Chr10	7600001	7700000		857	318	391	5
Chr10	7700001	7800000		901	324	388	6
Chr10	7800001	7900000		736	202	249	5
Chr10	7900001	8000000		852	212	278	8
Chr10	8000001	8100000		929	303	352	8
Chr10	8100001	8200000		419	165	212	6
Chr10	8200001	8300000		576	298	322	4
Chr10	8300001	8400000		666	286	361	29
Chr10	8400001	8500000		748	321	388	3
Chr10	8500001	8600000		939	494	641	10
Chr10	8600001	8700000		554	236	286	10
Chr10	8700001	8800000		568	277	337	8
Chr10	8800001	8900000		756	495	579	2
Chr10	8900001	9000000		884	378	453	12
Chr10	9000001	9100000		893	335	395	8
Chr10	9100001	9200000		1113	514	658	9
Chr10	9200001	9300000		616	274	377	3
Chr10	9300001	9400000		1295	582	732	9
Chr10	9400001	9500000		678	294	367	5
Chr10	9500001	9600000		1203	524	654	3
Chr10	9600001	9700000		893	248	280	3
Chr10	9700001	9800000		1171	664	822	18
Chr10	9800001	9900000		744	470	557	14
Chr10	9900001	10000000		766	529	666	8
Chr10	1E+07	10100000		571	304	390	5
Chr10	1E+07	10200000		733	352	456	13
Chr10	1E+07	10300000		746	435	591	11
Chr10	1E+07	10400000		983	547	683	8
Chr10	1E+07	10500000		1134	346	422	9
Chr10	1.1E+07	10600000		882	675	696	5
Chr10	1.1E+07	10700000		942	415	649	57
Chr10	1.1E+07	10800000		1111	432	513	9
Chr10	1.1E+07	10900000		47	0	6	4

					SNP		
				All	Bengal/Nona Bokra	PSSR/Bengal	PSSR/Nona Bokra
Chr10	1.1E+07	11000000		888	244	274	9
Chr10	1.1E+07	11100000		1029	281	418	205
Chr10	1.1E+07	11200000		1326	201	369	287
Chr10	1.1E+07	11300000		966	417	421	110
Chr10	1.1E+07	11400000		523	111	124	5
Chr10	1.1E+07	11500000		276	29	32	2
Chr10	1.2E+07	11600000		961	292	263	7
Chr10	1.2E+07	11700000		1055	577	697	7
Chr10	1.2E+07	11800000		939	497	635	12
Chr10	1.2E+07	11900000		751	556	693	13
Chr10	1.2E+07	12000000		896	612	759	163
Chr10	1.2E+07	12100000		805	528	701	118
Chr10	1.2E+07	12200000		998	592	682	513
Chr10	1.2E+07	12300000		1187	539	884	280
Chr10	1.2E+07	12400000		1133	667	718	540
Chr10	1.2E+07	12500000		1013	650	743	371
Chr10	1.3E+07	12600000		361	205	206	171
Chr10	1.3E+07	12700000		192	149	28	13
Chr10	1.3E+07	12800000		525	305	379	254
Chr10	1.3E+07	12900000		809	420	586	257
Chr10	1.3E+07	13000000		758	595	642	35
Chr10	1.3E+07	13100000		201	151	194	11
Chr10	1.3E+07	13200000		1194	593	552	10
Chr10	1.3E+07	13300000		801	248	320	188
Chr10	1.3E+07	13400000		927	309	291	286
Chr10	1.3E+07	13500000		1096	254	373	218
Chr10	1.4E+07	13600000		960	397	514	322
Chr10	1.4E+07	13700000		1379	411	636	293
Chr10	1.4E+07	13800000		1087	349	409	111
Chr10	1.4E+07	13900000		716	411	508	159
Chr10	1.4E+07	14000000		762	490	577	241
Chr10	1.4E+07	14100000		784	546	583	111
Chr10	1.4E+07	14200000		852	578	615	152
Chr10	1.4E+07	14300000		1358	774	833	547
Chr10	1.4E+07	14400000		611	282	421	370
Chr10	1.4E+07	14500000		654	503	233	468
Chr10	1.5E+07	14600000		866	556	647	355
Chr10	1.5E+07	14700000		1069	669	837	421
Chr10	1.5E+07	14800000		873	642	735	91
Chr10	1.5E+07	14900000		641	451	526	63
Chr10	1.5E+07	15000000		579	433	521	20
Chr10	1.5E+07	15100000		564	389	509	4
Chr10	1.5E+07	15200000		712	488	607	17
Chr10	1.5E+07	15300000		402	308	279	2
Chr10	1.5E+07	15400000		481	343	441	4

					SNP		
				All	Bengal/Nona Bokra	PSSR/Bengal	PSSR/Nona Bokra
Chr10	1.5E+07	15500000		597	414	553	20
Chr10	1.6E+07	15600000		842	595	793	15
Chr10	1.6E+07	15700000		869	540	689	15
Chr10	1.6E+07	15800000		858	400	520	7
Chr10	1.6E+07	15900000		1688	871	994	154
Chr10	1.6E+07	16000000		1317	681	769	111
Chr10	1.6E+07	16100000		1487	784	939	434
Chr10	1.6E+07	16200000		920	474	607	174
Chr10	1.6E+07	16300000		1024	190	249	472
Chr10	1.6E+07	16400000		1079	517	282	336
Chr10	1.6E+07	16500000		994	462	299	223
Chr10	1.7E+07	16600000		922	490	536	217
Chr10	1.7E+07	16700000		1192	868	837	206
Chr10	1.7E+07	16800000		666	356	507	317
Chr10	1.7E+07	16900000		653	312	420	44
Chr10	1.7E+07	17000000		785	263	315	110
Chr10	1.7E+07	17100000		832	333	407	18
Chr10	1.7E+07	17200000		814	352	477	15
Chr10	1.7E+07	17300000		880	509	652	315
Chr10	1.7E+07	17400000		1219	776	542	631
Chr10	1.7E+07	17500000		605	417	466	153
Chr10	1.8E+07	17600000		949	663	881	6
Chr10	1.8E+07	17700000		758	556	705	5
Chr10	1.8E+07	17800000		593	438	566	6
Chr10	1.8E+07	17900000		743	559	707	5
Chr10	1.8E+07	18000000		704	544	645	11
Chr10	1.8E+07	18100000		867	619	795	80
Chr10	1.8E+07	18200000		963	631	769	180
Chr10	1.8E+07	18300000		589	245	443	158
Chr10	1.8E+07	18400000		659	9	388	336
Chr10	1.8E+07	18500000		755	23	211	172
Chr10	1.9E+07	18600000		600	4	8	7
Chr10	1.9E+07	18700000		656	9	7	2
Chr10	1.9E+07	18800000		558	5	5	5
Chr10	1.9E+07	18900000		690	312	172	309
Chr10	1.9E+07	19000000		629	199	162	132
Chr10	1.9E+07	19100000		589	325	6	321
Chr10	1.9E+07	19200000		530	7	6	2
Chr10	1.9E+07	19300000		575	4	4	4
Chr10	1.9E+07	19400000		1054	3	58	49
Chr10	1.9E+07	19500000		647	83	12	79
Chr10	2E+07	19600000		672	34	24	14
Chr10	2E+07	19700000		811	1	131	104
Chr10	2E+07	19800000		556	8	30	25
Chr10	2E+07	19900000		1056	40	106	83

					SNP		
				All	Bengal/Nona Bokra	PSSR/Bengal	PSSR/Nona Bokra
Chr10	2E+07	20000000		774	279	39	254
Chr10	2E+07	20100000		573	259	213	116
Chr10	2E+07	20200000		637	379	478	247
Chr10	2E+07	20300000		999	669	793	220
Chr10	2E+07	20400000		795	654	624	10
Chr10	2E+07	20500000		559	445	454	1
Chr10	2.1E+07	20600000		452	326	383	4
Chr10	2.1E+07	20700000		619	457	566	9
Chr10	2.1E+07	20800000		562	414	549	6
Chr10	2.1E+07	20900000		864	648	812	18
Chr10	2.1E+07	21000000		562	419	518	0
Chr10	2.1E+07	21100000		450	344	404	2
Chr10	2.1E+07	21200000		732	579	698	5
Chr10	2.1E+07	21300000		585	444	557	1
Chr10	2.1E+07	21400000		461	352	436	5
Chr10	2.1E+07	21500000		608	417	494	185
Chr10	2.2E+07	21600000		558	432	539	11
Chr10	2.2E+07	21700000		607	511	563	10
Chr10	2.2E+07	21800000		576	452	520	53
Chr10	2.2E+07	21900000		509	464	62	460
Chr10	2.2E+07	22000000		482	449	9	456
Chr10	2.2E+07	22100000		339	235	109	317
Chr10	2.2E+07	22200000		577	11	565	520
Chr10	2.2E+07	22300000		106	8	97	92
Chr10	2.2E+07	22400000		14	11	9	8
Chr10	2.2E+07	22500000		20	9	13	11
Chr10	2.3E+07	22600000		673	236	548	248
Chr10	2.3E+07	22700000		585	394	498	32
Chr10	2.3E+07	22800000		734	493	555	147
Chr10	2.3E+07	22900000		517	377	463	29
Chr10	2.3E+07	23000000		519	404	477	10
Chr10	2.3E+07	23100000		585	427	512	35
Chr10	2.3E+07	23200000		611	378	442	137
Chr10	2.3E+07	23300000		17	7	13	5
Chr11	Chr11						
Chr11	1	100000		237	82	140	51
Chr11	100001	200000		628	412	571	76
Chr11	200001	300000		553	429	500	6
Chr11	300001	400000		462	334	419	4
Chr11	400001	500000		418	307	396	5
Chr11	500001	600000		213	74	182	82
Chr11	600001	700000		273	242	30	240
Chr11	700001	800000		608	512	318	282
Chr11	800001	900000		564	383	516	7
Chr11	900001	1000000		492	328	425	5

					SNP		
				All	Bengal/Nona Bokra	PSSR/Bengal	PSSR/Nona Bokra
Chr11	1000001	1100000		733	416	712	89
Chr11	1100001	1200000		545	407	471	4
Chr11	1200001	1300000		489	365	462	6
Chr11	1300001	1400000		485	372	459	10
Chr11	1400001	1500000		479	358	452	3
Chr11	1500001	1600000		468	324	435	2
Chr11	1600001	1700000		410	334	383	3
Chr11	1700001	1800000		337	239	317	8
Chr11	1800001	1900000		478	347	425	4
Chr11	1900001	2000000		521	377	490	5
Chr11	2000001	2100000		425	283	411	2
Chr11	2100001	2200000		706	499	604	136
Chr11	2200001	2300000		630	403	478	246
Chr11	2300001	2400000		667	454	536	168
Chr11	2400001	2500000		811	580	756	5
Chr11	2500001	2600000		794	520	585	241
Chr11	2600001	2700000		824	456	630	352
Chr11	2700001	2800000		583	467	444	118
Chr11	2800001	2900000		484	464	13	469
Chr11	2900001	3000000		19	10	14	5
Chr11	3000001	3100000		141	123	7	126
Chr11	3100001	3200000		333	292	23	290
Chr11	3200001	3300000		562	78	39	52
Chr11	3300001	3400000		621	11	5	9
Chr11	3400001	3500000		342	16	2	22
Chr11	3500001	3600000		1024	586	2	584
Chr11	3600001	3700000		1059	632	98	601
Chr11	3700001	3800000		650	409	496	228
Chr11	3800001	3900000		562	397	515	4
Chr11	3900001	4000000		823	519	691	7
Chr11	4000001	4100000		542	366	460	4
Chr11	4100001	4200000		688	449	565	5
Chr11	4200001	4300000		1062	893	780	3
Chr11	4300001	4400000		1017	542	296	228
Chr11	4400001	4500000		839	629	306	575
Chr11	4500001	4600000		633	615	14	611
Chr11	4600001	4700000		274	251	10	252
Chr11	4700001	4800000		567	545	12	516
Chr11	4800001	4900000		764	518	89	473
Chr11	4900001	5000000		1549	494	805	335
Chr11	5000001	5100000		1403	523	632	109
Chr11	5100001	5200000		1094	440	502	174
Chr11	5200001	5300000		327	92	85	39
Chr11	5300001	5400000		636	286	354	83
Chr11	5400001	5500000		745	447	641	180

					SNP		
				All	Bengal/Nona Bokra	PSSR/Bengal	PSSR/Nona Bokra
Chr11	5500001	5600000		784	431	511	405
Chr11	5600001	5700000		857	464	549	374
Chr11	5700001	5800000		1000	446	493	368
Chr11	5800001	5900000		992	758	680	63
Chr11	5900001	6000000		407	288	224	59
Chr11	6000001	6100000		831	609	583	84
Chr11	6100001	6200000		732	316	555	413
Chr11	6200001	6300000		815	460	593	358
Chr11	6300001	6400000		883	477	661	419
Chr11	6400001	6500000		507	270	369	171
Chr11	6500001	6600000		609	297	450	237
Chr11	6600001	6700000		1020	673	315	178
Chr11	6700001	6800000		289	10	51	12
Chr11	6800001	6900000		524	150	142	165
Chr11	6900001	7000000		1258	513	685	516
Chr11	7000001	7100000		1299	560	694	483
Chr11	7100001	7200000		923	333	400	228
Chr11	7200001	7300000		746	325	443	285
Chr11	7300001	7400000		860	303	640	233
Chr11	7400001	7500000		740	488	638	79
Chr11	7500001	7600000		627	452	585	12
Chr11	7600001	7700000		525	390	495	3
Chr11	7700001	7800000		808	559	735	144
Chr11	7800001	7900000		879	601	559	317
Chr11	7900001	8000000		516	350	426	111
Chr11	8000001	8100000		744	457	580	224
Chr11	8100001	8200000		437	351	322	34
Chr11	8200001	8300000		722	457	636	49
Chr11	8300001	8400000		811	424	675	97
Chr11	8400001	8500000		775	488	651	240
Chr11	8500001	8600000		933	644	843	102
Chr11	8600001	8700000		812	579	739	63
Chr11	8700001	8800000		1252	958	1157	25
Chr11	8800001	8900000		719	552	506	17
Chr11	8900001	9000000		488	295	368	159
Chr11	9000001	9100000		1061	704	580	640
Chr11	9100001	9200000		814	497	611	104
Chr11	9200001	9300000		1594	577	603	13
Chr11	9300001	9400000		621	140	182	5
Chr11	9400001	9500000		1016	407	521	11
Chr11	9500001	9600000		616	69	133	55
Chr11	9600001	9700000		229	6	15	49
Chr11	9700001	9800000		658	240	295	39
Chr11	9800001	9900000		904	372	410	10
Chr11	9900001	10000000		538	40	36	9

					SNP		
				All	Bengal/Nona Bokra	PSSR/Bengal	PSSR/Nona Bokra
Chr11	1E+07	10100000		822	4	150	123
Chr11	1E+07	10200000		1578	390	373	573
Chr11	1E+07	10300000		1429	340	643	546
Chr11	1E+07	10400000		1499	383	501	331
Chr11	1E+07	10500000		1308	317	360	317
Chr11	1.1E+07	10600000		1322	518	705	169
Chr11	1.1E+07	10700000		1137	419	474	366
Chr11	1.1E+07	10800000		781	161	264	190
Chr11	1.1E+07	10900000		1101	195	354	309
Chr11	1.1E+07	11000000		1286	344	704	553
Chr11	1.1E+07	11100000		1081	338	358	104
Chr11	1.1E+07	11200000		1092	379	453	19
Chr11	1.1E+07	11300000		593	151	162	11
Chr11	1.1E+07	11400000		769	157	195	6
Chr11	1.1E+07	11500000		985	220	255	15
Chr11	1.2E+07	11600000		1004	226	269	21
Chr11	1.2E+07	11700000		873	246	320	18
Chr11	1.2E+07	11800000		764	264	333	25
Chr11	1.2E+07	11900000		914	285	358	17
Chr11	1.2E+07	12000000		722	212	246	17
Chr11	1.2E+07	12100000		397	76	108	12
Chr11	1.2E+07	12200000		167	27	44	2
Chr11	1.2E+07	12300000		171	34	40	1
Chr11	1.2E+07	12400000		71	3	7	3
Chr11	1.2E+07	12500000		0	0	0	0
Chr11	1.3E+07	12600000		531	118	160	7
Chr11	1.3E+07	12700000		975	242	297	7
Chr11	1.3E+07	12800000		1249	375	463	9
Chr11	1.3E+07	12900000		915	224	308	11
Chr11	1.3E+07	13000000		278	11	22	2
Chr11	1.3E+07	13100000		1342	368	294	24
Chr11	1.3E+07	13200000		1526	437	522	21
Chr11	1.3E+07	13300000		1231	375	482	42
Chr11	1.3E+07	13400000		1310	389	477	22
Chr11	1.3E+07	13500000		1115	354	414	19
Chr11	1.4E+07	13600000		1028	355	409	28
Chr11	1.4E+07	13700000		794	199	265	17
Chr11	1.4E+07	13800000		1011	261	306	20
Chr11	1.4E+07	13900000		1032	192	352	126
Chr11	1.4E+07	14000000		949	8	469	380
Chr11	1.4E+07	14100000		1342	3	748	579
Chr11	1.4E+07	14200000		783	18	500	390
Chr11	1.4E+07	14300000		758	10	291	196
Chr11	1.4E+07	14400000		883	21	363	289
Chr11	1.4E+07	14500000		548	9	118	85

					SNP		
				All	Bengal/Nona Bokra	PSSR/Bengal	PSSR/Nona Bokra
Chr11	1.5E+07	14600000		1003	18	244	200
Chr11	1.5E+07	14700000		1257	12	447	359
Chr11	1.5E+07	14800000		1524	12	572	456
Chr11	1.5E+07	14900000		931	14	374	291
Chr11	1.5E+07	15000000		855	9	247	178
Chr11	1.5E+07	15100000		798	20	163	119
Chr11	1.5E+07	15200000		688	5	233	190
Chr11	1.5E+07	15300000		790	7	248	182
Chr11	1.5E+07	15400000		1510	216	587	334
Chr11	1.5E+07	15500000		1285	471	612	226
Chr11	1.6E+07	15600000		1256	103	495	376
Chr11	1.6E+07	15700000		1309	49	507	373
Chr11	1.6E+07	15800000		1089	233	401	384
Chr11	1.6E+07	15900000		1406	428	858	557
Chr11	1.6E+07	16000000		767	320	265	229
Chr11	1.6E+07	16100000		1345	339	183	293
Chr11	1.6E+07	16200000		1530	628	426	593
Chr11	1.6E+07	16300000		1358	341	709	605
Chr11	1.6E+07	16400000		1137	440	359	373
Chr11	1.6E+07	16500000		1821	934	638	575
Chr11	1.7E+07	16600000		1003	449	683	127
Chr11	1.7E+07	16700000		1141	552	730	21
Chr11	1.7E+07	16800000		1025	679	766	13
Chr11	1.7E+07	16900000		816	279	355	53
Chr11	1.7E+07	17000000		1061	758	944	130
Chr11	1.7E+07	17100000		1042	527	588	246
Chr11	1.7E+07	17200000		1337	434	456	304
Chr11	1.7E+07	17300000		1379	681	412	454
Chr11	1.7E+07	17400000		1476	648	732	497
Chr11	1.7E+07	17500000		1074	472	708	567
Chr11	1.8E+07	17600000		813	234	592	542
Chr11	1.8E+07	17700000		1017	518	655	535
Chr11	1.8E+07	17800000		984	503	374	521
Chr11	1.8E+07	17900000		2035	638	978	535
Chr11	1.8E+07	18000000		1141	656	785	426
Chr11	1.8E+07	18100000		867	479	637	274
Chr11	1.8E+07	18200000		1083	289	541	389
Chr11	1.8E+07	18300000		1320	342	656	560
Chr11	1.8E+07	18400000		1123	380	541	455
Chr11	1.8E+07	18500000		888	436	654	372
Chr11	1.9E+07	18600000		937	147	820	691
Chr11	1.9E+07	18700000		527	109	434	373
Chr11	1.9E+07	18800000		731	450	491	326
Chr11	1.9E+07	18900000		1090	613	786	501
Chr11	1.9E+07	19000000		898	465	633	401

					SNP		
				All	Bengal/Nona Bokra	PSSR/Bengal	PSSR/Nona Bokra
Chr11	1.9E+07	19100000		837	393	352	402
Chr11	1.9E+07	19200000		324	308	10	310
Chr11	1.9E+07	19300000		452	422	24	404
Chr11	1.9E+07	19400000		555	431	535	3
Chr11	1.9E+07	19500000		729	480	677	17
Chr11	2E+07	19600000		559	371	437	10
Chr11	2E+07	19700000		1180	523	533	293
Chr11	2E+07	19800000		1187	361	440	213
Chr11	2E+07	19900000		1333	532	434	326
Chr11	2E+07	20000000		907	504	637	232
Chr11	2E+07	20100000		860	163	471	473
Chr11	2E+07	20200000		696	403	429	244
Chr11	2E+07	20300000		255	33	21	4
Chr11	2E+07	20400000		689	337	487	204
Chr11	2E+07	20500000		430	228	214	183
Chr11	2.1E+07	20600000		859	375	593	499
Chr11	2.1E+07	20700000		614	414	510	142
Chr11	2.1E+07	20800000		558	428	517	10
Chr11	2.1E+07	20900000		744	386	539	279
Chr11	2.1E+07	21000000		1016	495	726	462
Chr11	2.1E+07	21100000		835	470	416	279
Chr11	2.1E+07	21200000		479	216	272	213
Chr11	2.1E+07	21300000		729	490	322	449
Chr11	2.1E+07	21400000		601	340	204	355
Chr11	2.1E+07	21500000		1038	727	835	230
Chr11	2.2E+07	21600000		490	366	329	24
Chr11	2.2E+07	21700000		486	210	176	94
Chr11	2.2E+07	21800000		964	541	722	68
Chr11	2.2E+07	21900000		1217	440	573	3
Chr11	2.2E+07	22000000		1367	725	907	113
Chr11	2.2E+07	22100000		1014	392	678	314
Chr11	2.2E+07	22200000		896	227	380	133
Chr11	2.2E+07	22300000		1027	537	566	209
Chr11	2.2E+07	22400000		578	140	237	139
Chr11	2.2E+07	22500000		1096	649	732	375
Chr11	2.3E+07	22600000		1102	570	508	502
Chr11	2.3E+07	22700000		1150	511	425	206
Chr11	2.3E+07	22800000		954	471	330	127
Chr11	2.3E+07	22900000		875	274	207	168
Chr11	2.3E+07	23000000		1299	640	683	548
Chr11	2.3E+07	23100000		1249	608	566	619
Chr11	2.3E+07	23200000		1139	533	689	385
Chr11	2.3E+07	23300000		1113	642	627	440
Chr11	2.3E+07	23400000		445	196	140	32
Chr11	2.3E+07	23500000		613	357	365	206

					SNP		
				All	Bengal/Nona Bokra	PSSR/Bengal	PSSR/Nona Bokra
Chr11	2.4E+07	23600000		227	164	176	48
Chr11	2.4E+07	23700000		506	285	317	312
Chr11	2.4E+07	23800000		566	297	317	262
Chr11	2.4E+07	23900000		641	290	439	242
Chr11	2.4E+07	24000000		1028	653	468	451
Chr11	2.4E+07	24100000		1055	843	329	273
Chr11	2.4E+07	24200000		887	543	654	261
Chr11	2.4E+07	24300000		916	624	775	154
Chr11	2.4E+07	24400000		754	500	530	273
Chr11	2.4E+07	24500000		977	512	638	529
Chr11	2.5E+07	24600000		237	211	104	5
Chr11	2.5E+07	24700000		627	509	568	11
Chr11	2.5E+07	24800000		838	628	791	4
Chr11	2.5E+07	24900000		753	578	731	7
Chr11	2.5E+07	25000000		662	414	474	297
Chr11	2.5E+07	25100000		665	387	480	286
Chr11	2.5E+07	25200000		890	481	691	389
Chr11	2.5E+07	25300000		683	63	624	543
Chr11	2.5E+07	25400000		460	10	444	360
Chr11	2.5E+07	25500000		498	3	485	386
Chr11	2.6E+07	25600000		853	389	660	419
Chr11	2.6E+07	25700000		690	215	561	199
Chr11	2.6E+07	25800000		652	270	410	263
Chr11	2.6E+07	25900000		807	346	579	368
Chr11	2.6E+07	26000000		335	116	259	172
Chr11	2.6E+07	26100000		652	353	375	338
Chr11	2.6E+07	26200000		429	220	246	247
Chr11	2.6E+07	26300000		669	435	319	374
Chr11	2.6E+07	26400000		386	241	217	250
Chr11	2.6E+07	26500000		530	279	315	275
Chr11	2.7E+07	26600000		701	349	421	344
Chr11	2.7E+07	26700000		196	45	152	5
Chr11	2.7E+07	26800000		240	119	121	75
Chr11	2.7E+07	26900000		481	161	320	106
Chr11	2.7E+07	27000000		614	301	345	266
Chr11	2.7E+07	27100000		464	246	291	244
Chr11	2.7E+07	27200000		640	291	305	294
Chr11	2.7E+07	27300000		739	235	210	204
Chr11	2.7E+07	27400000		734	261	217	157
Chr11	2.7E+07	27500000		947	416	537	311
Chr11	2.8E+07	27600000		570	306	231	68
Chr11	2.8E+07	27700000		846	452	335	274
Chr11	2.8E+07	27800000		1182	514	554	490
Chr11	2.8E+07	27900000		993	262	191	345
Chr11	2.8E+07	28000000		500	280	303	135

					SNP		
				All	Bengal/Nona Bokra	PSSR/Bengal	PSSR/Nona Bokra
Chr11	2.8E+07	28100000		337	101	138	27
Chr11	2.8E+07	28200000		516	145	100	116
Chr11	2.8E+07	28300000		339	19	69	35
Chr11	2.8E+07	28400000		579	10	45	2
Chr11	2.8E+07	28500000		978	298	443	313
Chr11	2.9E+07	28600000		156	5	36	43
Chr11	2.9E+07	28700000		871	472	269	431
Chr11	2.9E+07	28800000		1065	570	366	586
Chr11	2.9E+07	28900000		866	567	688	1
Chr11	2.9E+07	29000000		495	400	456	3
Chr11	2.9E+07	29100000		93	76	83	1
Chr12	Chr12						
Chr12	1	100000		209	26	129	97
Chr12	100001	200000		339	9	323	275
Chr12	200001	300000		579	7	543	488
Chr12	300001	400000		404	14	270	250
Chr12	400001	500000		284	12	168	147
Chr12	500001	600000		287	122	142	5
Chr12	600001	700000		846	469	563	44
Chr12	700001	800000		752	434	536	336
Chr12	800001	900000		648	384	421	306
Chr12	900001	1000000		371	332	141	217
Chr12	1000001	1100000		455	429	18	425
Chr12	1100001	1200000		435	414	26	405
Chr12	1200001	1300000		422	410	14	407
Chr12	1300001	1400000		514	375	319	246
Chr12	1400001	1500000		753	351	535	262
Chr12	1500001	1600000		637	402	532	147
Chr12	1600001	1700000		531	374	468	3
Chr12	1700001	1800000		458	317	421	2
Chr12	1800001	1900000		407	286	382	4
Chr12	1900001	2000000		392	268	362	10
Chr12	2000001	2100000		366	250	299	12
Chr12	2100001	2200000		473	345	424	33
Chr12	2200001	2300000		725	495	590	187
Chr12	2300001	2400000		590	421	523	15
Chr12	2400001	2500000		713	244	297	58
Chr12	2500001	2600000		777	188	11	185
Chr12	2600001	2700000		130	34	86	101
Chr12	2700001	2800000		637	6	618	544
Chr12	2800001	2900000		466	191	251	405
Chr12	2900001	3000000		702	40	416	311
Chr12	3000001	3100000		1128	494	804	640
Chr12	3100001	3200000		944	369	420	348
Chr12	3200001	3300000		673	69	170	220

					SNP		
				All	Bengal/Nona Bokra	PSSR/Bengal	PSSR/Nona Bokra
Chr12	3300001	3400000		754	325	5	327
Chr12	3400001	3500000		916	439	5	429
Chr12	3500001	3600000		768	111	75	129
Chr12	3600001	3700000		775	153	131	47
Chr12	3700001	3800000		929	148	179	256
Chr12	3800001	3900000		722	3	253	218
Chr12	3900001	4000000		692	343	14	323
Chr12	4000001	4100000		467	354	4	266
Chr12	4100001	4200000		740	580	14	495
Chr12	4200001	4300000		779	238	9	203
Chr12	4300001	4400000		129	4	6	2
Chr12	4400001	4500000		1038	83	291	281
Chr12	4500001	4600000		898	10	11	7
Chr12	4600001	4700000		832	444	578	11
Chr12	4700001	4800000		748	535	691	12
Chr12	4800001	4900000		656	440	621	13
Chr12	4900001	5000000		943	664	820	9
Chr12	5000001	5100000		883	677	806	1
Chr12	5100001	5200000		446	366	392	3
Chr12	5200001	5300000		45	27	20	0
Chr12	5300001	5400000		316	267	248	1
Chr12	5400001	5500000		563	418	449	8
Chr12	5500001	5600000		572	423	525	18
Chr12	5600001	5700000		437	285	402	6
Chr12	5700001	5800000		648	487	626	4
Chr12	5800001	5900000		1030	829	880	7
Chr12	5900001	6000000		161	102	88	6
Chr12	6000001	6100000		28	18	14	0
Chr12	6100001	6200000		821	591	733	1
Chr12	6200001	6300000		615	353	578	9
Chr12	6300001	6400000		255	165	218	2
Chr12	6400001	6500000		57	36	33	3
Chr12	6500001	6600000		629	346	557	5
Chr12	6600001	6700000		170	119	66	7
Chr12	6700001	6800000		772	591	682	3
Chr12	6800001	6900000		545	417	504	5
Chr12	6900001	7000000		1001	682	678	281
Chr12	7000001	7100000		505	286	413	154
Chr12	7100001	7200000		827	656	659	4
Chr12	7200001	7300000		843	611	799	7
Chr12	7300001	7400000		696	490	667	9
Chr12	7400001	7500000		723	504	620	1
Chr12	7500001	7600000		694	518	665	8
Chr12	7600001	7700000		596	463	583	0
Chr12	7700001	7800000		749	554	656	1

					SNP		
				All	Bengal/Nona Bokra	PSSR/Bengal	PSSR/Nona Bokra
Chr12	7800001	7900000		1040	901	623	2
Chr12	7900001	8000000		839	612	803	7
Chr12	8000001	8100000		722	552	683	3
Chr12	8100001	8200000		343	227	308	6
Chr12	8200001	8300000		886	650	806	1
Chr12	8300001	8400000		565	419	531	3
Chr12	8400001	8500000		738	576	666	9
Chr12	8500001	8600000		215	38	64	6
Chr12	8600001	8700000		233	175	121	4
Chr12	8700001	8800000		497	395	307	3
Chr12	8800001	8900000		1045	810	907	1
Chr12	8900001	9000000		762	504	708	4
Chr12	9000001	9100000		989	690	952	6
Chr12	9100001	9200000		653	496	634	4
Chr12	9200001	9300000		744	531	707	7
Chr12	9300001	9400000		418	274	384	5
Chr12	9400001	9500000		473	315	427	10
Chr12	9500001	9600000		538	391	513	5
Chr12	9600001	9700000		287	197	264	4
Chr12	9700001	9800000		593	408	544	7
Chr12	9800001	9900000		478	304	437	5
Chr12	9900001	10000000		520	362	414	7
Chr12	1E+07	10100000		655	488	609	4
Chr12	1E+07	10200000		721	529	616	18
Chr12	1E+07	10300000		603	464	572	5
Chr12	1E+07	10400000		547	411	459	0
Chr12	1E+07	10500000		493	352	478	4
Chr12	1.1E+07	10600000		319	222	294	1
Chr12	1.1E+07	10700000		493	367	465	6
Chr12	1.1E+07	10800000		863	669	763	2
Chr12	1.1E+07	10900000		502	359	492	3
Chr12	1.1E+07	11000000		343	253	310	4
Chr12	1.1E+07	11100000		343	244	325	2
Chr12	1.1E+07	11200000		316	204	293	3
Chr12	1.1E+07	11300000		414	318	402	5
Chr12	1.1E+07	11400000		248	182	226	1
Chr12	1.1E+07	11500000		294	198	265	3
Chr12	1.2E+07	11600000		209	112	191	16
Chr12	1.2E+07	11700000		318	208	284	11
Chr12	1.2E+07	11800000		310	249	301	3
Chr12	1.2E+07	11900000		385	270	326	0
Chr12	1.2E+07	12000000		315	193	239	8
Chr12	1.2E+07	12100000		178	109	141	2
Chr12	1.2E+07	12200000		350	257	309	3
Chr12	1.2E+07	12300000		282	205	244	2

					SNP		
				All	Bengal/Nona Bokra	PSSR/Bengal	PSSR/Nona Bokra
Chr12	1.2E+07	12400000		274	233	260	2
Chr12	1.2E+07	12500000		484	360	444	4
Chr12	1.3E+07	12600000		513	395	469	7
Chr12	1.3E+07	12700000		675	509	626	8
Chr12	1.3E+07	12800000		695	500	660	11
Chr12	1.3E+07	12900000		626	407	580	3
Chr12	1.3E+07	13000000		826	564	782	7
Chr12	1.3E+07	13100000		569	418	541	5
Chr12	1.3E+07	13200000		827	638	743	3
Chr12	1.3E+07	13300000		574	419	506	2
Chr12	1.3E+07	13400000		155	28	60	7
Chr12	1.3E+07	13500000		822	566	712	9
Chr12	1.4E+07	13600000		492	352	457	4
Chr12	1.4E+07	13700000		787	607	745	3
Chr12	1.4E+07	13800000		916	710	802	7
Chr12	1.4E+07	13900000		602	419	555	7
Chr12	1.4E+07	14000000		526	372	481	5
Chr12	1.4E+07	14100000		998	699	945	4
Chr12	1.4E+07	14200000		981	654	943	14
Chr12	1.4E+07	14300000		1116	812	1061	11
Chr12	1.4E+07	14400000		175	106	137	2
Chr12	1.4E+07	14500000		956	727	846	1
Chr12	1.5E+07	14600000		811	510	730	4
Chr12	1.5E+07	14700000		564	404	536	6
Chr12	1.5E+07	14800000		1040	760	918	9
Chr12	1.5E+07	14900000		632	506	606	5
Chr12	1.5E+07	15000000		769	565	713	0
Chr12	1.5E+07	15100000		720	551	652	8
Chr12	1.5E+07	15200000		388	306	362	6
Chr12	1.5E+07	15300000		695	498	608	5
Chr12	1.5E+07	15400000		641	496	509	3
Chr12	1.5E+07	15500000		736	557	653	13
Chr12	1.6E+07	15600000		356	216	314	9
Chr12	1.6E+07	15700000		166	84	137	2
Chr12	1.6E+07	15800000		464	332	442	3
Chr12	1.6E+07	15900000		690	470	632	11
Chr12	1.6E+07	16000000		712	499	609	3
Chr12	1.6E+07	16100000		302	240	197	2
Chr12	1.6E+07	16200000		881	758	551	2
Chr12	1.6E+07	16300000		622	539	368	3
Chr12	1.6E+07	16400000		557	413	443	14
Chr12	1.6E+07	16500000		640	513	469	4
Chr12	1.7E+07	16600000		657	467	609	4
Chr12	1.7E+07	16700000		378	265	344	2
Chr12	1.7E+07	16800000		295	243	134	6

					SNP		
				All	Bengal/Nona Bokra	PSSR/Bengal	PSSR/Nona Bokra
Chr12	1.7E+07	16900000		62	42	24	0
Chr12	1.7E+07	17000000		408	309	237	8
Chr12	1.7E+07	17100000		637	405	563	1
Chr12	1.7E+07	17200000		148	84	131	2
Chr12	1.7E+07	17300000		378	257	339	3
Chr12	1.7E+07	17400000		525	405	377	47
Chr12	1.7E+07	17500000		699	361	467	345
Chr12	1.8E+07	17600000		760	563	417	377
Chr12	1.8E+07	17700000		646	407	537	170
Chr12	1.8E+07	17800000		324	206	132	75
Chr12	1.8E+07	17900000		541	461	70	64
Chr12	1.8E+07	18000000		767	402	501	482
Chr12	1.8E+07	18100000		878	450	604	461
Chr12	1.8E+07	18200000		848	576	445	255
Chr12	1.8E+07	18300000		477	300	386	153
Chr12	1.8E+07	18400000		488	339	465	23
Chr12	1.8E+07	18500000		422	276	356	45
Chr12	1.9E+07	18600000		881	732	482	20
Chr12	1.9E+07	18700000		795	672	494	32
Chr12	1.9E+07	18800000		486	374	193	95
Chr12	1.9E+07	18900000		769	525	425	253
Chr12	1.9E+07	19000000		419	299	357	51
Chr12	1.9E+07	19100000		321	239	196	49
Chr12	1.9E+07	19200000		1018	677	654	396
Chr12	1.9E+07	19300000		475	342	362	12
Chr12	1.9E+07	19400000		832	675	604	5
Chr12	1.9E+07	19500000		677	470	629	92
Chr12	2E+07	19600000		726	541	682	7
Chr12	2E+07	19700000		656	503	603	3
Chr12	2E+07	19800000		718	532	565	46
Chr12	2E+07	19900000		857	390	680	342
Chr12	2E+07	20000000		837	453	665	250
Chr12	2E+07	20100000		545	316	441	199
Chr12	2E+07	20200000		312	194	218	20
Chr12	2E+07	20300000		61	45	16	0
Chr12	2E+07	20400000		179	115	89	7
Chr12	2E+07	20500000		86	62	24	3
Chr12	2.1E+07	20600000		37	6	5	4
Chr12	2.1E+07	20700000		62	2	12	9
Chr12	2.1E+07	20800000		491	348	384	90
Chr12	2.1E+07	20900000		1056	730	843	233
Chr12	2.1E+07	21000000		570	421	470	75
Chr12	2.1E+07	21100000		887	621	834	9
Chr12	2.1E+07	21200000		214	157	190	8
Chr12	2.1E+07	21300000		152	145	5	144

					SNP		
				All	Bengal/Nona Bokra	PSSR/Bengal	PSSR/Nona Bokra
Chr12	2.1E+07	21400000		278	256	13	259
Chr12	2.1E+07	21500000		290	277	11	282
Chr12	2.2E+07	21600000		612	564	54	566
Chr12	2.2E+07	21700000		486	442	51	462
Chr12	2.2E+07	21800000		490	331	204	342
Chr12	2.2E+07	21900000		723	471	619	44
Chr12	2.2E+07	22000000		424	276	407	7
Chr12	2.2E+07	22100000		400	245	375	80
Chr12	2.2E+07	22200000		934	627	652	342
Chr12	2.2E+07	22300000		874	513	668	332
Chr12	2.2E+07	22400000		584	336	441	230
Chr12	2.2E+07	22500000		956	774	424	145
Chr12	2.3E+07	22600000		625	325	392	339
Chr12	2.3E+07	22700000		486	348	465	3
Chr12	2.3E+07	22800000		679	500	591	9
Chr12	2.3E+07	22900000		546	429	370	4
Chr12	2.3E+07	23000000		369	260	336	9
Chr12	2.3E+07	23100000		559	394	486	45
Chr12	2.3E+07	23200000		530	300	388	131
Chr12	2.3E+07	23300000		418	319	397	1
Chr12	2.3E+07	23400000		643	490	567	2
Chr12	2.3E+07	23500000		918	570	708	294
Chr12	2.4E+07	23600000		629	409	492	144
Chr12	2.4E+07	23700000		666	343	502	113
Chr12	2.4E+07	23800000		511	218	309	245
Chr12	2.4E+07	23900000		647	275	417	290
Chr12	2.4E+07	24000000		562	384	454	106
Chr12	2.4E+07	24100000		603	414	492	9
Chr12	2.4E+07	24200000		552	422	502	4
Chr12	2.4E+07	24300000		640	507	491	1
Chr12	2.4E+07	24400000		550	441	497	6
Chr12	2.4E+07	24500000		662	420	593	8
Chr12	2.5E+07	24600000		561	379	503	8
Chr12	2.5E+07	24700000		821	349	436	10
Chr12	2.5E+07	24800000		596	8	4	2
Chr12	2.5E+07	24900000		270	10	15	6
Chr12	2.5E+07	25000000		169	13	9	9
Chr12	2.5E+07	25100000		314	4	4	4
Chr12	2.5E+07	25200000		215	70	110	28
Chr12	2.5E+07	25300000		107	17	15	9
Chr12	2.5E+07	25400000		145	89	101	7
Chr12	2.5E+07	25500000		33	17	17	11
Chr12	2.6E+07	25600000		304	13	278	242
Chr12	2.6E+07	25700000		441	13	403	353
Chr12	2.6E+07	25800000		422	61	352	235

					SNP		
				All	Bengal/Nona Bokra	PSSR/Bengal	PSSR/Nona Bokra
Chr12	2.6E+07	25900000		507	206	231	4
Chr12	2.6E+07	26000000		634	484	608	6
Chr12	2.6E+07	26100000		683	530	476	190
Chr12	2.6E+07	26200000		945	908	13	910
Chr12	2.6E+07	26300000		581	547	16	548
Chr12	2.6E+07	26400000		25	13	15	5
Chr12	2.6E+07	26500000		199	15	180	158
Chr12	2.7E+07	26600000		316	9	291	235
Chr12	2.7E+07	26700000		585	11	558	490
Chr12	2.7E+07	26800000		547	14	523	465
Chr12	2.7E+07	26900000		478	29	430	389
Chr12	2.7E+07	27000000		784	4	296	260
Chr12	2.7E+07	27100000		1532	8	319	267
Chr12	2.7E+07	27200000		672	17	527	426
Chr12	2.7E+07	27300000		570	87	515	343
Chr12	2.7E+07	27400000		551	385	471	116
Chr12	2.7E+07	27500000		533	401	497	38
Chr12	2.8E+07	27600000		147	126	146	0
	ChrUn						
	1	100000		476	169	202	12
	100001	200000		385	251	186	124
	200001	300000		70	10	5	20
	300001	400000		284	6	11	215
	400001	500000		163	5	11	107
	500001	600000		370	72	70	137
	ChrSy						
	1	100000		311	153	181	7
	100001	200000		634	294	395	63
	200001	300000		388	138	151	80
	300001	400000		548	127	173	20
	400001	500000		68	14	23	5
	500001	600000		512	165	203	7
	600001	700000		0	0	0	0

**Table S4.** Distribution of InDels in PSRR/Bengal, PSRR/Nona Bokra, and Bengal/Nona Bokra						
on 12 rice chromosomes						
					InDel	
			All	Bengal/Nona Bokra	PSSR/Bengal	PSSR/Nona Bokra
Chr1						
Chr1	1	100000	520	30	3	30
Chr1	100001	200000	417	21	1	19
Chr1	200001	300000	511	39	3	39
Chr1	300001	400000	396	27	0	27
Chr1	400001	500000	901	50	3	51
Chr1	500001	600000	476	41	3	44
Chr1	600001	700000	648	37	35	11
Chr1	700001	800000	1100	22	17	6
Chr1	800001	900000	801	27	22	0
Chr1	900001	1000000	855	12	10	0
Chr1	1000001	1100000	899	28	29	20
Chr1	1100001	1200000	53	1	0	0
Chr1	1200001	1300000	249	17	1	16
Chr1	1300001	1400000	667	39	2	40
Chr1	1400001	1500000	428	18	5	16
Chr1	1500001	1600000	862	28	29	34
Chr1	1600001	1700000	720	34	4	27
Chr1	1700001	1800000	1062	27	1	21
Chr1	1800001	1900000	876	27	2	25
Chr1	1900001	2000000	1175	4	1	3
Chr1	2000001	2100000	583	0	0	0
Chr1	2100001	2200000	1485	66	39	16
Chr1	2200001	2300000	1235	75	13	54
Chr1	2300001	2400000	806	39	23	36
Chr1	2400001	2500000	1026	17	19	12
Chr1	2500001	2600000	1013	34	34	20
Chr1	2600001	2700000	913	31	19	23
Chr1	2700001	2800000	971	42	25	33
Chr1	2800001	2900000	1460	62	50	27
Chr1	2900001	3000000	696	20	21	14
Chr1	3000001	3100000	1005	41	5	23
Chr1	3100001	3200000	805	30	13	18
Chr1	3200001	3300000	1167	21	10	14
Chr1	3300001	3400000	1182	47	14	44
Chr1	3400001	3500000	914	36	19	21
Chr1	3500001	3600000	790	44	19	36
Chr1	3600001	3700000	743	44	41	17
Chr1	3700001	3800000	751	32	32	22
Chr1	3800001	3900000	1494	89	19	73
Chr1	3900001	4000000	834	22	26	15
Chr1	4000001	4100000	976	36	23	22
Chr1	4100001	4200000	804	44	33	12
Chr1	4200001	4300000	849	30	28	13

					InDel	
			All	Bengal/Nona Bokra	PSSR/Bengal	PSSR/Nona Bokra
Chr1	4300001	4400000	747	37	28	13
Chr1	4400001	4500000	905	46	25	10
Chr1	4500001	4600000	882	32	37	11
Chr1	4600001	4700000	829	25	23	16
Chr1	4700001	4800000	617	35	28	13
Chr1	4800001	4900000	797	38	32	8
Chr1	4900001	5000000	1001	43	47	0
Chr1	5000001	5100000	886	43	36	1
Chr1	5100001	5200000	1107	62	58	0
Chr1	5200001	5300000	682	48	41	5
Chr1	5300001	5400000	996	58	38	28
Chr1	5400001	5500000	835	30	38	14
Chr1	5500001	5600000	970	55	43	18
Chr1	5600001	5700000	1005	47	24	29
Chr1	5700001	5800000	554	26	13	21
Chr1	5800001	5900000	68	3	1	5
Chr1	5900001	6000000	913	21	23	29
Chr1	6000001	6100000	1052	27	45	42
Chr1	6100001	6200000	1014	26	42	26
Chr1	6200001	6300000	1091	43	43	0
Chr1	6300001	6400000	1259	34	44	14
Chr1	6400001	6500000	501	26	23	17
Chr1	6500001	6600000	469	15	23	12
Chr1	6600001	6700000	942	27	34	7
Chr1	6700001	6800000	837	22	20	0
Chr1	6800001	6900000	1171	27	26	1
Chr1	6900001	7000000	1011	31	30	0
Chr1	7000001	7100000	1315	39	47	40
Chr1	7100001	7200000	1197	61	29	48
Chr1	7200001	7300000	1057	36	26	27
Chr1	7300001	7400000	1223	50	48	25
Chr1	7400001	7500000	707	48	45	0
Chr1	7500001	7600000	677	51	44	1
Chr1	7600001	7700000	1049	42	31	0
Chr1	7700001	7800000	886	40	35	0
Chr1	7800001	7900000	685	24	19	0
Chr1	7900001	8000000	56	5	2	3
Chr1	8000001	8100000	32	1	0	0
Chr1	8100001	8200000	27	0	0	0
Chr1	8200001	8300000	17	1	0	1
Chr1	8300001	8400000	30	2	2	0
Chr1	8400001	8500000	29	1	1	0
Chr1	8500001	8600000	34	2	3	2
Chr1	8600001	8700000	24	0	0	0
Chr1	8700001	8800000	239	27	0	29

					InDel	
			All	Bengal/Nona Bokra	PSSR/Bengal	PSSR/Nona Bokra
Chr1	8800001	8900000	436	24	0	27
Chr1	8900001	9000000	679	41	8	29
Chr1	9000001	9100000	728	42	39	5
Chr1	9100001	9200000	606	40	13	27
Chr1	9200001	9300000	636	35	16	17
Chr1	9300001	9400000	799	31	21	8
Chr1	9400001	9500000	811	45	43	0
Chr1	9500001	9600000	790	40	40	0
Chr1	9600001	9700000	589	32	31	2
Chr1	9700001	9800000	829	55	54	1
Chr1	9800001	9900000	792	36	30	0
Chr1	9900001	10000000	782	38	30	2
Chr1	10000001	10100000	638	34	26	8
Chr1	10100001	10200000	785	43	38	2
Chr1	10200001	10300000	658	35	33	0
Chr1	10300001	10400000	830	26	29	0
Chr1	10400001	10500000	748	29	24	0
Chr1	10500001	10600000	980	33	34	1
Chr1	10600001	10700000	842	47	33	1
Chr1	10700001	10800000	666	21	18	0
Chr1	10800001	10900000	661	30	24	9
Chr1	10900001	11000000	1016	46	34	19
Chr1	11000001	11100000	895	39	41	6
Chr1	11100001	11200000	1240	31	26	25
Chr1	11200001	11300000	926	39	37	3
Chr1	11300001	11400000	1154	45	44	16
Chr1	11400001	11500000	927	35	36	19
Chr1	11500001	11600000	1074	44	41	13
Chr1	11600001	11700000	799	34	32	1
Chr1	11700001	11800000	1017	52	42	0
Chr1	11800001	11900000	1095	34	26	2
Chr1	11900001	12000000	972	24	25	0
Chr1	12000001	12100000	883	29	27	0
Chr1	12100001	12200000	1062	37	37	1
Chr1	12200001	12300000	800	36	34	2
Chr1	12300001	12400000	810	1	35	31
Chr1	12400001	12500000	469	1	11	5
Chr1	12500001	12600000	847	0	35	30
Chr1	12600001	12700000	579	0	17	12
Chr1	12700001	12800000	686	28	24	0
Chr1	12800001	12900000	810	24	25	1
Chr1	12900001	13000000	616	33	30	1
Chr1	13000001	13100000	338	0	0	0
Chr1	13100001	13200000	1353	15	18	0
Chr1	13200001	13300000	904	26	22	5

					InDel	
			All	Bengal/Nona Bokra	PSSR/Bengal	PSSR/Nona Bokra
Chr1	13300001	13400000	1074	40	32	26
Chr1	13400001	13500000	942	22	11	19
Chr1	13500001	13600000	1021	25	26	27
Chr1	13600001	13700000	123	1	0	0
Chr1	13700001	13800000	1010	34	18	16
Chr1	13800001	13900000	891	36	20	30
Chr1	13900001	14000000	1292	50	41	7
Chr1	14000001	14100000	1212	29	29	9
Chr1	14100001	14200000	983	22	25	11
Chr1	14200001	14300000	1328	38	25	15
Chr1	14300001	14400000	1499	54	41	40
Chr1	14400001	14500000	1017	39	28	18
Chr1	14500001	14600000	870	20	18	10
Chr1	14600001	14700000	888	28	25	5
Chr1	14700001	14800000	1171	48	38	29
Chr1	14800001	14900000	999	47	8	33
Chr1	14900001	15000000	202	2	4	0
Chr1	15000001	15100000	130	2	1	1
Chr1	15100001	15200000	787	42	27	8
Chr1	15200001	15300000	669	26	10	6
Chr1	15300001	15400000	889	28	17	14
Chr1	15400001	15500000	1191	64	41	12
Chr1	15500001	15600000	712	40	34	1
Chr1	15600001	15700000	912	40	40	0
Chr1	15700001	15800000	318	9	9	0
Chr1	15800001	15900000	1255	53	46	0
Chr1	15900001	16000000	1031	38	32	0
Chr1	16000001	16100000	783	38	28	1
Chr1	16100001	16200000	965	27	28	0
Chr1	16200001	16300000	960	23	20	0
Chr1	16300001	16400000	871	41	42	1
Chr1	16400001	16500000	1081	17	21	1
Chr1	16500001	16600000	795	23	19	1
Chr1	16600001	16700000	685	13	14	0
Chr1	16700001	16800000	553	12	13	0
Chr1	16800001	16900000	209	1	2	0
Chr1	16900001	17000000	279	4	3	0
Chr1	17000001	17100000	876	12	11	0
Chr1	17100001	17200000	1067	13	17	0
Chr1	17200001	17300000	879	28	18	0
Chr1	17300001	17400000	727	39	30	1
Chr1	17400001	17500000	802	35	29	1
Chr1	17500001	17600000	847	26	18	1
Chr1	17600001	17700000	843	38	36	0
Chr1	17700001	17800000	916	37	36	0

					InDel	
			All	Bengal/Nona Bokra	PSSR/Bengal	PSSR/Nona Bokra
Chr1	17800001	17900000	1467	27	32	1
Chr1	17900001	18000000	877	48	48	9
Chr1	18000001	18100000	1057	42	37	23
Chr1	18100001	18200000	635	22	21	4
Chr1	18200001	18300000	949	22	29	0
Chr1	18300001	18400000	704	34	30	2
Chr1	18400001	18500000	857	42	37	5
Chr1	18500001	18600000	501	31	22	0
Chr1	18600001	18700000	752	33	32	1
Chr1	18700001	18800000	1288	22	27	18
Chr1	18800001	18900000	1025	27	25	14
Chr1	18900001	19000000	906	26	22	24
Chr1	19000001	19100000	1205	53	47	1
Chr1	19100001	19200000	830	31	33	0
Chr1	19200001	19300000	599	20	18	0
Chr1	19300001	19400000	913	28	32	9
Chr1	19400001	19500000	666	1	20	19
Chr1	19500001	19600000	408	0	2	2
Chr1	19600001	19700000	335	0	3	3
Chr1	19700001	19800000	117	0	1	1
Chr1	19800001	19900000	49	0	0	0
Chr1	19900001	20000000	481	2	12	12
Chr1	20000001	20100000	714	39	6	37
Chr1	20100001	20200000	652	35	0	35
Chr1	20200001	20300000	1096	49	0	50
Chr1	20300001	20400000	507	38	0	37
Chr1	20400001	20500000	541	30	2	30
Chr1	20500001	20600000	485	42	2	42
Chr1	20600001	20700000	595	30	1	30
Chr1	20700001	20800000	212	7	0	7
Chr1	20800001	20900000	116	3	0	3
Chr1	20900001	21000000	507	30	0	31
Chr1	21000001	21100000	228	16	0	16
Chr1	21100001	21200000	62	3	5	1
Chr1	21200001	21300000	282	25	17	0
Chr1	21300001	21400000	583	28	23	0
Chr1	21400001	21500000	705	27	5	0
Chr1	21500001	21600000	130	6	1	0
Chr1	21600001	21700000	1213	82	35	36
Chr1	21700001	21800000	1134	33	29	26
Chr1	21800001	21900000	925	36	19	11
Chr1	21900001	22000000	864	33	22	25
Chr1	22000001	22100000	1103	32	22	23
Chr1	22100001	22200000	746	20	33	11
Chr1	22200001	22300000	557	21	21	0

					InDel	
			All	Bengal/Nona Bokra	PSSR/Bengal	PSSR/Nona Bokra
Chr1	22300001	22400000	794	45	28	9
Chr1	22400001	22500000	1526	70	56	36
Chr1	22500001	22600000	788	35	26	0
Chr1	22600001	22700000	810	33	33	6
Chr1	22700001	22800000	848	39	7	36
Chr1	22800001	22900000	1214	37	38	33
Chr1	22900001	23000000	1181	28	32	15
Chr1	23000001	23100000	701	11	15	9
Chr1	23100001	23200000	923	27	17	17
Chr1	23200001	23300000	1073	32	31	21
Chr1	23300001	23400000	1209	27	34	34
Chr1	23400001	23500000	1634	34	40	20
Chr1	23500001	23600000	1187	23	20	9
Chr1	23600001	23700000	1454	29	36	21
Chr1	23700001	23800000	1161	51	35	30
Chr1	23800001	23900000	1047	53	47	27
Chr1	23900001	24000000	709	39	40	4
Chr1	24000001	24100000	1109	55	48	0
Chr1	24100001	24200000	727	33	27	11
Chr1	24200001	24300000	753	39	32	1
Chr1	24300001	24400000	624	35	35	1
Chr1	24400001	24500000	813	50	47	0
Chr1	24500001	24600000	748	35	29	0
Chr1	24600001	24700000	1155	45	49	1
Chr1	24700001	24800000	1093	54	41	27
Chr1	24800001	24900000	945	28	23	21
Chr1	24900001	25000000	1139	16	32	13
Chr1	25000001	25100000	1039	25	25	20
Chr1	25100001	25200000	886	21	22	1
Chr1	25200001	25300000	1211	31	39	15
Chr1	25300001	25400000	1071	37	26	30
Chr1	25400001	25500000	764	33	20	32
Chr1	25500001	25600000	283	14	11	9
Chr1	25600001	25700000	744	27	30	27
Chr1	25700001	25800000	96	2	1	1
Chr1	25800001	25900000	224	5	3	0
Chr1	25900001	26000000	284	1	1	2
Chr1	26000001	26100000	925	1	17	15
Chr1	26100001	26200000	801	0	36	28
Chr1	26200001	26300000	960	1	14	9
Chr1	26300001	26400000	987	0	35	32
Chr1	26400001	26500000	940	22	27	34
Chr1	26500001	26600000	446	1	15	13
Chr1	26600001	26700000	647	26	27	0
Chr1	26700001	26800000	847	23	24	2

					InDel	
			All	Bengal/Nona Bokra	PSSR/Bengal	PSSR/Nona Bokra
Chr1	26800001	26900000	770	31	31	1
Chr1	26900001	27000000	499	7	8	0
Chr1	27000001	27100000	838	40	28	4
Chr1	27100001	27200000	567	16	1	16
Chr1	27200001	27300000	597	24	24	1
Chr1	27300001	27400000	423	24	22	0
Chr1	27400001	27500000	362	17	15	0
Chr1	27500001	27600000	818	49	44	0
Chr1	27600001	27700000	830	46	42	8
Chr1	27700001	27800000	805	38	34	1
Chr1	27800001	27900000	936	32	29	0
Chr1	27900001	28000000	697	5	3	1
Chr1	28000001	28100000	592	2	1	1
Chr1	28100001	28200000	824	4	8	9
Chr1	28200001	28300000	809	0	17	20
Chr1	28300001	28400000	1054	20	21	19
Chr1	28400001	28500000	933	49	46	3
Chr1	28500001	28600000	1165	23	24	1
Chr1	28600001	28700000	809	47	49	3
Chr1	28700001	28800000	484	23	20	0
Chr1	28800001	28900000	710	36	28	0
Chr1	28900001	29000000	925	52	45	2
Chr1	29000001	29100000	434	23	20	0
Chr1	29100001	29200000	769	34	31	0
Chr1	29200001	29300000	760	31	31	0
Chr1	29300001	29400000	609	41	34	0
Chr1	29400001	29500000	713	50	43	2
Chr1	29500001	29600000	1045	75	61	1
Chr1	29600001	29700000	602	26	23	0
Chr1	29700001	29800000	418	29	24	1
Chr1	29800001	29900000	784	48	42	0
Chr1	29900001	30000000	957	54	53	0
Chr1	30000001	30100000	679	41	35	0
Chr1	30100001	30200000	589	40	32	0
Chr1	30200001	30300000	736	39	29	0
Chr1	30300001	30400000	631	33	34	0
Chr1	30400001	30500000	633	38	32	0
Chr1	30500001	30600000	675	33	36	0
Chr1	30600001	30700000	867	58	49	2
Chr1	30700001	30800000	1469	85	53	1
Chr1	30800001	30900000	984	49	44	7
Chr1	30900001	31000000	889	44	37	6
Chr1	31000001	31100000	824	40	36	1
Chr1	31100001	31200000	453	22	23	0
Chr1	31200001	31300000	867	35	30	3

					InDel	
			All	Bengal/Nona Bokra	PSSR/Bengal	PSSR/Nona Bokra
Chr1	31300001	31400000	872	36	27	13
Chr1	31400001	31500000	646	29	21	11
Chr1	31500001	31600000	845	35	30	6
Chr1	31600001	31700000	954	36	33	10
Chr1	31700001	31800000	842	32	28	0
Chr1	31800001	31900000	846	18	20	1
Chr1	31900001	32000000	719	0	0	0
Chr1	32000001	32100000	646	4	4	0
Chr1	32100001	32200000	773	3	3	4
Chr1	32200001	32300000	979	40	28	15
Chr1	32300001	32400000	902	23	18	8
Chr1	32400001	32500000	815	21	22	0
Chr1	32500001	32600000	784	7	7	0
Chr1	32600001	32700000	574	20	17	0
Chr1	32700001	32800000	460	29	22	1
Chr1	32800001	32900000	712	38	34	1
Chr1	32900001	33000000	554	40	35	0
Chr1	33000001	33100000	662	22	19	0
Chr1	33100001	33200000	573	27	22	0
Chr1	33200001	33300000	933	51	39	0
Chr1	33300001	33400000	653	46	35	0
Chr1	33400001	33500000	1068	45	32	0
Chr1	33500001	33600000	246	12	12	0
Chr1	33600001	33700000	517	28	23	1
Chr1	33700001	33800000	980	57	55	1
Chr1	33800001	33900000	497	28	24	0
Chr1	33900001	34000000	581	25	26	2
Chr1	34000001	34100000	552	21	24	0
Chr1	34100001	34200000	586	33	32	0
Chr1	34200001	34300000	879	41	40	0
Chr1	34300001	34400000	798	42	39	0
Chr1	34400001	34500000	962	67	58	1
Chr1	34500001	34600000	847	48	37	3
Chr1	34600001	34700000	776	36	26	24
Chr1	34700001	34800000	776	28	25	8
Chr1	34800001	34900000	791	42	36	9
Chr1	34900001	35000000	587	28	24	1
Chr1	35000001	35100000	700	35	31	8
Chr1	35100001	35200000	1071	42	40	13
Chr1	35200001	35300000	893	18	19	9
Chr1	35300001	35400000	769	37	31	17
Chr1	35400001	35500000	613	25	21	12
Chr1	35500001	35600000	694	37	30	17
Chr1	35600001	35700000	678	46	30	9
Chr1	35700001	35800000	664	36	29	8

					InDel	
			All	Bengal/Nona Bokra	PSSR/Bengal	PSSR/Nona Bokra
Chr1	35800001	35900000	590	31	25	5
Chr1	35900001	36000000	797	15	29	17
Chr1	36000001	36100000	807	30	21	18
Chr1	36100001	36200000	741	33	26	16
Chr1	36200001	36300000	892	39	33	11
Chr1	36300001	36400000	644	27	22	11
Chr1	36400001	36500000	861	36	32	12
Chr1	36500001	36600000	454	19	24	5
Chr1	36600001	36700000	693	33	25	14
Chr1	36700001	36800000	724	39	39	9
Chr1	36800001	36900000	449	22	19	4
Chr1	36900001	37000000	700	24	26	7
Chr1	37000001	37100000	686	27	21	3
Chr1	37100001	37200000	793	48	37	20
Chr1	37200001	37300000	655	22	17	7
Chr1	37300001	37400000	688	27	26	7
Chr1	37400001	37500000	817	13	2	10
Chr1	37500001	37600000	723	25	0	22
Chr1	37600001	37700000	757	15	6	15
Chr1	37700001	37800000	1062	12	18	17
Chr1	37800001	37900000	828	19	0	18
Chr1	37900001	38000000	707	5	2	4
Chr1	38000001	38100000	728	2	1	1
Chr1	38100001	38200000	928	17	1	16
Chr1	38200001	38300000	894	16	0	17
Chr1	38300001	38400000	850	14	4	16
Chr1	38400001	38500000	689	6	0	7
Chr1	38500001	38600000	731	24	0	24
Chr1	38600001	38700000	671	20	0	21
Chr1	38700001	38800000	1123	24	0	22
Chr1	38800001	38900000	789	3	0	2
Chr1	38900001	39000000	685	0	0	0
Chr1	39000001	39100000	688	2	0	1
Chr1	39100001	39200000	710	1	0	1
Chr1	39200001	39300000	720	6	0	5
Chr1	39300001	39400000	788	0	0	0
Chr1	39400001	39500000	535	0	0	0
Chr1	39500001	39600000	811	20	0	19
Chr1	39600001	39700000	682	4	0	4
Chr1	39700001	39800000	632	9	0	8
Chr1	39800001	39900000	699	1	0	1
Chr1	39900001	40000000	598	3	3	1
Chr1	40000001	40100000	822	2	2	0
Chr1	40100001	40200000	685	1	1	0
Chr1	40200001	40300000	736	2	0	2

					InDel	
			All	Bengal/Nona Bokra	PSSR/Bengal	PSSR/Nona Bokra
Chr1	40300001	40400000	921	23	4	26
Chr1	40400001	40500000	976	41	6	36
Chr1	40500001	40600000	1125	16	0	14
Chr1	40600001	40700000	981	35	0	32
Chr1	40700001	40800000	782	30	1	29
Chr1	40800001	40900000	874	9	6	9
Chr1	40900001	41000000	908	7	24	28
Chr1	41000001	41100000	820	40	23	17
Chr1	41100001	41200000	988	32	27	0
Chr1	41200001	41300000	968	9	36	22
Chr1	41300001	41400000	914	6	21	16
Chr1	41400001	41500000	118	0	4	5
Chr1	41500001	41600000	836	3	30	28
Chr1	41600001	41700000	304	4	2	2
Chr1	41700001	41800000	979	41	36	15
Chr1	41800001	41900000	847	7	33	21
Chr1	41900001	42000000	744	21	42	42
Chr1	42000001	42100000	998	13	58	43
Chr1	42100001	42200000	506	11	28	26
Chr1	42200001	42300000	685	10	17	16
Chr1	42300001	42400000	957	58	64	3
Chr1	42400001	42500000	740	35	38	0
Chr1	42500001	42600000	609	26	25	0
Chr1	42600001	42700000	646	20	25	4
Chr1	42700001	42800000	780	18	22	22
Chr1	42800001	42900000	426	4	18	14
Chr1	42900001	43000000	700	29	12	23
Chr1	43000001	43100000	576	2	32	24
Chr1	43100001	43200000	1165	62	72	0
Chr1	43200001	43300000	434	22	23	0
Chr2	Chr2					
Chr2	1	100000	487	29	1	29
Chr2	100001	200000	455	28	1	27
Chr2	200001	300000	427	32	1	33
Chr2	300001	400000	225	14	0	14
Chr2	400001	500000	358	11	9	5
Chr2	500001	600000	531	24	15	24
Chr2	600001	700000	535	26	13	14
Chr2	700001	800000	563	24	25	9
Chr2	800001	900000	589	40	33	0
Chr2	900001	1000000	746	48	23	49
Chr2	1000001	1100000	686	35	23	16
Chr2	1100001	1200000	535	38	3	35
Chr2	1200001	1300000	518	50	2	51
Chr2	1300001	1400000	642	41	34	13

					InDel	
			All	Bengal/Nona Bokra	PSSR/Bengal	PSSR/Nona Bokra
Chr2	1400001	1500000	758	33	36	21
Chr2	1500001	1600000	578	0	11	9
Chr2	1600001	1700000	38	4	5	1
Chr2	1700001	1800000	29	2	2	0
Chr2	1800001	1900000	24	0	0	0
Chr2	1900001	2000000	17	1	0	1
Chr2	2000001	2100000	541	33	16	29
Chr2	2100001	2200000	1031	47	38	28
Chr2	2200001	2300000	841	18	28	8
Chr2	2300001	2400000	827	18	20	16
Chr2	2400001	2500000	605	2	13	9
Chr2	2500001	2600000	360	3	7	5
Chr2	2600001	2700000	359	11	17	14
Chr2	2700001	2800000	501	24	25	18
Chr2	2800001	2900000	437	29	7	31
Chr2	2900001	3000000	1408	44	5	22
Chr2	3000001	3100000	1043	22	5	23
Chr2	3100001	3200000	414	26	4	27
Chr2	3200001	3300000	609	12	15	22
Chr2	3300001	3400000	503	1	0	2
Chr2	3400001	3500000	548	1	0	1
Chr2	3500001	3600000	51	0	1	1
Chr2	3600001	3700000	94	1	3	0
Chr2	3700001	3800000	209	2	1	1
Chr2	3800001	3900000	561	2	15	13
Chr2	3900001	4000000	826	1	29	19
Chr2	4000001	4100000	603	3	17	17
Chr2	4100001	4200000	251	21	16	2
Chr2	4200001	4300000	772	40	37	1
Chr2	4300001	4400000	1075	28	29	19
Chr2	4400001	4500000	823	17	15	8
Chr2	4500001	4600000	1185	35	30	24
Chr2	4600001	4700000	630	11	3	8
Chr2	4700001	4800000	1142	8	16	23
Chr2	4800001	4900000	1228	45	27	34
Chr2	4900001	5000000	541	42	0	42
Chr2	5000001	5100000	642	41	8	33
Chr2	5100001	5200000	830	41	26	20
Chr2	5200001	5300000	757	36	30	16
Chr2	5300001	5400000	682	26	23	15
Chr2	5400001	5500000	784	37	30	14
Chr2	5500001	5600000	898	52	38	17
Chr2	5600001	5700000	1005	52	37	27
Chr2	5700001	5800000	836	32	29	21
Chr2	5800001	5900000	879	48	42	14

					InDel	
			All	Bengal/Nona Bokra	PSSR/Bengal	PSSR/Nona Bokra
Chr2	5900001	6000000	977	46	38	14
Chr2	6000001	6100000	850	53	43	14
Chr2	6100001	6200000	956	59	45	11
Chr2	6200001	6300000	1295	45	28	9
Chr2	6300001	6400000	531	34	22	0
Chr2	6400001	6500000	739	38	34	0
Chr2	6500001	6600000	322	4	10	4
Chr2	6600001	6700000	749	43	25	0
Chr2	6700001	6800000	1201	71	50	16
Chr2	6800001	6900000	1524	69	70	3
Chr2	6900001	7000000	2233	115	126	0
Chr2	7000001	7100000	1074	33	37	0
Chr2	7100001	7200000	1247	51	50	3
Chr2	7200001	7300000	1190	40	48	3
Chr2	7300001	7400000	1252	36	46	18
Chr2	7400001	7500000	1241	48	36	25
Chr2	7500001	7600000	960	45	35	0
Chr2	7600001	7700000	705	35	29	1
Chr2	7700001	7800000	934	40	37	2
Chr2	7800001	7900000	928	18	17	0
Chr2	7900001	8000000	1231	71	43	3
Chr2	8000001	8100000	908	4	24	21
Chr2	8100001	8200000	1060	4	45	40
Chr2	8200001	8300000	1813	66	77	26
Chr2	8300001	8400000	1195	31	46	25
Chr2	8400001	8500000	2417	89	135	62
Chr2	8500001	8600000	307	12	8	11
Chr2	8600001	8700000	346	9	7	0
Chr2	8700001	8800000	462	22	25	15
Chr2	8800001	8900000	851	3	25	22
Chr2	8900001	9000000	1074	43	46	2
Chr2	9000001	9100000	709	30	26	2
Chr2	9100001	9200000	468	27	11	0
Chr2	9200001	9300000	314	11	8	0
Chr2	9300001	9400000	137	5	6	0
Chr2	9400001	9500000	848	46	48	1
Chr2	9500001	9600000	506	24	22	1
Chr2	9600001	9700000	34	1	1	0
Chr2	9700001	9800000	48	1	1	0
Chr2	9800001	9900000	276	9	0	10
Chr2	9900001	10000000	1413	31	37	37
Chr2	10000001	10100000	1195	24	25	25
Chr2	10100001	10200000	1124	28	14	24
Chr2	10200001	10300000	913	31	20	13
Chr2	10300001	10400000	1229	41	35	47

					InDel	
			All	Bengal/Nona Bokra	PSSR/Bengal	PSSR/Nona Bokra
Chr2	10400001	10500000	793	25	12	13
Chr2	10500001	10600000	1308	38	17	19
Chr2	10600001	10700000	1299	50	48	15
Chr2	10700001	10800000	1093	41	34	24
Chr2	10800001	10900000	1275	29	25	33
Chr2	10900001	11000000	1380	36	31	30
Chr2	11000001	11100000	1543	27	42	28
Chr2	11100001	11200000	1028	49	35	26
Chr2	11200001	11300000	1028	29	23	11
Chr2	11300001	11400000	829	30	28	4
Chr2	11400001	11500000	1083	37	32	13
Chr2	11500001	11600000	840	43	41	5
Chr2	11600001	11700000	890	30	27	0
Chr2	11700001	11800000	646	23	17	0
Chr2	11800001	11900000	399	20	12	0
Chr2	11900001	12000000	148	3	3	0
Chr2	12000001	12100000	780	32	31	1
Chr2	12100001	12200000	1012	31	35	0
Chr2	12200001	12300000	1017	34	28	0
Chr2	12300001	12400000	638	34	33	0
Chr2	12400001	12500000	947	36	47	1
Chr2	12500001	12600000	931	39	27	1
Chr2	12600001	12700000	821	30	26	2
Chr2	12700001	12800000	1095	44	24	0
Chr2	12800001	12900000	743	31	26	0
Chr2	12900001	13000000	712	24	18	0
Chr2	13000001	13100000	699	29	27	0
Chr2	13100001	13200000	591	22	19	2
Chr2	13200001	13300000	661	13	14	0
Chr2	13300001	13400000	564	23	15	0
Chr2	13400001	13500000	667	21	20	0
Chr2	13500001	13600000	823	14	10	0
Chr2	13600001	13700000	312	4	3	0
Chr2	13700001	13800000	268	6	2	0
Chr2	13800001	13900000	631	26	24	2
Chr2	13900001	14000000	737	31	21	2
Chr2	14000001	14100000	648	16	14	0
Chr2	14100001	14200000	731	27	29	0
Chr2	14200001	14300000	110	0	0	0
Chr2	14300001	14400000	864	27	25	0
Chr2	14400001	14500000	874	21	20	0
Chr2	14500001	14600000	802	21	17	0
Chr2	14600001	14700000	873	27	16	1
Chr2	14700001	14800000	918	13	10	1
Chr2	14800001	14900000	834	25	21	0

					InDel	
			All	Bengal/Nona Bokra	PSSR/Bengal	PSSR/Nona Bokra
Chr2	14900001	15000000	533	22	26	0
Chr2	15000001	15100000	826	18	19	0
Chr2	15100001	15200000	632	22	18	1
Chr2	15200001	15300000	1035	19	19	1
Chr2	15300001	15400000	899	36	32	0
Chr2	15400001	15500000	1003	39	36	1
Chr2	15500001	15600000	496	27	22	0
Chr2	15600001	15700000	728	22	20	15
Chr2	15700001	15800000	832	37	25	18
Chr2	15800001	15900000	682	29	29	2
Chr2	15900001	16000000	509	22	18	1
Chr2	16000001	16100000	894	31	26	3
Chr2	16100001	16200000	647	35	31	0
Chr2	16200001	16300000	639	41	40	0
Chr2	16300001	16400000	923	28	21	0
Chr2	16400001	16500000	1023	15	1	12
Chr2	16500001	16600000	974	28	0	24
Chr2	16600001	16700000	1515	21	20	12
Chr2	16700001	16800000	1542	10	11	0
Chr2	16800001	16900000	1709	11	14	6
Chr2	16900001	17000000	1059	22	17	12
Chr2	17000001	17100000	1334	20	17	15
Chr2	17100001	17200000	987	33	22	21
Chr2	17200001	17300000	1040	40	31	17
Chr2	17300001	17400000	714	22	14	12
Chr2	17400001	17500000	843	28	23	11
Chr2	17500001	17600000	936	29	25	23
Chr2	17600001	17700000	403	13	9	3
Chr2	17700001	17800000	444	15	11	0
Chr2	17800001	17900000	990	14	14	20
Chr2	17900001	18000000	1166	30	28	27
Chr2	18000001	18100000	1347	27	36	1
Chr2	18100001	18200000	905	16	17	0
Chr2	18200001	18300000	1658	16	13	2
Chr2	18300001	18400000	1044	25	17	22
Chr2	18400001	18500000	1436	27	17	18
Chr2	18500001	18600000	1440	38	31	17
Chr2	18600001	18700000	993	44	45	11
Chr2	18700001	18800000	911	42	37	1
Chr2	18800001	18900000	984	29	18	11
Chr2	18900001	19000000	913	44	23	23
Chr2	19000001	19100000	739	29	12	28
Chr2	19100001	19200000	1298	50	42	25
Chr2	19200001	19300000	1252	50	57	5
Chr2	19300001	19400000	1085	28	21	10

					InDel	
			All	Bengal/Nona Bokra	PSSR/Bengal	PSSR/Nona Bokra
Chr2	19400001	19500000	914	29	27	1
Chr2	19500001	19600000	330	22	22	0
Chr2	19600001	19700000	914	56	48	0
Chr2	19700001	19800000	996	37	36	0
Chr2	19800001	19900000	866	39	30	1
Chr2	19900001	20000000	931	55	56	3
Chr2	20000001	20100000	954	65	46	2
Chr2	20100001	20200000	1152	79	59	1
Chr2	20200001	20300000	709	29	24	13
Chr2	20300001	20400000	1034	27	17	9
Chr2	20400001	20500000	834	49	25	2
Chr2	20500001	20600000	1263	34	29	21
Chr2	20600001	20700000	842	31	24	6
Chr2	20700001	20800000	835	41	26	18
Chr2	20800001	20900000	785	38	32	13
Chr2	20900001	21000000	806	31	21	8
Chr2	21000001	21100000	1013	61	55	0
Chr2	21100001	21200000	713	56	47	6
Chr2	21200001	21300000	561	23	15	0
Chr2	21300001	21400000	570	17	14	14
Chr2	21400001	21500000	596	25	28	0
Chr2	21500001	21600000	823	48	50	0
Chr2	21600001	21700000	900	48	31	0
Chr2	21700001	21800000	870	51	42	0
Chr2	21800001	21900000	1164	57	46	0
Chr2	21900001	22000000	1108	40	36	14
Chr2	22000001	22100000	995	48	42	1
Chr2	22100001	22200000	974	19	21	13
Chr2	22200001	22300000	968	34	26	16
Chr2	22300001	22400000	786	42	37	3
Chr2	22400001	22500000	800	50	38	1
Chr2	22500001	22600000	920	80	56	0
Chr2	22600001	22700000	795	44	42	2
Chr2	22700001	22800000	578	36	33	1
Chr2	22800001	22900000	572	40	34	0
Chr2	22900001	23000000	22	1	1	0
Chr2	23000001	23100000	665	38	37	0
Chr2	23100001	23200000	627	21	20	6
Chr2	23200001	23300000	465	14	11	11
Chr2	23300001	23400000	181	7	5	6
Chr2	23400001	23500000	591	26	9	22
Chr2	23500001	23600000	887	61	32	40
Chr2	23600001	23700000	698	21	26	17
Chr2	23700001	23800000	179	0	5	3
Chr2	23800001	23900000	385	4	15	13

					InDel	
			All	Bengal/Nona Bokra	PSSR/Bengal	PSSR/Nona Bokra
Chr2	23900001	24000000	736	19	37	18
Chr2	24000001	24100000	716	40	34	0
Chr2	24100001	24200000	949	32	35	22
Chr2	24200001	24300000	1360	49	19	47
Chr2	24300001	24400000	1215	20	6	7
Chr2	24400001	24500000	796	22	9	9
Chr2	24500001	24600000	903	32	26	18
Chr2	24600001	24700000	1373	31	23	30
Chr2	24700001	24800000	1125	24	28	25
Chr2	24800001	24900000	818	24	28	21
Chr2	24900001	25000000	874	5	37	37
Chr2	25000001	25100000	885	6	12	9
Chr2	25100001	25200000	675	5	27	20
Chr2	25200001	25300000	1079	31	10	28
Chr2	25300001	25400000	1418	41	43	0
Chr2	25400001	25500000	860	38	30	0
Chr2	25500001	25600000	710	37	29	2
Chr2	25600001	25700000	712	50	43	0
Chr2	25700001	25800000	623	36	27	0
Chr2	25800001	25900000	834	53	44	2
Chr2	25900001	26000000	573	20	17	11
Chr2	26000001	26100000	1387	41	38	24
Chr2	26100001	26200000	1214	24	26	7
Chr2	26200001	26300000	854	39	36	13
Chr2	26300001	26400000	875	28	30	13
Chr2	26400001	26500000	700	4	25	24
Chr2	26500001	26600000	746	3	29	27
Chr2	26600001	26700000	814	7	34	32
Chr2	26700001	26800000	692	1	18	16
Chr2	26800001	26900000	517	4	15	13
Chr2	26900001	27000000	670	29	24	15
Chr2	27000001	27100000	686	37	35	11
Chr2	27100001	27200000	1174	49	44	20
Chr2	27200001	27300000	658	37	27	18
Chr2	27300001	27400000	766	47	42	17
Chr2	27400001	27500000	925	41	40	14
Chr2	27500001	27600000	771	41	42	4
Chr2	27600001	27700000	961	52	48	1
Chr2	27700001	27800000	704	23	21	1
Chr2	27800001	27900000	752	29	28	1
Chr2	27900001	28000000	742	40	33	0
Chr2	28000001	28100000	517	17	17	0
Chr2	28100001	28200000	713	40	40	0
Chr2	28200001	28300000	678	36	29	1
Chr2	28300001	28400000	729	51	43	1

					InDel	
			All	Bengal/Nona Bokra	PSSR/Bengal	PSSR/Nona Bokra
Chr2	28400001	28500000	726	22	19	2
Chr2	28500001	28600000	671	31	34	3
Chr2	28600001	28700000	658	38	33	13
Chr2	28700001	28800000	600	11	21	6
Chr2	28800001	28900000	901	1	28	22
Chr2	28900001	29000000	703	1	24	18
Chr2	29000001	29100000	492	1	13	14
Chr2	29100001	29200000	664	9	19	11
Chr2	29200001	29300000	682	26	18	27
Chr2	29300001	29400000	764	39	37	18
Chr2	29400001	29500000	950	29	21	12
Chr2	29500001	29600000	718	21	18	26
Chr2	29600001	29700000	51	2	3	2
Chr2	29700001	29800000	688	24	25	23
Chr2	29800001	29900000	805	41	26	24
Chr2	29900001	30000000	752	28	20	11
Chr2	30000001	30100000	726	35	28	19
Chr2	30100001	30200000	617	23	19	11
Chr2	30200001	30300000	658	20	15	11
Chr2	30300001	30400000	726	30	27	18
Chr2	30400001	30500000	740	19	27	18
Chr2	30500001	30600000	564	3	11	10
Chr2	30600001	30700000	810	13	22	17
Chr2	30700001	30800000	680	26	28	13
Chr2	30800001	30900000	662	24	17	7
Chr2	30900001	31000000	820	27	23	17
Chr2	31000001	31100000	701	29	29	20
Chr2	31100001	31200000	753	32	35	16
Chr2	31200001	31300000	893	39	29	25
Chr2	31300001	31400000	657	26	22	15
Chr2	31400001	31500000	827	30	30	14
Chr2	31500001	31600000	635	29	23	9
Chr2	31600001	31700000	607	27	28	2
Chr2	31700001	31800000	568	29	22	0
Chr2	31800001	31900000	511	21	20	0
Chr2	31900001	32000000	732	39	28	1
Chr2	32000001	32100000	879	42	42	2
Chr2	32100001	32200000	629	34	27	0
Chr2	32200001	32300000	555	25	26	1
Chr2	32300001	32400000	622	32	26	5
Chr2	32400001	32500000	1084	40	35	3
Chr2	32500001	32600000	629	27	29	1
Chr2	32600001	32700000	688	38	29	3
Chr2	32700001	32800000	466	21	17	1
Chr2	32800001	32900000	878	51	44	0

					InDel	
			All	Bengal/Nona Bokra	PSSR/Bengal	PSSR/Nona Bokra
Chr2	32900001	33000000	616	29	26	0
Chr2	33000001	33100000	671	38	34	5
Chr2	33100001	33200000	814	47	34	11
Chr2	33200001	33300000	883	21	20	13
Chr2	33300001	33400000	597	32	24	17
Chr2	33400001	33500000	862	42	40	10
Chr2	33500001	33600000	806	33	28	10
Chr2	33600001	33700000	957	45	44	24
Chr2	33700001	33800000	487	27	25	0
Chr2	33800001	33900000	736	50	36	12
Chr2	33900001	34000000	696	27	22	15
Chr2	34000001	34100000	675	36	17	40
Chr2	34100001	34200000	216	8	8	8
Chr2	34200001	34300000	1001	45	38	30
Chr2	34300001	34400000	571	27	19	11
Chr2	34400001	34500000	1047	50	39	15
Chr2	34500001	34600000	792	33	23	2
Chr2	34600001	34700000	742	35	34	8
Chr2	34700001	34800000	657	2	1	1
Chr2	34800001	34900000	860	13	4	11
Chr2	34900001	35000000	507	17	0	17
Chr2	35000001	35100000	702	22	0	15
Chr2	35100001	35200000	968	39	8	31
Chr2	35200001	35300000	639	10	6	5
Chr2	35300001	35400000	715	39	34	3
Chr2	35400001	35500000	458	24	19	1
Chr2	35500001	35600000	541	28	19	2
Chr2	35600001	35700000	462	34	21	4
Chr2	35700001	35800000	561	29	20	15
Chr2	35800001	35900000	887	40	34	14
Chr2	35900001	36000000	266	10	7	3
Chr3	Chr3					
Chr3	1	100000	46	1	1	0
Chr3	100001	200000	104	7	3	5
Chr3	200001	300000	683	32	27	3
Chr3	300001	400000	488	25	23	0
Chr3	400001	500000	535	38	31	2
Chr3	500001	600000	698	45	38	0
Chr3	600001	700000	589	35	26	1
Chr3	700001	800000	587	40	38	1
Chr3	800001	900000	935	34	30	0
Chr3	900001	1000000	731	33	32	1
Chr3	1000001	1100000	737	24	21	1
Chr3	1100001	1200000	745	43	38	2
Chr3	1200001	1300000	910	44	36	2

					InDel	
			All	Bengal/Nona Bokra	PSSR/Bengal	PSSR/Nona Bokra
Chr3	1300001	1400000	669	29	27	0
Chr3	1400001	1500000	1065	25	21	0
Chr3	1500001	1600000	792	36	30	1
Chr3	1600001	1700000	366	14	16	1
Chr3	1700001	1800000	691	44	40	6
Chr3	1800001	1900000	555	39	36	1
Chr3	1900001	2000000	641	39	32	2
Chr3	2000001	2100000	680	41	38	1
Chr3	2100001	2200000	501	29	1	28
Chr3	2200001	2300000	752	19	13	15
Chr3	2300001	2400000	518	28	24	0
Chr3	2400001	2500000	486	22	18	1
Chr3	2500001	2600000	478	24	18	0
Chr3	2600001	2700000	656	31	30	0
Chr3	2700001	2800000	858	37	49	6
Chr3	2800001	2900000	503	29	26	0
Chr3	2900001	3000000	611	32	29	3
Chr3	3000001	3100000	605	34	28	2
Chr3	3100001	3200000	565	36	34	0
Chr3	3200001	3300000	574	38	30	5
Chr3	3300001	3400000	862	47	48	1
Chr3	3400001	3500000	642	29	25	0
Chr3	3500001	3600000	492	27	27	0
Chr3	3600001	3700000	557	34	33	1
Chr3	3700001	3800000	549	30	32	3
Chr3	3800001	3900000	934	49	43	1
Chr3	3900001	4000000	676	13	15	0
Chr3	4000001	4100000	796	34	30	3
Chr3	4100001	4200000	511	18	17	1
Chr3	4200001	4300000	568	25	22	0
Chr3	4300001	4400000	912	35	45	2
Chr3	4400001	4500000	809	33	28	1
Chr3	4500001	4600000	702	38	33	0
Chr3	4600001	4700000	1156	54	42	22
Chr3	4700001	4800000	818	28	18	17
Chr3	4800001	4900000	879	32	32	11
Chr3	4900001	5000000	755	56	42	0
Chr3	5000001	5100000	848	40	46	1
Chr3	5100001	5200000	1039	58	53	0
Chr3	5200001	5300000	787	41	34	0
Chr3	5300001	5400000	581	28	23	1
Chr3	5400001	5500000	722	56	54	0
Chr3	5500001	5600000	662	39	39	0
Chr3	5600001	5700000	666	29	21	1
Chr3	5700001	5800000	729	35	29	2

					InDel	
			All	Bengal/Nona Bokra	PSSR/Bengal	PSSR/Nona Bokra
Chr3	5800001	5900000	658	44	41	0
Chr3	5900001	6000000	417	25	26	1
Chr3	6000001	6100000	574	34	27	11
Chr3	6100001	6200000	500	22	21	1
Chr3	6200001	6300000	512	30	32	0
Chr3	6300001	6400000	652	30	32	1
Chr3	6400001	6500000	605	29	25	0
Chr3	6500001	6600000	549	30	25	0
Chr3	6600001	6700000	528	28	23	1
Chr3	6700001	6800000	701	50	41	10
Chr3	6800001	6900000	733	40	11	30
Chr3	6900001	7000000	913	44	36	18
Chr3	7000001	7100000	873	37	31	21
Chr3	7100001	7200000	624	32	31	13
Chr3	7200001	7300000	791	35	27	19
Chr3	7300001	7400000	688	25	28	11
Chr3	7400001	7500000	547	28	20	4
Chr3	7500001	7600000	621	27	22	10
Chr3	7600001	7700000	787	38	24	13
Chr3	7700001	7800000	834	32	23	17
Chr3	7800001	7900000	863	21	21	5
Chr3	7900001	8000000	821	46	31	13
Chr3	8000001	8100000	555	29	7	23
Chr3	8100001	8200000	796	31	28	11
Chr3	8200001	8300000	857	27	28	11
Chr3	8300001	8400000	934	28	32	9
Chr3	8400001	8500000	727	33	27	6
Chr3	8500001	8600000	707	33	31	6
Chr3	8600001	8700000	713	44	32	12
Chr3	8700001	8800000	650	24	21	19
Chr3	8800001	8900000	588	33	20	11
Chr3	8900001	9000000	827	35	31	11
Chr3	9000001	9100000	549	20	15	9
Chr3	9100001	9200000	806	36	27	17
Chr3	9200001	9300000	799	31	33	16
Chr3	9300001	9400000	813	35	28	12
Chr3	9400001	9500000	999	39	37	19
Chr3	9500001	9600000	790	40	27	22
Chr3	9600001	9700000	837	24	20	12
Chr3	9700001	9800000	646	26	21	16
Chr3	9800001	9900000	912	34	33	12
Chr3	9900001	10000000	892	42	30	18
Chr3	10000001	10100000	851	41	29	16
Chr3	10100001	10200000	876	39	35	12
Chr3	10200001	10300000	850	39	28	21

					InDel	
			All	Bengal/Nona Bokra	PSSR/Bengal	PSSR/Nona Bokra
Chr3	10300001	10400000	858	22	5	21
Chr3	10400001	10500000	1020	28	16	17
Chr3	10500001	10600000	1061	28	25	20
Chr3	10600001	10700000	944	25	18	15
Chr3	10700001	10800000	1052	22	21	10
Chr3	10800001	10900000	903	18	15	16
Chr3	10900001	11000000	749	21	11	23
Chr3	11000001	11100000	970	21	1	18
Chr3	11100001	11200000	837	25	2	24
Chr3	11200001	11300000	966	27	3	25
Chr3	11300001	11400000	1085	35	1	29
Chr3	11400001	11500000	1183	24	1	24
Chr3	11500001	11600000	988	21	1	14
Chr3	11600001	11700000	734	22	0	22
Chr3	11700001	11800000	847	21	8	13
Chr3	11800001	11900000	818	31	23	7
Chr3	11900001	12000000	895	29	24	20
Chr3	12000001	12100000	769	7	24	26
Chr3	12100001	12200000	709	4	22	22
Chr3	12200001	12300000	595	8	17	16
Chr3	12300001	12400000	722	24	22	11
Chr3	12400001	12500000	668	37	21	17
Chr3	12500001	12600000	837	37	30	20
Chr3	12600001	12700000	911	49	39	21
Chr3	12700001	12800000	766	35	20	25
Chr3	12800001	12900000	507	46	0	46
Chr3	12900001	13000000	718	44	1	46
Chr3	13000001	13100000	201	16	1	14
Chr3	13100001	13200000	331	20	19	0
Chr3	13200001	13300000	710	34	30	0
Chr3	13300001	13400000	232	9	7	1
Chr3	13400001	13500000	261	0	0	0
Chr3	13500001	13600000	110	2	1	0
Chr3	13600001	13700000	385	29	24	0
Chr3	13700001	13800000	611	21	18	1
Chr3	13800001	13900000	606	29	26	0
Chr3	13900001	14000000	767	34	32	0
Chr3	14000001	14100000	923	34	31	0
Chr3	14100001	14200000	568	41	21	0
Chr3	14200001	14300000	1228	83	48	10
Chr3	14300001	14400000	707	40	40	3
Chr3	14400001	14500000	1034	71	45	1
Chr3	14500001	14600000	915	47	47	0
Chr3	14600001	14700000	885	50	43	1
Chr3	14700001	14800000	364	18	17	0

					InDel	
			All	Bengal/Nona Bokra	PSSR/Bengal	PSSR/Nona Bokra
Chr3	14800001	14900000	389	13	14	3
Chr3	14900001	15000000	832	47	30	2
Chr3	15000001	15100000	1066	39	38	2
Chr3	15100001	15200000	978	39	34	0
Chr3	15200001	15300000	673	37	31	0
Chr3	15300001	15400000	1314	78	47	4
Chr3	15400001	15500000	713	41	28	0
Chr3	15500001	15600000	698	41	34	2
Chr3	15600001	15700000	1218	82	74	0
Chr3	15700001	15800000	963	61	56	1
Chr3	15800001	15900000	1071	65	55	0
Chr3	15900001	16000000	904	41	22	26
Chr3	16000001	16100000	845	29	21	20
Chr3	16100001	16200000	1160	36	32	24
Chr3	16200001	16300000	1022	38	33	18
Chr3	16300001	16400000	982	34	28	1
Chr3	16400001	16500000	1156	26	22	12
Chr3	16500001	16600000	1275	27	30	3
Chr3	16600001	16700000	1066	32	24	21
Chr3	16700001	16800000	1439	42	35	25
Chr3	16800001	16900000	1245	36	30	20
Chr3	16900001	17000000	1053	52	43	14
Chr3	17000001	17100000	1020	44	42	15
Chr3	17100001	17200000	848	29	19	10
Chr3	17200001	17300000	879	59	47	2
Chr3	17300001	17400000	1005	35	31	1
Chr3	17400001	17500000	753	44	40	0
Chr3	17500001	17600000	933	40	32	0
Chr3	17600001	17700000	883	44	32	2
Chr3	17700001	17800000	691	31	31	2
Chr3	17800001	17900000	854	42	36	0
Chr3	17900001	18000000	1040	39	37	2
Chr3	18000001	18100000	951	26	23	3
Chr3	18100001	18200000	864	30	30	0
Chr3	18200001	18300000	884	11	12	1
Chr3	18300001	18400000	241	10	7	0
Chr3	18400001	18500000	483	18	9	0
Chr3	18500001	18600000	758	27	26	1
Chr3	18600001	18700000	669	23	17	0
Chr3	18700001	18800000	936	12	12	0
Chr3	18800001	18900000	684	12	10	0
Chr3	18900001	19000000	640	12	6	1
Chr3	19000001	19100000	1026	14	13	0
Chr3	19100001	19200000	871	28	26	0
Chr3	19200001	19300000	878	14	21	0

					InDel	
			All	Bengal/Nona Bokra	PSSR/Bengal	PSSR/Nona Bokra
Chr3	19300001	19400000	613	3	3	0
Chr3	19400001	19500000	853	11	14	1
Chr3	19500001	19600000	550	20	17	0
Chr3	19600001	19700000	807	13	12	0
Chr3	19700001	19800000	856	12	13	0
Chr3	19800001	19900000	896	18	10	0
Chr3	19900001	20000000	932	29	27	0
Chr3	20000001	20100000	806	17	24	1
Chr3	20100001	20200000	900	15	13	1
Chr3	20200001	20300000	1042	29	27	0
Chr3	20300001	20400000	876	24	19	0
Chr3	20400001	20500000	895	41	34	1
Chr3	20500001	20600000	820	30	27	0
Chr3	20600001	20700000	666	22	20	0
Chr3	20700001	20800000	858	42	31	1
Chr3	20800001	20900000	820	27	19	1
Chr3	20900001	21000000	865	39	39	0
Chr3	21000001	21100000	1013	40	31	20
Chr3	21100001	21200000	1061	43	38	8
Chr3	21200001	21300000	992	52	41	0
Chr3	21300001	21400000	507	20	19	10
Chr3	21400001	21500000	886	21	19	8
Chr3	21500001	21600000	785	20	18	9
Chr3	21600001	21700000	691	34	32	12
Chr3	21700001	21800000	698	23	14	9
Chr3	21800001	21900000	282	2	4	2
Chr3	21900001	22000000	974	20	20	8
Chr3	22000001	22100000	1157	32	29	11
Chr3	22100001	22200000	1047	29	20	13
Chr3	22200001	22300000	962	39	32	16
Chr3	22300001	22400000	1159	47	37	16
Chr3	22400001	22500000	949	30	27	0
Chr3	22500001	22600000	915	41	36	0
Chr3	22600001	22700000	858	49	45	0
Chr3	22700001	22800000	773	53	35	1
Chr3	22800001	22900000	1188	74	57	3
Chr3	22900001	23000000	829	28	27	2
Chr3	23000001	23100000	1088	48	40	11
Chr3	23100001	23200000	914	17	18	12
Chr3	23200001	23300000	1087	3	17	3
Chr3	23300001	23400000	679	37	40	3
Chr3	23400001	23500000	836	52	45	1
Chr3	23500001	23600000	914	33	23	12
Chr3	23600001	23700000	903	33	40	1
Chr3	23700001	23800000	919	32	25	11

					InDel	
			All	Bengal/Nona Bokra	PSSR/Bengal	PSSR/Nona Bokra
Chr3	23800001	23900000	866	43	41	6
Chr3	23900001	24000000	910	44	38	0
Chr3	24000001	24100000	553	27	22	0
Chr3	24100001	24200000	372	11	13	0
Chr3	24200001	24300000	982	53	50	0
Chr3	24300001	24400000	1074	58	43	2
Chr3	24400001	24500000	932	39	32	13
Chr3	24500001	24600000	952	43	37	21
Chr3	24600001	24700000	1084	48	36	21
Chr3	24700001	24800000	955	37	24	18
Chr3	24800001	24900000	1078	37	31	25
Chr3	24900001	25000000	970	30	27	30
Chr3	25000001	25100000	794	42	29	26
Chr3	25100001	25200000	449	29	22	2
Chr3	25200001	25300000	203	20	1	18
Chr3	25300001	25400000	543	36	2	37
Chr3	25400001	25500000	637	27	0	27
Chr3	25500001	25600000	136	15	1	14
Chr3	25600001	25700000	44	1	0	1
Chr3	25700001	25800000	69	1	1	0
Chr3	25800001	25900000	38	1	0	0
Chr3	25900001	26000000	128	5	5	1
Chr3	26000001	26100000	38	2	1	1
Chr3	26100001	26200000	60	2	0	1
Chr3	26200001	26300000	57	4	4	0
Chr3	26300001	26400000	34	1	1	0
Chr3	26400001	26500000	214	5	1	4
Chr3	26500001	26600000	694	27	19	9
Chr3	26600001	26700000	1224	77	54	1
Chr3	26700001	26800000	1305	45	50	36
Chr3	26800001	26900000	1030	49	42	10
Chr3	26900001	27000000	618	34	32	0
Chr3	27000001	27100000	1041	64	38	6
Chr3	27100001	27200000	837	15	36	19
Chr3	27200001	27300000	621	17	19	11
Chr3	27300001	27400000	662	34	34	1
Chr3	27400001	27500000	698	49	42	2
Chr3	27500001	27600000	166	2	6	7
Chr3	27600001	27700000	429	4	12	8
Chr3	27700001	27800000	939	39	29	6
Chr3	27800001	27900000	828	34	25	21
Chr3	27900001	28000000	959	23	21	20
Chr3	28000001	28100000	892	35	22	17
Chr3	28100001	28200000	756	42	41	12
Chr3	28200001	28300000	585	33	32	0

					InDel	
			All	Bengal/Nona Bokra	PSSR/Bengal	PSSR/Nona Bokra
Chr3	28300001	28400000	853	54	51	1
Chr3	28400001	28500000	692	43	43	1
Chr3	28500001	28600000	118	5	2	3
Chr3	28600001	28700000	23	2	1	1
Chr3	28700001	28800000	217	0	5	4
Chr3	28800001	28900000	544	1	15	13
Chr3	28900001	29000000	196	2	7	5
Chr3	29000001	29100000	646	47	3	47
Chr3	29100001	29200000	459	17	3	14
Chr3	29200001	29300000	691	37	37	0
Chr3	29300001	29400000	476	2	20	14
Chr3	29400001	29500000	719	7	32	29
Chr3	29500001	29600000	811	31	21	15
Chr3	29600001	29700000	992	46	49	24
Chr3	29700001	29800000	782	25	15	18
Chr3	29800001	29900000	868	43	35	17
Chr3	29900001	30000000	593	20	18	13
Chr3	30000001	30100000	818	48	39	22
Chr3	30100001	30200000	760	29	26	9
Chr3	30200001	30300000	543	19	19	8
Chr3	30300001	30400000	1004	41	30	18
Chr3	30400001	30500000	931	27	25	10
Chr3	30500001	30600000	390	26	22	7
Chr3	30600001	30700000	90	10	5	0
Chr3	30700001	30800000	525	30	25	0
Chr3	30800001	30900000	593	32	31	0
Chr3	30900001	31000000	652	27	20	0
Chr3	31000001	31100000	656	45	41	0
Chr3	31100001	31200000	518	32	21	0
Chr3	31200001	31300000	736	41	42	0
Chr3	31300001	31400000	744	56	47	0
Chr3	31400001	31500000	750	53	42	0
Chr3	31500001	31600000	747	43	40	0
Chr3	31600001	31700000	626	43	41	0
Chr3	31700001	31800000	649	39	29	0
Chr3	31800001	31900000	611	31	27	1
Chr3	31900001	32000000	956	60	54	0
Chr3	32000001	32100000	699	39	37	0
Chr3	32100001	32200000	683	35	36	7
Chr3	32200001	32300000	899	41	32	19
Chr3	32300001	32400000	393	3	11	5
Chr3	32400001	32500000	731	21	30	12
Chr3	32500001	32600000	678	29	28	14
Chr3	32600001	32700000	771	56	36	31
Chr3	32700001	32800000	535	35	25	1

					InDel	
			All	Bengal/Nona Bokra	PSSR/Bengal	PSSR/Nona Bokra
Chr3	32800001	32900000	600	42	38	0
Chr3	32900001	33000000	761	39	34	0
Chr3	33000001	33100000	632	32	28	0
Chr3	33100001	33200000	821	49	43	1
Chr3	33200001	33300000	1015	46	37	0
Chr3	33300001	33400000	643	38	36	0
Chr3	33400001	33500000	562	29	22	2
Chr3	33500001	33600000	96	7	6	0
Chr3	33600001	33700000	11	3	1	0
Chr3	33700001	33800000	20	3	2	0
Chr3	33800001	33900000	422	23	23	0
Chr3	33900001	34000000	609	36	35	0
Chr3	34000001	34100000	737	39	36	0
Chr3	34100001	34200000	546	21	20	4
Chr3	34200001	34300000	835	53	38	16
Chr3	34300001	34400000	985	40	29	21
Chr3	34400001	34500000	813	34	29	18
Chr3	34500001	34600000	688	2	21	19
Chr3	34600001	34700000	885	45	35	18
Chr3	34700001	34800000	723	49	24	23
Chr3	34800001	34900000	627	30	2	30
Chr3	34900001	35000000	244	21	4	18
Chr3	35000001	35100000	22	0	0	0
Chr3	35100001	35200000	20	1	2	0
Chr3	35200001	35300000	841	84	4	83
Chr3	35300001	35400000	1097	65	50	5
Chr3	35400001	35500000	920	55	38	5
Chr3	35500001	35600000	701	22	23	25
Chr3	35600001	35700000	572	30	15	28
Chr3	35700001	35800000	469	23	8	12
Chr3	35800001	35900000	426	23	17	11
Chr3	35900001	36000000	414	29	24	1
Chr3	36000001	36100000	204	11	11	0
Chr3	36100001	36200000	311	17	6	12
Chr3	36200001	36300000	447	35	0	35
Chr3	36300001	36400000	432	22	1	23
Chr3	36400001	36500000	50	1	1	2
Chr4	Chr4					
Chr4	1	100000	994	9	4	7
Chr4	100001	200000	1621	39	12	33
Chr4	200001	300000	1461	28	20	20
Chr4	300001	400000	1211	45	30	40
Chr4	400001	500000	1309	42	41	25
Chr4	500001	600000	870	32	19	21
Chr4	600001	700000	1877	33	17	21

					InDel	
			All	Bengal/Nona Bokra	PSSR/Bengal	PSSR/Nona Bokra
Chr4	700001	800000	865	12	11	8
Chr4	800001	900000	557	12	10	9
Chr4	900001	1000000	1543	42	13	31
Chr4	1000001	1100000	1052	26	19	27
Chr4	1100001	1200000	610	19	13	12
Chr4	1200001	1300000	1153	30	31	0
Chr4	1300001	1400000	698	6	4	0
Chr4	1400001	1500000	450	0	0	0
Chr4	1500001	1600000	283	0	1	0
Chr4	1600001	1700000	618	6	8	1
Chr4	1700001	1800000	1642	49	35	0
Chr4	1800001	1900000	987	36	36	0
Chr4	1900001	2000000	700	16	14	1
Chr4	2000001	2100000	875	16	12	7
Chr4	2100001	2200000	214	1	0	0
Chr4	2200001	2300000	278	0	1	1
Chr4	2300001	2400000	540	0	0	0
Chr4	2400001	2500000	1403	34	27	26
Chr4	2500001	2600000	868	16	17	5
Chr4	2600001	2700000	535	0	0	3
Chr4	2700001	2800000	725	1	5	4
Chr4	2800001	2900000	1151	18	16	17
Chr4	2900001	3000000	918	7	4	7
Chr4	3000001	3100000	169	0	0	0
Chr4	3100001	3200000	321	0	2	0
Chr4	3200001	3300000	451	1	1	0
Chr4	3300001	3400000	714	7	2	4
Chr4	3400001	3500000	858	8	1	4
Chr4	3500001	3600000	914	11	6	10
Chr4	3600001	3700000	1206	12	12	7
Chr4	3700001	3800000	944	7	5	4
Chr4	3800001	3900000	815	7	11	8
Chr4	3900001	4000000	679	7	5	6
Chr4	4000001	4100000	1048	15	8	13
Chr4	4100001	4200000	1226	12	8	9
Chr4	4200001	4300000	1148	8	11	11
Chr4	4300001	4400000	965	0	30	28
Chr4	4400001	4500000	1190	1	35	30
Chr4	4500001	4600000	916	8	19	17
Chr4	4600001	4700000	1087	14	11	12
Chr4	4700001	4800000	882	4	25	19
Chr4	4800001	4900000	427	15	3	1
Chr4	4900001	5000000	657	16	10	9
Chr4	5000001	5100000	1145	20	23	22
Chr4	5100001	5200000	1679	47	39	22

					InDel	
			All	Bengal/Nona Bokra	PSSR/Bengal	PSSR/Nona Bokra
Chr4	5200001	5300000	1182	38	32	3
Chr4	5300001	5400000	1155	47	26	2
Chr4	5400001	5500000	756	42	23	1
Chr4	5500001	5600000	937	34	35	0
Chr4	5600001	5700000	550	33	27	0
Chr4	5700001	5800000	608	15	15	5
Chr4	5800001	5900000	1220	13	28	18
Chr4	5900001	6000000	1089	37	22	17
Chr4	6000001	6100000	1157	26	19	9
Chr4	6100001	6200000	1092	11	6	9
Chr4	6200001	6300000	1101	32	30	21
Chr4	6300001	6400000	1091	19	18	14
Chr4	6400001	6500000	1390	24	25	11
Chr4	6500001	6600000	1168	20	25	16
Chr4	6600001	6700000	504	19	0	0
Chr4	6700001	6800000	347	6	0	1
Chr4	6800001	6900000	309	4	0	0
Chr4	6900001	7000000	1737	29	40	27
Chr4	7000001	7100000	570	5	4	11
Chr4	7100001	7200000	858	15	11	14
Chr4	7200001	7300000	820	13	20	9
Chr4	7300001	7400000	902	24	23	16
Chr4	7400001	7500000	489	8	13	10
Chr4	7500001	7600000	784	8	14	8
Chr4	7600001	7700000	299	0	0	0
Chr4	7700001	7800000	282	0	1	0
Chr4	7800001	7900000	580	5	5	4
Chr4	7900001	8000000	482	7	6	2
Chr4	8000001	8100000	1060	2	5	2
Chr4	8100001	8200000	945	2	2	2
Chr4	8200001	8300000	1471	30	31	5
Chr4	8300001	8400000	943	14	12	2
Chr4	8400001	8500000	661	8	8	5
Chr4	8500001	8600000	1154	26	25	2
Chr4	8600001	8700000	915	13	14	2
Chr4	8700001	8800000	1036	12	18	1
Chr4	8800001	8900000	1259	17	16	1
Chr4	8900001	9000000	741	9	7	3
Chr4	9000001	9100000	1011	11	15	1
Chr4	9100001	9200000	28	0	0	0
Chr4	9200001	9300000	928	14	19	2
Chr4	9300001	9400000	1077	21	22	6
Chr4	9400001	9500000	984	6	10	1
Chr4	9500001	9600000	461	0	0	0
Chr4	9600001	9700000	1301	23	28	3

					InDel	
			All	Bengal/Nona Bokra	PSSR/Bengal	PSSR/Nona Bokra
Chr4	9700001	9800000	580	7	9	1
Chr4	9800001	9900000	668	4	5	1
Chr4	9900001	10000000	1066	27	22	6
Chr4	10000001	10100000	1338	19	18	3
Chr4	10100001	10200000	382	1	1	0
Chr4	10200001	10300000	614	2	0	0
Chr4	10300001	10400000	1295	6	8	1
Chr4	10400001	10500000	689	9	13	1
Chr4	10500001	10600000	886	3	1	0
Chr4	10600001	10700000	1467	23	20	5
Chr4	10700001	10800000	1641	21	23	4
Chr4	10800001	10900000	1313	14	15	0
Chr4	10900001	11000000	808	14	17	5
Chr4	11000001	11100000	1108	16	15	8
Chr4	11100001	11200000	934	9	11	4
Chr4	11200001	11300000	1126	27	23	5
Chr4	11300001	11400000	677	19	14	4
Chr4	11400001	11500000	1000	27	26	15
Chr4	11500001	11600000	1181	34	22	8
Chr4	11600001	11700000	952	22	25	2
Chr4	11700001	11800000	858	19	18	6
Chr4	11800001	11900000	1488	28	21	23
Chr4	11900001	12000000	550	14	12	11
Chr4	12000001	12100000	1376	35	34	20
Chr4	12100001	12200000	1165	20	17	1
Chr4	12200001	12300000	777	25	27	0
Chr4	12300001	12400000	878	32	29	1
Chr4	12400001	12500000	590	35	20	0
Chr4	12500001	12600000	835	42	27	5
Chr4	12600001	12700000	1593	78	47	12
Chr4	12700001	12800000	508	9	6	5
Chr4	12800001	12900000	140	1	1	0
Chr4	12900001	13000000	1444	27	37	23
Chr4	13000001	13100000	618	8	7	5
Chr4	13100001	13200000	1082	24	23	18
Chr4	13200001	13300000	1041	26	24	13
Chr4	13300001	13400000	1403	14	18	11
Chr4	13400001	13500000	824	27	36	1
Chr4	13500001	13600000	760	32	26	0
Chr4	13600001	13700000	849	33	30	1
Chr4	13700001	13800000	598	15	13	0
Chr4	13800001	13900000	738	34	33	0
Chr4	13900001	14000000	615	18	12	1
Chr4	14000001	14100000	595	14	20	0
Chr4	14100001	14200000	717	16	15	0

					InDel	
			All	Bengal/Nona Bokra	PSSR/Bengal	PSSR/Nona Bokra
Chr4	14200001	14300000	478	4	3	0
Chr4	14300001	14400000	324	2	3	0
Chr4	14400001	14500000	143	1	2	1
Chr4	14500001	14600000	545	6	10	4
Chr4	14600001	14700000	533	12	3	15
Chr4	14700001	14800000	483	20	1	21
Chr4	14800001	14900000	349	15	1	15
Chr4	14900001	15000000	113	0	0	0
Chr4	15000001	15100000	272	6	0	5
Chr4	15100001	15200000	191	6	0	7
Chr4	15200001	15300000	204	6	1	6
Chr4	15300001	15400000	356	15	1	15
Chr4	15400001	15500000	164	0	2	0
Chr4	15500001	15600000	29	1	1	1
Chr4	15600001	15700000	2	0	0	0
Chr4	15700001	15800000	4	0	0	0
Chr4	15800001	15900000	344	17	0	17
Chr4	15900001	16000000	1433	48	2	47
Chr4	16000001	16100000	461	20	2	18
Chr4	16100001	16200000	1344	34	20	33
Chr4	16200001	16300000	1725	69	74	7
Chr4	16300001	16400000	927	24	25	0
Chr4	16400001	16500000	587	23	21	0
Chr4	16500001	16600000	859	48	47	2
Chr4	16600001	16700000	1223	63	41	0
Chr4	16700001	16800000	193	14	9	0
Chr4	16800001	16900000	46	0	1	0
Chr4	16900001	17000000	46	1	0	1
Chr4	17000001	17100000	35	2	2	0
Chr4	17100001	17200000	52	2	2	0
Chr4	17200001	17300000	50	3	3	1
Chr4	17300001	17400000	66	0	0	0
Chr4	17400001	17500000	397	21	0	21
Chr4	17500001	17600000	316	12	2	12
Chr4	17600001	17700000	906	21	0	21
Chr4	17700001	17800000	1255	33	29	31
Chr4	17800001	17900000	1077	20	25	17
Chr4	17900001	18000000	1123	36	34	32
Chr4	18000001	18100000	1474	23	17	18
Chr4	18100001	18200000	1330	31	26	18
Chr4	18200001	18300000	1903	43	41	9
Chr4	18300001	18400000	1008	18	8	4
Chr4	18400001	18500000	1345	54	41	12
Chr4	18500001	18600000	1689	88	53	6
Chr4	18600001	18700000	1544	52	53	41

					InDel	
			All	Bengal/Nona Bokra	PSSR/Bengal	PSSR/Nona Bokra
Chr4	18700001	18800000	784	24	18	18
Chr4	18800001	18900000	939	42	7	23
Chr4	18900001	19000000	864	56	63	13
Chr4	19000001	19100000	698	0	24	18
Chr4	19100001	19200000	514	24	29	6
Chr4	19200001	19300000	1004	51	54	0
Chr4	19300001	19400000	797	45	35	1
Chr4	19400001	19500000	854	65	59	2
Chr4	19500001	19600000	1081	73	72	0
Chr4	19600001	19700000	884	48	47	1
Chr4	19700001	19800000	651	25	20	3
Chr4	19800001	19900000	857	50	43	4
Chr4	19900001	20000000	640	33	28	1
Chr4	20000001	20100000	777	28	32	11
Chr4	20100001	20200000	818	48	38	5
Chr4	20200001	20300000	894	34	22	8
Chr4	20300001	20400000	802	28	28	6
Chr4	20400001	20500000	1116	50	33	33
Chr4	20500001	20600000	1029	43	38	20
Chr4	20600001	20700000	1312	60	46	18
Chr4	20700001	20800000	1341	41	42	28
Chr4	20800001	20900000	1210	49	22	28
Chr4	20900001	21000000	839	34	22	8
Chr4	21000001	21100000	1105	63	13	52
Chr4	21100001	21200000	314	24	1	24
Chr4	21200001	21300000	812	31	22	25
Chr4	21300001	21400000	583	28	10	14
Chr4	21400001	21500000	742	37	22	23
Chr4	21500001	21600000	779	14	20	21
Chr4	21600001	21700000	631	2	25	20
Chr4	21700001	21800000	624	16	12	17
Chr4	21800001	21900000	881	36	27	25
Chr4	21900001	22000000	636	0	14	14
Chr4	22000001	22100000	803	29	31	15
Chr4	22100001	22200000	541	20	4	17
Chr4	22200001	22300000	359	22	1	24
Chr4	22300001	22400000	36	3	2	1
Chr4	22400001	22500000	329	18	20	2
Chr4	22500001	22600000	310	17	20	0
Chr4	22600001	22700000	261	9	10	0
Chr4	22700001	22800000	58	1	1	0
Chr4	22800001	22900000	125	9	8	3
Chr4	22900001	23000000	34	2	1	0
Chr4	23000001	23100000	24	0	0	0
Chr4	23100001	23200000	29	2	1	1

					InDel	
			All	Bengal/Nona Bokra	PSSR/Bengal	PSSR/Nona Bokra
Chr4	23200001	23300000	25	1	1	0
Chr4	23300001	23400000	117	6	3	1
Chr4	23400001	23500000	755	49	39	0
Chr4	23500001	23600000	794	34	34	0
Chr4	23600001	23700000	628	14	12	8
Chr4	23700001	23800000	883	33	24	22
Chr4	23800001	23900000	985	51	33	17
Chr4	23900001	24000000	802	39	31	6
Chr4	24000001	24100000	775	33	28	15
Chr4	24100001	24200000	834	34	35	11
Chr4	24200001	24300000	742	38	36	0
Chr4	24300001	24400000	478	12	15	5
Chr4	24400001	24500000	641	29	28	5
Chr4	24500001	24600000	649	22	12	14
Chr4	24600001	24700000	805	27	32	9
Chr4	24700001	24800000	821	24	20	7
Chr4	24800001	24900000	660	36	24	19
Chr4	24900001	25000000	1254	73	39	27
Chr4	25000001	25100000	755	18	22	12
Chr4	25100001	25200000	764	36	35	21
Chr4	25200001	25300000	766	28	18	13
Chr4	25300001	25400000	459	25	8	25
Chr4	25400001	25500000	515	40	3	38
Chr4	25500001	25600000	555	32	3	30
Chr4	25600001	25700000	526	39	1	36
Chr4	25700001	25800000	321	16	2	17
Chr4	25800001	25900000	100	4	2	2
Chr4	25900001	26000000	30	1	1	2
Chr4	26000001	26100000	18	0	0	0
Chr4	26100001	26200000	53	0	2	0
Chr4	26200001	26300000	164	0	0	0
Chr4	26300001	26400000	143	4	3	2
Chr4	26400001	26500000	41	1	0	0
Chr4	26500001	26600000	25	1	0	0
Chr4	26600001	26700000	59	0	1	1
Chr4	26700001	26800000	31	3	1	2
Chr4	26800001	26900000	45	2	2	2
Chr4	26900001	27000000	52	2	2	0
Chr4	27000001	27100000	28	1	1	0
Chr4	27100001	27200000	527	23	19	0
Chr4	27200001	27300000	651	40	42	1
Chr4	27300001	27400000	726	44	35	0
Chr4	27400001	27500000	661	33	37	0
Chr4	27500001	27600000	688	33	29	1
Chr4	27600001	27700000	706	33	33	0

					InDel	
			All	Bengal/Nona Bokra	PSSR/Bengal	PSSR/Nona Bokra
Chr4	27700001	27800000	754	44	43	0
Chr4	27800001	27900000	801	42	38	0
Chr4	27900001	28000000	664	27	22	7
Chr4	28000001	28100000	771	37	22	31
Chr4	28100001	28200000	788	30	31	9
Chr4	28200001	28300000	766	44	30	22
Chr4	28300001	28400000	971	29	24	14
Chr4	28400001	28500000	1028	59	36	26
Chr4	28500001	28600000	643	37	24	11
Chr4	28600001	28700000	451	31	2	30
Chr4	28700001	28800000	566	39	3	38
Chr4	28800001	28900000	548	30	2	28
Chr4	28900001	29000000	438	17	3	18
Chr4	29000001	29100000	618	25	10	34
Chr4	29100001	29200000	671	27	6	28
Chr4	29200001	29300000	586	27	2	27
Chr4	29300001	29400000	981	55	15	54
Chr4	29400001	29500000	585	25	28	6
Chr4	29500001	29600000	583	34	35	0
Chr4	29600001	29700000	619	20	15	0
Chr4	29700001	29800000	670	25	22	10
Chr4	29800001	29900000	363	26	9	19
Chr4	29900001	30000000	395	24	25	15
Chr4	30000001	30100000	358	11	12	7
Chr4	30100001	30200000	760	14	23	21
Chr4	30200001	30300000	520	1	18	14
Chr4	30300001	30400000	480	1	20	22
Chr4	30400001	30500000	500	0	19	18
Chr4	30500001	30600000	371	1	16	13
Chr4	30600001	30700000	418	3	20	16
Chr4	30700001	30800000	658	15	32	16
Chr4	30800001	30900000	714	23	36	9
Chr4	30900001	31000000	727	13	20	20
Chr4	31000001	31100000	820	50	42	3
Chr4	31100001	31200000	775	35	27	7
Chr4	31200001	31300000	717	21	29	10
Chr4	31300001	31400000	1413	51	40	31
Chr4	31400001	31500000	1074	56	54	30
Chr4	31500001	31600000	654	17	19	14
Chr4	31600001	31700000	729	16	8	6
Chr4	31700001	31800000	710	24	18	17
Chr4	31800001	31900000	722	33	23	14
Chr4	31900001	32000000	546	20	20	13
Chr4	32000001	32100000	764	41	43	2
Chr4	32100001	32200000	745	23	23	0

					InDel	
			All	Bengal/Nona Bokra	PSSR/Bengal	PSSR/Nona Bokra
Chr4	32200001	32300000	657	20	18	0
Chr4	32300001	32400000	493	27	24	0
Chr4	32400001	32500000	652	27	24	0
Chr4	32500001	32600000	581	14	12	0
Chr4	32600001	32700000	545	33	32	1
Chr4	32700001	32800000	693	32	26	0
Chr4	32800001	32900000	520	24	21	0
Chr4	32900001	33000000	513	26	20	1
Chr4	33000001	33100000	561	30	21	0
Chr4	33100001	33200000	656	36	20	30
Chr4	33200001	33300000	131	9	8	3
Chr4	33300001	33400000	638	37	20	18
Chr4	33400001	33500000	666	24	15	23
Chr4	33500001	33600000	680	21	24	1
Chr4	33600001	33700000	623	1	32	31
Chr4	33700001	33800000	609	3	0	1
Chr4	33800001	33900000	603	17	0	11
Chr4	33900001	34000000	172	1	1	1
Chr4	34000001	34100000	309	1	0	0
Chr4	34100001	34200000	88	4	5	0
Chr4	34200001	34300000	336	17	16	0
Chr4	34300001	34400000	916	57	51	0
Chr4	34400001	34500000	571	30	27	0
Chr4	34500001	34600000	759	41	34	0
Chr4	34600001	34700000	525	26	27	0
Chr4	34700001	34800000	608	34	33	0
Chr4	34800001	34900000	679	41	35	0
Chr4	34900001	35000000	662	39	33	0
Chr4	35000001	35100000	770	46	36	0
Chr4	35100001	35200000	614	30	25	6
Chr4	35200001	35300000	502	27	20	3
Chr4	35300001	35400000	501	32	29	0
Chr4	35400001	35500000	583	35	34	1
Chr4	35500001	35600000	20	0	0	0
Chr5	Chr5					
Chr5	1	100000	561	25	19	5
Chr5	100001	200000	801	37	26	16
Chr5	200001	300000	436	29	4	29
Chr5	300001	400000	551	30	22	12
Chr5	400001	500000	677	28	31	13
Chr5	500001	600000	770	43	34	8
Chr5	600001	700000	662	45	19	24
Chr5	700001	800000	1163	67	35	35
Chr5	800001	900000	1158	27	31	32
Chr5	900001	1000000	881	26	22	12

					InDel	
			All	Bengal/Nona Bokra	PSSR/Bengal	PSSR/Nona Bokra
Chr5	1000001	1100000	706	24	23	6
Chr5	1100001	1200000	827	34	29	17
Chr5	1200001	1300000	780	40	28	23
Chr5	1300001	1400000	365	10	6	3
Chr5	1400001	1500000	721	39	36	17
Chr5	1500001	1600000	773	25	16	10
Chr5	1600001	1700000	783	41	25	16
Chr5	1700001	1800000	1367	75	58	28
Chr5	1800001	1900000	1432	23	26	33
Chr5	1900001	2000000	1537	78	79	17
Chr5	2000001	2100000	1201	40	40	44
Chr5	2100001	2200000	1433	58	38	68
Chr5	2200001	2300000	719	40	9	40
Chr5	2300001	2400000	1349	58	21	55
Chr5	2400001	2500000	733	32	34	1
Chr5	2500001	2600000	798	36	33	0
Chr5	2600001	2700000	793	36	35	0
Chr5	2700001	2800000	806	35	40	1
Chr5	2800001	2900000	530	35	27	1
Chr5	2900001	3000000	579	37	34	1
Chr5	3000001	3100000	804	32	37	6
Chr5	3100001	3200000	917	31	32	30
Chr5	3200001	3300000	525	32	29	0
Chr5	3300001	3400000	773	50	42	0
Chr5	3400001	3500000	271	22	12	0
Chr5	3500001	3600000	903	20	18	24
Chr5	3600001	3700000	726	31	15	28
Chr5	3700001	3800000	386	24	27	10
Chr5	3800001	3900000	341	0	12	10
Chr5	3900001	4000000	201	0	4	5
Chr5	4000001	4100000	368	1	11	9
Chr5	4100001	4200000	594	2	22	13
Chr5	4200001	4300000	521	16	11	15
Chr5	4300001	4400000	637	15	14	3
Chr5	4400001	4500000	341	14	11	0
Chr5	4500001	4600000	585	20	20	0
Chr5	4600001	4700000	897	45	40	1
Chr5	4700001	4800000	916	51	34	18
Chr5	4800001	4900000	44	4	2	1
Chr5	4900001	5000000	27	2	0	1
Chr5	5000001	5100000	31	0	2	0
Chr5	5100001	5200000	526	18	27	0
Chr5	5200001	5300000	462	21	26	0
Chr5	5300001	5400000	1129	53	63	3
Chr5	5400001	5500000	1068	34	40	3

					InDel	
			All	Bengal/Nona Bokra	PSSR/Bengal	PSSR/Nona Bokra
Chr5	5500001	5600000	1184	19	19	0
Chr5	5600001	5700000	1173	9	10	1
Chr5	5700001	5800000	911	17	22	0
Chr5	5800001	5900000	1347	36	40	6
Chr5	5900001	6000000	1024	37	31	40
Chr5	6000001	6100000	1213	64	15	25
Chr5	6100001	6200000	1160	34	25	34
Chr5	6200001	6300000	1072	53	22	37
Chr5	6300001	6400000	1476	40	27	28
Chr5	6400001	6500000	1069	2	32	26
Chr5	6500001	6600000	1088	46	30	31
Chr5	6600001	6700000	1195	40	22	37
Chr5	6700001	6800000	985	38	30	11
Chr5	6800001	6900000	1333	64	5	59
Chr5	6900001	7000000	793	38	2	37
Chr5	7000001	7100000	320	6	1	7
Chr5	7100001	7200000	426	1	0	1
Chr5	7200001	7300000	666	6	1	4
Chr5	7300001	7400000	754	6	14	12
Chr5	7400001	7500000	899	0	23	20
Chr5	7500001	7600000	662	1	20	17
Chr5	7600001	7700000	549	1	10	9
Chr5	7700001	7800000	723	2	18	15
Chr5	7800001	7900000	1008	0	26	20
Chr5	7900001	8000000	878	1	24	17
Chr5	8000001	8100000	644	1	13	13
Chr5	8100001	8200000	802	11	13	14
Chr5	8200001	8300000	933	16	18	19
Chr5	8300001	8400000	1176	16	8	19
Chr5	8400001	8500000	1142	19	11	16
Chr5	8500001	8600000	706	25	18	12
Chr5	8600001	8700000	1083	29	25	24
Chr5	8700001	8800000	1180	20	20	15
Chr5	8800001	8900000	559	13	17	13
Chr5	8900001	9000000	979	11	13	10
Chr5	9000001	9100000	809	9	10	12
Chr5	9100001	9200000	604	9	18	18
Chr5	9200001	9300000	510	3	5	7
Chr5	9300001	9400000	1116	11	12	16
Chr5	9400001	9500000	521	20	12	14
Chr5	9500001	9600000	614	11	10	9
Chr5	9600001	9700000	642	10	13	6
Chr5	9700001	9800000	949	10	10	7
Chr5	9800001	9900000	518	2	1	1
Chr5	9900001	10000000	253	1	1	0

					InDel	
			All	Bengal/Nona Bokra	PSSR/Bengal	PSSR/Nona Bokra
Chr5	10000001	10100000	251	0	0	0
Chr5	10100001	10200000	469	5	8	2
Chr5	10200001	10300000	542	6	2	4
Chr5	10300001	10400000	741	5	8	9
Chr5	10400001	10500000	808	5	4	4
Chr5	10500001	10600000	484	7	10	11
Chr5	10600001	10700000	314	2	4	3
Chr5	10700001	10800000	515	16	18	12
Chr5	10800001	10900000	640	28	18	18
Chr5	10900001	11000000	716	21	17	11
Chr5	11000001	11100000	963	25	17	19
Chr5	11100001	11200000	396	9	5	6
Chr5	11200001	11300000	407	14	9	5
Chr5	11300001	11400000	663	14	10	10
Chr5	11400001	11500000	1042	12	15	17
Chr5	11500001	11600000	153	1	2	0
Chr5	11600001	11700000	751	26	17	28
Chr5	11700001	11800000	1102	13	7	8
Chr5	11800001	11900000	477	0	2	2
Chr5	11900001	12000000	738	12	8	15
Chr5	12000001	12100000	767	7	6	6
Chr5	12100001	12200000	839	15	9	6
Chr5	12200001	12300000	1000	12	7	7
Chr5	12300001	12400000	1012	23	13	17
Chr5	12400001	12500000	460	9	4	6
Chr5	12500001	12600000	394	7	7	5
Chr5	12600001	12700000	1150	23	13	17
Chr5	12700001	12800000	709	10	5	4
Chr5	12800001	12900000	913	10	12	10
Chr5	12900001	13000000	656	12	12	10
Chr5	13000001	13100000	716	25	13	16
Chr5	13100001	13200000	408	14	10	8
Chr5	13200001	13300000	667	14	16	8
Chr5	13300001	13400000	685	22	14	10
Chr5	13400001	13500000	875	26	21	22
Chr5	13500001	13600000	854	27	22	24
Chr5	13600001	13700000	769	33	21	20
Chr5	13700001	13800000	889	34	33	31
Chr5	13800001	13900000	972	27	25	13
Chr5	13900001	14000000	804	21	23	15
Chr5	14000001	14100000	1047	12	7	5
Chr5	14100001	14200000	862	20	15	8
Chr5	14200001	14300000	896	28	21	27
Chr5	14300001	14400000	933	22	22	18
Chr5	14400001	14500000	851	31	26	4

					InDel	
			All	Bengal/Nona Bokra	PSSR/Bengal	PSSR/Nona Bokra
Chr5	14500001	14600000	791	13	14	3
Chr5	14600001	14700000	1109	40	25	24
Chr5	14700001	14800000	1043	36	31	25
Chr5	14800001	14900000	834	18	23	17
Chr5	14900001	15000000	929	23	19	10
Chr5	15000001	15100000	888	27	26	15
Chr5	15100001	15200000	786	26	17	26
Chr5	15200001	15300000	907	16	6	6
Chr5	15300001	15400000	157	0	0	0
Chr5	15400001	15500000	329	12	11	7
Chr5	15500001	15600000	1124	39	34	13
Chr5	15600001	15700000	617	29	19	8
Chr5	15700001	15800000	679	18	17	9
Chr5	15800001	15900000	646	26	15	9
Chr5	15900001	16000000	1053	30	22	16
Chr5	16000001	16100000	734	21	17	14
Chr5	16100001	16200000	921	42	34	18
Chr5	16200001	16300000	1009	35	32	11
Chr5	16300001	16400000	883	24	25	10
Chr5	16400001	16500000	470	20	14	8
Chr5	16500001	16600000	905	10	33	28
Chr5	16600001	16700000	869	32	30	13
Chr5	16700001	16800000	1199	36	37	12
Chr5	16800001	16900000	887	24	18	13
Chr5	16900001	17000000	482	1	17	13
Chr5	17000001	17100000	852	5	22	18
Chr5	17100001	17200000	983	20	26	3
Chr5	17200001	17300000	864	29	26	0
Chr5	17300001	17400000	839	37	35	1
Chr5	17400001	17500000	1057	36	40	0
Chr5	17500001	17600000	1058	54	42	2
Chr5	17600001	17700000	929	40	41	0
Chr5	17700001	17800000	629	20	15	1
Chr5	17800001	17900000	964	42	43	1
Chr5	17900001	18000000	865	39	36	0
Chr5	18000001	18100000	730	47	40	1
Chr5	18100001	18200000	252	17	16	0
Chr5	18200001	18300000	673	17	17	0
Chr5	18300001	18400000	644	21	23	1
Chr5	18400001	18500000	1056	37	38	15
Chr5	18500001	18600000	1256	49	39	19
Chr5	18600001	18700000	1222	32	27	11
Chr5	18700001	18800000	1069	53	33	38
Chr5	18800001	18900000	967	39	33	19
Chr5	18900001	19000000	1203	46	33	17

					InDel	
			All	Bengal/Nona Bokra	PSSR/Bengal	PSSR/Nona Bokra
Chr5	19000001	19100000	736	36	27	22
Chr5	19100001	19200000	970	38	30	25
Chr5	19200001	19300000	992	27	27	21
Chr5	19300001	19400000	1004	31	29	19
Chr5	19400001	19500000	1346	99	69	16
Chr5	19500001	19600000	950	16	18	15
Chr5	19600001	19700000	1025	30	27	12
Chr5	19700001	19800000	463	23	7	25
Chr5	19800001	19900000	681	15	17	13
Chr5	19900001	20000000	828	41	39	0
Chr5	20000001	20100000	972	36	38	1
Chr5	20100001	20200000	859	44	39	0
Chr5	20200001	20300000	828	33	34	0
Chr5	20300001	20400000	908	31	33	1
Chr5	20400001	20500000	879	31	35	7
Chr5	20500001	20600000	533	21	23	0
Chr5	20600001	20700000	1099	44	59	1
Chr5	20700001	20800000	958	39	37	0
Chr5	20800001	20900000	783	31	22	0
Chr5	20900001	21000000	732	49	49	3
Chr5	21000001	21100000	690	43	28	2
Chr5	21100001	21200000	771	36	31	0
Chr5	21200001	21300000	754	33	30	0
Chr5	21300001	21400000	635	1	17	17
Chr5	21400001	21500000	633	10	15	11
Chr5	21500001	21600000	724	40	32	9
Chr5	21600001	21700000	1110	55	46	20
Chr5	21700001	21800000	1002	40	29	21
Chr5	21800001	21900000	507	18	10	1
Chr5	21900001	22000000	690	31	19	9
Chr5	22000001	22100000	895	39	33	15
Chr5	22100001	22200000	816	31	20	12
Chr5	22200001	22300000	1056	42	43	9
Chr5	22300001	22400000	745	38	32	0
Chr5	22400001	22500000	909	35	28	5
Chr5	22500001	22600000	653	33	35	7
Chr5	22600001	22700000	780	43	39	0
Chr5	22700001	22800000	678	38	24	1
Chr5	22800001	22900000	757	26	24	0
Chr5	22900001	23000000	677	39	35	0
Chr5	23000001	23100000	751	33	26	1
Chr5	23100001	23200000	608	28	26	1
Chr5	23200001	23300000	625	25	25	0
Chr5	23300001	23400000	707	29	25	0
Chr5	23400001	23500000	968	43	36	1

					InDel	
			All	Bengal/Nona Bokra	PSSR/Bengal	PSSR/Nona Bokra
Chr5	23500001	23600000	740	41	18	19
Chr5	23600001	23700000	81	7	4	0
Chr5	23700001	23800000	199	8	7	0
Chr5	23800001	23900000	355	19	22	1
Chr5	23900001	24000000	956	51	44	1
Chr5	24000001	24100000	616	46	34	1
Chr5	24100001	24200000	895	31	31	3
Chr5	24200001	24300000	618	9	21	10
Chr5	24300001	24400000	515	29	28	0
Chr5	24400001	24500000	654	30	21	0
Chr5	24500001	24600000	660	37	32	0
Chr5	24600001	24700000	752	43	40	1
Chr5	24700001	24800000	815	41	38	1
Chr5	24800001	24900000	956	29	26	2
Chr5	24900001	25000000	657	28	22	0
Chr5	25000001	25100000	995	49	37	0
Chr5	25100001	25200000	792	34	38	0
Chr5	25200001	25300000	501	25	28	1
Chr5	25300001	25400000	588	35	29	1
Chr5	25400001	25500000	731	29	33	0
Chr5	25500001	25600000	1013	45	38	0
Chr5	25600001	25700000	784	58	59	0
Chr5	25700001	25800000	663	38	36	2
Chr5	25800001	25900000	466	39	25	0
Chr5	25900001	26000000	510	25	25	0
Chr5	26000001	26100000	537	20	17	1
Chr5	26100001	26200000	902	64	47	0
Chr5	26200001	26300000	956	52	30	1
Chr5	26300001	26400000	812	54	42	2
Chr5	26400001	26500000	467	26	26	0
Chr5	26500001	26600000	582	27	27	1
Chr5	26600001	26700000	523	34	29	0
Chr5	26700001	26800000	820	32	30	0
Chr5	26800001	26900000	552	39	41	2
Chr5	26900001	27000000	759	43	42	4
Chr5	27000001	27100000	947	43	25	14
Chr5	27100001	27200000	404	21	12	0
Chr5	27200001	27300000	663	42	31	4
Chr5	27300001	27400000	790	38	38	17
Chr5	27400001	27500000	856	63	48	1
Chr5	27500001	27600000	581	45	32	6
Chr5	27600001	27700000	585	33	26	2
Chr5	27700001	27800000	505	31	24	0
Chr5	27800001	27900000	650	33	30	3
Chr5	27900001	28000000	980	42	40	10

					InDel	
			All	Bengal/Nona Bokra	PSSR/Bengal	PSSR/Nona Bokra
Chr5	28000001	28100000	853	39	43	8
Chr5	28100001	28200000	679	43	33	1
Chr5	28200001	28300000	664	30	23	3
Chr5	28300001	28400000	646	32	26	6
Chr5	28400001	28500000	643	33	22	18
Chr5	28500001	28600000	1019	39	29	17
Chr5	28600001	28700000	592	30	23	10
Chr5	28700001	28800000	758	27	24	11
Chr5	28800001	28900000	692	36	35	8
Chr5	28900001	29000000	596	50	42	6
Chr5	29000001	29100000	843	35	34	6
Chr5	29100001	29200000	903	39	37	13
Chr5	29200001	29300000	517	30	26	2
Chr5	29300001	29400000	529	31	27	3
Chr5	29400001	29500000	789	32	20	0
Chr5	29500001	29600000	673	22	27	9
Chr5	29600001	29700000	163	1	10	8
Chr5	29700001	29800000	28	2	2	0
Chr5	29800001	29900000	94	0	0	0
Chr5	29900001	30000000	40	0	0	0
Chr6	Chr6					
Chr6	1	100000	253	0	0	0
Chr6	100001	200000	725	27	19	0
Chr6	200001	300000	528	26	26	3
Chr6	300001	400000	517	27	15	11
Chr6	400001	500000	849	53	38	24
Chr6	500001	600000	630	23	20	4
Chr6	600001	700000	682	30	29	9
Chr6	700001	800000	938	31	27	24
Chr6	800001	900000	986	45	34	23
Chr6	900001	1000000	813	19	20	18
Chr6	1000001	1100000	675	13	15	7
Chr6	1100001	1200000	744	17	14	18
Chr6	1200001	1300000	627	16	11	9
Chr6	1300001	1400000	561	26	15	10
Chr6	1400001	1500000	783	38	31	33
Chr6	1500001	1600000	568	29	22	20
Chr6	1600001	1700000	838	49	38	28
Chr6	1700001	1800000	767	47	41	12
Chr6	1800001	1900000	716	51	32	9
Chr6	1900001	2000000	568	45	25	9
Chr6	2000001	2100000	319	25	25	1
Chr6	2100001	2200000	777	39	39	0
Chr6	2200001	2300000	541	32	31	1
Chr6	2300001	2400000	831	51	44	2

					InDel	
			All	Bengal/Nona Bokra	PSSR/Bengal	PSSR/Nona Bokra
Chr6	2400001	2500000	462	26	21	0
Chr6	2500001	2600000	619	38	31	0
Chr6	2600001	2700000	470	18	15	1
Chr6	2700001	2800000	594	38	35	2
Chr6	2800001	2900000	792	58	53	1
Chr6	2900001	3000000	628	40	29	0
Chr6	3000001	3100000	1052	73	39	1
Chr6	3100001	3200000	263	19	12	11
Chr6	3200001	3300000	317	7	14	5
Chr6	3300001	3400000	515	41	27	0
Chr6	3400001	3500000	770	15	15	18
Chr6	3500001	3600000	682	10	7	7
Chr6	3600001	3700000	225	10	12	0
Chr6	3700001	3800000	25	3	3	2
Chr6	3800001	3900000	8	0	0	0
Chr6	3900001	4000000	23	0	0	0
Chr6	4000001	4100000	273	21	13	1
Chr6	4100001	4200000	631	31	16	24
Chr6	4200001	4300000	1250	79	56	13
Chr6	4300001	4400000	1722	108	70	2
Chr6	4400001	4500000	574	43	30	3
Chr6	4500001	4600000	707	11	15	12
Chr6	4600001	4700000	930	43	31	16
Chr6	4700001	4800000	974	39	35	26
Chr6	4800001	4900000	747	42	31	0
Chr6	4900001	5000000	444	24	17	0
Chr6	5000001	5100000	605	35	21	0
Chr6	5100001	5200000	937	41	33	1
Chr6	5200001	5300000	140	6	9	0
Chr6	5300001	5400000	48	2	2	1
Chr6	5400001	5500000	34	2	1	0
Chr6	5500001	5600000	263	17	4	10
Chr6	5600001	5700000	1080	51	36	10
Chr6	5700001	5800000	807	48	31	20
Chr6	5800001	5900000	707	30	23	17
Chr6	5900001	6000000	899	34	12	21
Chr6	6000001	6100000	578	31	2	30
Chr6	6100001	6200000	528	28	1	27
Chr6	6200001	6300000	173	10	1	9
Chr6	6300001	6400000	41	4	5	2
Chr6	6400001	6500000	29	1	1	0
Chr6	6500001	6600000	36	1	0	0
Chr6	6600001	6700000	311	2	8	5
Chr6	6700001	6800000	607	5	22	19
Chr6	6800001	6900000	881	12	27	19

					InDel	
			All	Bengal/Nona Bokra	PSSR/Bengal	PSSR/Nona Bokra
Chr6	6900001	7000000	910	17	33	24
Chr6	7000001	7100000	1295	40	39	39
Chr6	7100001	7200000	1228	44	30	47
Chr6	7200001	7300000	1292	35	24	39
Chr6	7300001	7400000	1574	44	10	24
Chr6	7400001	7500000	1391	62	35	41
Chr6	7500001	7600000	867	10	30	24
Chr6	7600001	7700000	822	23	22	24
Chr6	7700001	7800000	939	18	22	14
Chr6	7800001	7900000	743	57	47	0
Chr6	7900001	8000000	867	48	20	28
Chr6	8000001	8100000	1496	69	47	25
Chr6	8100001	8200000	1288	31	33	36
Chr6	8200001	8300000	751	22	17	19
Chr6	8300001	8400000	1046	43	26	31
Chr6	8400001	8500000	936	15	19	10
Chr6	8500001	8600000	851	2	15	2
Chr6	8600001	8700000	1083	18	11	11
Chr6	8700001	8800000	949	31	22	28
Chr6	8800001	8900000	1058	18	22	25
Chr6	8900001	9000000	1685	45	79	50
Chr6	9000001	9100000	1317	19	20	16
Chr6	9100001	9200000	1138	19	35	34
Chr6	9200001	9300000	1645	22	78	63
Chr6	9300001	9400000	1248	59	71	4
Chr6	9400001	9500000	959	29	34	0
Chr6	9500001	9600000	1426	69	74	1
Chr6	9600001	9700000	1174	24	26	21
Chr6	9700001	9800000	281	6	1	3
Chr6	9800001	9900000	1164	18	12	12
Chr6	9900001	10000000	1116	25	21	23
Chr6	10000001	10100000	1321	28	32	23
Chr6	10100001	10200000	1161	17	15	17
Chr6	10200001	10300000	550	12	18	7
Chr6	10300001	10400000	968	21	21	22
Chr6	10400001	10500000	1326	25	8	23
Chr6	10500001	10600000	1020	21	1	18
Chr6	10600001	10700000	848	23	0	19
Chr6	10700001	10800000	798	11	18	19
Chr6	10800001	10900000	771	11	12	10
Chr6	10900001	11000000	481	19	12	16
Chr6	11000001	11100000	962	32	27	10
Chr6	11100001	11200000	1495	47	44	3
Chr6	11200001	11300000	790	40	35	1
Chr6	11300001	11400000	866	32	24	0

					InDel	
			All	Bengal/Nona Bokra	PSSR/Bengal	PSSR/Nona Bokra
Chr6	11400001	11500000	782	38	23	17
Chr6	11500001	11600000	1087	49	16	30
Chr6	11600001	11700000	741	27	25	13
Chr6	11700001	11800000	120	5	3	4
Chr6	11800001	11900000	567	21	18	8
Chr6	11900001	12000000	878	17	23	15
Chr6	12000001	12100000	993	40	33	3
Chr6	12100001	12200000	807	23	27	0
Chr6	12200001	12300000	914	33	26	1
Chr6	12300001	12400000	654	28	27	1
Chr6	12400001	12500000	606	38	34	0
Chr6	12500001	12600000	560	12	7	0
Chr6	12600001	12700000	849	45	32	0
Chr6	12700001	12800000	781	22	23	1
Chr6	12800001	12900000	1158	31	36	0
Chr6	12900001	13000000	1090	38	36	0
Chr6	13000001	13100000	1015	43	38	1
Chr6	13100001	13200000	886	37	39	14
Chr6	13200001	13300000	1120	28	27	20
Chr6	13300001	13400000	935	35	37	2
Chr6	13400001	13500000	1074	38	33	1
Chr6	13500001	13600000	962	38	43	0
Chr6	13600001	13700000	970	49	44	0
Chr6	13700001	13800000	831	46	39	0
Chr6	13800001	13900000	753	24	20	1
Chr6	13900001	14000000	1082	38	36	8
Chr6	14000001	14100000	1252	40	33	10
Chr6	14100001	14200000	1032	15	13	4
Chr6	14200001	14300000	1192	28	24	3
Chr6	14300001	14400000	1009	35	31	14
Chr6	14400001	14500000	1135	27	25	5
Chr6	14500001	14600000	976	23	25	6
Chr6	14600001	14700000	559	8	7	2
Chr6	14700001	14800000	1053	20	18	6
Chr6	14800001	14900000	1228	17	12	3
Chr6	14900001	15000000	817	15	14	6
Chr6	15000001	15100000	1078	8	15	5
Chr6	15100001	15200000	830	22	22	6
Chr6	15200001	15300000	1288	10	21	9
Chr6	15300001	15400000	1119	24	21	8
Chr6	15400001	15500000	387	10	7	4
Chr6	15500001	15600000	1294	16	12	4
Chr6	15600001	15700000	1259	27	17	9
Chr6	15700001	15800000	1045	34	28	14
Chr6	15800001	15900000	1142	35	26	13

					InDel	
			All	Bengal/Nona Bokra	PSSR/Bengal	PSSR/Nona Bokra
Chr6	15900001	16000000	886	20	17	7
Chr6	16000001	16100000	1130	38	39	10
Chr6	16100001	16200000	1322	10	13	5
Chr6	16200001	16300000	1192	25	21	18
Chr6	16300001	16400000	1180	24	23	18
Chr6	16400001	16500000	1621	45	22	42
Chr6	16500001	16600000	1464	44	26	41
Chr6	16600001	16700000	918	21	23	13
Chr6	16700001	16800000	813	30	23	18
Chr6	16800001	16900000	983	44	36	23
Chr6	16900001	17000000	1119	25	28	23
Chr6	17000001	17100000	937	26	23	8
Chr6	17100001	17200000	1078	48	39	18
Chr6	17200001	17300000	1309	43	38	15
Chr6	17300001	17400000	1110	25	25	6
Chr6	17400001	17500000	1098	28	20	21
Chr6	17500001	17600000	937	32	35	4
Chr6	17600001	17700000	748	29	19	0
Chr6	17700001	17800000	1120	43	36	7
Chr6	17800001	17900000	900	25	22	9
Chr6	17900001	18000000	762	56	47	3
Chr6	18000001	18100000	389	16	15	0
Chr6	18100001	18200000	762	25	25	1
Chr6	18200001	18300000	225	9	4	0
Chr6	18300001	18400000	60	0	0	0
Chr6	18400001	18500000	60	2	2	0
Chr6	18500001	18600000	228	0	1	0
Chr6	18600001	18700000	48	1	0	0
Chr6	18700001	18800000	216	4	3	0
Chr6	18800001	18900000	697	25	19	1
Chr6	18900001	19000000	523	17	13	0
Chr6	19000001	19100000	297	15	13	0
Chr6	19100001	19200000	392	15	12	0
Chr6	19200001	19300000	356	11	10	0
Chr6	19300001	19400000	852	37	39	1
Chr6	19400001	19500000	833	45	45	0
Chr6	19500001	19600000	687	21	24	11
Chr6	19600001	19700000	920	34	25	16
Chr6	19700001	19800000	851	36	27	10
Chr6	19800001	19900000	675	13	20	6
Chr6	19900001	20000000	1359	52	43	0
Chr6	20000001	20100000	790	41	36	4
Chr6	20100001	20200000	874	45	42	0
Chr6	20200001	20300000	645	35	33	0
Chr6	20300001	20400000	370	15	11	3

					InDel	
			All	Bengal/Nona Bokra	PSSR/Bengal	PSSR/Nona Bokra
Chr6	20400001	20500000	978	41	36	0
Chr6	20500001	20600000	887	13	14	3
Chr6	20600001	20700000	688	15	13	6
Chr6	20700001	20800000	1264	37	25	28
Chr6	20800001	20900000	947	36	29	11
Chr6	20900001	21000000	1144	39	31	19
Chr6	21000001	21100000	1132	54	45	10
Chr6	21100001	21200000	678	43	40	0
Chr6	21200001	21300000	1170	40	26	19
Chr6	21300001	21400000	1301	42	44	17
Chr6	21400001	21500000	718	16	22	17
Chr6	21500001	21600000	1102	18	31	26
Chr6	21600001	21700000	792	2	23	20
Chr6	21700001	21800000	117	2	5	5
Chr6	21800001	21900000	25	3	5	2
Chr6	21900001	22000000	113	2	2	2
Chr6	22000001	22100000	985	4	30	23
Chr6	22100001	22200000	923	27	33	3
Chr6	22200001	22300000	656	43	32	20
Chr6	22300001	22400000	895	18	17	23
Chr6	22400001	22500000	693	9	5	6
Chr6	22500001	22600000	650	7	4	5
Chr6	22600001	22700000	1166	22	18	14
Chr6	22700001	22800000	578	2	2	4
Chr6	22800001	22900000	1562	33	38	11
Chr6	22900001	23000000	696	3	2	1
Chr6	23000001	23100000	985	22	21	17
Chr6	23100001	23200000	1385	66	56	23
Chr6	23200001	23300000	899	22	20	8
Chr6	23300001	23400000	1321	49	42	14
Chr6	23400001	23500000	1527	51	53	0
Chr6	23500001	23600000	746	19	28	5
Chr6	23600001	23700000	948	29	41	8
Chr6	23700001	23800000	753	30	21	14
Chr6	23800001	23900000	837	35	20	10
Chr6	23900001	24000000	683	24	20	1
Chr6	24000001	24100000	539	35	8	24
Chr6	24100001	24200000	291	26	4	18
Chr6	24200001	24300000	507	27	2	25
Chr6	24300001	24400000	654	39	3	39
Chr6	24400001	24500000	691	49	39	14
Chr6	24500001	24600000	645	39	34	1
Chr6	24600001	24700000	1006	31	26	1
Chr6	24700001	24800000	784	41	35	1
Chr6	24800001	24900000	559	41	26	1

					InDel	
			All	Bengal/Nona Bokra	PSSR/Bengal	PSSR/Nona Bokra
Chr6	24900001	25000000	862	43	29	1
Chr6	25000001	25100000	761	46	40	0
Chr6	25100001	25200000	585	27	25	0
Chr6	25200001	25300000	251	8	8	0
Chr6	25300001	25400000	355	18	16	0
Chr6	25400001	25500000	364	17	17	0
Chr6	25500001	25600000	342	16	15	0
Chr6	25600001	25700000	513	17	17	0
Chr6	25700001	25800000	728	23	24	0
Chr6	25800001	25900000	603	37	32	0
Chr6	25900001	26000000	687	43	38	0
Chr6	26000001	26100000	946	51	49	3
Chr6	26100001	26200000	915	29	33	0
Chr6	26200001	26300000	748	34	29	0
Chr6	26300001	26400000	740	46	41	0
Chr6	26400001	26500000	776	48	42	0
Chr6	26500001	26600000	997	64	68	0
Chr6	26600001	26700000	866	50	43	0
Chr6	26700001	26800000	1029	40	41	0
Chr6	26800001	26900000	809	48	44	0
Chr6	26900001	27000000	933	28	29	0
Chr6	27000001	27100000	1272	22	21	3
Chr6	27100001	27200000	843	46	33	12
Chr6	27200001	27300000	783	26	22	15
Chr6	27300001	27400000	767	36	39	25
Chr6	27400001	27500000	1012	53	50	35
Chr6	27500001	27600000	863	33	28	27
Chr6	27600001	27700000	135	13	1	11
Chr6	27700001	27800000	230	0	5	3
Chr6	27800001	27900000	826	0	32	28
Chr6	27900001	28000000	673	9	13	11
Chr6	28000001	28100000	1113	51	38	26
Chr6	28100001	28200000	805	29	30	8
Chr6	28200001	28300000	783	32	24	12
Chr6	28300001	28400000	934	55	49	9
Chr6	28400001	28500000	716	36	25	13
Chr6	28500001	28600000	999	31	40	20
Chr6	28600001	28700000	890	45	29	25
Chr6	28700001	28800000	715	48	40	6
Chr6	28800001	28900000	794	40	28	9
Chr6	28900001	29000000	840	46	42	11
Chr6	29000001	29100000	822	22	20	14
Chr6	29100001	29200000	780	5	27	25
Chr6	29200001	29300000	359	1	12	9
Chr6	29300001	29400000	371	0	15	13

					InDel	
			All	Bengal/Nona Bokra	PSSR/Bengal	PSSR/Nona Bokra
Chr6	29400001	29500000	582	2	33	32
Chr6	29500001	29600000	703	34	31	6
Chr6	29600001	29700000	1010	24	25	2
Chr6	29700001	29800000	811	21	22	22
Chr6	29800001	29900000	904	22	19	14
Chr6	29900001	30000000	390	12	6	1
Chr6	30000001	30100000	732	24	21	2
Chr6	30100001	30200000	775	18	18	4
Chr6	30200001	30300000	334	12	11	1
Chr6	30300001	30400000	534	11	8	1
Chr6	30400001	30500000	601	13	9	1
Chr6	30500001	30600000	345	7	0	6
Chr6	30600001	30700000	26	0	0	0
Chr6	30700001	30800000	274	1	17	18
Chr6	30800001	30900000	501	3	2	3
Chr6	30900001	31000000	707	0	0	0
Chr6	31000001	31100000	563	1	1	0
Chr6	31100001	31200000	469	0	0	0
Chr6	31200001	31300000	168	1	0	0
Chr7	Chr7					
Chr7	1	100000	631	33	32	1
Chr7	100001	200000	870	42	31	0
Chr7	200001	300000	943	22	25	0
Chr7	300001	400000	489	20	23	0
Chr7	400001	500000	748	49	30	9
Chr7	500001	600000	615	36	21	10
Chr7	600001	700000	1107	30	30	19
Chr7	700001	800000	932	67	43	26
Chr7	800001	900000	709	36	33	0
Chr7	900001	1000000	1097	55	40	0
Chr7	1000001	1100000	652	38	29	0
Chr7	1100001	1200000	850	38	32	12
Chr7	1200001	1300000	741	46	40	26
Chr7	1300001	1400000	136	10	7	0
Chr7	1400001	1500000	414	23	21	1
Chr7	1500001	1600000	992	64	44	1
Chr7	1600001	1700000	937	22	11	12
Chr7	1700001	1800000	1215	50	32	5
Chr7	1800001	1900000	656	36	31	0
Chr7	1900001	2000000	884	42	44	1
Chr7	2000001	2100000	905	23	20	10
Chr7	2100001	2200000	733	24	21	13
Chr7	2200001	2300000	929	29	26	15
Chr7	2300001	2400000	710	23	12	6
Chr7	2400001	2500000	475	24	22	0

					InDel	
			All	Bengal/Nona Bokra	PSSR/Bengal	PSSR/Nona Bokra
Chr7	2500001	2600000	918	37	31	1
Chr7	2600001	2700000	933	61	53	1
Chr7	2700001	2800000	165	11	6	0
Chr7	2800001	2900000	33	1	2	2
Chr7	2900001	3000000	26	0	1	0
Chr7	3000001	3100000	16	0	0	0
Chr7	3100001	3200000	541	0	0	0
Chr7	3200001	3300000	365	15	17	0
Chr7	3300001	3400000	599	47	3	47
Chr7	3400001	3500000	412	16	1	16
Chr7	3500001	3600000	42	3	2	1
Chr7	3600001	3700000	44	1	1	0
Chr7	3700001	3800000	67	2	3	2
Chr7	3800001	3900000	372	4	1	3
Chr7	3900001	4000000	515	0	0	0
Chr7	4000001	4100000	80	5	5	2
Chr7	4100001	4200000	632	41	39	1
Chr7	4200001	4300000	727	49	4	46
Chr7	4300001	4400000	740	47	3	47
Chr7	4400001	4500000	491	27	0	27
Chr7	4500001	4600000	719	48	4	46
Chr7	4600001	4700000	937	30	30	7
Chr7	4700001	4800000	908	40	11	32
Chr7	4800001	4900000	669	24	23	5
Chr7	4900001	5000000	775	16	19	13
Chr7	5000001	5100000	904	44	46	6
Chr7	5100001	5200000	787	20	34	21
Chr7	5200001	5300000	531	16	13	1
Chr7	5300001	5400000	893	43	34	1
Chr7	5400001	5500000	821	36	33	2
Chr7	5500001	5600000	712	41	31	0
Chr7	5600001	5700000	787	20	42	21
Chr7	5700001	5800000	664	5	24	14
Chr7	5800001	5900000	771	54	10	45
Chr7	5900001	6000000	538	31	1	30
Chr7	6000001	6100000	843	49	31	24
Chr7	6100001	6200000	664	23	15	1
Chr7	6200001	6300000	1008	52	31	3
Chr7	6300001	6400000	451	10	7	2
Chr7	6400001	6500000	371	8	5	0
Chr7	6500001	6600000	102	0	0	0
Chr7	6600001	6700000	773	36	28	0
Chr7	6700001	6800000	926	33	23	1
Chr7	6800001	6900000	1079	61	46	2
Chr7	6900001	7000000	938	37	34	11

					InDel	
			All	Bengal/Nona Bokra	PSSR/Bengal	PSSR/Nona Bokra
Chr7	7000001	7100000	983	30	22	0
Chr7	7100001	7200000	906	38	39	4
Chr7	7200001	7300000	935	44	36	8
Chr7	7300001	7400000	1097	74	46	0
Chr7	7400001	7500000	877	30	23	0
Chr7	7500001	7600000	1127	31	26	0
Chr7	7600001	7700000	907	36	33	0
Chr7	7700001	7800000	885	38	31	9
Chr7	7800001	7900000	870	19	19	2
Chr7	7900001	8000000	906	29	27	11
Chr7	8000001	8100000	1086	36	36	7
Chr7	8100001	8200000	1056	30	15	18
Chr7	8200001	8300000	683	8	7	1
Chr7	8300001	8400000	1249	44	39	1
Chr7	8400001	8500000	1060	35	32	2
Chr7	8500001	8600000	1218	31	30	22
Chr7	8600001	8700000	1148	20	16	17
Chr7	8700001	8800000	1158	23	20	11
Chr7	8800001	8900000	964	32	25	8
Chr7	8900001	9000000	1044	20	20	16
Chr7	9000001	9100000	913	51	31	10
Chr7	9100001	9200000	887	22	21	0
Chr7	9200001	9300000	1032	37	27	0
Chr7	9300001	9400000	1013	36	33	0
Chr7	9400001	9500000	795	34	32	0
Chr7	9500001	9600000	723	21	22	2
Chr7	9600001	9700000	298	1	2	0
Chr7	9700001	9800000	390	4	5	0
Chr7	9800001	9900000	479	7	8	0
Chr7	9900001	10000000	391	14	12	7
Chr7	10000001	10100000	647	28	19	2
Chr7	10100001	10200000	472	10	7	0
Chr7	10200001	10300000	879	41	38	0
Chr7	10300001	10400000	463	17	18	0
Chr7	10400001	10500000	571	3	5	0
Chr7	10500001	10600000	858	10	12	0
Chr7	10600001	10700000	803	19	16	0
Chr7	10700001	10800000	1017	33	31	1
Chr7	10800001	10900000	529	16	17	0
Chr7	10900001	11000000	766	25	23	0
Chr7	11000001	11100000	767	27	22	0
Chr7	11100001	11200000	634	27	22	0
Chr7	11200001	11300000	1015	50	36	1
Chr7	11300001	11400000	1137	58	56	0
Chr7	11400001	11500000	586	12	16	0

					InDel	
			All	Bengal/Nona Bokra	PSSR/Bengal	PSSR/Nona Bokra
Chr7	11500001	11600000	1229	66	53	0
Chr7	11600001	11700000	1009	46	39	0
Chr7	11700001	11800000	608	12	11	0
Chr7	11800001	11900000	505	14	20	2
Chr7	11900001	12000000	714	14	16	1
Chr7	12000001	12100000	484	5	5	0
Chr7	12100001	12200000	328	3	2	0
Chr7	12200001	12300000	167	0	0	0
Chr7	12300001	12400000	633	20	16	0
Chr7	12400001	12500000	319	7	5	0
Chr7	12500001	12600000	520	11	10	0
Chr7	12600001	12700000	471	18	15	1
Chr7	12700001	12800000	493	17	19	0
Chr7	12800001	12900000	621	24	22	1
Chr7	12900001	13000000	431	30	22	0
Chr7	13000001	13100000	973	45	33	0
Chr7	13100001	13200000	740	24	17	0
Chr7	13200001	13300000	1140	48	50	0
Chr7	13300001	13400000	1368	53	46	0
Chr7	13400001	13500000	503	24	18	1
Chr7	13500001	13600000	474	26	21	1
Chr7	13600001	13700000	1089	39	36	1
Chr7	13700001	13800000	762	37	41	0
Chr7	13800001	13900000	529	13	9	0
Chr7	13900001	14000000	96	0	1	0
Chr7	14000001	14100000	87	1	0	0
Chr7	14100001	14200000	139	2	2	0
Chr7	14200001	14300000	98	0	0	0
Chr7	14300001	14400000	193	2	3	0
Chr7	14400001	14500000	300	0	1	0
Chr7	14500001	14600000	1025	65	48	0
Chr7	14600001	14700000	1248	50	52	17
Chr7	14700001	14800000	989	26	35	14
Chr7	14800001	14900000	296	5	4	3
Chr7	14900001	15000000	1256	33	24	4
Chr7	15000001	15100000	847	30	23	17
Chr7	15100001	15200000	590	9	7	3
Chr7	15200001	15300000	936	44	35	1
Chr7	15300001	15400000	1999	88	68	1
Chr7	15400001	15500000	1378	33	6	12
Chr7	15500001	15600000	1459	29	21	19
Chr7	15600001	15700000	1350	33	15	25
Chr7	15700001	15800000	1335	39	38	17
Chr7	15800001	15900000	1392	33	35	8
Chr7	15900001	16000000	888	36	28	0

					InDel	
			All	Bengal/Nona Bokra	PSSR/Bengal	PSSR/Nona Bokra
Chr7	16000001	16100000	1144	35	31	17
Chr7	16100001	16200000	1351	28	18	11
Chr7	16200001	16300000	1177	28	32	19
Chr7	16300001	16400000	1435	39	31	25
Chr7	16400001	16500000	1113	35	22	23
Chr7	16500001	16600000	1199	26	24	18
Chr7	16600001	16700000	1138	54	41	20
Chr7	16700001	16800000	867	21	10	22
Chr7	16800001	16900000	703	30	29	17
Chr7	16900001	17000000	833	45	36	7
Chr7	17000001	17100000	630	24	19	1
Chr7	17100001	17200000	799	41	36	6
Chr7	17200001	17300000	893	49	39	13
Chr7	17300001	17400000	1217	39	32	19
Chr7	17400001	17500000	927	27	28	13
Chr7	17500001	17600000	584	3	14	10
Chr7	17600001	17700000	581	1	18	8
Chr7	17700001	17800000	826	1	23	21
Chr7	17800001	17900000	582	1	22	18
Chr7	17900001	18000000	220	2	7	6
Chr7	18000001	18100000	314	1	11	8
Chr7	18100001	18200000	801	7	20	17
Chr7	18200001	18300000	466	15	7	14
Chr7	18300001	18400000	754	24	21	18
Chr7	18400001	18500000	1499	24	34	11
Chr7	18500001	18600000	1070	38	31	19
Chr7	18600001	18700000	804	28	19	16
Chr7	18700001	18800000	1213	22	24	17
Chr7	18800001	18900000	1180	38	20	35
Chr7	18900001	19000000	1111	27	30	13
Chr7	19000001	19100000	1265	24	23	12
Chr7	19100001	19200000	1202	24	28	0
Chr7	19200001	19300000	767	18	17	1
Chr7	19300001	19400000	1167	24	27	9
Chr7	19400001	19500000	1210	20	22	13
Chr7	19500001	19600000	1083	24	32	21
Chr7	19600001	19700000	910	25	19	12
Chr7	19700001	19800000	1026	20	19	1
Chr7	19800001	19900000	822	17	19	0
Chr7	19900001	20000000	1000	22	19	0
Chr7	20000001	20100000	1222	38	24	0
Chr7	20100001	20200000	1247	10	10	0
Chr7	20200001	20300000	662	12	14	0
Chr7	20300001	20400000	979	17	19	0
Chr7	20400001	20500000	918	36	38	0

					InDel	
			All	Bengal/Nona Bokra	PSSR/Bengal	PSSR/Nona Bokra
Chr7	20500001	20600000	1625	20	27	0
Chr7	20600001	20700000	1008	26	26	0
Chr7	20700001	20800000	1234	32	34	1
Chr7	20800001	20900000	1051	9	8	3
Chr7	20900001	21000000	1623	43	51	43
Chr7	21000001	21100000	1093	7	16	12
Chr7	21100001	21200000	1161	35	23	14
Chr7	21200001	21300000	934	31	17	22
Chr7	21300001	21400000	1646	27	16	15
Chr7	21400001	21500000	1454	41	14	32
Chr7	21500001	21600000	615	32	19	8
Chr7	21600001	21700000	1226	77	70	3
Chr7	21700001	21800000	1267	69	54	8
Chr7	21800001	21900000	1106	49	38	31
Chr7	21900001	22000000	745	45	25	0
Chr7	22000001	22100000	763	35	29	0
Chr7	22100001	22200000	614	31	27	8
Chr7	22200001	22300000	907	34	31	8
Chr7	22300001	22400000	902	33	33	13
Chr7	22400001	22500000	1063	17	29	17
Chr7	22500001	22600000	914	38	31	14
Chr7	22600001	22700000	472	28	30	13
Chr7	22700001	22800000	703	33	31	9
Chr7	22800001	22900000	758	34	29	4
Chr7	22900001	23000000	1160	36	36	39
Chr7	23000001	23100000	660	23	17	10
Chr7	23100001	23200000	785	30	32	16
Chr7	23200001	23300000	904	39	28	9
Chr7	23300001	23400000	987	19	20	0
Chr7	23400001	23500000	981	30	30	2
Chr7	23500001	23600000	765	33	31	1
Chr7	23600001	23700000	811	35	34	0
Chr7	23700001	23800000	853	36	31	0
Chr7	23800001	23900000	685	44	38	0
Chr7	23900001	24000000	654	28	23	0
Chr7	24000001	24100000	972	48	43	0
Chr7	24100001	24200000	772	25	25	0
Chr7	24200001	24300000	809	32	30	7
Chr7	24300001	24400000	836	29	26	13
Chr7	24400001	24500000	1295	78	87	1
Chr7	24500001	24600000	1429	71	56	0
Chr7	24600001	24700000	1021	33	39	1
Chr7	24700001	24800000	933	50	49	13
Chr7	24800001	24900000	430	17	14	2
Chr7	24900001	25000000	775	40	43	1

					InDel	
			All	Bengal/Nona Bokra	PSSR/Bengal	PSSR/Nona Bokra
Chr7	25000001	25100000	321	25	15	0
Chr7	25100001	25200000	402	23	9	0
Chr7	25200001	25300000	747	46	33	0
Chr7	25300001	25400000	846	46	45	13
Chr7	25400001	25500000	769	41	43	4
Chr7	25500001	25600000	787	37	31	0
Chr7	25600001	25700000	704	34	27	7
Chr7	25700001	25800000	837	46	40	8
Chr7	25800001	25900000	743	25	26	9
Chr7	25900001	26000000	791	42	45	6
Chr7	26000001	26100000	1281	53	45	16
Chr7	26100001	26200000	708	26	23	6
Chr7	26200001	26300000	742	43	36	1
Chr7	26300001	26400000	829	54	49	0
Chr7	26400001	26500000	669	43	37	0
Chr7	26500001	26600000	582	21	25	1
Chr7	26600001	26700000	513	38	34	1
Chr7	26700001	26800000	1276	69	52	9
Chr7	26800001	26900000	867	57	41	6
Chr7	26900001	27000000	382	26	19	6
Chr7	27000001	27100000	531	10	8	8
Chr7	27100001	27200000	666	25	10	9
Chr7	27200001	27300000	215	2	0	0
Chr7	27300001	27400000	164	5	1	0
Chr7	27400001	27500000	554	15	4	6
Chr7	27500001	27600000	1093	54	41	12
Chr7	27600001	27700000	807	46	40	20
Chr7	27700001	27800000	1159	91	75	5
Chr7	27800001	27900000	726	45	38	13
Chr7	27900001	28000000	1053	58	41	19
Chr7	28000001	28100000	861	45	10	37
Chr7	28100001	28200000	208	18	1	17
Chr7	28200001	28300000	29	4	3	2
Chr7	28300001	28400000	563	21	5	15
Chr7	28400001	28500000	787	29	28	19
Chr7	28500001	28600000	569	37	31	0
Chr7	28600001	28700000	536	26	24	0
Chr7	28700001	28800000	748	23	33	19
Chr7	28800001	28900000	732	29	16	21
Chr7	28900001	29000000	765	30	27	11
Chr7	29000001	29100000	681	47	39	0
Chr7	29100001	29200000	635	36	30	1
Chr7	29200001	29300000	625	38	32	13
Chr7	29300001	29400000	873	38	21	22
Chr7	29400001	29500000	838	37	35	1

					InDel	
			All	Bengal/Nona Bokra	PSSR/Bengal	PSSR/Nona Bokra
Chr7	29500001	29600000	808	39	30	21
Chr7	29600001	29700000	817	20	13	5
Chr8	Chr8					
Chr8	1	100000	738	26	27	0
Chr8	100001	200000	861	27	31	0
Chr8	200001	300000	988	39	21	11
Chr8	300001	400000	803	34	18	29
Chr8	400001	500000	671	10	15	16
Chr8	500001	600000	511	3	15	14
Chr8	600001	700000	575	4	18	15
Chr8	700001	800000	793	4	29	23
Chr8	800001	900000	724	17	37	25
Chr8	900001	1000000	171	0	5	4
Chr8	1000001	1100000	603	31	3	30
Chr8	1100001	1200000	759	20	20	16
Chr8	1200001	1300000	458	9	9	7
Chr8	1300001	1400000	457	10	3	4
Chr8	1400001	1500000	482	3	3	2
Chr8	1500001	1600000	1302	50	36	33
Chr8	1600001	1700000	1004	39	35	20
Chr8	1700001	1800000	849	37	37	12
Chr8	1800001	1900000	731	7	4	5
Chr8	1900001	2000000	680	13	11	14
Chr8	2000001	2100000	1268	61	42	24
Chr8	2100001	2200000	851	54	33	14
Chr8	2200001	2300000	906	46	42	4
Chr8	2300001	2400000	828	36	31	14
Chr8	2400001	2500000	478	29	7	21
Chr8	2500001	2600000	177	7	1	7
Chr8	2600001	2700000	368	11	8	1
Chr8	2700001	2800000	562	14	15	1
Chr8	2800001	2900000	697	31	19	1
Chr8	2900001	3000000	1163	81	40	27
Chr8	3000001	3100000	896	51	19	48
Chr8	3100001	3200000	648	22	23	18
Chr8	3200001	3300000	841	29	34	26
Chr8	3300001	3400000	804	35	33	4
Chr8	3400001	3500000	966	51	41	0
Chr8	3500001	3600000	926	49	46	14
Chr8	3600001	3700000	742	32	31	1
Chr8	3700001	3800000	640	12	6	4
Chr8	3800001	3900000	626	14	22	3
Chr8	3900001	4000000	885	34	34	0
Chr8	4000001	4100000	975	48	33	1
Chr8	4100001	4200000	590	19	22	0

					InDel	
			All	Bengal/Nona Bokra	PSSR/Bengal	PSSR/Nona Bokra
Chr8	4200001	4300000	730	36	28	1
Chr8	4300001	4400000	1455	56	44	3
Chr8	4400001	4500000	504	26	22	0
Chr8	4500001	4600000	648	44	37	2
Chr8	4600001	4700000	763	35	24	17
Chr8	4700001	4800000	868	66	54	1
Chr8	4800001	4900000	525	28	12	0
Chr8	4900001	5000000	320	10	9	0
Chr8	5000001	5100000	851	42	36	2
Chr8	5100001	5200000	695	33	32	2
Chr8	5200001	5300000	995	46	37	6
Chr8	5300001	5400000	663	35	22	13
Chr8	5400001	5500000	677	44	39	1
Chr8	5500001	5600000	775	26	23	5
Chr8	5600001	5700000	1421	49	28	26
Chr8	5700001	5800000	998	36	32	29
Chr8	5800001	5900000	725	38	15	28
Chr8	5900001	6000000	756	12	9	11
Chr8	6000001	6100000	991	30	26	20
Chr8	6100001	6200000	1052	33	22	17
Chr8	6200001	6300000	777	31	30	9
Chr8	6300001	6400000	299	3	3	1
Chr8	6400001	6500000	316	0	6	1
Chr8	6500001	6600000	309	3	5	2
Chr8	6600001	6700000	135	1	0	1
Chr8	6700001	6800000	187	1	2	1
Chr8	6800001	6900000	151	3	0	2
Chr8	6900001	7000000	172	0	0	0
Chr8	7000001	7100000	154	0	0	0
Chr8	7100001	7200000	624	2	1	1
Chr8	7200001	7300000	523	9	5	5
Chr8	7300001	7400000	513	19	6	20
Chr8	7400001	7500000	929	27	21	15
Chr8	7500001	7600000	445	13	18	2
Chr8	7600001	7700000	714	25	25	0
Chr8	7700001	7800000	1054	56	40	0
Chr8	7800001	7900000	767	41	30	1
Chr8	7900001	8000000	845	52	43	1
Chr8	8000001	8100000	810	20	17	0
Chr8	8100001	8200000	556	28	23	0
Chr8	8200001	8300000	1072	61	48	0
Chr8	8300001	8400000	713	59	42	0
Chr8	8400001	8500000	830	49	28	16
Chr8	8500001	8600000	1137	59	16	59
Chr8	8600001	8700000	998	46	51	13

					InDel	
			All	Bengal/Nona Bokra	PSSR/Bengal	PSSR/Nona Bokra
Chr8	8700001	8800000	934	46	44	0
Chr8	8800001	8900000	1181	74	47	0
Chr8	8900001	9000000	1016	47	40	9
Chr8	9000001	9100000	1331	55	39	22
Chr8	9100001	9200000	935	37	40	1
Chr8	9200001	9300000	599	18	19	3
Chr8	9300001	9400000	1042	27	27	17
Chr8	9400001	9500000	1200	64	39	8
Chr8	9500001	9600000	639	18	14	10
Chr8	9600001	9700000	747	22	19	16
Chr8	9700001	9800000	809	29	11	13
Chr8	9800001	9900000	849	28	16	13
Chr8	9900001	10000000	931	13	15	12
Chr8	10000001	10100000	1111	29	28	16
Chr8	10100001	10200000	1003	33	23	25
Chr8	10200001	10300000	1024	29	29	23
Chr8	10300001	10400000	1086	30	28	11
Chr8	10400001	10500000	1057	25	25	12
Chr8	10500001	10600000	1163	19	18	8
Chr8	10600001	10700000	1196	29	26	19
Chr8	10700001	10800000	1182	31	22	19
Chr8	10800001	10900000	1112	29	24	17
Chr8	10900001	11000000	984	17	21	8
Chr8	11000001	11100000	600	17	14	11
Chr8	11100001	11200000	473	2	10	5
Chr8	11200001	11300000	458	14	7	11
Chr8	11300001	11400000	1060	19	15	15
Chr8	11400001	11500000	856	10	13	6
Chr8	11500001	11600000	762	25	17	17
Chr8	11600001	11700000	678	10	8	6
Chr8	11700001	11800000	902	23	21	16
Chr8	11800001	11900000	1285	28	22	20
Chr8	11900001	12000000	1372	26	16	24
Chr8	12000001	12100000	1328	27	9	16
Chr8	12100001	12200000	527	8	10	7
Chr8	12200001	12300000	309	16	7	11
Chr8	12300001	12400000	587	12	12	13
Chr8	12400001	12500000	458	9	5	3
Chr8	12500001	12600000	327	5	3	1
Chr8	12600001	12700000	492	4	1	1
Chr8	12700001	12800000	427	8	8	7
Chr8	12800001	12900000	563	11	12	12
Chr8	12900001	13000000	299	0	2	1
Chr8	13000001	13100000	451	4	6	5
Chr8	13100001	13200000	331	3	4	2

					InDel	
			All	Bengal/Nona Bokra	PSSR/Bengal	PSSR/Nona Bokra
Chr8	13200001	13300000	54	0	0	0
Chr8	13300001	13400000	301	4	6	5
Chr8	13400001	13500000	473	3	4	1
Chr8	13500001	13600000	584	11	9	7
Chr8	13600001	13700000	583	7	6	6
Chr8	13700001	13800000	468	9	7	6
Chr8	13800001	13900000	392	2	4	2
Chr8	13900001	14000000	478	15	19	13
Chr8	14000001	14100000	552	5	15	8
Chr8	14100001	14200000	455	15	11	10
Chr8	14200001	14300000	595	8	7	8
Chr8	14300001	14400000	401	14	14	17
Chr8	14400001	14500000	537	20	11	17
Chr8	14500001	14600000	586	6	7	5
Chr8	14600001	14700000	567	9	9	6
Chr8	14700001	14800000	487	12	10	13
Chr8	14800001	14900000	200	1	4	2
Chr8	14900001	15000000	811	10	22	10
Chr8	15000001	15100000	1233	25	44	41
Chr8	15100001	15200000	697	10	10	9
Chr8	15200001	15300000	491	22	18	10
Chr8	15300001	15400000	882	29	26	20
Chr8	15400001	15500000	790	25	28	10
Chr8	15500001	15600000	719	24	24	1
Chr8	15600001	15700000	826	40	37	1
Chr8	15700001	15800000	544	19	24	0
Chr8	15800001	15900000	666	13	13	0
Chr8	15900001	16000000	919	20	21	1
Chr8	16000001	16100000	617	11	11	1
Chr8	16100001	16200000	548	2	2	0
Chr8	16200001	16300000	183	0	0	0
Chr8	16300001	16400000	1506	89	67	0
Chr8	16400001	16500000	833	18	15	7
Chr8	16500001	16600000	713	26	27	24
Chr8	16600001	16700000	1205	13	25	21
Chr8	16700001	16800000	805	16	7	5
Chr8	16800001	16900000	959	31	9	18
Chr8	16900001	17000000	1522	52	48	20
Chr8	17000001	17100000	942	15	15	6
Chr8	17100001	17200000	1427	25	29	22
Chr8	17200001	17300000	721	18	11	16
Chr8	17300001	17400000	1381	35	12	25
Chr8	17400001	17500000	754	13	10	9
Chr8	17500001	17600000	1334	50	21	37
Chr8	17600001	17700000	1541	44	41	1

					InDel	
			All	Bengal/Nona Bokra	PSSR/Bengal	PSSR/Nona Bokra
Chr8	17700001	17800000	517	9	8	0
Chr8	17800001	17900000	1371	36	34	0
Chr8	17900001	18000000	817	13	16	0
Chr8	18000001	18100000	1402	16	17	0
Chr8	18100001	18200000	775	20	22	1
Chr8	18200001	18300000	863	23	23	0
Chr8	18300001	18400000	890	35	27	0
Chr8	18400001	18500000	508	29	25	0
Chr8	18500001	18600000	876	22	21	8
Chr8	18600001	18700000	1446	34	19	16
Chr8	18700001	18800000	1068	26	13	14
Chr8	18800001	18900000	1078	36	14	17
Chr8	18900001	19000000	843	46	27	5
Chr8	19000001	19100000	1136	49	36	21
Chr8	19100001	19200000	932	35	25	12
Chr8	19200001	19300000	1087	28	31	6
Chr8	19300001	19400000	1206	39	25	32
Chr8	19400001	19500000	1152	40	33	14
Chr8	19500001	19600000	1015	36	38	5
Chr8	19600001	19700000	1402	48	25	24
Chr8	19700001	19800000	1135	40	21	27
Chr8	19800001	19900000	1518	59	37	43
Chr8	19900001	20000000	1097	48	34	34
Chr8	20000001	20100000	1221	49	23	38
Chr8	20100001	20200000	1158	49	41	8
Chr8	20200001	20300000	1136	63	14	62
Chr8	20300001	20400000	762	25	18	12
Chr8	20400001	20500000	1058	38	33	10
Chr8	20500001	20600000	667	29	23	4
Chr8	20600001	20700000	1116	48	51	12
Chr8	20700001	20800000	857	43	41	0
Chr8	20800001	20900000	952	38	44	0
Chr8	20900001	21000000	918	21	17	6
Chr8	21000001	21100000	966	40	1	37
Chr8	21100001	21200000	1115	23	2	22
Chr8	21200001	21300000	1075	19	2	21
Chr8	21300001	21400000	1326	32	28	6
Chr8	21400001	21500000	724	14	17	1
Chr8	21500001	21600000	873	56	52	2
Chr8	21600001	21700000	832	34	28	0
Chr8	21700001	21800000	1168	33	35	2
Chr8	21800001	21900000	1046	38	29	1
Chr8	21900001	22000000	636	12	11	0
Chr8	22000001	22100000	638	29	31	1
Chr8	22100001	22200000	1116	57	54	0

					InDel	
			All	Bengal/Nona Bokra	PSSR/Bengal	PSSR/Nona Bokra
Chr8	22200001	22300000	921	36	34	0
Chr8	22300001	22400000	731	22	22	0
Chr8	22400001	22500000	775	44	43	0
Chr8	22500001	22600000	499	23	21	0
Chr8	22600001	22700000	586	42	36	1
Chr8	22700001	22800000	927	44	36	0
Chr8	22800001	22900000	762	26	28	0
Chr8	22900001	23000000	724	38	31	0
Chr8	23000001	23100000	863	44	33	0
Chr8	23100001	23200000	681	32	26	0
Chr8	23200001	23300000	848	46	40	1
Chr8	23300001	23400000	656	39	40	1
Chr8	23400001	23500000	807	41	35	0
Chr8	23500001	23600000	669	38	31	1
Chr8	23600001	23700000	458	26	22	0
Chr8	23700001	23800000	28	1	1	0
Chr8	23800001	23900000	27	7	7	1
Chr8	23900001	24000000	22	2	2	1
Chr8	24000001	24100000	47	6	5	1
Chr8	24100001	24200000	12	1	1	0
Chr8	24200001	24300000	19	3	3	2
Chr8	24300001	24400000	422	0	14	12
Chr8	24400001	24500000	681	2	19	18
Chr8	24500001	24600000	923	4	35	24
Chr8	24600001	24700000	537	5	24	18
Chr8	24700001	24800000	908	11	25	30
Chr8	24800001	24900000	941	8	41	38
Chr8	24900001	25000000	89	3	4	5
Chr8	25000001	25100000	44	4	1	2
Chr8	25100001	25200000	43	1	1	0
Chr8	25200001	25300000	54	4	5	0
Chr8	25300001	25400000	461	27	10	20
Chr8	25400001	25500000	845	39	42	1
Chr8	25500001	25600000	694	21	13	9
Chr8	25600001	25700000	578	34	1	34
Chr8	25700001	25800000	586	33	1	31
Chr8	25800001	25900000	408	29	10	18
Chr8	25900001	26000000	496	22	20	0
Chr8	26000001	26100000	853	38	38	21
Chr8	26100001	26200000	1044	34	38	12
Chr8	26200001	26300000	1141	19	21	20
Chr8	26300001	26400000	1020	25	21	19
Chr8	26400001	26500000	1092	22	24	22
Chr8	26500001	26600000	1014	51	51	26
Chr8	26600001	26700000	222	6	3	5

					InDel	
			All	Bengal/Nona Bokra	PSSR/Bengal	PSSR/Nona Bokra
Chr8	26700001	26800000	780	22	19	22
Chr8	26800001	26900000	1201	22	36	26
Chr8	26900001	27000000	1024	34	24	11
Chr8	27000001	27100000	1188	41	28	27
Chr8	27100001	27200000	754	39	26	22
Chr8	27200001	27300000	844	45	44	12
Chr8	27300001	27400000	974	47	35	26
Chr8	27400001	27500000	1082	46	30	29
Chr8	27500001	27600000	861	35	35	11
Chr8	27600001	27700000	586	22	19	16
Chr8	27700001	27800000	963	29	31	9
Chr8	27800001	27900000	683	42	42	7
Chr8	27900001	28000000	616	28	26	3
Chr8	28000001	28100000	760	27	19	16
Chr8	28100001	28200000	979	57	33	20
Chr8	28200001	28300000	697	43	39	12
Chr8	28300001	28400000	608	33	27	1
Chr8	28400001	28500000	266	4	2	0
Chr9	Chr9					
Chr9	1	100000	174	3	1	0
Chr9	100001	200000	413	14	10	2
Chr9	200001	300000	884	34	31	5
Chr9	300001	400000	459	15	12	1
Chr9	400001	500000	603	24	20	8
Chr9	500001	600000	563	8	9	5
Chr9	600001	700000	1277	30	22	22
Chr9	700001	800000	999	20	16	9
Chr9	800001	900000	925	24	21	4
Chr9	900001	1000000	1006	36	35	17
Chr9	1000001	1100000	976	35	24	6
Chr9	1100001	1200000	753	32	24	5
Chr9	1200001	1300000	679	31	28	3
Chr9	1300001	1400000	672	17	15	0
Chr9	1400001	1500000	685	13	14	1
Chr9	1500001	1600000	747	23	16	4
Chr9	1600001	1700000	458	18	13	1
Chr9	1700001	1800000	761	28	27	0
Chr9	1800001	1900000	787	32	32	4
Chr9	1900001	2000000	795	11	18	0
Chr9	2000001	2100000	957	23	26	1
Chr9	2100001	2200000	534	22	21	0
Chr9	2200001	2300000	461	9	7	3
Chr9	2300001	2400000	669	24	22	2
Chr9	2400001	2500000	650	22	15	1
Chr9	2500001	2600000	732	27	20	3

					InDel	
			All	Bengal/Nona Bokra	PSSR/Bengal	PSSR/Nona Bokra
Chr9	2600001	2700000	608	28	28	3
Chr9	2700001	2800000	325	7	8	0
Chr9	2800001	2900000	295	4	6	0
Chr9	2900001	3000000	862	17	19	4
Chr9	3000001	3100000	757	21	19	2
Chr9	3100001	3200000	910	31	32	1
Chr9	3200001	3300000	528	13	13	0
Chr9	3300001	3400000	519	20	19	0
Chr9	3400001	3500000	757	21	17	0
Chr9	3500001	3600000	726	14	12	2
Chr9	3600001	3700000	596	24	28	3
Chr9	3700001	3800000	919	22	22	0
Chr9	3800001	3900000	828	26	24	7
Chr9	3900001	4000000	793	21	13	16
Chr9	4000001	4100000	1077	22	15	17
Chr9	4100001	4200000	1254	32	24	22
Chr9	4200001	4300000	2032	44	11	44
Chr9	4300001	4400000	1420	73	30	52
Chr9	4400001	4500000	812	33	30	1
Chr9	4500001	4600000	342	29	23	1
Chr9	4600001	4700000	526	22	12	0
Chr9	4700001	4800000	908	37	30	1
Chr9	4800001	4900000	91	1	0	0
Chr9	4900001	5000000	411	20	14	0
Chr9	5000001	5100000	624	18	19	0
Chr9	5100001	5200000	999	50	47	0
Chr9	5200001	5300000	549	16	10	0
Chr9	5300001	5400000	888	49	38	2
Chr9	5400001	5500000	855	37	31	0
Chr9	5500001	5600000	339	11	13	0
Chr9	5600001	5700000	1369	40	30	0
Chr9	5700001	5800000	775	35	33	0
Chr9	5800001	5900000	809	32	23	3
Chr9	5900001	6000000	910	32	24	28
Chr9	6000001	6100000	799	38	24	24
Chr9	6100001	6200000	914	11	23	6
Chr9	6200001	6300000	1332	33	20	35
Chr9	6300001	6400000	910	30	22	25
Chr9	6400001	6500000	653	17	10	3
Chr9	6500001	6600000	1180	9	20	10
Chr9	6600001	6700000	969	19	15	14
Chr9	6700001	6800000	1481	25	19	20
Chr9	6800001	6900000	940	19	17	0
Chr9	6900001	7000000	467	25	20	1
Chr9	7000001	7100000	972	18	14	3

					InDel	
			All	Bengal/Nona Bokra	PSSR/Bengal	PSSR/Nona Bokra
Chr9	7100001	7200000	894	29	29	16
Chr9	7200001	7300000	1060	50	25	33
Chr9	7300001	7400000	946	25	29	22
Chr9	7400001	7500000	959	16	19	15
Chr9	7500001	7600000	537	5	7	9
Chr9	7600001	7700000	415	11	7	6
Chr9	7700001	7800000	1018	33	21	2
Chr9	7800001	7900000	504	12	15	2
Chr9	7900001	8000000	732	46	32	21
Chr9	8000001	8100000	697	23	21	14
Chr9	8100001	8200000	751	38	34	3
Chr9	8200001	8300000	150	9	8	1
Chr9	8300001	8400000	113	1	1	0
Chr9	8400001	8500000	82	1	0	0
Chr9	8500001	8600000	504	22	22	0
Chr9	8600001	8700000	449	14	17	1
Chr9	8700001	8800000	589	31	17	0
Chr9	8800001	8900000	389	23	11	0
Chr9	8900001	9000000	525	12	5	3
Chr9	9000001	9100000	785	22	23	0
Chr9	9100001	9200000	1125	26	17	4
Chr9	9200001	9300000	1057	38	28	9
Chr9	9300001	9400000	1209	36	23	16
Chr9	9400001	9500000	481	3	13	12
Chr9	9500001	9600000	501	2	17	16
Chr9	9600001	9700000	574	6	16	13
Chr9	9700001	9800000	725	25	5	6
Chr9	9800001	9900000	737	33	26	0
Chr9	9900001	10000000	761	37	30	0
Chr9	10000001	10100000	500	17	13	3
Chr9	10100001	10200000	1016	30	28	12
Chr9	10200001	10300000	1191	28	19	11
Chr9	10300001	10400000	1388	32	17	23
Chr9	10400001	10500000	1027	19	12	1
Chr9	10500001	10600000	587	7	6	0
Chr9	10600001	10700000	105	0	0	0
Chr9	10700001	10800000	995	29	24	26
Chr9	10800001	10900000	993	42	39	10
Chr9	10900001	11000000	484	23	1	23
Chr9	11000001	11100000	644	25	6	27
Chr9	11100001	11200000	844	19	7	19
Chr9	11200001	11300000	1067	21	23	12
Chr9	11300001	11400000	767	23	11	10
Chr9	11400001	11500000	852	32	17	17
Chr9	11500001	11600000	484	16	6	14

					InDel	
			All	Bengal/Nona Bokra	PSSR/Bengal	PSSR/Nona Bokra
Chr9	11600001	11700000	721	24	2	24
Chr9	11700001	11800000	942	65	2	65
Chr9	11800001	11900000	733	46	2	47
Chr9	11900001	12000000	602	46	1	45
Chr9	12000001	12100000	965	59	5	59
Chr9	12100001	12200000	644	30	0	30
Chr9	12200001	12300000	699	47	5	47
Chr9	12300001	12400000	997	50	26	39
Chr9	12400001	12500000	1390	33	41	27
Chr9	12500001	12600000	1368	42	28	26
Chr9	12600001	12700000	722	20	8	17
Chr9	12700001	12800000	666	41	0	40
Chr9	12800001	12900000	711	34	13	15
Chr9	12900001	13000000	767	49	36	1
Chr9	13000001	13100000	627	21	18	0
Chr9	13100001	13200000	813	34	34	0
Chr9	13200001	13300000	448	11	12	0
Chr9	13300001	13400000	425	12	12	1
Chr9	13400001	13500000	456	6	4	0
Chr9	13500001	13600000	336	11	9	0
Chr9	13600001	13700000	554	22	17	5
Chr9	13700001	13800000	488	16	5	15
Chr9	13800001	13900000	33	2	1	1
Chr9	13900001	14000000	93	1	0	1
Chr9	14000001	14100000	582	20	21	0
Chr9	14100001	14200000	853	39	30	0
Chr9	14200001	14300000	219	6	6	0
Chr9	14300001	14400000	679	35	33	0
Chr9	14400001	14500000	1611	58	60	0
Chr9	14500001	14600000	690	24	20	2
Chr9	14600001	14700000	1050	53	36	30
Chr9	14700001	14800000	841	39	27	18
Chr9	14800001	14900000	954	50	45	2
Chr9	14900001	15000000	964	49	43	6
Chr9	15000001	15100000	953	69	46	0
Chr9	15100001	15200000	578	25	17	3
Chr9	15200001	15300000	1011	61	49	21
Chr9	15300001	15400000	808	31	28	16
Chr9	15400001	15500000	1008	32	34	28
Chr9	15500001	15600000	1252	50	25	20
Chr9	15600001	15700000	1277	46	32	37
Chr9	15700001	15800000	891	64	44	23
Chr9	15800001	15900000	1085	53	52	21
Chr9	15900001	16000000	1104	38	29	17
Chr9	16000001	16100000	719	53	37	1

					InDel	
			All	Bengal/Nona Bokra	PSSR/Bengal	PSSR/Nona Bokra
Chr9	16100001	16200000	939	58	49	11
Chr9	16200001	16300000	963	35	22	14
Chr9	16300001	16400000	797	27	28	14
Chr9	16400001	16500000	846	35	26	16
Chr9	16500001	16600000	762	33	29	12
Chr9	16600001	16700000	879	38	29	14
Chr9	16700001	16800000	808	26	27	9
Chr9	16800001	16900000	791	33	31	15
Chr9	16900001	17000000	786	30	28	10
Chr9	17000001	17100000	497	21	19	11
Chr9	17100001	17200000	699	28	23	13
Chr9	17200001	17300000	969	35	33	11
Chr9	17300001	17400000	947	53	40	18
Chr9	17400001	17500000	815	24	26	11
Chr9	17500001	17600000	1029	38	40	19
Chr9	17600001	17700000	817	31	29	17
Chr9	17700001	17800000	973	40	37	14
Chr9	17800001	17900000	838	36	40	12
Chr9	17900001	18000000	892	49	35	23
Chr9	18000001	18100000	727	27	18	8
Chr9	18100001	18200000	729	41	36	9
Chr9	18200001	18300000	200	20	2	20
Chr9	18300001	18400000	69	11	6	1
Chr9	18400001	18500000	268	3	2	2
Chr9	18500001	18600000	577	5	21	16
Chr9	18600001	18700000	783	27	24	20
Chr9	18700001	18800000	943	31	29	13
Chr9	18800001	18900000	897	39	34	18
Chr9	18900001	19000000	983	41	30	13
Chr9	19000001	19100000	785	28	28	19
Chr9	19100001	19200000	1091	33	19	35
Chr9	19200001	19300000	1057	52	28	52
Chr9	19300001	19400000	822	35	41	33
Chr9	19400001	19500000	888	34	38	7
Chr9	19500001	19600000	614	41	33	2
Chr9	19600001	19700000	796	50	45	13
Chr9	19700001	19800000	748	29	26	12
Chr9	19800001	19900000	860	48	40	6
Chr9	19900001	20000000	637	29	33	0
Chr9	20000001	20100000	506	43	39	1
Chr9	20100001	20200000	852	68	58	2
Chr9	20200001	20300000	931	58	49	15
Chr9	20300001	20400000	894	56	42	20
Chr9	20400001	20500000	925	38	30	8
Chr9	20500001	20600000	765	37	41	9

					InDel	
			All	Bengal/Nona Bokra	PSSR/Bengal	PSSR/Nona Bokra
Chr9	20600001	20700000	819	5	22	16
Chr9	20700001	20800000	764	37	20	28
Chr9	20800001	20900000	771	10	27	14
Chr9	20900001	21000000	717	30	23	20
Chr9	21000001	21100000	535	20	2	18
Chr9	21100001	21200000	651	29	11	15
Chr9	21200001	21300000	883	7	29	20
Chr9	21300001	21400000	578	18	28	11
Chr9	21400001	21500000	94	0	0	0
Chr9	21500001	21600000	97	5	5	0
Chr9	21600001	21700000	400	22	13	0
Chr9	21700001	21800000	696	42	37	1
Chr9	21800001	21900000	949	51	43	2
Chr9	21900001	22000000	694	47	35	0
Chr9	22000001	22100000	751	63	44	4
Chr9	22100001	22200000	475	31	25	6
Chr9	22200001	22300000	1398	84	72	21
Chr9	22300001	22400000	1226	53	28	28
Chr9	22400001	22500000	570	31	21	13
Chr9	22500001	22600000	633	24	16	19
Chr9	22600001	22700000	377	22	16	7
Chr9	22700001	22800000	575	42	37	0
Chr9	22800001	22900000	866	30	29	1
Chr9	22900001	23000000	261	13	13	0
Chr9	23000001	23100000	0	0	0	0
Chr10	Chr10					
Chr10	1	100000	715	26	30	2
Chr10	100001	200000	1134	41	41	6
Chr10	200001	300000	867	18	12	14
Chr10	300001	400000	772	30	22	10
Chr10	400001	500000	1503	57	30	38
Chr10	500001	600000	1093	21	7	19
Chr10	600001	700000	1105	24	24	11
Chr10	700001	800000	1116	25	17	18
Chr10	800001	900000	1399	59	30	13
Chr10	900001	1000000	1010	30	43	14
Chr10	1000001	1100000	522	29	30	13
Chr10	1100001	1200000	846	23	27	14
Chr10	1200001	1300000	720	22	15	5
Chr10	1300001	1400000	1016	21	24	11
Chr10	1400001	1500000	1435	35	36	32
Chr10	1500001	1600000	1177	26	31	3
Chr10	1600001	1700000	768	26	15	16
Chr10	1700001	1800000	900	32	21	18
Chr10	1800001	1900000	903	30	22	12

					InDel	
			All	Bengal/Nona Bokra	PSSR/Bengal	PSSR/Nona Bokra
Chr10	1900001	2000000	595	6	11	10
Chr10	2000001	2100000	1387	48	39	8
Chr10	2100001	2200000	1459	40	33	37
Chr10	2200001	2300000	1823	61	28	17
Chr10	2300001	2400000	1449	14	24	29
Chr10	2400001	2500000	1543	46	29	38
Chr10	2500001	2600000	1270	22	23	19
Chr10	2600001	2700000	1015	26	12	21
Chr10	2700001	2800000	1259	23	17	18
Chr10	2800001	2900000	1567	52	55	5
Chr10	2900001	3000000	2133	77	42	0
Chr10	3000001	3100000	1750	53	37	28
Chr10	3100001	3200000	1137	23	16	22
Chr10	3200001	3300000	321	0	0	0
Chr10	3300001	3400000	578	17	3	10
Chr10	3400001	3500000	1282	52	29	48
Chr10	3500001	3600000	1031	21	29	16
Chr10	3600001	3700000	1660	46	19	18
Chr10	3700001	3800000	1615	32	22	20
Chr10	3800001	3900000	788	33	9	22
Chr10	3900001	4000000	849	35	17	24
Chr10	4000001	4100000	1083	40	19	24
Chr10	4100001	4200000	332	1	4	1
Chr10	4200001	4300000	1856	28	23	19
Chr10	4300001	4400000	1331	27	7	21
Chr10	4400001	4500000	1228	9	4	10
Chr10	4500001	4600000	1614	17	11	11
Chr10	4600001	4700000	1194	20	25	1
Chr10	4700001	4800000	1461	26	21	20
Chr10	4800001	4900000	984	18	14	6
Chr10	4900001	5000000	1315	6	4	0
Chr10	5000001	5100000	377	2	1	0
Chr10	5100001	5200000	713	1	2	1
Chr10	5200001	5300000	1494	30	30	3
Chr10	5300001	5400000	882	13	25	13
Chr10	5400001	5500000	1549	37	53	38
Chr10	5500001	5600000	821	20	23	17
Chr10	5600001	5700000	1010	34	27	0
Chr10	5700001	5800000	390	25	22	1
Chr10	5800001	5900000	726	12	9	0
Chr10	5900001	6000000	604	8	10	1
Chr10	6000001	6100000	1066	10	11	0
Chr10	6100001	6200000	628	20	19	1
Chr10	6200001	6300000	528	15	14	0
Chr10	6300001	6400000	609	14	15	1

					InDel	
			All	Bengal/Nona Bokra	PSSR/Bengal	PSSR/Nona Bokra
Chr10	6400001	6500000	474	4	6	0
Chr10	6500001	6600000	857	14	14	0
Chr10	6600001	6700000	1148	16	18	0
Chr10	6700001	6800000	853	12	11	0
Chr10	6800001	6900000	579	18	14	2
Chr10	6900001	7000000	661	10	10	1
Chr10	7000001	7100000	307	7	5	0
Chr10	7100001	7200000	316	8	6	0
Chr10	7200001	7300000	690	9	12	0
Chr10	7300001	7400000	818	17	19	0
Chr10	7400001	7500000	1310	26	24	1
Chr10	7500001	7600000	1115	35	35	0
Chr10	7600001	7700000	978	21	24	0
Chr10	7700001	7800000	1024	14	17	1
Chr10	7800001	7900000	809	8	10	0
Chr10	7900001	8000000	932	12	12	1
Chr10	8000001	8100000	1015	8	8	0
Chr10	8100001	8200000	455	5	4	0
Chr10	8200001	8300000	630	10	9	0
Chr10	8300001	8400000	717	4	4	0
Chr10	8400001	8500000	815	18	18	0
Chr10	8500001	8600000	1033	18	17	1
Chr10	8600001	8700000	595	4	6	0
Chr10	8700001	8800000	618	7	11	0
Chr10	8800001	8900000	834	20	21	0
Chr10	8900001	9000000	1010	25	28	2
Chr10	9000001	9100000	1004	22	21	1
Chr10	9100001	9200000	1255	23	24	0
Chr10	9200001	9300000	701	24	25	0
Chr10	9300001	9400000	1471	31	36	0
Chr10	9400001	9500000	757	11	7	2
Chr10	9500001	9600000	1382	15	15	0
Chr10	9600001	9700000	1042	11	9	1
Chr10	9700001	9800000	1364	35	36	1
Chr10	9800001	9900000	828	19	18	0
Chr10	9900001	10000000	876	43	37	0
Chr10	10000001	10100000	649	19	17	1
Chr10	10100001	10200000	820	22	21	0
Chr10	10200001	10300000	831	24	27	0
Chr10	10300001	10400000	1147	47	46	1
Chr10	10400001	10500000	1333	32	31	0
Chr10	10500001	10600000	1053	34	21	0
Chr10	10600001	10700000	1075	21	22	7
Chr10	10700001	10800000	1262	29	24	0
Chr10	10800001	10900000	51	0	0	0

					InDel	
			All	Bengal/Nona Bokra	PSSR/Bengal	PSSR/Nona Bokra
Chr10	10900001	11000000	1097	21	20	0
Chr10	11000001	11100000	1222	24	18	11
Chr10	11100001	11200000	1566	8	20	15
Chr10	11200001	11300000	1137	34	35	5
Chr10	11300001	11400000	656	8	6	1
Chr10	11400001	11500000	311	0	1	0
Chr10	11500001	11600000	1235	18	13	1
Chr10	11600001	11700000	1265	38	44	0
Chr10	11700001	11800000	1118	33	39	2
Chr10	11800001	11900000	865	47	39	1
Chr10	11900001	12000000	1050	46	37	16
Chr10	12000001	12100000	925	53	43	7
Chr10	12100001	12200000	1149	34	26	32
Chr10	12200001	12300000	1342	27	27	16
Chr10	12300001	12400000	1296	26	14	22
Chr10	12400001	12500000	1108	14	12	5
Chr10	12500001	12600000	382	5	4	2
Chr10	12600001	12700000	206	7	0	1
Chr10	12700001	12800000	581	20	15	9
Chr10	12800001	12900000	898	27	20	11
Chr10	12900001	13000000	900	40	34	3
Chr10	13000001	13100000	235	15	11	1
Chr10	13100001	13200000	1472	45	43	1
Chr10	13200001	13300000	911	18	18	9
Chr10	13300001	13400000	1052	18	16	18
Chr10	13400001	13500000	1244	8	9	9
Chr10	13500001	13600000	1080	20	26	17
Chr10	13600001	13700000	1606	26	37	15
Chr10	13700001	13800000	1257	35	31	9
Chr10	13800001	13900000	829	44	36	19
Chr10	13900001	14000000	873	28	25	12
Chr10	14000001	14100000	972	47	31	6
Chr10	14100001	14200000	1034	55	35	13
Chr10	14200001	14300000	1611	74	40	43
Chr10	14300001	14400000	714	20	28	30
Chr10	14400001	14500000	749	43	14	32
Chr10	14500001	14600000	995	34	32	18
Chr10	14600001	14700000	1242	49	52	28
Chr10	14700001	14800000	1047	55	45	5
Chr10	14800001	14900000	754	40	35	5
Chr10	14900001	15000000	678	35	30	1
Chr10	15000001	15100000	648	18	19	2
Chr10	15100001	15200000	847	33	30	1
Chr10	15200001	15300000	511	26	19	0
Chr10	15300001	15400000	551	31	25	3

					InDel	
			All	Bengal/Nona Bokra	PSSR/Bengal	PSSR/Nona Bokra
Chr10	15400001	15500000	669	24	22	2
Chr10	15500001	15600000	974	40	36	0
Chr10	15600001	15700000	1003	59	51	2
Chr10	15700001	15800000	1003	23	25	1
Chr10	15800001	15900000	2114	88	93	4
Chr10	15900001	16000000	1678	81	66	7
Chr10	16000001	16100000	1775	67	48	30
Chr10	16100001	16200000	1054	41	35	12
Chr10	16200001	16300000	1193	20	24	19
Chr10	16300001	16400000	1247	39	11	19
Chr10	16400001	16500000	1254	43	27	18
Chr10	16500001	16600000	1097	33	35	11
Chr10	16600001	16700000	1500	80	45	14
Chr10	16700001	16800000	758	31	24	21
Chr10	16800001	16900000	768	22	24	1
Chr10	16900001	17000000	902	24	21	7
Chr10	17000001	17100000	949	25	26	0
Chr10	17100001	17200000	937	26	29	1
Chr10	17200001	17300000	1002	30	24	17
Chr10	17300001	17400000	1451	40	21	34
Chr10	17400001	17500000	713	41	31	12
Chr10	17500001	17600000	1128	56	48	4
Chr10	17600001	17700000	888	41	41	2
Chr10	17700001	17800000	692	40	38	2
Chr10	17800001	17900000	896	55	59	1
Chr10	17900001	18000000	843	45	41	1
Chr10	18000001	18100000	1031	55	49	9
Chr10	18100001	18200000	1128	53	36	18
Chr10	18200001	18300000	676	30	32	11
Chr10	18300001	18400000	772	1	33	27
Chr10	18400001	18500000	871	0	11	8
Chr10	18500001	18600000	732	0	0	0
Chr10	18600001	18700000	790	1	1	0
Chr10	18700001	18800000	706	3	2	1
Chr10	18800001	18900000	799	21	10	12
Chr10	18900001	19000000	723	10	8	9
Chr10	19000001	19100000	703	30	0	29
Chr10	19100001	19200000	645	0	0	0
Chr10	19200001	19300000	677	1	0	0
Chr10	19300001	19400000	1310	0	4	3
Chr10	19400001	19500000	814	6	2	5
Chr10	19500001	19600000	790	5	3	2
Chr10	19600001	19700000	982	1	11	10
Chr10	19700001	19800000	679	2	9	4
Chr10	19800001	19900000	1365	4	8	6

					InDel	
			All	Bengal/Nona Bokra	PSSR/Bengal	PSSR/Nona Bokra
Chr10	19900001	20000000	952	25	3	22
Chr10	20000001	20100000	673	21	10	8
Chr10	20100001	20200000	730	35	17	20
Chr10	20200001	20300000	1172	59	49	15
Chr10	20300001	20400000	995	80	48	1
Chr10	20400001	20500000	686	29	28	0
Chr10	20500001	20600000	522	23	19	0
Chr10	20600001	20700000	721	36	29	0
Chr10	20700001	20800000	664	48	47	1
Chr10	20800001	20900000	999	44	46	0
Chr10	20900001	21000000	642	34	27	0
Chr10	21000001	21100000	526	24	23	0
Chr10	21100001	21200000	856	42	41	1
Chr10	21200001	21300000	692	43	38	4
Chr10	21300001	21400000	539	45	34	0
Chr10	21400001	21500000	700	31	35	7
Chr10	21500001	21600000	640	23	24	0
Chr10	21600001	21700000	722	45	37	0
Chr10	21700001	21800000	690	38	27	1
Chr10	21800001	21900000	581	34	2	34
Chr10	21900001	22000000	555	22	2	22
Chr10	22000001	22100000	389	22	3	24
Chr10	22100001	22200000	650	1	32	28
Chr10	22200001	22300000	114	1	4	2
Chr10	22300001	22400000	18	2	1	2
Chr10	22400001	22500000	22	1	2	0
Chr10	22500001	22600000	777	17	27	11
Chr10	22600001	22700000	690	28	27	0
Chr10	22700001	22800000	850	32	33	12
Chr10	22800001	22900000	602	37	29	3
Chr10	22900001	23000000	612	33	25	0
Chr10	23000001	23100000	697	43	31	7
Chr10	23100001	23200000	697	28	19	10
Chr10	23200001	23300000	18	0	0	0
Chr11	Chr11					
Chr11	1	100000	261	3	2	2
Chr11	100001	200000	750	39	35	6
Chr11	200001	300000	620	24	21	1
Chr11	300001	400000	533	34	24	0
Chr11	400001	500000	475	20	18	0
Chr11	500001	600000	237	9	4	3
Chr11	600001	700000	312	14	1	15
Chr11	700001	800000	678	28	15	10
Chr11	800001	900000	636	20	19	1
Chr11	900001	1000000	558	26	23	0

					InDel	
			All	Bengal/Nona Bokra	PSSR/Bengal	PSSR/Nona Bokra
Chr11	1000001	1100000	809	14	27	4
Chr11	1100001	1200000	621	29	25	1
Chr11	1200001	1300000	552	23	23	1
Chr11	1300001	1400000	567	42	34	0
Chr11	1400001	1500000	541	23	20	0
Chr11	1500001	1600000	529	22	20	0
Chr11	1600001	1700000	473	33	26	0
Chr11	1700001	1800000	392	30	24	1
Chr11	1800001	1900000	555	36	30	1
Chr11	1900001	2000000	602	36	26	0
Chr11	2000001	2100000	491	20	17	0
Chr11	2100001	2200000	825	37	31	10
Chr11	2200001	2300000	738	32	18	14
Chr11	2300001	2400000	770	37	30	8
Chr11	2400001	2500000	955	53	51	0
Chr11	2500001	2600000	926	45	46	22
Chr11	2600001	2700000	944	37	24	21
Chr11	2700001	2800000	668	34	26	6
Chr11	2800001	2900000	553	38	3	40
Chr11	2900001	3000000	24	2	3	3
Chr11	3000001	3100000	164	7	1	6
Chr11	3100001	3200000	399	25	6	25
Chr11	3200001	3300000	697	8	7	3
Chr11	3300001	3400000	735	1	1	0
Chr11	3400001	3500000	401	1	0	1
Chr11	3500001	3600000	1222	45	0	39
Chr11	3600001	3700000	1280	36	3	31
Chr11	3700001	3800000	733	40	29	21
Chr11	3800001	3900000	664	40	35	4
Chr11	3900001	4000000	928	41	36	0
Chr11	4000001	4100000	642	43	40	1
Chr11	4100001	4200000	835	45	29	1
Chr11	4200001	4300000	1378	91	60	0
Chr11	4300001	4400000	1269	76	27	23
Chr11	4400001	4500000	1011	56	17	51
Chr11	4500001	4600000	766	51	1	52
Chr11	4600001	4700000	311	21	0	21
Chr11	4700001	4800000	686	59	0	56
Chr11	4800001	4900000	915	65	5	62
Chr11	4900001	5000000	1886	44	62	33
Chr11	5000001	5100000	1802	55	52	10
Chr11	5100001	5200000	1315	24	18	16
Chr11	5200001	5300000	367	9	3	2
Chr11	5300001	5400000	718	27	27	4
Chr11	5400001	5500000	877	40	42	12

					InDel	
			All	Bengal/Nona Bokra	PSSR/Bengal	PSSR/Nona Bokra
Chr11	5500001	5600000	904	19	21	16
Chr11	5600001	5700000	988	45	34	26
Chr11	5700001	5800000	1227	42	28	28
Chr11	5800001	5900000	1202	53	37	4
Chr11	5900001	6000000	509	38	12	3
Chr11	6000001	6100000	982	41	27	3
Chr11	6100001	6200000	843	27	27	17
Chr11	6200001	6300000	919	21	26	20
Chr11	6300001	6400000	996	31	31	19
Chr11	6400001	6500000	590	25	21	10
Chr11	6500001	6600000	717	19	16	5
Chr11	6600001	6700000	1212	52	11	11
Chr11	6700001	6800000	322	0	0	0
Chr11	6800001	6900000	656	8	8	10
Chr11	6900001	7000000	1531	40	50	27
Chr11	7000001	7100000	1540	30	30	18
Chr11	7100001	7200000	1119	25	21	14
Chr11	7200001	7300000	875	21	24	13
Chr11	7300001	7400000	971	19	14	10
Chr11	7400001	7500000	841	34	35	4
Chr11	7500001	7600000	727	40	39	0
Chr11	7600001	7700000	629	44	48	1
Chr11	7700001	7800000	936	38	37	8
Chr11	7800001	7900000	1083	69	45	23
Chr11	7900001	8000000	601	38	29	8
Chr11	8000001	8100000	854	33	27	15
Chr11	8100001	8200000	534	22	16	1
Chr11	8200001	8300000	800	16	15	1
Chr11	8300001	8400000	894	12	12	6
Chr11	8400001	8500000	873	29	23	14
Chr11	8500001	8600000	1042	30	31	1
Chr11	8600001	8700000	921	32	27	3
Chr11	8700001	8800000	1479	46	37	0
Chr11	8800001	8900000	877	38	19	2
Chr11	8900001	9000000	564	20	14	5
Chr11	9000001	9100000	1229	46	31	36
Chr11	9100001	9200000	929	41	43	5
Chr11	9200001	9300000	1928	27	29	3
Chr11	9300001	9400000	717	5	8	0
Chr11	9400001	9500000	1172	26	28	1
Chr11	9500001	9600000	765	6	7	2
Chr11	9600001	9700000	276	0	1	2
Chr11	9700001	9800000	742	18	22	2
Chr11	9800001	9900000	1145	25	27	1
Chr11	9900001	10000000	700	2	2	0

					InDel	
			All	Bengal/Nona Bokra	PSSR/Bengal	PSSR/Nona Bokra
Chr11	10000001	10100000	1033	1	7	7
Chr11	10100001	10200000	1795	14	9	12
Chr11	10200001	10300000	1612	10	20	17
Chr11	10300001	10400000	1706	15	15	12
Chr11	10400001	10500000	1474	18	12	11
Chr11	10500001	10600000	1508	25	25	12
Chr11	10600001	10700000	1281	21	16	19
Chr11	10700001	10800000	940	19	22	18
Chr11	10800001	10900000	1258	21	15	20
Chr11	10900001	11000000	1429	14	18	17
Chr11	11000001	11100000	1233	23	23	10
Chr11	11100001	11200000	1221	17	20	1
Chr11	11200001	11300000	670	13	11	0
Chr11	11300001	11400000	882	16	17	0
Chr11	11400001	11500000	1106	5	4	0
Chr11	11500001	11600000	1153	15	15	2
Chr11	11600001	11700000	984	9	8	2
Chr11	11700001	11800000	841	7	11	3
Chr11	11800001	11900000	1021	10	13	0
Chr11	11900001	12000000	790	4	4	2
Chr11	12000001	12100000	440	6	6	1
Chr11	12100001	12200000	185	2	1	1
Chr11	12200001	12300000	188	0	0	0
Chr11	12300001	12400000	76	0	0	0
Chr11	12400001	12500000	0	0	0	0
Chr11	12500001	12600000	591	8	8	0
Chr11	12600001	12700000	1062	7	7	0
Chr11	12700001	12800000	1365	5	6	0
Chr11	12800001	12900000	1013	9	12	0
Chr11	12900001	13000000	314	0	1	0
Chr11	13000001	13100000	1498	10	10	1
Chr11	13100001	13200000	1704	9	8	0
Chr11	13200001	13300000	1386	8	9	1
Chr11	13300001	13400000	1451	9	10	0
Chr11	13400001	13500000	1206	2	4	1
Chr11	13500001	13600000	1132	12	15	2
Chr11	13600001	13700000	915	13	16	0
Chr11	13700001	13800000	1185	17	22	0
Chr11	13800001	13900000	1184	12	19	5
Chr11	13900001	14000000	1092	0	30	24
Chr11	14000001	14100000	1673	0	62	45
Chr11	14100001	14200000	938	0	31	25
Chr11	14200001	14300000	882	0	8	4
Chr11	14300001	14400000	989	6	19	17
Chr11	14400001	14500000	601	0	0	0

					InDel	
			All	Bengal/Nona Bokra	PSSR/Bengal	PSSR/Nona Bokra
Chr11	14500001	14600000	1206	0	19	14
Chr11	14600001	14700000	1461	3	18	16
Chr11	14700001	14800000	1738	1	22	18
Chr11	14800001	14900000	1082	0	28	23
Chr11	14900001	15000000	972	0	9	7
Chr11	15000001	15100000	886	0	2	2
Chr11	15100001	15200000	769	0	9	7
Chr11	15200001	15300000	903	0	15	13
Chr11	15300001	15400000	1772	11	29	22
Chr11	15400001	15500000	1528	37	39	24
Chr11	15500001	15600000	1441	8	19	17
Chr11	15600001	15700000	1496	4	22	18
Chr11	15700001	15800000	1267	14	25	22
Chr11	15800001	15900000	1642	22	49	33
Chr11	15900001	16000000	887	21	17	3
Chr11	16000001	16100000	1540	16	5	15
Chr11	16100001	16200000	1746	25	14	25
Chr11	16200001	16300000	1532	21	40	30
Chr11	16300001	16400000	1356	24	14	12
Chr11	16400001	16500000	2194	59	22	32
Chr11	16500001	16600000	1190	26	33	5
Chr11	16600001	16700000	1292	32	29	0
Chr11	16700001	16800000	1194	46	41	1
Chr11	16800001	16900000	992	10	14	0
Chr11	16900001	17000000	1277	77	67	15
Chr11	17000001	17100000	1246	58	41	19
Chr11	17100001	17200000	1533	20	20	10
Chr11	17200001	17300000	1722	67	21	31
Chr11	17300001	17400000	1770	48	40	34
Chr11	17400001	17500000	1212	29	26	16
Chr11	17500001	17600000	926	13	12	9
Chr11	17600001	17700000	1198	44	42	31
Chr11	17700001	17800000	1149	41	33	26
Chr11	17800001	17900000	2402	31	32	21
Chr11	17900001	18000000	1302	40	39	18
Chr11	18000001	18100000	1020	44	32	15
Chr11	18100001	18200000	1225	21	23	23
Chr11	18200001	18300000	1525	11	21	20
Chr11	18300001	18400000	1320	30	31	31
Chr11	18400001	18500000	1038	51	42	33
Chr11	18500001	18600000	1071	16	38	36
Chr11	18600001	18700000	604	9	30	29
Chr11	18700001	18800000	820	26	19	16
Chr11	18800001	18900000	1240	31	32	25
Chr11	18900001	19000000	1025	34	37	16

					InDel	
			All	Bengal/Nona Bokra	PSSR/Bengal	PSSR/Nona Bokra
Chr11	19000001	19100000	948	29	17	17
Chr11	19100001	19200000	399	29	1	30
Chr11	19200001	19300000	503	18	3	19
Chr11	19300001	19400000	654	34	35	2
Chr11	19400001	19500000	830	48	44	2
Chr11	19500001	19600000	676	39	36	0
Chr11	19600001	19700000	1485	53	45	22
Chr11	19700001	19800000	1487	30	30	14
Chr11	19800001	19900000	1585	35	16	22
Chr11	19900001	20000000	1091	50	39	12
Chr11	20000001	20100000	952	6	16	17
Chr11	20100001	20200000	820	44	25	19
Chr11	20200001	20300000	279	0	1	0
Chr11	20300001	20400000	796	27	25	16
Chr11	20400001	20500000	488	15	12	10
Chr11	20500001	20600000	991	27	28	34
Chr11	20600001	20700000	686	35	31	5
Chr11	20700001	20800000	655	35	32	1
Chr11	20800001	20900000	830	20	19	7
Chr11	20900001	21000000	1139	22	13	18
Chr11	21000001	21100000	935	12	9	9
Chr11	21100001	21200000	561	18	7	16
Chr11	21200001	21300000	849	43	12	38
Chr11	21300001	21400000	669	23	3	20
Chr11	21400001	21500000	1291	77	68	14
Chr11	21500001	21600000	568	15	16	2
Chr11	21600001	21700000	558	11	6	3
Chr11	21700001	21800000	1142	39	39	5
Chr11	21800001	21900000	1493	34	31	0
Chr11	21900001	22000000	1590	41	42	8
Chr11	22000001	22100000	1177	35	34	27
Chr11	22100001	22200000	1065	18	25	10
Chr11	22200001	22300000	1244	49	28	17
Chr11	22300001	22400000	644	5	9	3
Chr11	22400001	22500000	1319	48	44	22
Chr11	22500001	22600000	1335	42	40	41
Chr11	22600001	22700000	1425	31	27	8
Chr11	22700001	22800000	1166	39	18	17
Chr11	22800001	22900000	1049	20	16	12
Chr11	22900001	23000000	1539	51	39	39
Chr11	23000001	23100000	1467	39	21	38
Chr11	23100001	23200000	1315	31	29	22
Chr11	23200001	23300000	1331	51	39	24
Chr11	23300001	23400000	531	15	6	3
Chr11	23400001	23500000	785	28	12	18

					InDel	
			All	Bengal/Nona Bokra	PSSR/Bengal	PSSR/Nona Bokra
Chr11	23500001	23600000	273	16	12	8
Chr11	23600001	23700000	563	26	12	25
Chr11	23700001	23800000	658	24	13	19
Chr11	23800001	23900000	745	28	22	14
Chr11	23900001	24000000	1243	69	20	32
Chr11	24000001	24100000	1202	39	19	14
Chr11	24100001	24200000	1057	43	32	10
Chr11	24200001	24300000	1079	47	43	10
Chr11	24300001	24400000	918	42	22	12
Chr11	24400001	24500000	1139	39	20	19
Chr11	24500001	24600000	316	20	8	0
Chr11	24600001	24700000	780	54	42	0
Chr11	24700001	24800000	953	39	31	0
Chr11	24800001	24900000	837	34	27	0
Chr11	24900001	25000000	740	26	20	20
Chr11	25000001	25100000	757	32	25	18
Chr11	25100001	25200000	1041	45	39	38
Chr11	25200001	25300000	781	8	40	32
Chr11	25300001	25400000	544	1	16	14
Chr11	25400001	25500000	578	2	15	13
Chr11	25500001	25600000	1020	42	33	36
Chr11	25600001	25700000	789	14	17	13
Chr11	25700001	25800000	762	27	21	16
Chr11	25800001	25900000	947	29	24	33
Chr11	25900001	26000000	383	14	14	4
Chr11	26000001	26100000	761	21	6	21
Chr11	26100001	26200000	495	9	11	9
Chr11	26200001	26300000	796	34	14	17
Chr11	26300001	26400000	436	22	8	17
Chr11	26400001	26500000	607	27	25	21
Chr11	26500001	26600000	837	43	18	21
Chr11	26600001	26700000	235	5	10	0
Chr11	26700001	26800000	279	7	3	2
Chr11	26800001	26900000	566	8	11	3
Chr11	26900001	27000000	708	28	14	15
Chr11	27000001	27100000	534	15	9	11
Chr11	27100001	27200000	757	27	28	20
Chr11	27200001	27300000	865	11	13	16
Chr11	27300001	27400000	843	11	18	5
Chr11	27400001	27500000	1110	40	29	26
Chr11	27500001	27600000	719	23	14	3
Chr11	27600001	27700000	1044	44	28	18
Chr11	27700001	27800000	1414	35	30	20
Chr11	27800001	27900000	1161	18	15	23
Chr11	27900001	28000000	628	7	7	5

					InDel	
			All	Bengal/Nona Bokra	PSSR/Bengal	PSSR/Nona Bokra
Chr11	28000001	28100000	414	7	7	2
Chr11	28100001	28200000	617	10	3	4
Chr11	28200001	28300000	398	1	5	3
Chr11	28300001	28400000	739	1	2	2
Chr11	28400001	28500000	1155	17	20	19
Chr11	28500001	28600000	177	0	0	1
Chr11	28600001	28700000	978	18	7	16
Chr11	28700001	28800000	1306	45	24	45
Chr11	28800001	28900000	1032	42	43	0
Chr11	28900001	29000000	562	31	24	0
Chr11	29000001	29100000	109	5	5	0
Chr12	Chr12					
Chr12	1	100000	230	0	5	2
Chr12	100001	200000	370	2	10	10
Chr12	200001	300000	643	0	19	17
Chr12	300001	400000	440	1	10	9
Chr12	400001	500000	329	1	16	16
Chr12	500001	600000	345	10	11	1
Chr12	600001	700000	996	39	34	5
Chr12	700001	800000	866	34	23	26
Chr12	800001	900000	746	21	19	21
Chr12	900001	1000000	422	29	8	16
Chr12	1000001	1100000	529	29	5	28
Chr12	1100001	1200000	495	28	2	27
Chr12	1200001	1300000	495	44	2	45
Chr12	1300001	1400000	593	30	15	14
Chr12	1400001	1500000	837	20	16	15
Chr12	1500001	1600000	717	33	28	10
Chr12	1600001	1700000	605	27	22	0
Chr12	1700001	1800000	521	30	28	0
Chr12	1800001	1900000	481	34	22	2
Chr12	1900001	2000000	445	21	21	0
Chr12	2000001	2100000	409	18	15	0
Chr12	2100001	2200000	552	30	31	1
Chr12	2200001	2300000	826	28	28	6
Chr12	2300001	2400000	696	41	34	0
Chr12	2400001	2500000	848	23	22	5
Chr12	2500001	2600000	893	20	0	20
Chr12	2600001	2700000	146	3	6	9
Chr12	2700001	2800000	714	1	36	28
Chr12	2800001	2900000	531	20	20	37
Chr12	2900001	3000000	821	5	38	29
Chr12	3000001	3100000	1284	26	38	34
Chr12	3100001	3200000	1104	32	32	33
Chr12	3200001	3300000	773	1	12	10

					InDel	
			All	Bengal/Nona Bokra	PSSR/Bengal	PSSR/Nona Bokra
Chr12	3300001	3400000	893	31	1	29
Chr12	3400001	3500000	1076	41	0	34
Chr12	3500001	3600000	940	11	3	9
Chr12	3600001	3700000	938	17	12	3
Chr12	3700001	3800000	1161	18	15	29
Chr12	3800001	3900000	886	1	17	18
Chr12	3900001	4000000	916	34	1	28
Chr12	4000001	4100000	591	35	0	23
Chr12	4100001	4200000	939	68	0	35
Chr12	4200001	4300000	887	15	0	15
Chr12	4300001	4400000	151	0	0	0
Chr12	4400001	4500000	1282	12	21	20
Chr12	4500001	4600000	1133	1	0	1
Chr12	4600001	4700000	969	27	22	0
Chr12	4700001	4800000	829	31	26	0
Chr12	4800001	4900000	731	23	28	1
Chr12	4900001	5000000	1110	31	22	0
Chr12	5000001	5100000	1056	62	49	0
Chr12	5100001	5200000	533	29	21	0
Chr12	5200001	5300000	49	0	0	0
Chr12	5300001	5400000	383	13	9	0
Chr12	5400001	5500000	698	35	23	0
Chr12	5500001	5600000	639	28	22	1
Chr12	5600001	5700000	523	37	34	0
Chr12	5700001	5800000	775	34	37	1
Chr12	5800001	5900000	1268	64	49	0
Chr12	5900001	6000000	200	4	2	0
Chr12	6000001	6100000	38	1	0	0
Chr12	6100001	6200000	965	33	33	0
Chr12	6200001	6300000	717	21	25	2
Chr12	6300001	6400000	299	3	6	0
Chr12	6400001	6500000	72	1	1	0
Chr12	6500001	6600000	723	19	17	0
Chr12	6600001	6700000	209	11	5	0
Chr12	6700001	6800000	897	47	37	0
Chr12	6800001	6900000	645	34	29	1
Chr12	6900001	7000000	1242	61	35	18
Chr12	7000001	7100000	550	15	10	1
Chr12	7100001	7200000	1030	74	46	1
Chr12	7200001	7300000	958	32	29	0
Chr12	7300001	7400000	784	25	22	0
Chr12	7400001	7500000	856	43	33	1
Chr12	7500001	7600000	774	30	28	0
Chr12	7600001	7700000	668	34	30	0
Chr12	7700001	7800000	866	42	37	0

					InDel	
			All	Bengal/Nona Bokra	PSSR/Bengal	PSSR/Nona Bokra
Chr12	7800001	7900000	1356	86	45	0
Chr12	7900001	8000000	971	39	38	0
Chr12	8000001	8100000	825	39	38	0
Chr12	8100001	8200000	374	8	7	0
Chr12	8200001	8300000	1018	32	29	0
Chr12	8300001	8400000	632	26	17	0
Chr12	8400001	8500000	871	19	17	0
Chr12	8500001	8600000	236	1	0	0
Chr12	8600001	8700000	289	13	8	1
Chr12	8700001	8800000	620	40	15	0
Chr12	8800001	8900000	1280	62	45	1
Chr12	8900001	9000000	843	27	25	0
Chr12	9000001	9100000	1118	28	28	0
Chr12	9100001	9200000	720	25	22	0
Chr12	9200001	9300000	858	41	38	1
Chr12	9300001	9400000	462	16	18	0
Chr12	9400001	9500000	518	20	19	0
Chr12	9500001	9600000	612	28	28	0
Chr12	9600001	9700000	320	4	3	0
Chr12	9700001	9800000	646	13	10	0
Chr12	9800001	9900000	531	17	21	0
Chr12	9900001	10000000	604	24	17	1
Chr12	10000001	10100000	774	25	25	0
Chr12	10100001	10200000	803	17	17	0
Chr12	10200001	10300000	674	31	26	1
Chr12	10300001	10400000	648	22	17	0
Chr12	10400001	10500000	562	32	32	0
Chr12	10500001	10600000	353	11	10	1
Chr12	10600001	10700000	536	17	17	0
Chr12	10700001	10800000	1064	65	55	2
Chr12	10800001	10900000	559	26	21	0
Chr12	10900001	11000000	384	24	23	0
Chr12	11000001	11100000	393	25	29	0
Chr12	11100001	11200000	360	20	21	0
Chr12	11200001	11300000	447	22	19	1
Chr12	11300001	11400000	269	14	12	0
Chr12	11400001	11500000	315	8	7	0
Chr12	11500001	11600000	228	5	7	0
Chr12	11600001	11700000	332	5	3	0
Chr12	11700001	11800000	327	7	6	0
Chr12	11800001	11900000	412	9	5	0
Chr12	11900001	12000000	341	11	10	0
Chr12	12000001	12100000	201	8	8	0
Chr12	12100001	12200000	377	12	15	0
Chr12	12200001	12300000	304	7	10	0

					InDel	
			All	Bengal/Nona Bokra	PSSR/Bengal	PSSR/Nona Bokra
Chr12	12300001	12400000	299	12	10	0
Chr12	12400001	12500000	533	25	19	0
Chr12	12500001	12600000	567	24	19	1
Chr12	12600001	12700000	726	12	13	1
Chr12	12700001	12800000	753	19	18	0
Chr12	12800001	12900000	678	17	14	1
Chr12	12900001	13000000	929	38	37	0
Chr12	13000001	13100000	619	14	13	0
Chr12	13100001	13200000	997	44	32	0
Chr12	13200001	13300000	660	14	13	0
Chr12	13300001	13400000	167	1	0	0
Chr12	13400001	13500000	948	32	30	0
Chr12	13500001	13600000	559	20	20	0
Chr12	13600001	13700000	918	47	39	0
Chr12	13700001	13800000	1113	63	57	0
Chr12	13800001	13900000	685	27	23	0
Chr12	13900001	14000000	580	16	15	0
Chr12	14000001	14100000	1142	39	34	0
Chr12	14100001	14200000	1091	28	20	0
Chr12	14200001	14300000	1240	28	23	0
Chr12	14300001	14400000	198	0	0	0
Chr12	14400001	14500000	1097	40	38	0
Chr12	14500001	14600000	925	29	28	0
Chr12	14600001	14700000	650	26	19	0
Chr12	14700001	14800000	1234	44	35	0
Chr12	14800001	14900000	748	43	39	0
Chr12	14900001	15000000	869	21	17	1
Chr12	15000001	15100000	840	25	15	0
Chr12	15100001	15200000	454	25	22	0
Chr12	15200001	15300000	832	40	29	0
Chr12	15300001	15400000	777	41	28	0
Chr12	15400001	15500000	892	33	33	0
Chr12	15500001	15600000	394	14	11	1
Chr12	15600001	15700000	185	0	4	0
Chr12	15700001	15800000	517	16	13	0
Chr12	15800001	15900000	780	22	22	1
Chr12	15900001	16000000	833	29	22	0
Chr12	16000001	16100000	381	18	8	0
Chr12	16100001	16200000	1113	54	30	0
Chr12	16200001	16300000	809	50	28	0
Chr12	16300001	16400000	647	24	16	0
Chr12	16400001	16500000	812	60	41	0
Chr12	16500001	16600000	733	14	20	0
Chr12	16600001	16700000	419	16	12	0
Chr12	16700001	16800000	373	12	4	1

					InDel	
			All	Bengal/Nona Bokra	PSSR/Bengal	PSSR/Nona Bokra
Chr12	16800001	16900000	75	4	2	0
Chr12	16900001	17000000	531	33	13	0
Chr12	17000001	17100000	716	19	16	0
Chr12	17100001	17200000	159	1	1	0
Chr12	17200001	17300000	419	13	13	0
Chr12	17300001	17400000	626	26	20	1
Chr12	17400001	17500000	789	26	14	10
Chr12	17500001	17600000	875	49	27	22
Chr12	17600001	17700000	734	28	25	11
Chr12	17700001	17800000	392	17	6	4
Chr12	17800001	17900000	597	14	4	2
Chr12	17900001	18000000	905	34	33	39
Chr12	18000001	18100000	982	23	13	16
Chr12	18100001	18200000	954	15	15	13
Chr12	18200001	18300000	530	18	21	10
Chr12	18300001	18400000	568	40	34	2
Chr12	18400001	18500000	512	15	11	2
Chr12	18500001	18600000	1145	57	26	1
Chr12	18600001	18700000	1007	48	25	1
Chr12	18700001	18800000	547	16	8	3
Chr12	18800001	18900000	873	41	24	18
Chr12	18900001	19000000	478	19	16	2
Chr12	19000001	19100000	390	17	10	5
Chr12	19100001	19200000	1261	76	38	33
Chr12	19200001	19300000	594	32	16	1
Chr12	19300001	19400000	1043	55	37	0
Chr12	19400001	19500000	757	30	33	5
Chr12	19500001	19600000	821	33	32	0
Chr12	19600001	19700000	791	59	55	1
Chr12	19700001	19800000	869	37	33	6
Chr12	19800001	19900000	991	28	26	17
Chr12	19900001	20000000	956	30	34	14
Chr12	20000001	20100000	629	21	31	16
Chr12	20100001	20200000	379	11	6	1
Chr12	20200001	20300000	76	1	1	0
Chr12	20300001	20400000	217	11	2	0
Chr12	20400001	20500000	101	4	3	1
Chr12	20500001	20600000	38	0	0	0
Chr12	20600001	20700000	65	0	0	0
Chr12	20700001	20800000	566	32	18	8
Chr12	20800001	20900000	1259	47	39	16
Chr12	20900001	21000000	663	28	27	3
Chr12	21000001	21100000	977	28	25	0
Chr12	21100001	21200000	236	8	7	0
Chr12	21200001	21300000	169	14	1	12

					InDel	
			All	Bengal/Nona Bokra	PSSR/Bengal	PSSR/Nona Bokra
Chr12	21300001	21400000	303	17	1	16
Chr12	21400001	21500000	334	26	0	27
Chr12	21500001	21600000	672	26	0	27
Chr12	21600001	21700000	538	25	0	24
Chr12	21700001	21800000	534	19	7	20
Chr12	21800001	21900000	826	26	20	2
Chr12	21900001	22000000	486	24	25	1
Chr12	22000001	22100000	446	14	18	4
Chr12	22100001	22200000	1135	45	42	8
Chr12	22200001	22300000	1014	39	33	14
Chr12	22300001	22400000	641	15	17	7
Chr12	22400001	22500000	1193	79	24	7
Chr12	22500001	22600000	736	32	31	32
Chr12	22600001	22700000	570	27	32	0
Chr12	22700001	22800000	846	43	37	0
Chr12	22800001	22900000	678	28	19	0
Chr12	22900001	23000000	404	17	12	0
Chr12	23000001	23100000	635	39	31	1
Chr12	23100001	23200000	631	36	27	4
Chr12	23200001	23300000	498	32	28	0
Chr12	23300001	23400000	781	49	35	0
Chr12	23400001	23500000	1102	52	47	16
Chr12	23500001	23600000	743	41	30	16
Chr12	23600001	23700000	774	20	28	4
Chr12	23700001	23800000	580	13	14	15
Chr12	23800001	23900000	734	26	30	15
Chr12	23900001	24000000	680	42	30	9
Chr12	24000001	24100000	741	50	55	0
Chr12	24100001	24200000	648	46	38	3
Chr12	24200001	24300000	809	59	43	0
Chr12	24300001	24400000	607	18	17	0
Chr12	24400001	24500000	744	33	29	0
Chr12	24500001	24600000	639	21	22	0
Chr12	24600001	24700000	935	13	14	1
Chr12	24700001	24800000	700	1	1	1
Chr12	24800001	24900000	311	0	0	0
Chr12	24900001	25000000	201	0	0	1
Chr12	25000001	25100000	366	2	2	1
Chr12	25100001	25200000	249	2	1	2
Chr12	25200001	25300000	126	2	1	0
Chr12	25300001	25400000	173	13	14	1
Chr12	25400001	25500000	39	1	3	0
Chr12	25500001	25600000	334	4	15	12
Chr12	25600001	25700000	524	1	22	19
Chr12	25700001	25800000	487	6	18	14

					InDel	
			All	Bengal/Nona Bokra	PSSR/Bengal	PSSR/Nona Bokra
Chr12	25800001	25900000	628	18	14	0
Chr12	25900001	26000000	763	37	36	0
Chr12	26000001	26100000	830	58	36	22
Chr12	26100001	26200000	1151	73	1	74
Chr12	26200001	26300000	668	38	3	39
Chr12	26300001	26400000	28	1	1	1
Chr12	26400001	26500000	220	2	11	8
Chr12	26500001	26600000	342	1	10	9
Chr12	26600001	26700000	665	1	27	25
Chr12	26700001	26800000	618	1	18	17
Chr12	26800001	26900000	536	2	18	16
Chr12	26900001	27000000	967	0	28	28
Chr12	27000001	27100000	2082	2	23	21
Chr12	27100001	27200000	868	0	71	60
Chr12	27200001	27300000	653	18	35	19
Chr12	27300001	27400000	653	49	36	8
Chr12	27400001	27500000	630	29	29	3
Chr12	27500001	27600000	172	11	11	0
	ChrUn					
	1	100000	520	4	7	1
	100001	200000	487	12	14	6
	200001	300000	75	0	0	2
	300001	400000	317	0	0	13
	400001	500000	176	0	0	7
	500001	600000	395	2	2	3
	ChrSy					
	1	100000	362	15	13	1
	100001	200000	739	23	26	2
	200001	300000	464	6	4	4
	300001	400000	607	4	7	0
	400001	500000	69	0	0	0
	500001	600000	581	9	5	0
	600001	700000	0	0	0	0

Table S5. Amplification and validation of randomly selected SNPs and InDels by PCR followed by Sanger sequencing

Variants	Genomic location	Nipponbare(Ref)	Bengal	Nona Bokra	PSRR-1	Gene harboring the variants	sequecning result
SNP1	Chr4: 33669880	C	C	C	T (T)	LOC_Os04g56480	correct
SNP2	Chr4:34411764	C	C	A	A (A)	LOC_Os04g57780	correct
SNP3	Chr7:5458710	T	T	C	C(C)	LOC_Os07g10150	correct
SNP4	Chr7:6044099	G	G	G	A(A)	LOC_Os07g11000	correct
SNP5	Chr7:23903485	G	G	A	A(A)	LOC_Os07g39860	correct
SNP6	Chr7:23622343	A	A	T	T(T)	LOC_Os07g39430	correct
SNP7	Chr7:5642461	G	G	A	A(A)	LOC_Os07g10480	correct
Insertion1	Chr7:5642498	A	A	ATTAGG	ATTAGG(ATTAGG)	LOC_Os07g10480	correct
Insertion2	Chr7:23797123	G	G	GGTCAT	GGTCAT(GGTCAT)	LOC_Os07g39700	correct
Insertion3	Chr7:23837833	G	G	GT	GT(GT)	LOC_Os07g39780	correct
Insertion4	Chr4:33399612	G	G	GC	GC (GC)	LOC_Os04g56080	correct
Insertion5	Chr7:5492109	C	C	CTACCGTAG	CTACCGTAG(CTACCGTAG)	LOC_Os07g10220	correct
Insertion6	Chr4:34331543	A	A	AGTTTTCTCAGC	A(AGTTTTCTCAGC)	LOC_Os04g57660	wrong
Insertion7	Chr4:34506529	T	T	TG	TG(TG)	LOC_Os04g57920	correct
Insertion8	Chr7:5272729	A	A	AGT	AGT(AGT)	LOC_Os07g09900	correct
Deletion1	Chr7:5507388	CGCCGAAG	CGCCGAAG	C	C(C)	LOC_Os07g10254	correct
Deletion2	Chr7:5768328	TCGGCGGCGG	TCGGCGGCGG	TCGGCGGCGG	T(T)	LOC_Os07g10610	correct
Deletion3	Chr7:23797125	TG	TG	T	T(T)	LOC_Os07g39700	correct
Deletion4	Chr7:23797127	CTGGA	CTGGA	C	C(C)	LOC_Os07g39700	correct
Deletion5	Chr7:5823862	TG	TG	T	T(T)	LOC_Os07g10730	correct
Deletion6	Chr7:5823864	GAGAAT	GAGAAT	A	A(A)	LOC_Os07g10730	correct
Delition7	Chr4:33560772	AT	AT	A	A(A)	LOC_Os04g56290	correct
Deletion8	Chr4:34457223	ATC	ATC	A	A(A)	LOC_Os04g57870	correct
Deletion9	Chr7:5507412	TACCGG	TACCGG	T	T(T)	LOC_Os07g10254	correct
Deletion10	Chr4:34457210	TAA	TAA	T	T(T)	LOC_Os04g57870	correct
Deletion11	Chr7:5277268	CA	CA	A	A(A)	LOC_Os07g09914	correct
Deletion12	Chr7:5272731	GCA	GCA	GCA	GCA(G)	LOC_Os07g09900	wrong

Note: in the colum PSRR-1, the nucleotide (s) inside of parentheses was/were identified as PSRR-1 specific SNPs or InDels; while the one(s) out side of the parentheses were determined by Sanger sequenencing. All the SNPs and InDels tested here for Bengal were correct.
